# Hidden Morphological Features in Organically Synthesized Porous Carbon Revealed through Analysis of Contradictory NMR Spectra

**DOI:** 10.1021/acsomega.6c03870

**Published:** 2026-09-18

**Authors:** Catherine Mollart, Adam Rowling, Ellena Sherrett, Michael J. G. Peach, Todor T. Koev, Yaroslav Z. Khimyak, Abbie Trewin

**Affiliations:** † Department of Chemistry, 4396 Lancaster University, Lancaster LA1 4YB, U.K.; ‡ School of Chemistry, Pharmacy and Pharmacology, 6106 University of East Anglia, Norwich Research Park, Norwich NR4 7TJ, U.K.

## Abstract

A new family of carbon-based materials named “Organically synthesised porous carbon” (OSPC) have proven to have exceptional properties as anode materials in lithium-ion batteries. Experimental X-ray photoelectron spectroscopy characterization has confirmed that the framework of OSPC-1 synthesized via two different synthetic routes, OSPC-1a and OSPC-1b, is based upon *sp* and *sp* ^3^ carbon. Strangely, however, the solid-state NMR spectra for both OSPC-1a and OSPC-1b do not appear consistent with this model. Here, we show that a dense highly interpenetrated morphology based on *sp* and *sp* ^3^ carbon can fully rationalize the solid-state NMR spectra of both OSPC-1a and OSPC-1b. We suggest that this structure is dominant, but that the porosity arises from a newly uncovered morphology with an open noninterpenetrated highly porous OSPC-1 framework structure best approximated by the topological features present in the *dia*, *pts-c*, and *unc-c* topologies. This impacts the conductivity and ion storage, properties of OSPC materials and so it is imperative that this is considered when designing new OSPC materials with targeted properties for potential applications.

## Graphical abstract

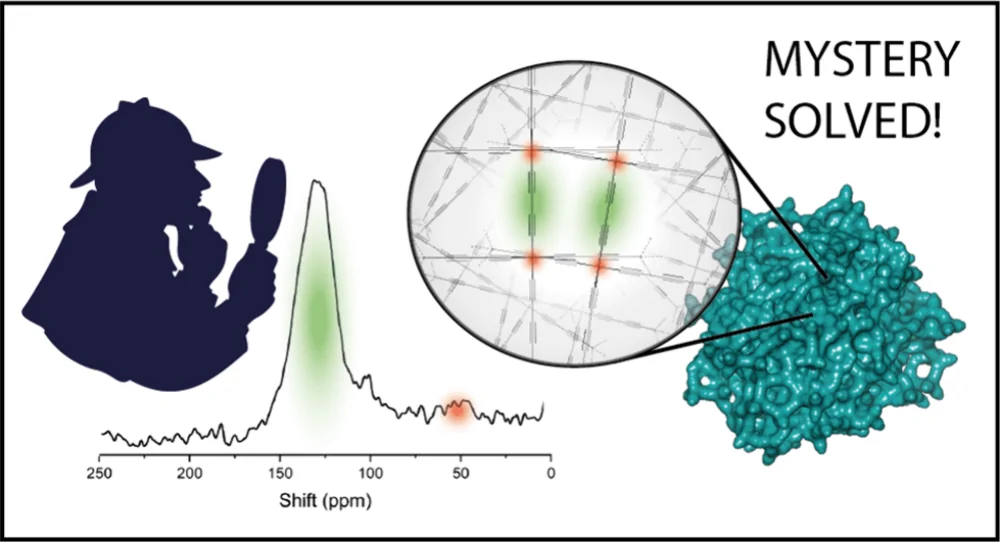

## 1 Introduction

“Organically synthesized porous carbon 1” (OSPC-1), shows great potential as a future electrode material in ion batteries as shown in our previous work.^1,2^ OSPCs fall into the diverse class of materials called microporous organic polymers (MOPs). Other materials included in the MOPs class are hyper-cross-linked polymers (HCPs),^3^ conjugated microporous polymers (CMPs),^4,5^ polymers of intrinsic microporosity (PIMs),^6^ and covalent triazine-based frameworks (CTFs).^7^ OSPCs sit within the subclass of CMPs with applications in gas uptake,^8^ solid-state electrolyte for fuel cell technologies,^9^ and as a nano porous supercapacitor.^10,11^ We note that while the framework of OSPCs is built entirely with carbon atoms, like diamond or graphite and other carbon materials, its synthesis and properties are closer to a porous polymer and it fits firmly within the MOP family. It has been synthesized via two different routes; the first route reported was via a catalytic Eglinton homocoupling of tetraethynylmethane (Figure 1(a), the catalytic mechanism and details are shown in Figure SI.1­(a) and SI.2­(a)) and is known as OSPC-1a.^1^ The second route is through a one-pot strategy via a continuous nucleophilic condensation reaction from commercially available starting materials that allows synthesis on a larger scale and is known as OSPC-1b, (Figure 1(b), the catalytic mechanism and details are shown in Figure SI.1­(b) and SI.2­(b)).2 The simpler one-pot synthesis strategy opens the door to a wide variety of potential chemical functionalization and the scope for discovery of new materials with exceptional properties is vast.

**Figure 1:**
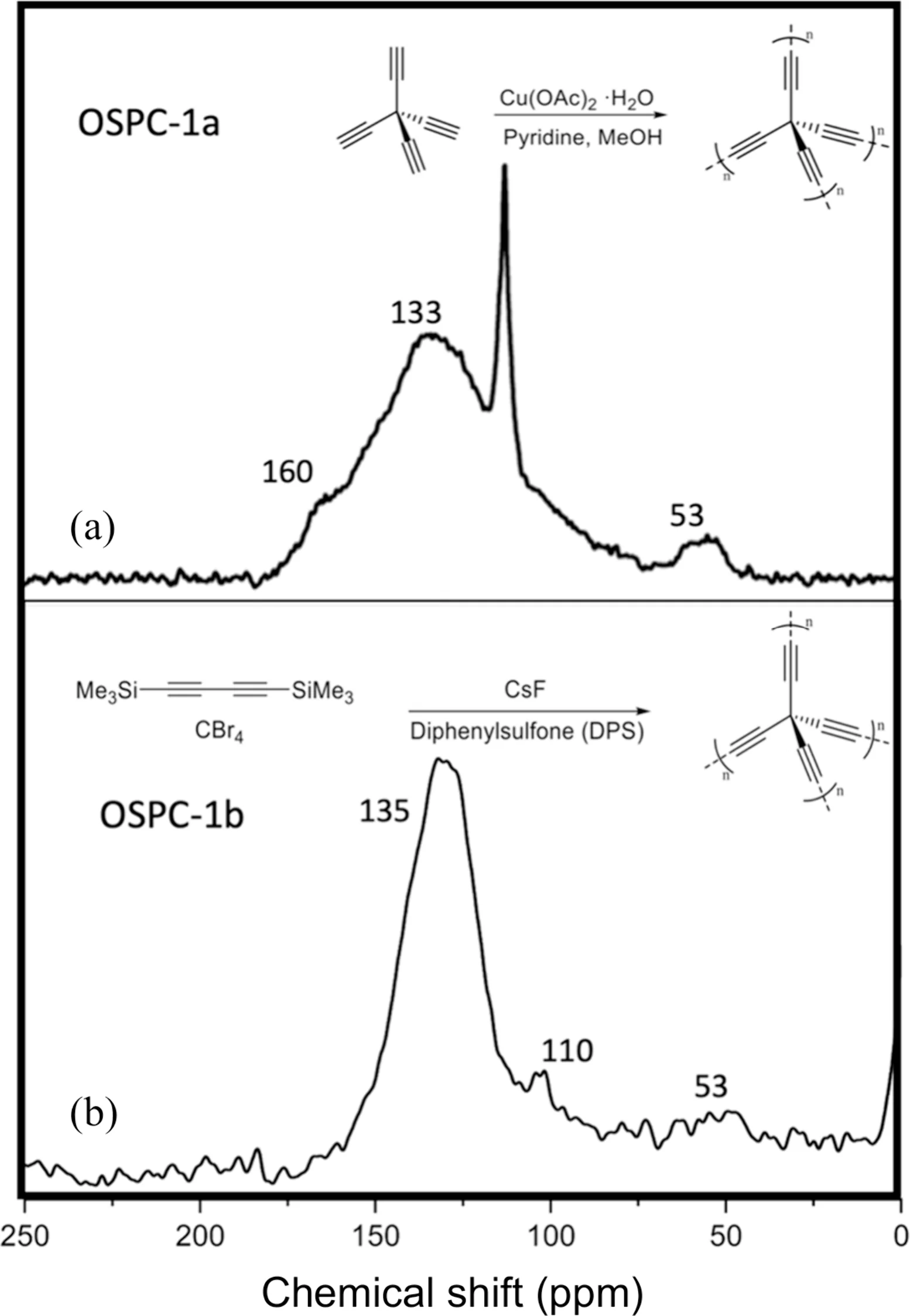
Experimental NMR spectra of (a) OSPC-1a^1^ and (b) OSPC-1b^2^ with the respective synthetic methods shown as inset. Due to XPS data (See Figure SI.3), the peak at ∼ 133–135 ppm is assigned as a C­(*sp*) and the peak at 53 ppm is assigned as a C­(*sp* ^3^). However, these regions are more usually assigned to C­(sp^2^). We aim to resolve this contradiction here and rationalize the observed chemical shifts for these carbon environments. The peaks at 160 ppm in (a) and 110 ppm in (b) were not assigned. The sharp peak at ∼ 110 ppm in (a) is due to the equipment used. See Figure SI.8 for further details.

OSPC-1a was found to have a high Brunauer–Emmett–Teller surface area (SA_BET_) of 766 m^2^ g^–1^, increasing to 909 m^2^ g^–1^ for OSPC-1b. As an anode in a lithium-ion battery, a high lithium-ion uptake of 748 mAh g^–1^ for OSPC-1a and 640 mAh g^–1^ for OSPC-1b (after 5 cycles) was found, more than the amount of lithium taken up by graphite anodes (∼340 mAh g^–1^) in state-of-the-art lithium-ion batteries (LIBs). Furthermore, both OSPC-1a and OSPC-1b demonstrated an ability to be charged at a high rate without any signs^1,2^ of detrimental lithium plating or dendrite formation that can cause explosions of devices.

The resulting structure of both OSPC-1a and OSPC-1b is amorphous as determined through powder X-ray diffraction (PXRD). Trewin et al. produced models of OSPC-1 using an in-house developed code, Ambuild, that replicate experimentally observed structural and gas uptake properties.^1^ These models were based upon a repeating carbon tetrahedral node linked by butadiynyl linkers, shown in Figure 1, common to both OSPC-1a and OSPC-1b. The chemical composition was confirmed through X-ray photoelectron spectroscopy (XPS) (Figure SI.3­(a)), which verified the presence of C­(*sp*), C­(*sp* ^3^) for both OSPC-1a and OSPC-1b, alongside very small amounts of oxygenated carbon for OSPC-1a, and small amounts of bromine for OSPC-1b, proving that the OSPC-1 material is a new carbon allotrope whose structure can only be rationalized as the proposed model. A further member of the OSPC family, OSPC-0, has also been reported where the C­(*sp* ^3^) are linked via one ethynyl linker, shown in Figure SI.1­(c). Similarly, XPS data verified the presence of C­(*sp*) and C­(*sp* ^3^) for OSPC-0b, shown in Figure SI.3­(c). Raman spectroscopy for OSPC-1a, OSPC-1b, and OSPC-0b were collected with modeling confirming that the resulting spectra were consistent with models based on just C­(*sp*) and C­(*sp* ^3^).^1,2^

Strangely, however, the solid-state NMR spectrum for both OSPC-1a and OSPC-1b (shown in Figure 1 and in Figure SI.4­(a)) and for OSPC-0b (shown in Figure SI.4­(b)), do not appear to be consistent with this model. For all OSPC NMR spectra, there are broad peaks centered at 56 and 133 ppm, consistent with an amorphous material, but these peaks would typically be assigned to C­(*sp* ^3^) and C­(*sp* ^2^) respectively. The solid-state NMR peak positions of some common C­(*sp*), C­(*sp* ^2^), and C­(*sp* ^3^) containing materials are shown in in Table SI.1 and Table SI.2.^12–27^ The range of peak values observed for C­(*sp* ^3^) is broad and covers the region of the first peak and part of the second broad peak. The range of peak values observed for C­(*sp* ^2^) is narrower and is centered on the second peak observed in the OSPC NMR spectrum at 133 ppm. For C­(*sp*), three ranges are seen, of which only one range covers the lower part of the second peak. An initial assessment of the experimental NMR spectra, without knowledge of the synthetic routes or additional characterization, would result in an assignment of the material as containing large amounts of C­(*sp* ^2^), similarly to graphitic materials including amorphous carbon and hard carbons.

Characterization through XPS is highly reliable and very commonly used to identify carbon hybridization composition of materials.^28^ It is extremely specific and can be used to detect very small differences in ratios between different carbon hybridization states.^28–30^ However, deconvolution between *sp* and *sp* ^2^ components is well recognized as challenging. The binding energy difference between these state is small (∼0.1–1.0 eV) and the resulting peak overlap means that the extracted ratios are sensitive to the choice charging correction, background subtraction method, and assumed peak shape parameters. The deconvolution reported for OSPC-1b, yields an approximate surface *sp* ^3^ *:sp* ratio of 55:45 across a series of samples, though the amorphous nature of the material produces inherent sample to sample variability. Further, the high surface specificity of XPS means that this is not representative of the bulk polymer due to the expected high concentration of sp^3^ C end groups at the surface. Taking these aspects into account, we estimate an upper bound of approximately 14% on any undetected *sp* ^2^ contribution at the surface. This upper bound is insufficient to account for the upfield shift observed in the ^13^C solid-state NMR spectrum, indicating that the NMR anomaly cannot be attributed to unresolved *sp* *^2^* carbon content. This contradiction between the initial solid-state NMR spectrum peak assignments and the XPS characterization data poses a conundrum that demands a detailed investigation to rationalize and resolve this challenge. Here we verify the NMR results and show that through calculation of the NMR spectra for models of OSPC-1a and for OSPC-1b, that take into account the amorphous structure, synthetic mechanism, and potential end groups, we can rationalize the observed NMR spectrum and hence confirm the OSPC structure.

## 2 Results and Discussion

### 2.1 Repeated NMR Experiment

OSPC-1 has been synthesized via two different chemical reaction routes giving very similar NMR spectra. These synthetic routes leave little opportunity for other chemical transformations that may account for the unexpected NMR spectra. To further confirm the validity of the obtained spectra, we rerun the experiment for OSPC-0b and OSPC-1b on a separate machine in a different institution and collected ^13^C spectra. The resulting spectra are shown in Figure SI.5 and Figure SI.6 respectively, and are similar to those previously collected, shown in Figure 1. This confirms that the anomalous results are due to the inherent material itself. Additionally, we collected the ^29^Si {^1^H} spectra for OSPC-1b, which strangely shows two distinct ^29^Si environments (Figure SI.7).

### 2.2 Starting Material

The synthesis of OSPC-1a is via the Eglinton coupling of tetraethynylmethane, which is produced *in situ* from a triethylsilyl (TES) protected form.^1^ The tetraethynylmethane is not isolated and so its NMR spectrum has not been collected. The ^13^C NMR spectrum of the TES-protected tetraethynylmethane is shown in Figure SI.9 alongside the calculated ^13^C NMR spectra in Figure SI.10. In both spectra we see five peaks at ∼ 100 ppm (C­(*sp*)), 82 ppm (C­(*sp*)), 24 ppm (C­(*sp* *^3^*)), 7 ppm (TES) and 4 ppm (TES) with a good match between experimental and calculated spectra. We use this spectrum to calibrate subsequent calculated spectra using a shift equation determined to align the experimental and calculated spectral peak positions. A shift is determined individually for the Gaussian and CASTEP determined spectra. The calculated spectrum of tetraethynylmethane is shown in Figure SI.11 with three peaks at ∼ 85 ppm (C­(*sp*)), 75 ppm (C­(*sp*)), and 27 ppm (C­(*sp* *^3^*)), reflecting the three distinct chemical carbon environments.

### 2.3 Fragment Models

The smallest OSPC fragment is a dimer constructed from two tetraethynylmethane units bonded through one strut, as shown in Figure SI.12. This 2-node OSPC-1 fragment exhibits a similarly shaped spectrum to the tetraethynylmethane but has two additional peaks at ∼ 81 and 76 ppm, assigned to the C­(*sp*) of the strut.

Figure 2(a) shows a larger 5-node tetrahedral OSPC-1 fragment and the resulting calculated NMR spectrum. This fragment is representative of the overall OSPC-1 repeat unit. The spectrum has a broadly similar shape to the experimental spectrum with a set of peaks of a narrow range at low ppm that result directly from the C­(*sp* ^3^), and a set of peaks with a broad range at higher values. The peaks at lower values result directly from within the model and the broad set of peaks at higher values are directly attributed to C­(*sp*) within the model. However, the peak positions do not match well to those observed experimentally being at ∼ 38 ppm and 70–80 ppm.

**Figure 2:**
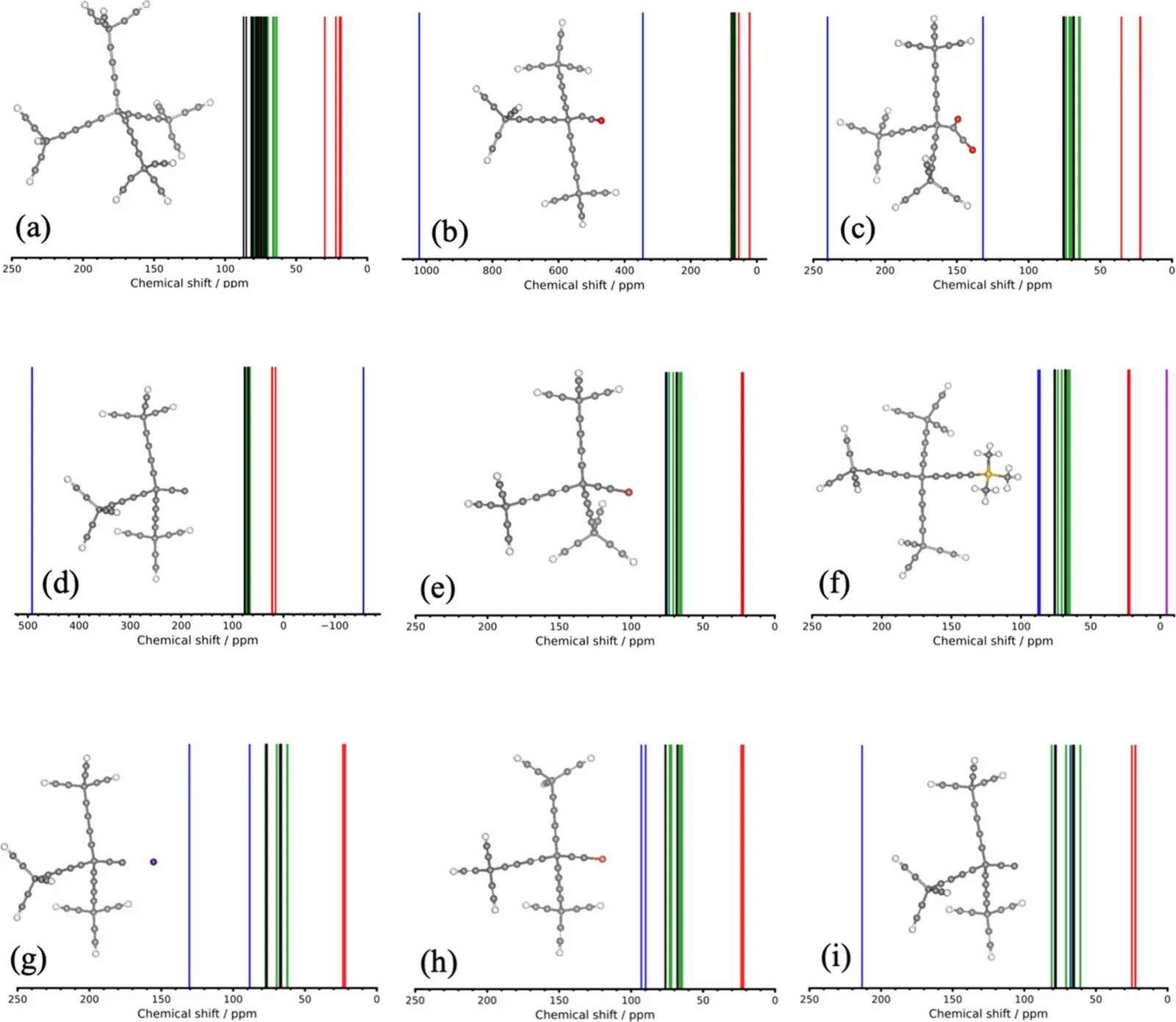
Fragment models and the calculated NMR using Gaussian of (a) OSPC-1 and the most likely OSPC-1a oxygenated surface species (b) ketenyl radical, (c) oxo-ketene, (d) radical and OSPC-1b end groups, (e) bromine, (f) tetramethylsilane, (g) OSPC-1 anion with a Cs in close proximity, (h) OSPC-1 anion with a Cu in close proximity, (i) OSPC-1 anion. Key: green – *sp* carbon in saturated OSPC chain, red – *sp* *^3^* carbon node, black – *sp* carbon in unsaturated alkyne, blue – *sp* carbon in saturated chain between OSPC-1 and end group, purple – *sp* *^3^* carbon in TMS end group.

As OSPC-1 is a kinetic product, it is possible that the tetrahedral nodal structure could adopt a range of structures resulting from diversity in the angles of the struts around the node. To assess the influence of this angle on the NMR spectrum, an angle scan was performed, varying one of the strut-node-strut angles systematically taking values between 60° and 160°. The resulting potential energy curve in Figure SI.13 shows a clear minimum at 109.5° and a relatively shallow curve with an energy penalty of ∼ 5 kJ mol^–1^ when distorted by ± 20°. At each angle the NMR spectrum was calculated, examples of which are shown in Figure SI.14. We can see that there is a slight shift downfield shift as the angle varies but no significant differences.

Due to this inherent flexibility of the strut-node-strut angle, it is possible for a range of framework macrocyclic ring (MCR) features to form. Figures SI.15–SI.18 show a 3-node, 4-node, 5-node, and 6-node OSPC-1 macrocyclic rings, and the respective resulting calculated NMR spectra. For the 3-node MCR, peak 1 is at 29 ppm, a slight shift upfield to 28 ppm is observed for the 4-node MCR. For the 5-node and 6-node MCRs, a large shift downfield is observed for peak 1. Going from the 2-node model through to the 6-node model, we can see that the peak positions do shift, particularly the peak assigned to the C­(*sp* ^3^). In the 2-node model this peak is located at 28 ppm whereas in both the 5-node model and the 6-node model the peak shifts to 38 ppm.

Fragment models that are representative of the OSPC-1 structure are required in order to reliably determine the origins of the observed NMR spectral features. Hence, it is important to assess what terminating end groups may also be present within the material. The mechanisms, outlined in Figure SI.2, shows that for OSPC-1a the terminating hydrogen atom is removed and replaced with a copper atom which is itself subsequently removed to leave a carbon radical terminating group. Two of these radicals can react together to form the OSPC-1 strut. Any unreacted radicals can either: be protonated to return to the ethynyl group, remain within the structure, or can react with atmospheric oxygen to form oxygenated species, most likely a ketenyl radical­(−CCO) or an oxo-ketene – C­(O)­CO.^31^ Therefore, the possible terminating groups for OSPC-1a are an ethynyl (i.e., unreacted group), a copper atom, a carbon radical, – CCO, and – C­(O)­CO. Experimental elemental analysis and XPS data (Figure SI.3) indicate that some oxygen is present in OSPC-1a but there is no indication of any copper being present.^1^

For OSPC-1b, the terminating TMS group of a bis­(trimethylsilyl) butadiyne is removed and replaced with a cesium, which can then react with a carbon tetrabromide. This repeats until all bromine atoms and TMS groups have reacted to form the OSPC-1 network. Therefore, the possible terminating end groups for OSPC-1b are a TMS, a bromine, and a cesium. Figure 2(b)–(i) show the structure and resulting NMR spectrum for a 4-node OSPC-1 fragment with one ethynyl group terminated with the potential end groups for OSPC-1a and OSPC-1b. In all cases, there is a clear link to the experimental spectra obtained for OSPC-1a or OSPC-1b.

Electron paramagnetic resonance (EPR) spectra collected for a similar material to OSPC-1a, Si-DA,^32^ which has the same underlying framework topology and linking struts but where the nodal carbon is replaced by a silicon atom, Figure SI.8, identifies the presence of radicals within the framework structure. These radicals could be end groups remaining from the synthetic mechanism or they could be a biradical resonance form of the butadiynyl struts, as suggested by Dong et al. shown in Figure SI.4.^32^ If the radical originates from resonance structures, then these should also be present in OSPC-1b. However, if they originate from remaining terminating end groups then these will not be present in OSPC-1b as it is synthesized via an alternative route. There may still be a background of spins present in OSPC-1b from side reactions or other sources, which would be present in the EPR spectrum due to the high sensitivity of the technique. OSPC-0 is similar in structure to OSPC-1 but has one alkyne group connecting the carbon nodes, so cannot adopt the resonance structure of Si-DA. OSPC-0b is synthesized through the same reaction under the same conditions as OSPC-1b, therefore, the spin background of OSPC-0b should be the same as OSPC-1b. To assess whether radicals are present in OSPC-1b, an EPR spectrum was collected, shown in Figure 3. If OSPC-1b adopted the resonance structure to any significant extent, we would observe a signal well in excess of any signal observed in OSPC-0. No significant difference was observed between OSPC-1b and 0b, and both samples produced a significantly smaller signal than a standard sample of strong pitch, a known radical source. We can conclude that OSPC-1b does not adopt the radical resonance structure, and it is likely that any radicals present in OSPC-1a will originate from terminating groups.^33–36^ Also, we do not see hyperfine splitting in our spectra, which would be expected for the proposed biradical resonance structure. This is also absent in the reported EPR spectra of Si-DA (Figure SI.8).^32,37–39^ Similar terminating groups could be present in Si-DA so it would be difficult to confirm that the radicals observed are from the resonance structure. An estimate of the proportion of end groups per mole combined with quantitative EPR of Si-DA would show if there are significantly more radicals present than end groups, which would indicate that Si-DA adopts the proposed resonance structure.^32^ Radicals are extremely difficult to account for in calculated NMR spectra due to the long-range nature of the interactions. Table SI.1 shows that most radicals appear in the experimental spectra in the 65–105 ppm range. We therefore believe that they contribute to the observed spectrum of OSPC-1a, but do not account explicitly for the large broad peak centered at 133 ppm. They may, however, influence the peak positions of the local framework, which potentially offers one route to rationalize the spectrum of OSPC-1a in the absence of an alternative explanation. As the NMR spectra that result from the simple fragment models, even accounting for potential end groups, do not adequately rationalize the observed experimental spectra, we now look to more complex structural features.

**Figure 3:**
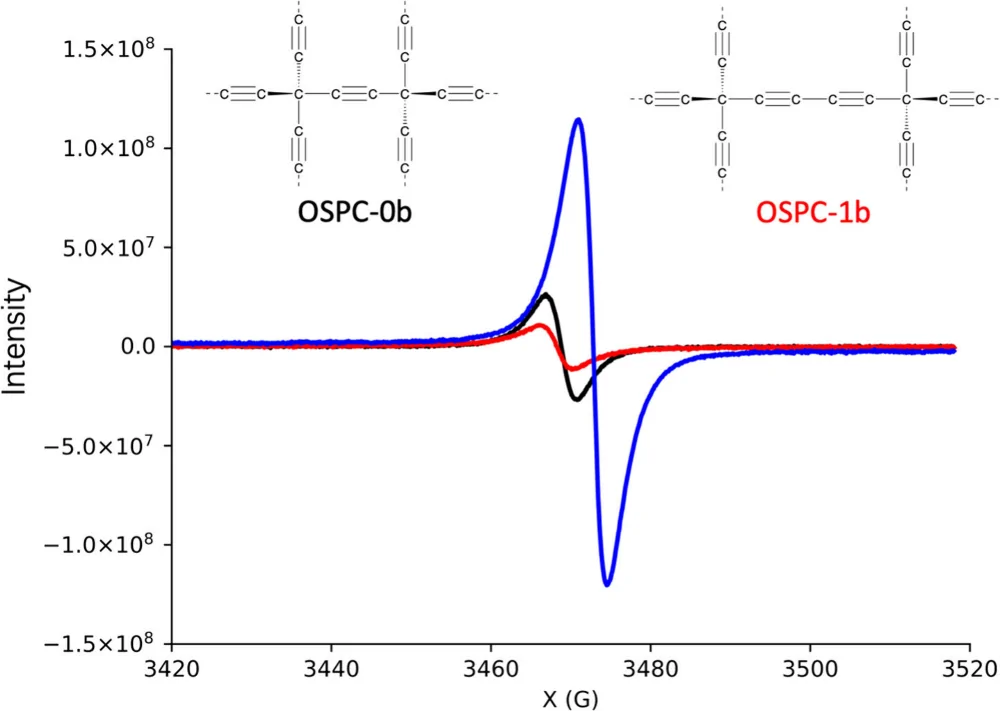
EPR of OSPC-1b and OSPC-0b. OSPC-1b = red, OSPC-0b = black, strong pitch (known radical source) = blue.

### 2.4 Periodic OSPC-1 Models

#### 2.4.1 Crystalline Models

OSPC-1 is an amorphous framework material with no sign of order within the structure.^1^ We can, however, use crystalline models of OSPC-1 to assess the relative energies of different models, framework interactions, and their resultant NMR spectra. Figures SI.23 to SI.29 show the starting model topology and the resulting predicted crystal structure and associated NMR spectra of a set of crystalline topologies that OSPC-1 can adopt. The chosen topologies all contain a tetrahedral node and are a mix of noninterpenetrating and interpenetrating frameworks to varying degrees. Figure 4(a) shows the energy of the crystalline set plotted against the respective density. We can see that in general, the energy decreases as we move to less dense models with the *dia-c* topology being the lowest energy model but with only a small increase in energy to the *dia* and *dia-c6* models. This is unusual, as in general energy density plots tend to approach a minimum energy for high densities. This suggests that it is unfavorable for the OSPC-1 framework to self-interact and so more open structures are favored over the range of densities assessed here.

**Figure 4:**
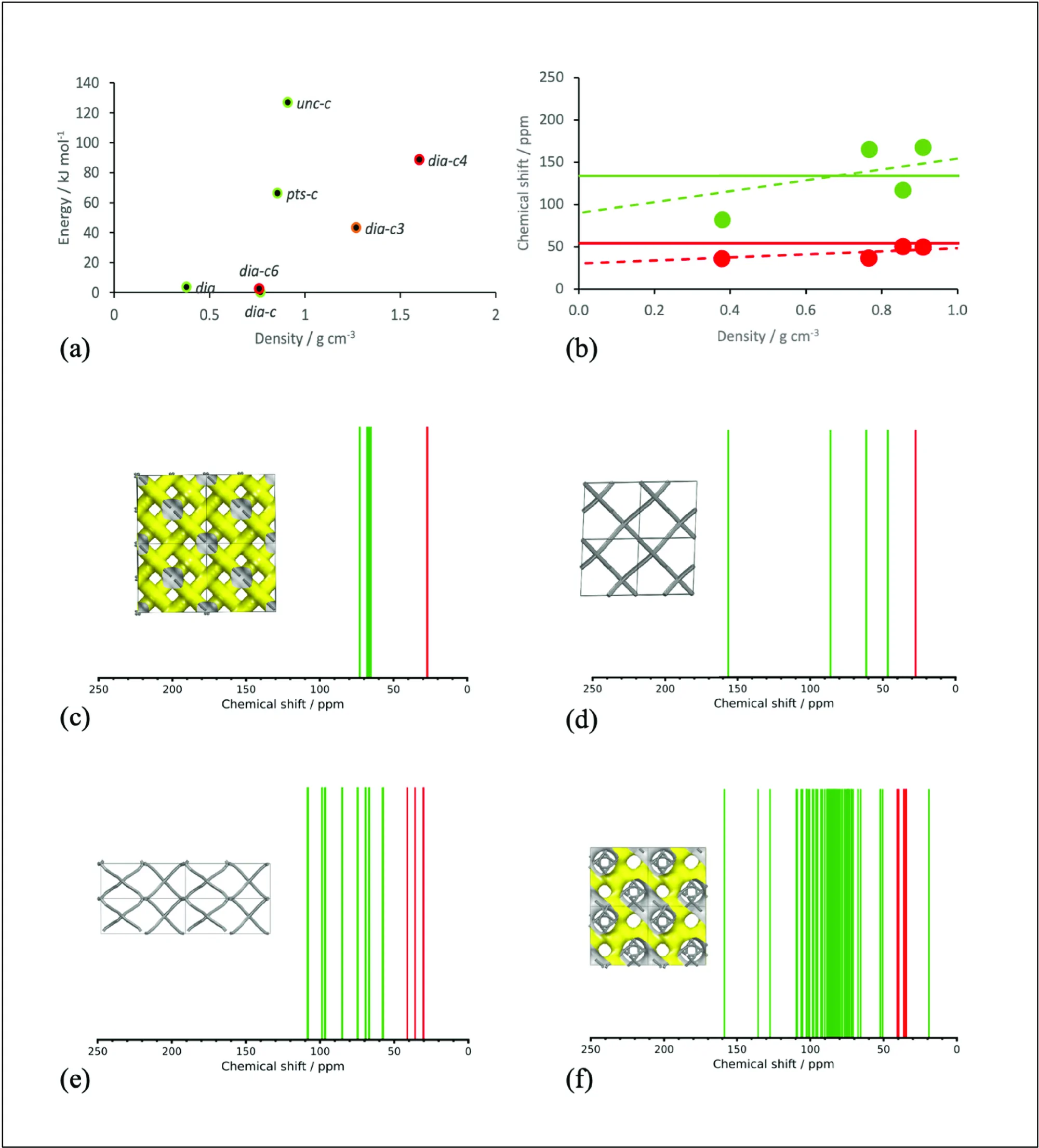
Periodic crystalline models and the NMR, Structure optimization and NMR calculated using CASTEP. (a) plot of normalized energy per t-site against density. Key: Green – keeps OSPC-1 motif, orange – shows some changes but overall keeps motif, red – shows breakdown of structure. (b) Density against the highest chemical shift peak of *sp* (green) and *sp* ^3^ (red) carbon atoms for dia, dia-c, pts-c, and unc-c models. The solid green and red lines are the experimental values assigned to the *sp* and *sp* ^3^ carbon, respectively. The dashed green and red lines are lines of best fit with the origin at 90 and 30 ppm for *sp* and *sp* *^3^* carbon respectively (the approximate respective values observed from the calculated NMR spectra of the proposed fragment models). NMR spectra of OSPC-1 in the (c) dia, (d) dia-c, (e) pts-c, (f) unc-c topologies. (b)–(e) key: green – sp carbon in saturated OSPC chain, red – sp^3^ carbon node. Surface accessible surface shown in yellow generated using a probe radius of 1.4 Å.

Assessing the resulting structures, we can see that only the *dia, dia-c*, *pts-c,* and *unc-c* have retained the expected OSPC-1 network bonding motif. The *dia-c3* model retains the overall network structure but with some bonding rearrangement, whereas *dia-c6* and *dia-c4* exhibit a breakdown of the network structure to layered structures with MCR structures observed. This suggests a complex relationship between density, topology, and energy with potential for framework interactions to result in structural changes.

The resultant NMR spectra for the *dia, dia-c*, *pts-c,* and *unc-c* models are shown in Figure 4(b)–(e) respectively. For the *dia* model we see an NMR spectrum that is very similar to that observed for the OSPC-1 fragment models. For the *dia-c* model we see some shift of peaks to higher values and a new peak at ∼ 160 ppm. For the *pts-c* model we see a spread of peaks between ∼ 30 and 110 ppm. For the *unc-c* model a broad spectrum is observed between ∼ 20 and 160 ppm. The NMR spectrum from the low density *dia* structure does not match well to the experimentally obtained NMR spectra. For the *dia-c*, *pts-c,* and *unc-c* structures, however, there are some features that do match to the experimentally obtained NMR spectra. For example, the broadened range of peak values particularly to higher ppm values.

The solvent accessible surface areas for the *dia, dia-c*, *pts-c,* and *unc-c* model topologies were calculated and are shown in Table SI.3 with the surfaces are shown in the insets for Figure 4 (b)–(e). The *dia* model shows a particularly high solvent accessible surface area of 6428 m^2^ g^–1^. The *unc-c* shows a high solvent accessible surface area of 1295 m^2^ g^–1^. However, the *dia-c* and *pts-c* models show zero surface area. None of these models represents a good approximation of the experimentally determined BET surface areas of ∼ 700–900 m^2^ g^–1^.

However, this does give some clues as to the origin of the experimental NMR behavior. Overall, the *unc-c* model has an NMR spectrum that is the closest to the experimentally observed spectra, while *dia* is the least close match. Table SI.3 shows the closest linker–linker carbon distance from each model. The *dia* model has the largest linker C–linker C distance at 11.02 Å and the *unc-c* model has the smallest distance at 2.76 Å. This suggests that close linker–linker interactions play an important role in the NMR spectra obtained. For materials that result from chemistry that is reversible, the thermodynamic product is expected and is likely to be crystalline. Here, we have not fully explored the crystalline structural space as our material forms through irreversible chemistry and so the resulting network is amorphous.^40,41^ The crystalline models have however given us valuable insight into the structural motifs and interactions that may be present that we can now explore further.

#### 2.4.2 Chain Models

We would normally expect that interactions between nuclei do not affect the NMR behavior beyond a few “bond-lengths”. However, OSPC-1 shows extensive conjugation throughout the system, and we observe that linker–linker interactions do influence our calculated NMR spectra. To systematically control these long-range interactions, we assess the calculated NMR properties of periodic models of an OSPC-1 chain as a function of cell length, shown in Figure SI.30. A simple model was constructed comprising one chain connecting the nodal carbons via two of the four ethynyl linkers. The remaining two groups were initially set as hydrogen terminated ethynyl groups, to best approximate the radical terminated end groups in OSPC-1a and with the chain carbon atoms being relevant to both OSPC-1a and OSPC-1b, giving the *ethynyl end groups* models. These are necessary for the simulation to function, but we do not expect to see protons in the real-world structure. We assess two further sets of models: one with the two nonchain positions instead as bromine atoms, the *bromine end groups* models, and one where there is one bromine atom and one ethynyl group capped with a TMS group, the *TMS-Br end groups* models.

The cell length was initially chosen to inhibit any linker–linker interactions. The *y-* and *z-*cell lengths were then systematically reduced. The chain runs in the *x-*direction and so the cell length in the *x-*direction is fixed by the chain bonding. The calculated NMR spectrum of the initial periodic OSPC-1 ethynyl end group chain model, Figure SI.30, shows a similar shape to that of the nonperiodic models with a peak at 34 ppm, resulting from the C­(*sp* ^3^), and a set of peaks between 74 and 81 ppm, resulting from the C­(*sp*). A further ethynyl end group chain model is assessed where there are two chains within the unit cell with one chain running in the *x*-direction and the second chain running in the *z*-direction. In this case only the *y*-cell length is changed.

Tables SI.4–17 and Figures SI.31–44 show the structural models assessed and the energy profiles and SI Section 9 shows the NMR spectra for a selection of the models. For set 1, where the cell length in the *y*-direction is decreased systematically, we see that the energy increases to a maximum at *y*=∼3.1 Å followed by a sharp decrease in the energy to a minimum at *y*=∼2.5 Å with a steep increase at smaller *y* values. Assessing these structures, we can see that the OSPC-1 structure breaks down, similarly to the structures observed for the *dia-c6*, *dia-c3*, and *dia-c4* topological models.

For sets 2–10 we fix the *y*-cell length to values between 3.5 Å and 3.1 Å and systematically decrease the *z-*cell length. In all cases we can see that there is a decrease in energy until *z* reaches ∼ 7.0 Å followed by a sharp increase in energy. For set 11, we fix the *z* cell length to 6.0 Å and systematically decrease the *y* cell length. Here, the energy decreases to a broad minimum between ∼ 2.4 Å and 2.5 Å followed by an increase to smaller *y* values. For set 12, where there are two chains running in the *x*- and *-z* directions, the *y*-cell length is decreased systematically. We see that the energy overall decreases to a minimum at ∼ 14 Å, followed by an increase in energy at smaller *y*-cell lengths. However, we see no breakdown of the OSPC-1 structure at smaller y-cell lengths. For sets 2–11, we observe very different peak positions in the NMR spectra as a function of the cell parameters, whereas for set 12 only a general broadening of peaks is observed.

#### 2.4.3 Energy Mapping

To assess the single chain models further, Figure SI.45 maps the energy going from the least dense model to the densest via the lowest energy structures at each step. We can see that there is a large increase in energy for any increase in density, reflecting the energy profile observed for the crystalline models shown in Figure 4(a). At higher densities, there is a minimum between model MF (*y* = 3.12 Å and *z* = 6.9 Å) and model MT (*y* = 3.10 Å and *z* = 6.0 Å). Model MT is the densest structure in which the OSPC-1 chemical structure remains intact. To explore the nature of this energy region further, we undertake calculations of the interaction energy of OSPC-1 fragments. First, we look at two dimers of OSPC-1 with the distance between two carbon atoms on each fragment changed systematically. Figure SI.46 shows the energy profile as a function of distance between either two linker carbon atoms or a linker and a node atom. In each case we see that a shallow energy well is observed between ∼ 9 Å and 7 Å distance. This does not correlate well with what is observed for the periodic chain systems and so we increase the number of dimers to three. Figure SI.47 shows the energy profile for three dimers stacked on top of each other with the distance measured between the outer dimer linker carbon atoms. Here, we see a very similar energy profile to the periodic chains with the energy increasing to a C–C distance of ∼ 4 Å, followed by a minimum at ∼ 3.9 Å, followed by a further maximum at which point the OSPC-1 structure breaks down in a similar way to that previously observed. Figure SI.47­(c) shows the energy of six structures labeled **a** to **f**. The NMR spectra for these fragment structures are shown in SI section 9. The NMR spectra show a broadening of peak distributions and peak shifting to higher values, similar to the NMR spectra of models MF to RT.

It is reasonable to assume that within the amorphous OSPC-1 material, a range of the structures within this minimum may exist. As such, we have assessed the NMR spectra for structures MF to RT, shown individually in SI section 9. Figure 5(a) shows the NMR spectra for MF to RT plotted together. We see four distinct sets of peaks (SI section 10): (i) Peak A in the range ∼ 80–140 ppm, originating from the *sp* carbons with most peaks being at ∼ 135 ppm, (ii) Peak B in the range ∼ 50–60 ppm, originating from the *sp* *^3^* carbons with most peaks being at ∼ 57 ppm, (iii) Peak C in the range ∼ 165–180 ppm, originating from the ethynyl end group *sp* carbons with most peaks being at ∼ 170 ppm, and (iv) Peak D in the range ∼ 80–110 ppm, originating from the ethynyl end group *sp* carbons with most peaks being at ∼ 100 ppm.

**Figure 5:**
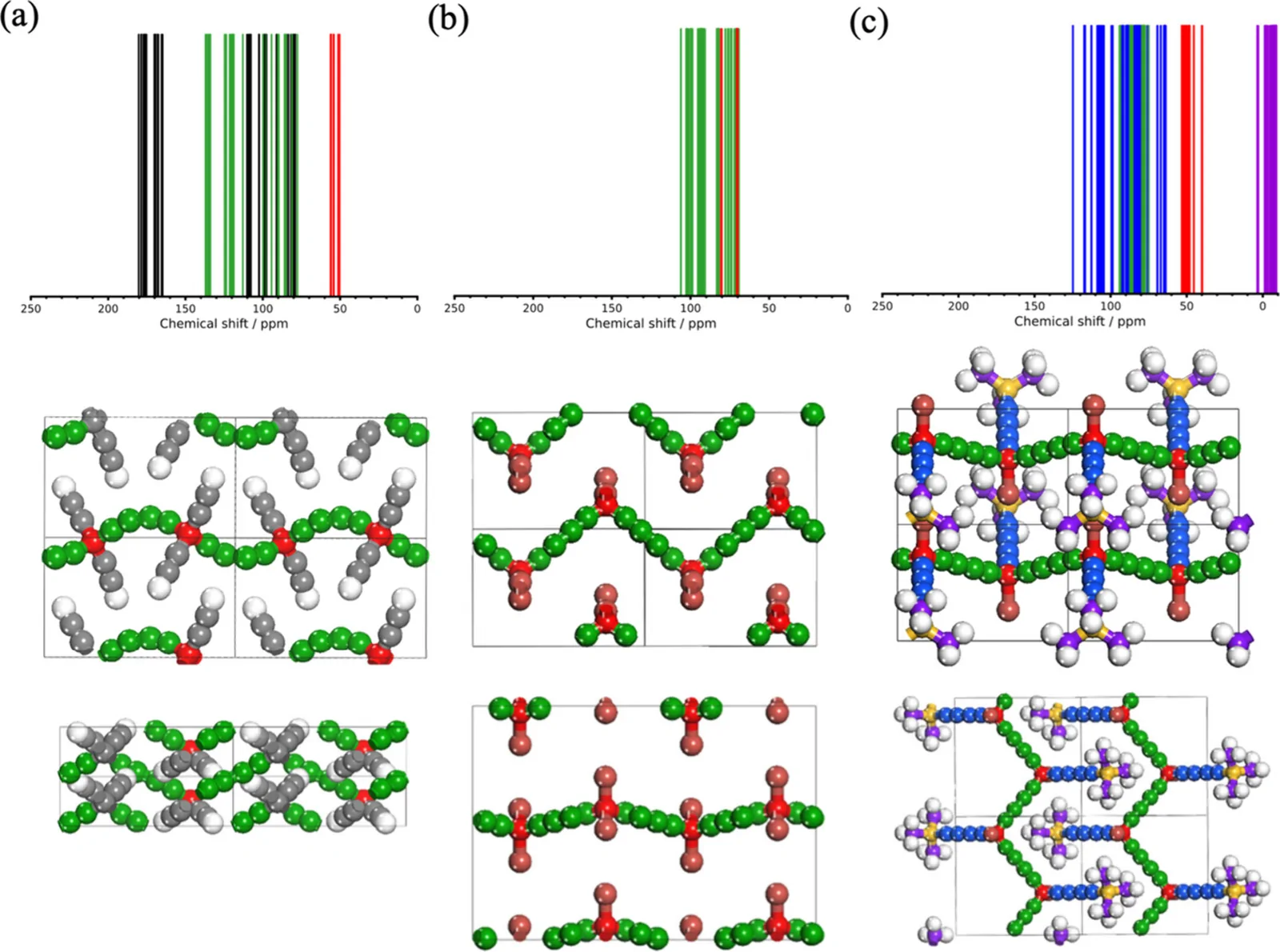
Calculated NMR spectra for (a) ethynyl end groups models MF–RT, (b) bromine end groups models AT–BF, and (c) TMS-Br end groups models BG–BQ. The NMR peaks are color coded to show the carbon atoms from which they originate in the respective example model structure shown below the NMR spectra. Key: green – *sp* carbon in OSPC chain, red – *sp* *^3^* carbon node, black – *sp* carbon in ethynyl end group, blue – *sp* carbon in ethynyl TMS end group, purple – *sp* *^3^* carbon in TMS end group.

Similarly, energy mapping of the Br-, and TMS-Br end group periodic chain models show an energy minimum is found as the chains become closer together and the cell volume is decreased (Tables SI.16 and 17 and Figures SI.43 and 44). These systems are unable to pack as densely as the ethynyl end group models due to the bulky size of the end groups. However, we are able to assess the resulting NMR data for a range of these structures and therefore identify the resulting peaks sets. Figures 5(b) and (c) shows the NMR spectra for the collected models for the bromine end group models and the TMS-Br end group models, respectively. We can identify four additional carbon peak sets (SI section 10): (v) Peak E in the range ∼ 70–80 ppm, originating from the *sp* *^3^* carbon bonded to Br, (vi) Peak F in the range ∼ 80–125 ppm, originating from the *sp* carbons in the ethynyl group with a TMS end group with most peaks being at ∼ 110 ppm, (vii) Peak G in the range ∼ −10–5 ppm, originating from the *sp* *^3^* carbons in the TMS end groups with most peaks being at ∼ 3

ppm, and (viii) Peak H in the range ∼ 40–50 ppm, originating from the *sp* *^3^* carbon bonded to 1x Br and 1x ethynyl-TMS end groups with most peaks being at ∼ 45 ppm. Peak sets A–H can be identified in the experimental NMR spectra of OSPC-1a and OSPC-1b, shown in SI Section 10.

Having identified the origin of the main peaks in the spectra, we conclude that through-space effects arising from the close interaction between the highly conjugated *sp* chains of the OSPC framework are inducing the shift to higher values for the *sp* and *sp* *^3^* C. Through space effects such as this have been seen arising from intermolecular ring current shielding/deshielding effects resulting in shifts of up to 23 ppm.^42,43^ Here, we see that the peak attributed to the *sp* C is shifted by ∼ 40 ppm and the peak attributed to the *sp* *^3^* C is shifted by ∼ 20 ppm. The closest distances between the *sp* C atoms in the MF-RT model set are ∼ 2.8 Å, closer than that expected for through space bonding such as π-π stacking or H-bonding interactions but not close enough to be considered as chemical bonding. A similar effect is found for the acetylenic carbon shift when bound within a platinum complex where they argue that the chemical shift is due to nonlocal effects and conclude that “Chemical shift tensors of the alkenyl carbons are similar to those of an alkene but not because the alkenyl bond becomes an effective double bond in the complex”.^44^

### 2.5 ^13^C Full NMR Spectra Modeling

It is not possible to determine the absolute peak intensities of the calculated spectra and so we look to see whether we can model the experimental spectra by positioning Gaussian broadening functions at the main peak locations. We use peaks A–H, identified from the energy mapping around which to position a Gaussian function. These are shown in Figure 6 for OSPC-1a and OSPC-1b. The total plots obtained, shown in blue, demonstrate an extremely good fit to the experimentally observed spectra in both cases. The ratio of the areas under the respective Gaussian fitted peaks for each set gives an indication of the relative population of each carbon environment within the system, however, we note that this is an estimate as peak intensities may be influenced by a number of factors. For OSPC-1a, we get a ratio for peak A­(*sp*):B­(*sp* *^3^*) of 8.4:1, close to the ideal ratio expected for OSPC-1 of 8:1.^1^ The ratio of peaks A:C and D, the ratio of ethynyl linkers to end groups, is 8.4:2, suggesting that 1 in 4 ethynyl positions is an end group. However, it must be noted that the ethynyl end group NMR peak may be influenced by the specific nature of the end group. For example, if it is terminated in a carbon radical then this may influence the peak intensity.^45,46^ For OSPC-1b, it is slightly more complex as there are two different types of end group present. We get a ratio for peaks A­(*sp*):B­(*sp* *^3^*) of 9.5:1, and the ratio of peaks B­(*sp* ^3^):E­(Br linked *sp* *^3^*), is approximately 3.5:2, suggesting that approximately 1 in every 3 ethynyl positions is occupied by a Br end group. It is difficult to estimate the TMS content as only part of the peak is experimentally observed. The ratios are shown in Tables SI.18 and SI.19.

**Figure 6:**
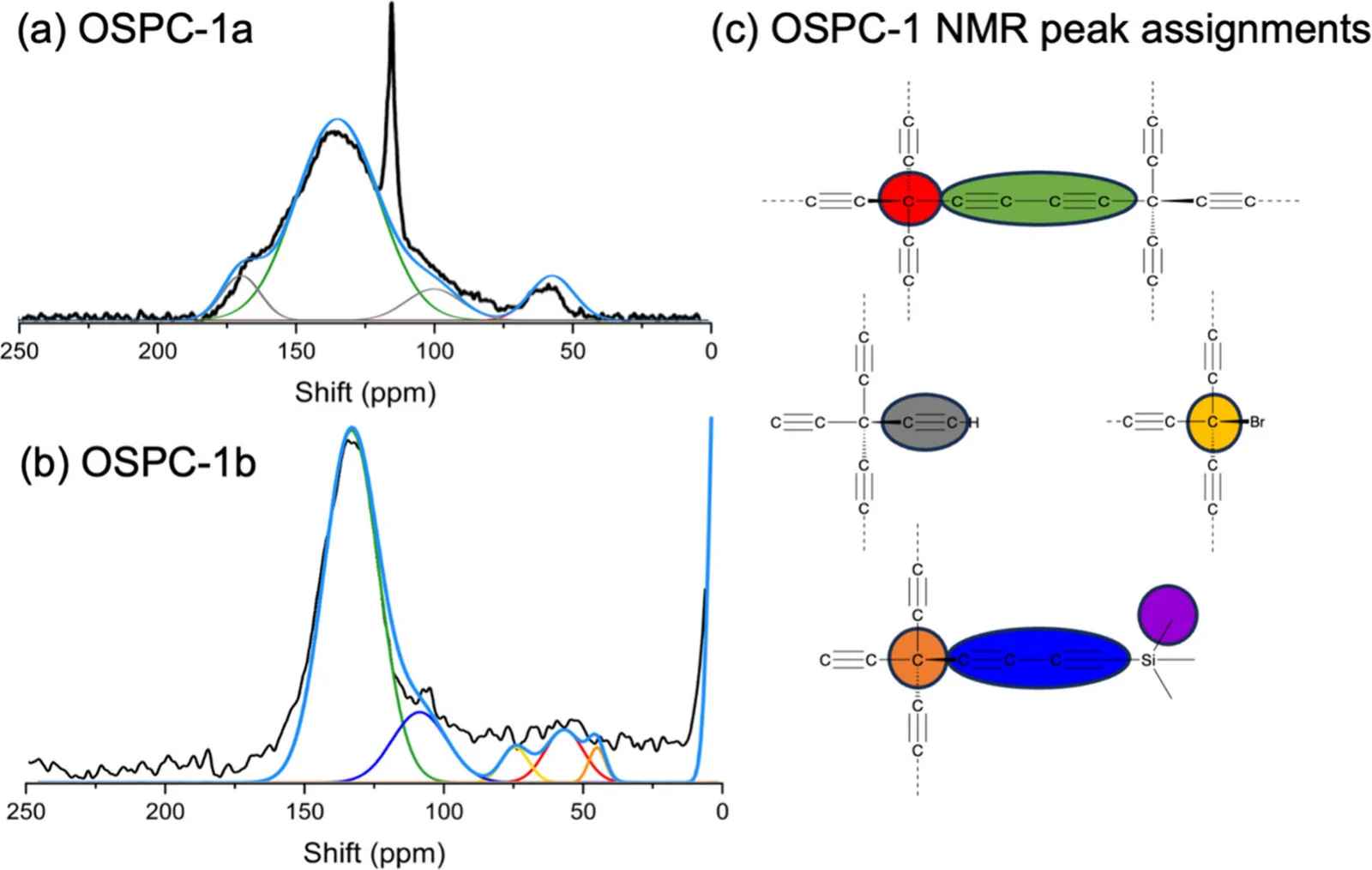
Experimental solid state NMR spectra of (a) OSPC-1a and (b) OSPC-1b compared to a modeled spectra derived from the computationally determined NMR peak positions A-H with a Gaussian function applied. The light blue line is the total modeled spectra. The individual Gaussian function assignments are shown in (c) where green is attributed to sp C in the ethynyl linking chains in the OSPC-1 framework, red is attributed to *sp* *^3^* C in the OSPC-1 framework, gray is attributed to ethynyl end groups, orange is attributed to *sp* *^3^* C attached to a TMS terminated ethynyl group, dark blue is attributed to sp C in a TMS terminated ethynyl group, purple is a *sp* *^3^* C in a TMS terminal group, and yellow is a *sp*3 attached to a Br atom. Full assignments shown in SI section 10.

This rationalization of the OSPC-1 NMR spectra characterizes the structure as being a dense nonporous system in which the ethynyl groups are close enough to shift the peaks observed. However, this is in contrast to the known porosity of OSPC-1a and OSPC-1b having measured surface areas of between 700 and 900 m^2^ g^–1^.^1^

There are two scenarios that may rationalize these observations: (1) that the porosity results from mesoporosity. However, a clear type II nitrogen uptake isotherm is obtained (with a large degree of microporosity and clear peaks within the micropore region of the pore size distribution) that discounts this scenario,^1^ and (2) A mixture of OSPC-1 structural forms are present of which only a dense form, which this model approximates, dominates the experimental NMR spectrum.

It is not possible to excite carbon in the bulk polymeric OSPC structure via cross-polarization because they do not contain any hydrogen groups. The spectra shown in Figure 1 and in Figure SI.4 have been obtained through direct excitation of the ^13^C nuclei, which can reduce the signal-to-noise ratio by up to 4 times. A cross-polarization spectrum would exclude the bulk structure as the end groups would be overly amplified and therefore hide peaks from the bulk, and provide information about the structural environments around end groups and any impurities trapped during network formation or adsorbed on the surface. As the less dense phase would intrinsically compromise a small fraction of the mass of a particle, it is likely that it would not be detected with the equipment that was available.

Returning to the two distinct unexpected environments observed in the ^29^Si {^1^H} spectra for OSPC-1b, the calculated ^29^Si NMR shift for the models BG–BQ and an additional high-volume model are plotted as a function of volume in Figure SI.48 compared to the experimental ^29^Si {^1^H} spectra. We can see that all shift values correlate to the experimental peak at ∼ −20 ppm. We can therefore assign this peak as originating from the Si of the terminating trimethylsilyl end groups (TMS end group) of the OSPC-1 polymer. We now hypothesize that the broad, multishouldered peak at ∼ 0 ppm is due to molecular tetramethylsilane (molecular TMS) based species. Molecular TMS is a potential side product from the synthetic route for OSPC-0b and OSPC-1b, via consecutive condensation reactions between the byproduct trimethylsilyl fluoride, resulting in molecular TMS and silicon tetrafluoride (SiF_4_). While we expect that most of these volatile species would be removed during evacuation/degassing, it is not unreasonable to expect that some species would be kinetically trapped within the microporous structure. Figure SI.49 and Figure SI.50 show the calculated NMR shifts for molecular TMS, SiF_4_, intermediate species, and various models of molecular TMS interacting with OSPC fragments. We can see several calculated shifts that occur within the bounds of the experimental set of peaks. The calculated shift from the TMS end group occurs at ∼ −19 ppm, in line with the value obtained from periodic models. The calculated shift for SiF_4_ occurs just above this at ∼ −19.3 ppm and so we infer that it also contributes to the experimental peak observed at ∼ −20 ppm. The calculated shift for molecular TMS interacting with a OSPC fragment or with a bromine terminated fragment is ∼ 3 ppm but we observed that the calculated shift is dependent upon the distance from the OSPC fragment. Given that OSPC has a range of pore sizes then we expect that this will result in a broad range of values, we therefore separate the molecular TMS into three categories; a shift centered at 0 ppm representing molecular TMS within pores but not directly interacting with OSPC, a shift centered at 3 ppm representing molecular TMS directly interacting with OSPC fragments including when bromine terminated, and a broad shift representing molecular TMS interacting with the packed OSPC polymer taking into account the diverse and amorphous nature of the pore structure. For molecular TMS interacting with a TMS end group we calculate a shift of ∼ −8 ppm for the Si atom in the molecular TMS, and ∼ −27 ppm for the Si atom from the TMS end group. Taking these assignments into account, a modeled spectrum is generated, shown in Figure SI.51, that shows a good match to the experimental spectra.

### 2.6 Structure Rationalization

OSPC-1a and OSPC-1b exhibit a mixture of meso and microporosity. We have assumed that OSPC-1a and OSPC-1b have the same bulk chemical structure and that the only differences are the end group chemistry. Given the similarity of the collected NMR, XPS, and Raman spectra, this is a reasonable assumption to make. However, we must note that the different synthetic route and end group chemistry may effect structural properties including connectivity, network interpenetration, densities, and pore sizes.

For the more porous OSPC-1b, a total surface area of 909 m^2^ g^–1^ was obtained, of which 617 m^2^ g^–1^ can be attributed to micropores, with the remaining 292 m^2^ g^–1^ being attributed to mesopores.^1^ The pore size distribution of OSPC-1b, given in Figure SI.52, shows a large proportion of pore sizes with diameters below 0.5 nm, followed by a set of small peaks at 0.6–0.8 nm, followed by a larger peak centered at 1.2 nm and a smaller peak centered at 1.7 nm. Porous polymers tend to produce a range of surface areas for different batches.^47^ To explore this for OSPC-0b and OSPC-1b we undertake repeat measurements for a number of batches, shown in Table SI. 1. For OSPC-1b, we find that high through-put BET analysis on 5-points of the isotherm gives an average surface area of 329 m^2^ g^–1^ with standard deviation of 62 m^2^ g^–1^. 5-point BET analysis severely underestimates the surface area but gives a good approximation of the expected spread in the samples tested – i.e. the underestimation is consistent between samples and so the spread determined is reflective of that that would be observed if a full isotherm had been collected. Further, surface areas determined from application of the BET equation have large errors associated of around 100 m^2^ g^–1^. Therefore, for OSPC-1b we expect total surface areas in the range of 700 – 1100 m^2^ g^–1^ with similar break down of roughly 66% being microporous (462–726 m^2^ g^–1^) and 33% being mesoporous (231–363 m^2^ g^–1^).

Previous studies have postulated that the structure of particles of the CMP family of materials (of which OSPC is a member) is complex due to the phase separation influences of the reaction mix.^47^ Spherical particles of CMPs were suggested to have a shell-like structure, with regions of higher and lower density. The order and ratios of these depended upon the specific monomer and solvent reaction mix. It is therefore reasonable to suggest that a similar scenario is occurring for OSPC-1, where there are dense regions of highly interpenetrated OSPC-1 framework material and small amounts of open low density high surface area OSPC-1 framework.

To assess and explore the plausibility of this proposal, we construct a sphere of OSPC-1 (with ethynyl end groups, Figure SI.54) using Ambuild (as set out in SI section 2) which is approximately 7 nm in diameter, approximating the structure of the highly porous shell. In this model, the OSPC-1 framework is highly open at the edge of the sphere forming large pores with a higher density at the core and so mimicking the edges of the particles. Measuring the diameter of these large open edge pores, we see that they are between 1 and 2 nm with most being at around 1.1 nm. The pore size distribution was determined, shown in Figure SI.55, with a particularly good match in the region of 0.6 to 1.1 nm. The small size of the spherical model in comparison to the much larger expected size of the particles, limits the extent to which we can model pore sizes that require larger scale models to become evident. Therefore, pores that have a diameter of greater than 1 nm are likely to be highly under-represented in the calculated PSD. Additionally, as we were unable to achieve the very high core densities proposed using this technique, it is reasonable that a good match is observed for the size and density ranges that this model mimics, rather than the whole of the experimental PSD.

To further probe the potential structural features that may be present within the amorphous framework over the range of densities proposed, the pore size distribution was determined for the crystalline OSPC-1 models in the *dia*, *unc-c*, and *pts-c* topologies (*dia-c* is nonporous and has pore diameters smaller than 0.5 nm), shown in Figure 7. We see that the experimentally determined pore size distribution has a peak at ∼ 0.6 nm, a smaller peak at ∼ 0.8 nm, a large broad peak centered at ∼ 1.2 nm, and a smaller broad centered peak at ∼ 1.7 nm. The pore size distribution for the *dia* model has one peak at ∼ 1.15 nm, the *unc-c* model has one peak at ∼ 0.77 nm, and the *pts-c* model has one peak at 0.59 nm. These match exceptionally well with the peaks observed experimentally and suggest that the amorphous framework contains similar structural features. We have highlighted these structural features in Figure SI. 23, 28, and 29. This supports the hypothesized shell model, where the core is a dense interpenetrated structure, surrounded by a shell of very highly porous noninterpenetrated OSPC-1 that approximates to a structure containing similar features found in the *dia*, *unc-c*, and *pts-c* topologies. We label this as an *open-amorphous-framework* topology.

**Figure 7:**
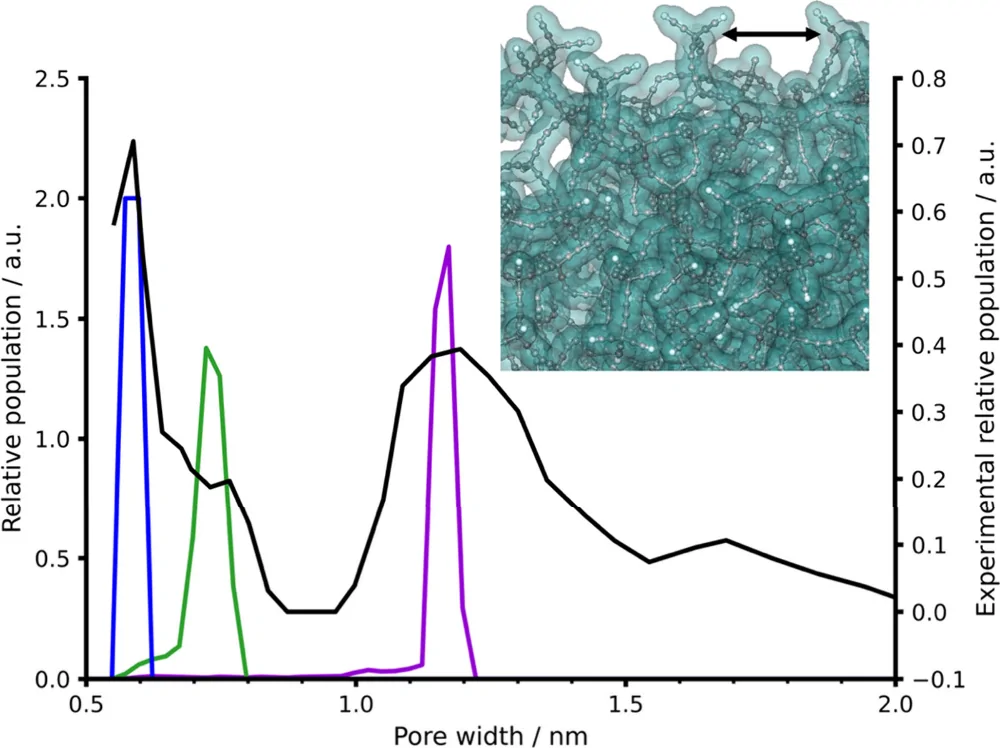
Experimental pore size distribution of OSPC-1b, black, showing the calculated pore size distribution for models of OSPC-1 with idealized crystalline topologies calculated using Poreblazer 4.0.^48^ Key: purple – *dia*, green – *unc-c*, blue – *pts-c*. Inset is a section from the spherical model showing a van der Waals surface (transparent green) that has a pore highlighted with diameter ∼ 1.2 nm.

High resolution TEM was collected for OSPC-1a, shown in Figure SI.55.^1^ The particles observed are not inconsistent with this hypothesis and show similar surface topology to that expected. To further investigate this structural model, high resolution SEM was performed on OSPC-1b powder (Figure 8 and Figure SI.56). Rather than showing a core–shell structure, these show the presence of “fuzzy” regions on the surface of particles. Higher magnification of these regions reveals a diffuse structure. Applying backscattering to the image confirms that the shell regions are a different morphology to the core particles. The significant contrast observed between the two regions could imply a mixture of two materials.^19^ However, EDS analysis of five sites across a single particle, Table 1, reveals no significant difference in elemental composition. This confirms that the dense and fuzzy regions are two very different morphologies of the same material. Lower magnification SEM images were also captured with and without backscattering (SI.58), which confirmed that the fuzzy morphology was present throughout the OSPC-1b sample on particles of all sizes. Although modeled previously as perfectly spherical core and shell, for an amorphous material with irregular particles it is entirely within expectations for the shell to be unevenly distributed around the surface of the particles. Further, systems with particles of different morphologies are common, especially where there are crystalline and amorphous phases.^49,50^ We therefore assign the particles to a dense structure approximated by structural model MF, and the “fuzzy” regions to the *open-amorphous-framework* topology.

**Figure 8:**
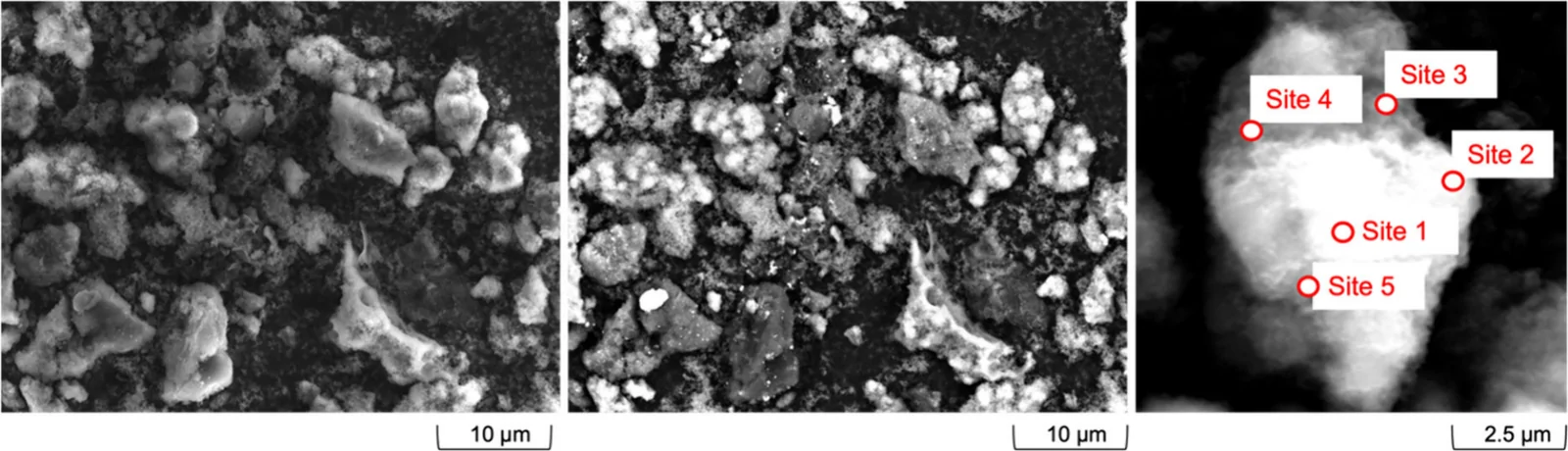
SEM imaging of OSPC-1b taken without (left) and with backscattering (middle). The highlighted particle (right) was investigated through EDS, results for each site shown in Table 1.

**Table 1:** EDS for Each Site Identified in Figure 8

Wt%	Site 1	Site 2	Site 3	Site 4	Site 5
C	86.4	85.5	85.0	82.3	77.9
O	6.2	7.8	8.4	5.7	5.5
Br	5.4	5.0	4.8	9.3	12.8
Si	1.2	1.2	1.2	2.0	2.9
Na	0.4	0.3	0.3	0.3	0.2

Assessing the regions shown in Figure 8 and Figures SI.56 and 57, we can cautiously estimate the relative volumes of each morphology and generate an estimated surface area. Estimating volumes of morphology from an SEM image is challenging as the distinction between morphologies is not clear and a 2D image can result in underestimation of either morphology. These caveats aside, we estimate that the percentage of the volume ranges between 45 and 90% for the dense morphology and 10–55% for the fuzzy morphology depending upon the particle, with an estimated overall ratio of 70:30 respectively. From Figure 7, we can see that all pore sizes originating from our models are within the microprous range, being less than 2 nm in width and thus the comparable experimentally determined surface area will be 462 – 726 m^2^ g^–1^. As a rough sanity check, using our densely packed chain models and our *unc-c* and *dia* models to estimate the relationship between density, pore width and surface area, and the relative ratios of porous models based upon the pore size distribution (see SI section 12), we can estimate that when the fuzzy morphology is 10% of the volume, the surface area from the modeled microporous regions is approximately 160 m^2^ g^–1^, and at 55% of the volume the surface area from the modeled microporous regions is approximately 900 m^2^ g^–1^. At the estimated 30% overall fuzzy volume, the surface area is approximately 500 m^2^ g^–1^, which combined with micropore volume that we are not able to model (such as interparticulate volume), puts us comfortably within the experimentally determined micropore surface area range of 462 – 726 m^2^ g^–1^. However, we note again that this is a rough estimate only, with further work being required to confirm the relative ratio of dense to fuzzy regions and the respective surface area of each phase.

## 3 Conclusions

We have shown that the solid-state NMR spectra of both OSPC-1a and OSPC-1b can only be rationalized by a dense highly interpenetrated structure where the ethynyl chains can interact. We suggest that this structure is dominant, but that porosity arises from small regions of open noninterpenetrated highly porous OSPC-1 framework structure best approximated by the topological features present in the *dia*, *pts-c*, and *unc-c* topologies. This has important implications for the properties of OSPC materials, including the conductivity, gas uptake, and ion storage, where the relative ratios of these two different structural forms could greatly alter their respective capabilities. It is imperative that the bimodal structural nature is taken into account when considering new material targets or synthetic adaptions to direct to either structural form. These understandings will therefore greatly aid in the design of new OSPC materials with targeted properties for potential applications including as electrode materials, electrochemical activity, and photocatalysis.

## 4 Experimental Section/Methods

All materials were synthesized according to the one-pot method. This method and more information are outlined in SI Section 1.

### 4.1 NMR

Solid state NMR was performed on a Bruker AVANCE III HD 400 Wb using a 2.5 mm probe. The results were obtained through the Hahn echo direct excitation of ^13^C. The magic angle spinning (MAS) frequency was 25 kHz and the D1 was 60 s.

Additional solid-state NMR was performed on a Bruker AVANCE III HD 800 Wb using a 3.2 mm probe. The results were obtained through the Hahn echo direct excitation of ^13^C. The MAS frequency was 16 kHz and the D1 was 300 s.

^1^H–^13^C and ^1^H–^29^Si cross-polarization magic-angle spinning (CP/MAS) experiments were performed on a Bruker Avance III spectrometer, equipped with a double resonance 4 mm HX probe, operating at a ^1^H frequency of 300.11 MHz. ^29^Si­{^1^H} spectra were acquired using ^1^H π/2 *rf*. pulse of 4.5 μs, contact time of 4 ms, recycle delay of 10 s, and a minimum of 6k scans, at a MAS rate of 10 kHz. ^13^C­{^1^H} spectra were acquired using ^1^H π/2 *rf*. pulse of 4.5 μs, contact time of 1 ms, recycle delay of 5 s, and a minimum of 8k scans, at a MAS rate of 10 kHz. ^29^Si and ^13^C spectra were externally referenced to TMS. These are shown in Figure SI.5–7.

### 4.2 EPR

EPR was performed on a Bruker EMXplus spectrometer operating at 9.815 GHz at 2.133 mW, receiver gain 2.00 × 10^4^, time constant 0.64 ms, sweep width 100 G, conversion time 8 ms, modulation amplitude 1G, and 32 scans.

### 4.3 SEM

SEM was performed on a Field-free UHR-SEM BrightBeam of the TESCAN AMBER model. Electron backscatter diffraction was performed with an Oxford Instruments Symmetry S3. EDS was performed on an Oxford Instruments Ultim Max 40 using a 40 mm^2^ large area EDS detector.

### 4.4 Computational *Fragment* Models

All fragment calculations were performed using Gaussian 09. For each fragment model, the (B3LYP/6–311G) model chemistry was used for a geometry optimization followed by an NMR shielding calculation utilizing the continuous set of gauge transformations (CSGT).^51–56^

### 4.5 Computational *Periodic* Models

All models had their geometry optimized using the CASTEP plane wave DFT code, with the PBE exchange-correlation functional, a Monkhorst–Pack k-point grid of 1 × 1 × 1 and a cutoff energy of 50 Ry.^57–64^ The periodic carbon NMR spectra were obtained with the same parameters.^57,59–63,65–68^

## Supplementary Material



## Data Availability

Computational files are available for download from github.com/catherinemollart/OSPC_NMR. These include the output and structure files for convergence test results, crystalline models, and model sets 1–13

## Notes

Synthetic Strategies; computational method; TES validation of method; structural fragment models and their NMR spectra; structural periodic models and their NMR spectra; OSPC-1 chain-chain structural models; OSPC-1 chain-chain structural models with different end groups; energy mapping; NMR spectra; peak assignments; NMR relaxation times; and pore size distribution, TEM, SEM, and approximated surface areas (PDF)

## References

[1] Zhao Z., Das S., Xing G., Fayon P., Heasman P., Jay M., Bailey S., Lambert C., Yamada H., Wakihara T. (2018). A 3D Organically
Synthesized Porous Carbon Material for Lithium-Ion
Batteries. Angew. Chem., Int. Ed..

[2] Rowling A., Doulcet J., Dawson R., Tapia-Ruiz N., Trewin A. (2024). Facile Synthesis of Organically Synthesized Porous
Carbon Using a Commercially Available Route with Exceptional Electrochemical
Performance. ACS Appl. Mater. Interfaces.

[3] Wood C. D., Tan B., Trewin A., Niu H., Bradshaw D., Rosseinsky M. J., Khimyak Y. Z., Campbell N. L., Kirk R., Stoeckel E. (2007). Hydrogen Storage in Microporous Hypercrosslinked Organic Polymer
Networks. Chem. Mater..

[4] Weber J., Thomas A. (2008). Toward Stable Interfaces
in Conjugated Polymers: Microporous
Poly­(p-phenylene) and Poly­(phenyleneethynylene) Based on a Spirobifluorene
Building Block. J. Am. Chem. Soc..

[5] Jiang J.-X., Su F., Trewin A., Wood C. D., Campbell N. L., Niu H., Dickinson C., Ganin A. Y., Rosseinsky M. J., Khimyak Y. Z. (2007). Conjugated
Microporous Poly­(aryleneethynylene)
Networks. Angew. Chem., Int. Ed..

[6] McKeown N. B., Budd P. M. (2006). Polymers of intrinsic microporosity (PIMs): organic
materials for membrane separations, heterogenous catalysis and hydrogen
storage. Chem. Soc. Rev..

[7] Kuhn P., Antonietti M., Thomas A. (2008). Porous, Covalent Triazine-Based Frameworks
Prepared by Ionothermal Synthesis. Angew. Chem.,
Int. Ed..

[8] Liu A., Mollart C., Trewin A., Fan X., Lau C. H. (2023). Photo-Modulating
CO2 Uptake of Hypercross-linked Polymers Upcycled from Polystyrene
Waste. ChemSusChem.

[9] Wang C., Yan T., Xing G., Bailey S., Lambert C., Fayon P., Trewin A., Ben T. (2022). Electron and proton conducting framework
organic salt single crystals. J. Solid State
Chem..

[10] Fayon P., Thomas J. M. H., Trewin A. (2016). Structure
and Properties of a Nanoporous
Supercapacitor. J. Phys. Chem. C.

[11] Kou Y., Xu Y., Guo Z., Jiang D. (2011). Supercapacitive Energy Storage and
Electric Power Supply Using an Aza-Fused π-Conjugated Microporous
Framework. Angew. Chem. - Int. Ed..

[12] Breher F., Grunenberg J., Lawrence S. C., Mountford P., Rüegger H. (2004). A Monomeric Organolithium Compound Containing a Free
Pyramidal Carbanion in Solution and in the Solid State. Angew. Chem. - Int. Ed..

[13] Brownbill N. J., Sprick R. S., Bonillo B., Pawsey S., Aussenac F., Fielding A. J., Cooper A. I., Blanc F. (2018). Structural Elucidation
of Amorphous Photocatalytic Polymers from Dynamic Nuclear Polarization
Enhanced Solid State NMR. Macromolecules.

[14] Cataldo F., Capitani D. (1999). Preparation and characterization of carbonaceous matter
rich in diamond-like carbon and carbyne moieties. Mater. Chem. Phys..

[15] Chisholm M. H., Ho D., Huffman J. C., Marchant N. S. (1989). Reactions
between certain trialkoxytungsten
alkylidyne complexes and carbon monoxide. Competitive carbon-carbon
bond formation leading to alkyne or ketenyl complexes. Organometallics.

[16] Cooper T. M., Krein D. M., Burke A. R., McLean D. G., Rogers J. E., Slagle J. E., Fleitz P. A. (2006). Spectroscopic
Characterization of
a Series of Platinum Acetylide Complexes Having a Localized Triplet
Exciton. J. Phys. Chem. A.

[17] Dubois M., Guérin K., Petit E., Batisse N., Hamwi A., Komatsu N., Giraudet J., Pirotte P., Masin F. (2009). Solid-State
NMR Study of Nanodiamonds Produced by the Detonation Technique. J. Phys. Chem. C.

[18] Fukui K., Sato K., Shiomi D., Takui T., Itoh K., Gotoh K., Kubo T., Yamamoto K., Nakasuji K., Naito A. (1999). Electronic structure
of a stable phenalenyl radical in crystalline
state as studied by SQUID measurements, cw-ESR, and 13C CP/MAS NMR
spectroscopy. Synth. Met..

[19] George L., Netsch K.-P., Penn G., Kollenz G., Wentrup C. (2006). Oxoketene–oxoketene,
imidoylketene–imidoylketene and oxoketenimine–imidoylketene
rearrangements. 1,3-Shifts of phenyl groups. Org. Biomol. Chem..

[20] Gu S., Wu S., Cao L., Li M., Qin N., Zhu J., Wang Z., Li Y., Li Z., Chen J. (2019). Tunable Redox Chemistry and Stability of Radical Intermediates in
2D Covalent Organic Frameworks for High Performance Sodium Ion Batteries. J. Am. Chem. Soc..

[21] Harris K. J., Bernard G. M., McDonald C., McDonald R., Ferguson M. J., Wasylishen R. E. (2006). Acetylenic
Carbon-13 Chemical Shift Tensors for Diphenylacetylene
and (η2-Diphenylacetylene)­Pt­(PPh3)­2: A Solid-State NMR and Theoretical
Study. Inorg. Chem..

[22] He H., Riedl T., Lerf A., Klinowski J. (1996). Solid-State
NMR Studies of the Structure of Graphite Oxide. J. Phys. Chem..

[23] Huang T.-K., Chi Y., Peng S.-M., Lee G.-H., Wang S.-L., Liao F.-L. (1995). Synthesis
and Characterization of a (Ketenyl)metal Cluster Complex, an Intermediate
in the Oxidative Decarbonation of an Acetylide Ligand. Organometallics.

[24] Jiang J.-X., Su F., Trewin A., Wood C. D., Niu H., Jones J. T. A., Khimyak Y. Z., Cooper A. I. (2008). Synthetic Control of the Pore Dimension
and Surface Area in Conjugated Microporous Polymer and Copolymer Networks. J. Am. Chem. Soc..

[25] Karakhanov E., Maximov A., Terenina M., Vinokurov V., Kulikov L., Makeeva D., Glotov A. (2020). Selective
hydrogenation
of terminal alkynes over palladium nanoparticles within the pores
of amino-modified porous aromatic frameworks. Catal. Today.

[26] Panich A. M. (2007). Solid state
nuclear magnetic resonance studies of nanocarbons. Diam. Relat. Mater..

[27] Ren S., Dawson R., Laybourn A., Jiang J.-X., Khimyak Y., Adams D. J., Cooper A. I. (2012). Functional
conjugated microporous
polymers: from 1,3,5-benzene to 1,3,5-triazine. Polym. Chem..

[28] Sabbatini, L. , De Giglio, E. Polymer Surface Characterization; De Gruyter, 2022 10.1515/9783110701098.

[29] Taki Y., Takai O. (1998). XPS structural characterization of hydrogenated amorphous carbon
thin films prepared by shielded arc ion plating. Thin Solid Films.

[30] Tao F., Qiao M. H., Li Z. H., Yang L., Dai Y. J., Huang H. G., Xu G. Q. (2003). Adsorption of phenylacetylene on
Si(100)-21: Reaction mechanism and formation of a styrene-like \ensuremath
pi-conjugation system. Phys. Rev. B.

[31] Bowman M. C., Burke A. D., Turney J. M., Schaefer H. F. (2018). Mechanisms of the
Ethynyl Radical Reaction with Molecular Oxygen. J. Phys. Chem. A.

[32] Dong C., Chu J., Cao L., Cui F., Liang S., Zhang X., Tao X., Wang H.-G., Zhu G. (2023). A Three-Dimensional Silicon-Diacetylene
Porous Organic Radical Polymer. CCS Chem..

[33] Elias J., Faccinetto A., Vezin H., Mercier X. (2023). Investigation of resonance-stabilized
radicals associated with soot particle inception using advanced electron
paramagnetic resonance techniques. Commun. Chem..

[34] Green U., Shenberger Y., Aizenshtat Z., Cohen H., Ruthstein S. (2014). Exploring
the Radical Nature of a Carbon Surface by Electron Paramagnetic Resonance
and a Calibrated Gas Flow. J. Vis. Exp..

[35] Tian L., Koshland C. P., Yano J., Yachandra V. K., Yu I. T. S., Lee S. C., Lucas D. (2009). Carbon-Centered
Free
Radicals in Particulate Matter Emissions from Wood and Coal Combustion. Energy Fuels.

[36] Vitiello G., De Falco G., Picca F., Commodo M., D’Errico G., Minutolo P., D’Anna A. (2019). Role of radicals
in carbon clustering
and soot inception: A combined EPR and Raman spectroscopic study. Combust. Flame.

[37] Caliskan B., Caliskan A. C. (2021). Electron paramagnetic
resonance study of the paramagnetic
centers in gamma-irradiated oxalic acid dihydrate single crystal. Radiat. Phys. Chem..

[38] Closs G. L., Forbes M. D. E., Piotrowiak P. (1992). Spin and reaction
dynamics in flexible
polymethylene biradicals as studied by EPR, NMR, optical spectroscopy,
and magnetic field effects. Measurements and mechanisms of scalar
electron spin-spin coupling. J. Am. Chem. Soc..

[39] Liu Y., Villamena F. A., Rockenbauer A., Song Y., Zweier J. L. (2013). Structural
Factors Controlling the Spin–Spin Exchange Coupling: EPR Spectroscopic
Studies of Highly Asymmetric Trityl–Nitroxide Biradicals. J. Am. Chem. Soc..

[40] Thomas J. M. H., Trewin A. (2014). Amorphous PAF-1: Guiding
the Rational Design of Ultraporous
Materials. J. Phys. Chem. C.

[41] Fayon P., Trewin A. (2016). Formation mechanism
of ultra porous framework materials. Phys. Chem.
Chem. Phys..

[42] Ma Z., Halling M. D., Solum M. S., Harper J. K., Orendt A. M., Facelli J. C., Pugmire R. J., Grant D. M., Amick A. W., Scott L. T. (2007). Ring Current Effects in Crystals. Evidence from 13C
Chemical Shift Tensors for Intermolecular Shielding in 4,7-Di-t-butylacenaphthene
versus 4,7-Di-t-butylacenaphthylene. J. Phys.
Chem. A.

[43] Mejia J. E., Butler-Au H. E., Thompson N. E., Goldman K. D., Zengel E. R., Pike R. D., Mao J., Bayse C. A. (2025). Identification of
π-Stacking Motifs in Naphthalene Diimides via Solid-State NMR. J. Am. Chem. Soc..

[44] Harris K. J., Bernard G. M., McDonald C., McDonald R., Ferguson M. J., Wasylishen R. E. (2006). Acetylenic
Carbon-13 Chemical Shift Tensors for Diphenylacetylene
and (η2-Diphenylacetylene)­Pt­(PPh3)­2: A Solid-State NMR and Theoretical
Study. Inorg. Chem..

[45] Botto R. E., Wilson R., Winans R. E. (1987). Evaluation
of the reliability of
solid 13C-NMR spectroscopy for the quantitative analysis of coals:
study of whole coals and maceral concentrates. Energy Fuels.

[46] La
Mar G. N. (1971). Problem with carbon-13 intensities in proton-decoupled nuclear magnetic
resonance spectra. Undermining the Overhauser effect with free radicals. J. Am. Chem. Soc..

[47] Mollart C., Trewin A. (2024). Conjugated microporous
polymer frameworks for sustainable
energy materials – elucidating the influence of solvents on
the porosity properties for future design principles. Journal of Materials Chemistry A.

[48] Sarkisov L., Bueno-Perez R., Sutharson M., Fairen-Jimenez D. (2020). Materials
Informatics with PoreBlazer v4.0 and the CSD MOF Database. Chem. Mater..

[49] Karpowich L., Wilcke S., Yu R., Harley G., Reimer J. A., De Jonghe L. C. (2007). Synthesis
and characterization of mixed-morphology
CePO4 nanoparticles. J. Solid State Chem..

[50] Lan H., Wang J., Cheng L., Yu D., Wang H., Guo L. (2024). The synthesis
and application of crystalline–amorphous hybrid
materials. Chem. Soc. Rev..

[51] Becke A. D. (1993). Density-functional
thermochemistry. III. The role of exact exchange. J. Chem. Phys..

[52] Frisch, M. J. ; Trucks, G. W. ; Schlegel, H. B. ; Scuseria, G. E. ; Robb, M. A. ; Cheeseman, J. R. ; Scalmani, G. ; Barone, V. ; Petersson, G. A. ; Nakatsuji, H. , Gaussian 09; Gaussian, Inc., 2009.

[53] Lee C., Yang W., Parr R. G. (1988). Development
of the Colle-Salvetti
correlation-energy formula into a functional of the electron density. Phys. Rev. B.

[54] Stephens P. J., Devlin F. J., Chabalowski C. F., Frisch M. J. (1994). Ab Initio Calculation
of Vibrational Absorption and Circular Dichroism Spectra Using Density
Functional Force Fields. J. Phys. Chem..

[55] Vosko S. H., Wilk L., Nusair M. (1980). Can. J. Phys..

[56] Krishnan R., Binkley J. S., Seeger R., Pople J. A. (1980). Self-consistent
molecular orbital methods. XX. A basis set for correlated wave functions. J. Chem. Phys..

[57] Clark S. J., Segall M. D., Pickard C. J., Hasnip P. J., Probert M. I. J., Refson K., Payne M. C. (2005). First principles
methods using CASTEP. Z. für Kristallogr.
- Cryst. Mater..

[58] Francis G. P., Payne M. C. (1990). Finite basis set
corrections to total energy pseudopotential
calculations. J. Phys.: Condens. Matter.

[59] Hohenberg P., Kohn W. (1964). Inhomogeneous Electron Gas. Phys. Rev..

[60] Kohn W., Sham L. J. (1965). Self-Consistent Equations Including Exchange and Correlation
Effects. Phys. Rev..

[61] Monkhorst H. J., Pack J. D. (1976). Special points for
Brillouin-zone integrations. Phys. Rev. B.

[62] Payne M. C., Teter M. P., Allan D. C., Arias T. A., Joannopoulos J. D. (1992). Iterative
minimization techniques for ab initio total-energy calculations: molecular
dynamics and conjugate gradients. Rev. Mod.
Phys..

[63] Perdew J. P., Burke K., Ernzerhof M. (1996). Generalized
Gradient Approximation
Made Simple. Phys. Rev. Lett..

[64] Pfrommer B. G., Côté M., Louie S. G., Cohen M. L. (1997). Relaxation of Crystals
with the Quasi-Newton Method. J. Comput. Phys..

[65] Bonhomme C., Gervais C., Babonneau F., Coelho C., Pourpoint F., Azaïs T., Ashbrook S. E., Griffin J. M., Yates J. R., Mauri F. (2012). First-Principles Calculation of NMR Parameters Using
the Gauge Including Projector Augmented Wave Method: A Chemist’s
Point of View. Chem. Rev..

[66] Pickard C. J., Mauri F. (2001). All-electron magnetic
response with pseudopotentials: NMR chemical
shifts. Phys. Rev. B.

[67] Profeta M., Mauri F., Pickard C. J. (2003). Accurate
First Principles Prediction
of 17O NMR Parameters in SiO2: Assignment of the Zeolite Ferrierite
Spectrum. J. Am. Chem. Soc..

[68] Yates J. R., Pickard C. J., Mauri F. (2007). Calculation
of NMR chemical shifts
for extended systems using ultrasoft pseudopotentials. Phys. Rev. B.

