# Supplementary material for: Hidden Morphological Features in Organically Synthesized Porous Carbon Revealed through Analysis of Contradictory NMR Spectra
Source: ACS Omega. 2026 Sep 18;11(38):56979–92. doi: 10.1021/acsomega.6c03870 (PMC13628613; doi:10.1021/acsomega.6c03870)
Supplement: Supplementary file 1 [file ao6c03870_si_001.pdf]

Hidden Morphological Features in Organically Synthesized Porous Carbon Revealed Through Analysis of Contradictory NMR Spectra

Catherine Mollart,[A] Adam Rowling,[A] Ellena Sherrett,[A] Michael J G Peach,[A] Todor T. Koev,[B] Yaroslav Z. Khimyak,[B] and Abbie Trewin[A*]

A. Department of Chemistry, Lancaster University, UK, LA1 4YB. * E-mail: a.trewin@lancaster.ac.uk

B. School of Chemistry, Pharmacy and Pharmacology, University of East Anglia, Norwich Research Park, Norwich, UK, NR4 7TJ

Supporting Information

1. Synthetic Strategies

2. Computational

3. TES validation of method

4. Structural Fragment Models and their NMR spectra

5. Structural Periodic Models and their NMR spectra

6. OSPC-1 Chain-Chain Structural Models

7. OSPC-1 Chain-Chain Structural Models with different end groups

8. Energy Mapping

9. NMR Spectra

10. Peak assignments

11. NMR relaxation times

12. Pore size distribution, TEM, SEM, and approximated surface areas

1. Synthetic Strategies and characterisation

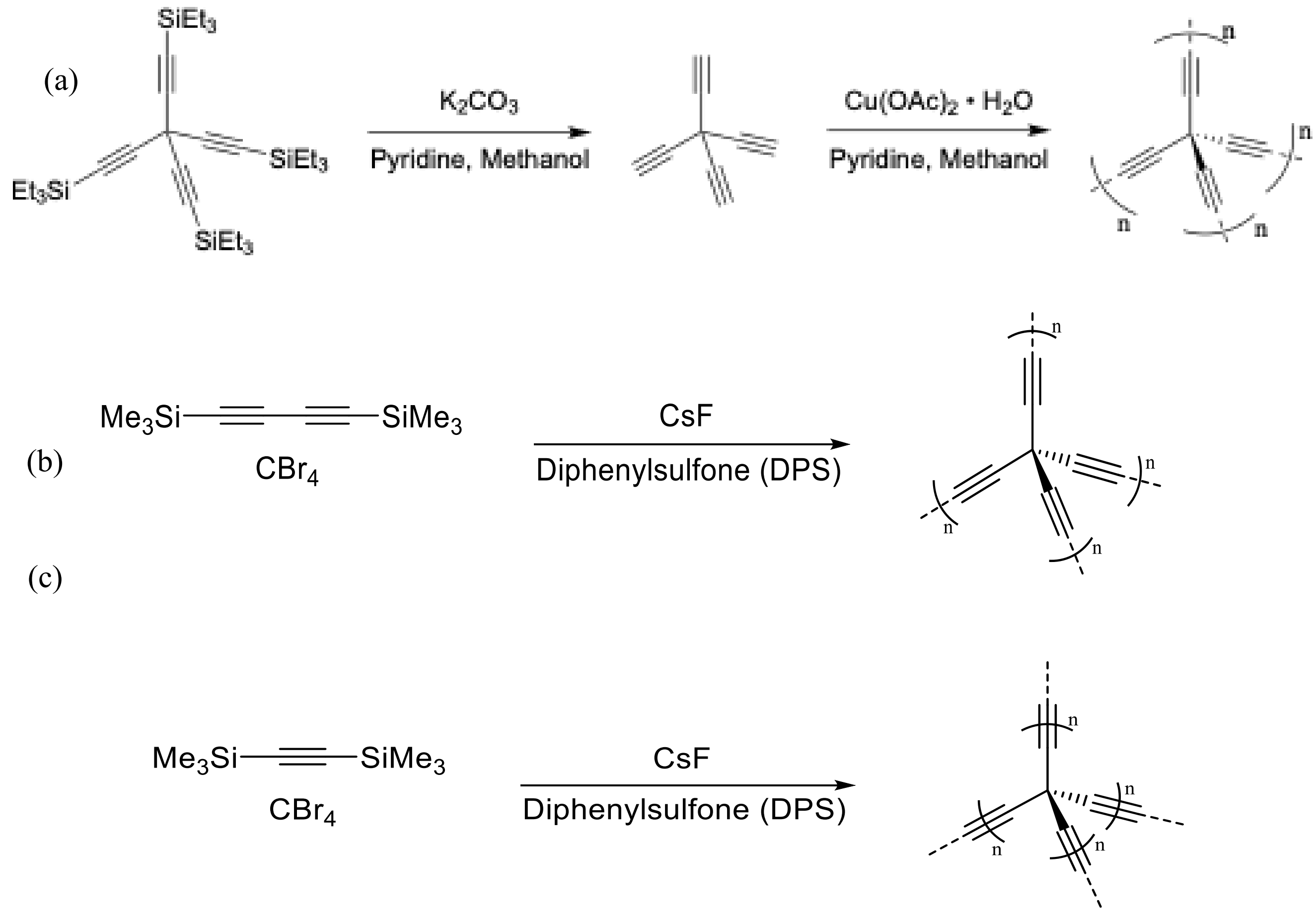

Figure SI.1: (a) Synthesis of the OSPC-1a framework *via* Eglinton homocoupling of tetraethynylmethane[1] **(b)** Synthesis of OSPC-1b *via* a one-pot strategy.[2] **(c)** Synthesis of OSPC-0b *via* a one pot strategy.[2]

Materials
Bistrimethylsilyl acetylene (99%), cesium fluoride (99%), and diphenyl sulfone (99%) were obtained from Alfa Aesar. Bistrimethylsilyl butadiyne (98%) was obtained from both Alfa Aesar and Manchester Organics. Carbon tetrabromide (99%) and dichlorobenzene (99%) were both obtained from Sigma-Aldrich. N-methylpyrolodinone was obtained from Fisher Scientific. All chemicals were used as purchased unless otherwise stated. Microwave vials were obtained from Biotage. Commercial graphite electrodes were obtained from MTI. The stainless-steel reactor and insert were obtained from BAOSHISHAN UK.

One pot synthesis of OSPC-1b: Bis(trimethylsilyl)diacetylene (0.40 g, 2 mmol), carbon tetrabromide (0.32 g, 1 mmol), CsF (0.75 g, 5 mmol) and diphenylsulfone (5 g) were added to a 20-mL microwave vial. The microwave vial was capped, and the mixture was heated at 250 °C overnight. Then, the rection mixture was cooled to 150 °C and dichlorobenzene (5 mL) was added. The black precipitate was filtered out and was suspended in NaOH aq. solution (50 mL, 2M) overnight. The black solid was then collected by filtration and rinsed with H_2O (25 mL). The resultant solid underwent further purification using Soxhlet extraction with methanol overnight and the product was then dried under high vacuum overnight to give OSPC-1b as a black powder (100 - 150 mg).

One pot synthesis of OSPC-0b: Bis(trimethylsilyl)acetylene (0.34 g, 2 mmol), carbon tetrabromide (0.33 g, 1 mmol) and CsF (0.75 g, 5 mmol) and diphenyl sulfone (2.5 g) were added to a 20-mL microwave vial. The microwave vial was capped, and

the mixture was heated at 250 °C overnight. Then, the rection mixture was cooled to 150 °C and dichlorobenzene (5 mL) was added. The black precipitate was filtered out and was suspended in NaOH aq. solution (50 mL, 2M) overnight. The black solid was then collected by filtration and rinsed with H_2O (25 mL). The resultant solid underwent further purification using Soxhlet extraction with methanol overnight and the product was then dried under high vacuum overnight to give OSPC-0b as a black powder (50 - 100 mg).

(a)

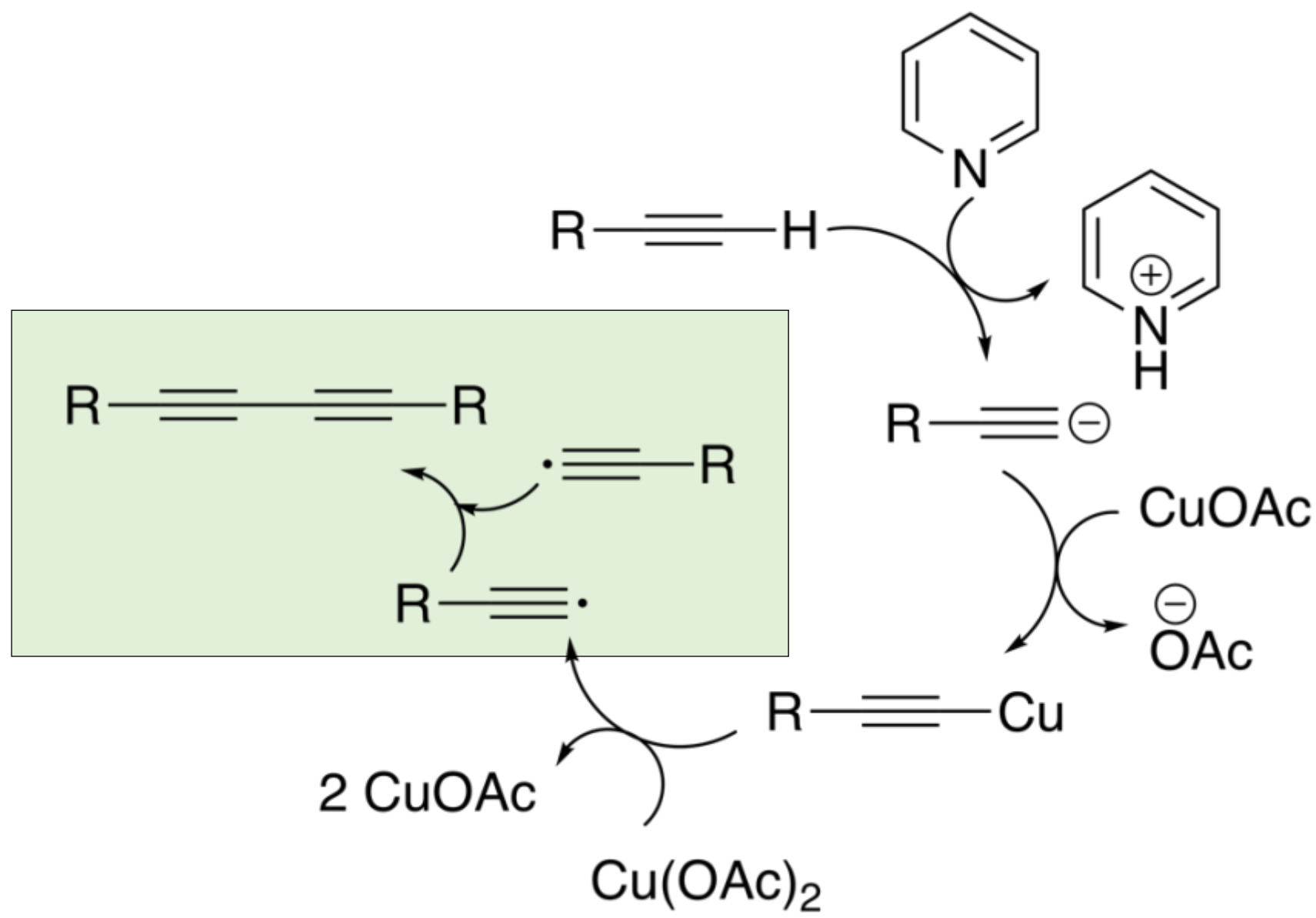

(b)

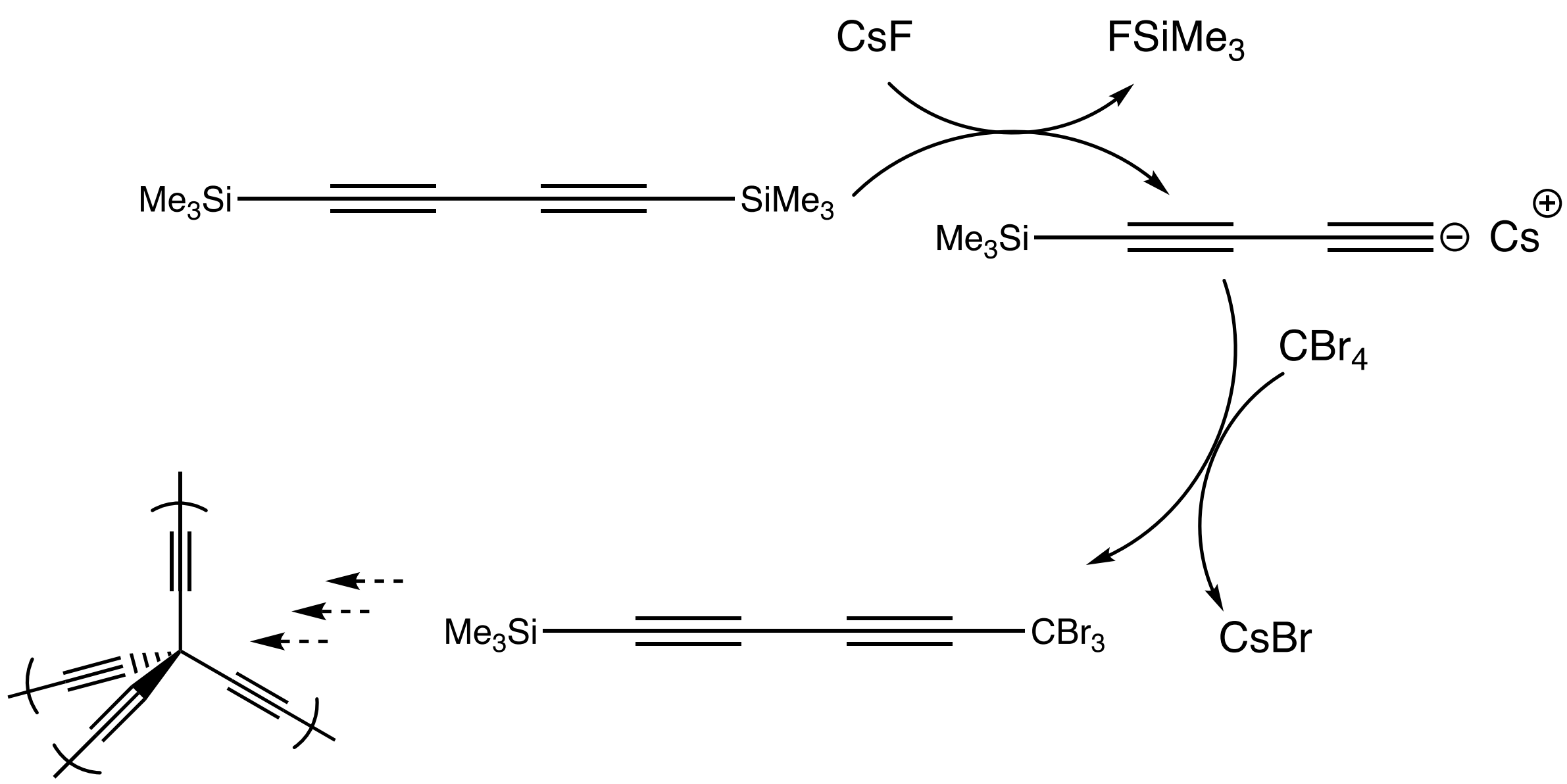

Figure SI.2: (a) Mechanism of the Eglinton homocoupling used in the synthesis of OSPC-1a. The key network forming step where two radicals combine is highlighted by the green box.[1] **(b)** The one pot synthetic strategy for synthesis of OSPC-1b *via* a continuous nucleophilic condensation reaction.[2] OSPC-0b is synthesised *via* the same mechanism.

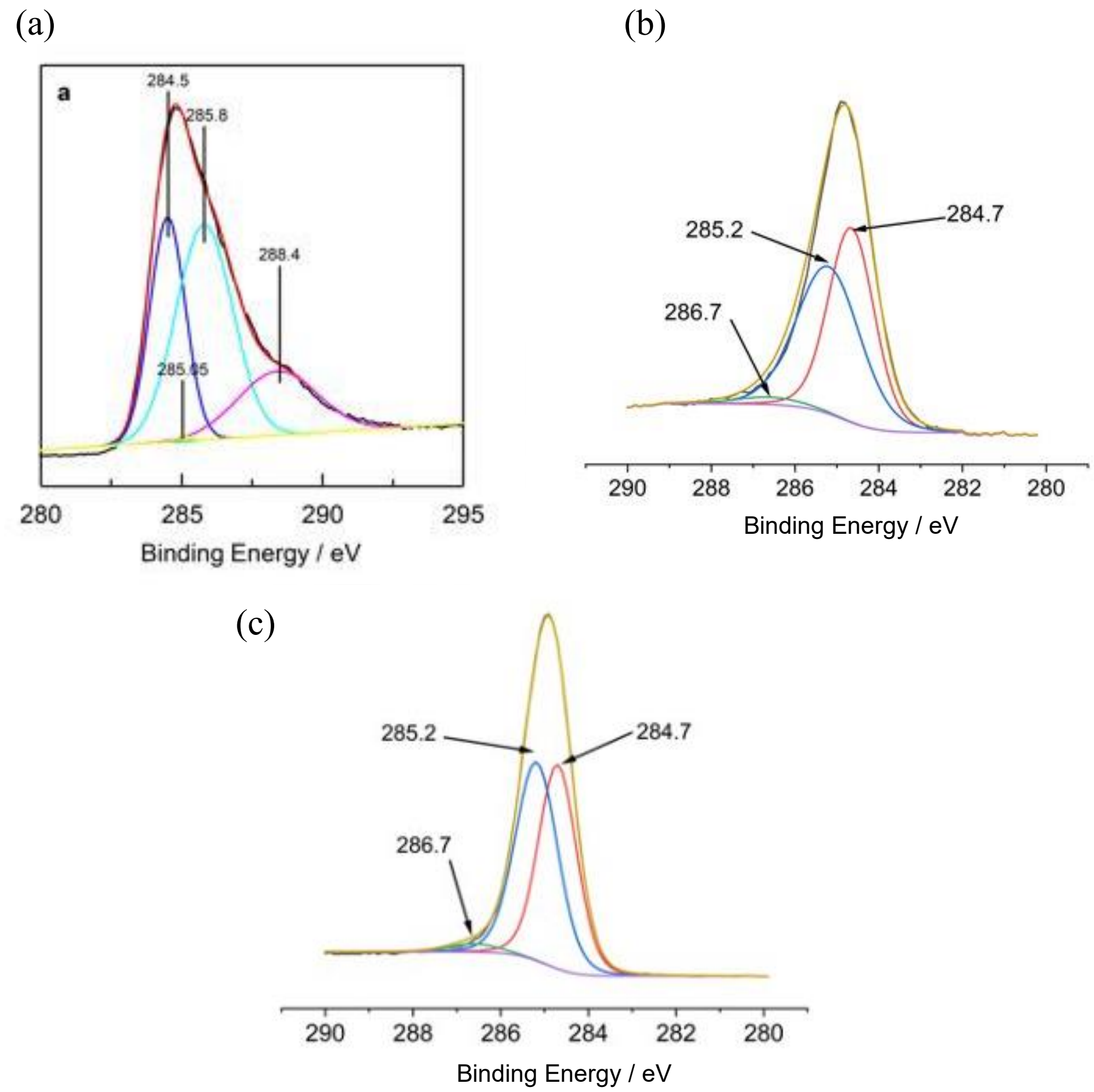

1, 2

Figure SI.3: (a) Fitted C1s XPS spectrum of OSPC-1a.[1] **(b)** Fitted C1s XPS spectrum of OSPC-1b. **(c)** Fitted C1s XPS spectrum of OSPC-0b.

Table SI.1: Surface areas of OSPC-0b and OSPC-1b determined through application of BET equation. Batch 1 and 2 were obtained using a full isotherm with 100 points whereas Batch 3-6 were obtained using 5 points. This is a high through put methodology that commonly gives a much lower surface area than that obtained through full analysis. It does give a good estimation of the range of surface areas that are obtained across those batches.

	Full BET analysis		5-point BET					
	Batch 1	Batch 2	Batch 3	Batch 4	Batch 5	Batch 6	Average	Standard deviation
OSPC-0b	473	261	77	60			69	12
OSPC-1b	726	909	259	344	306	405	329	62

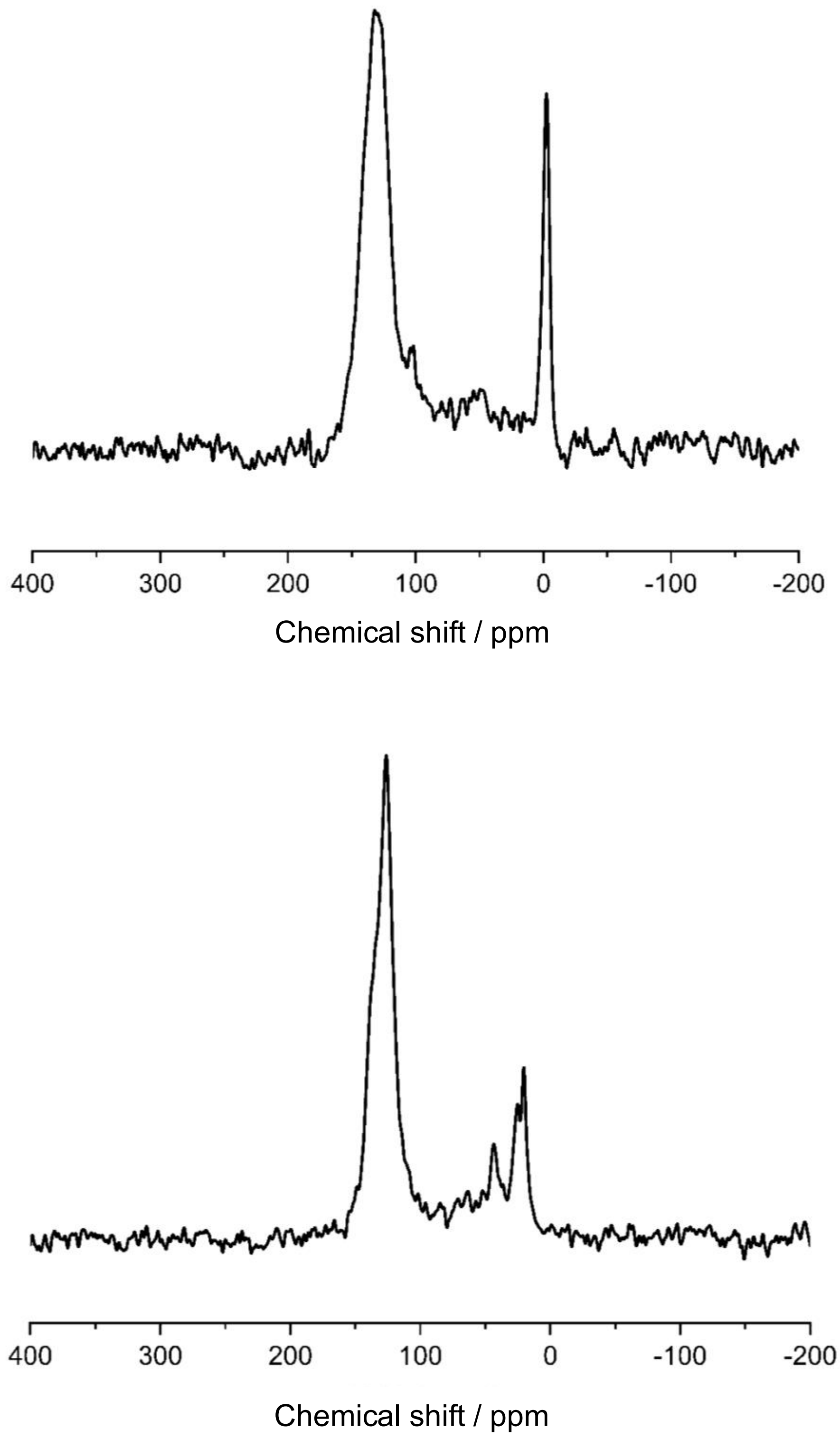

[1, 2]**Figure SI.4:** Solid state NMR spectra of OSPC-1b (top) and OSPC-0b (bottom) obtained at a high MAS speed of 25 kHz. To increase the spinning speed a smaller mass of sample had to be used with a lower resolution spectrometer.

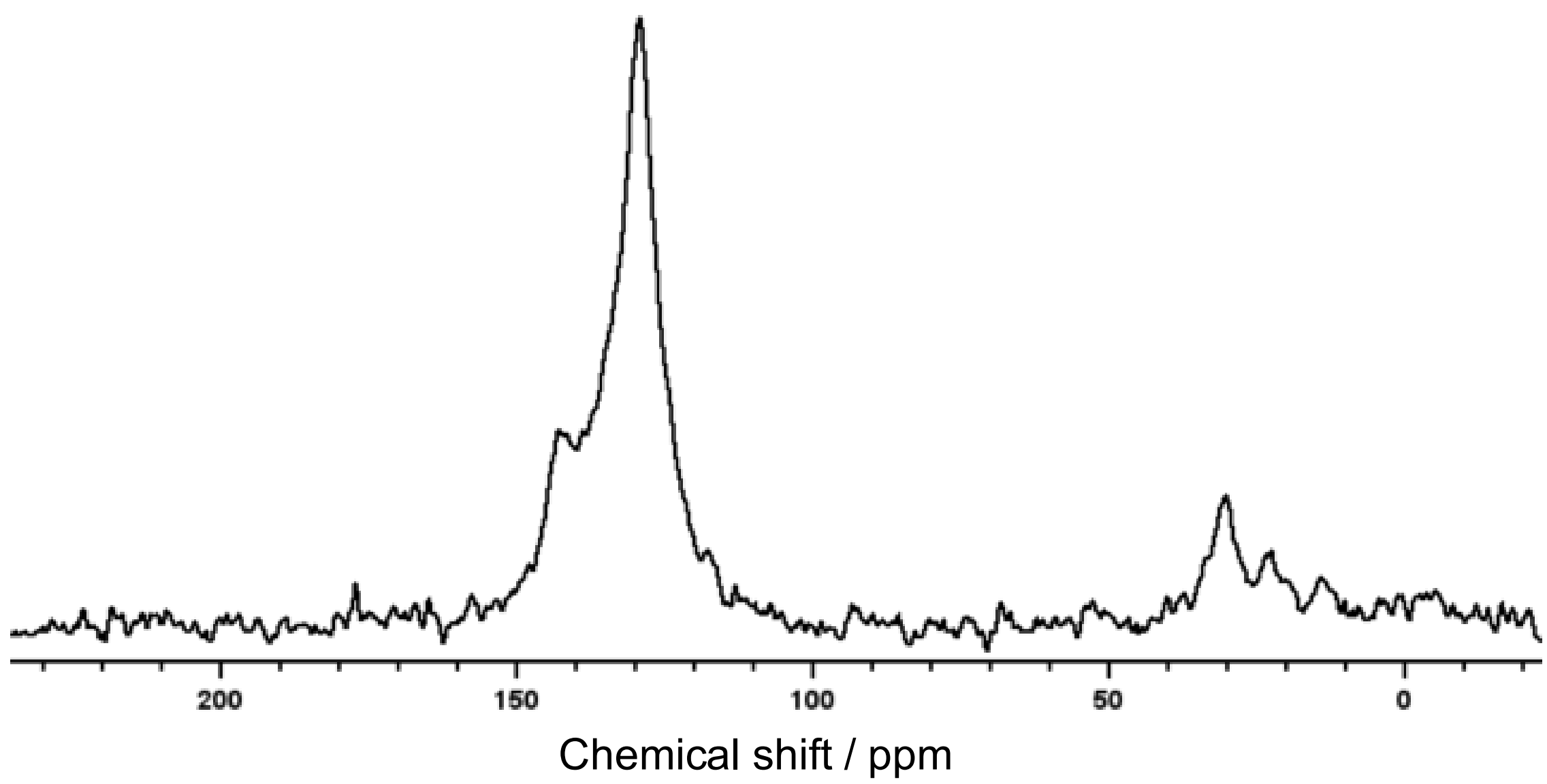

Figure SI.5: Repeat ^{1}H -^{13}C CP/MAS solid-state NMR spectrum of OSPC-0b.

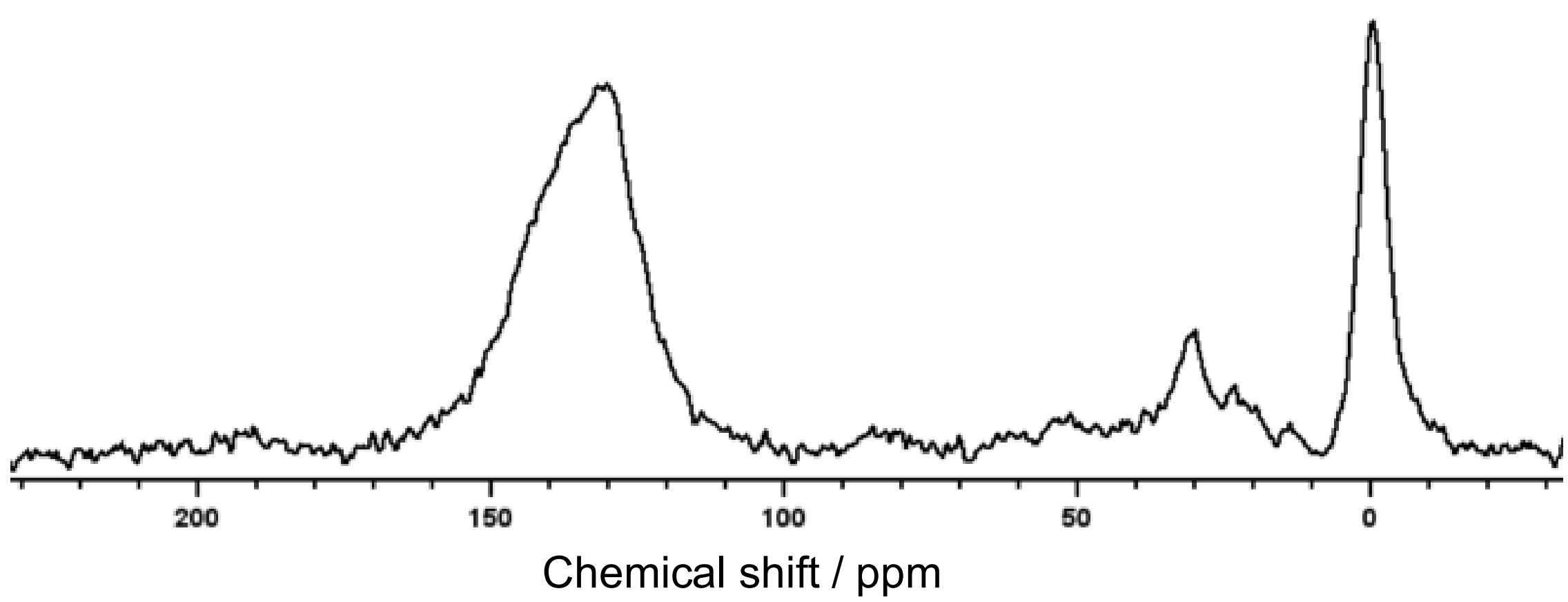

Figure SI.6: Repeat ^{1}H -^{13}C CP/MAS solid-state NMR spectrum of OSPC-1b.

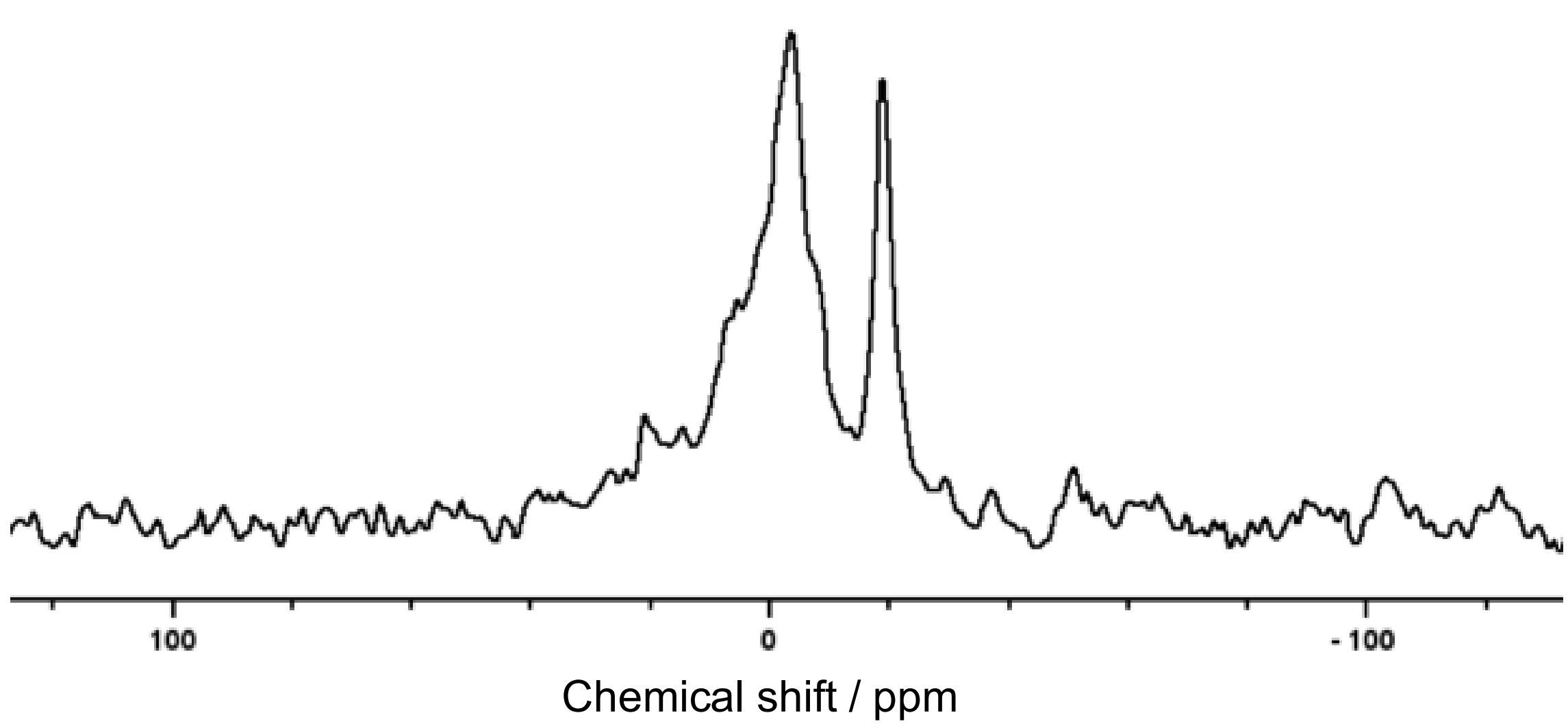

Figure SI.7: $^{29}Si\{^1H\}$ solid-state NMR spectrum of OSPC-1b.

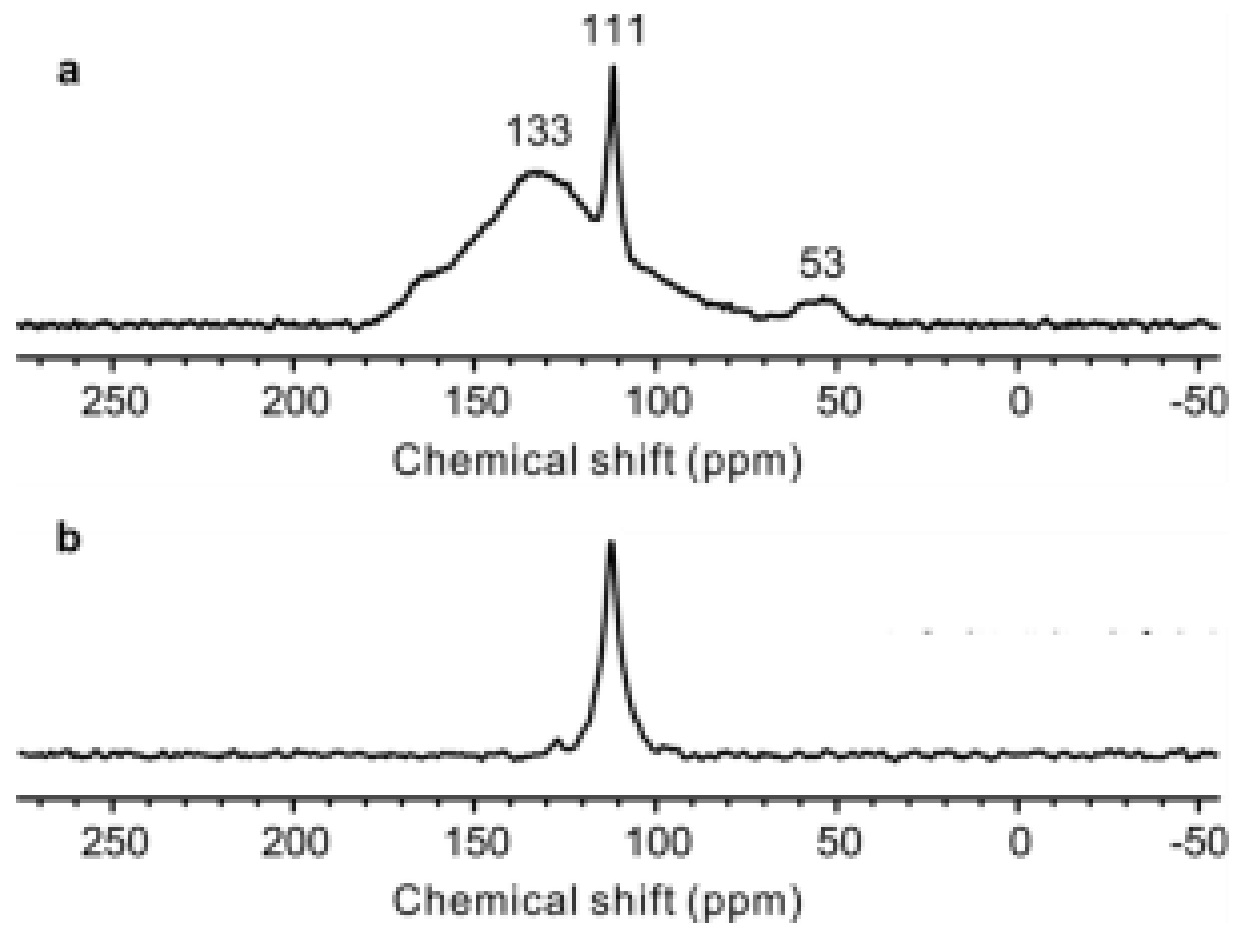

(a)

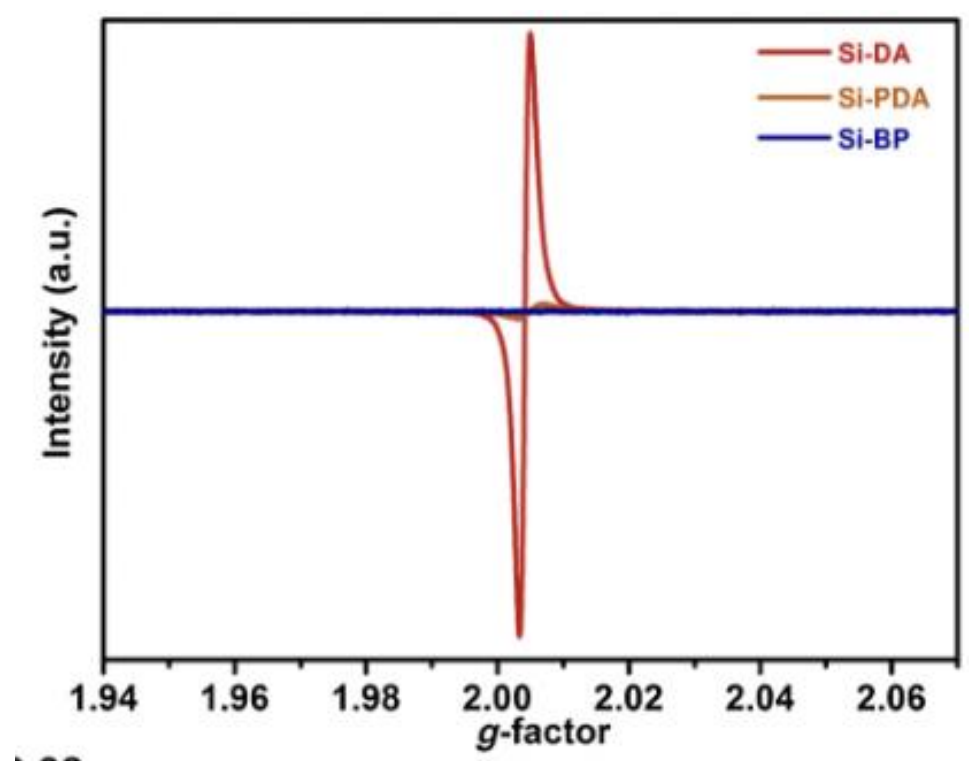

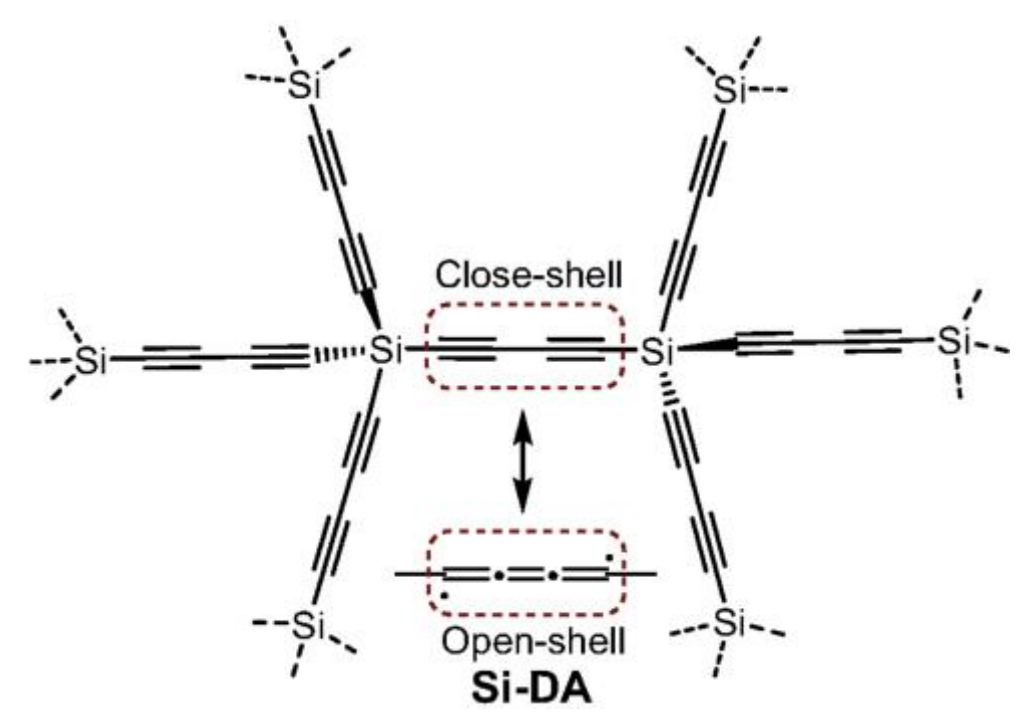

(b) **(c)**

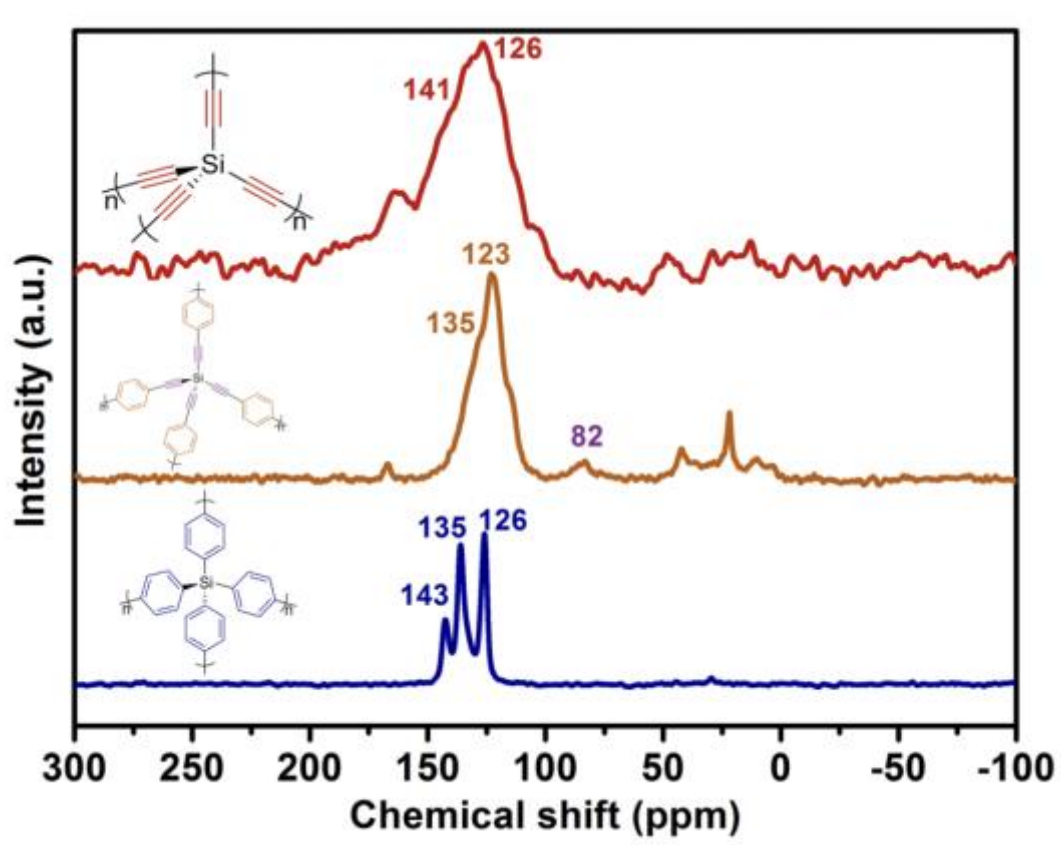

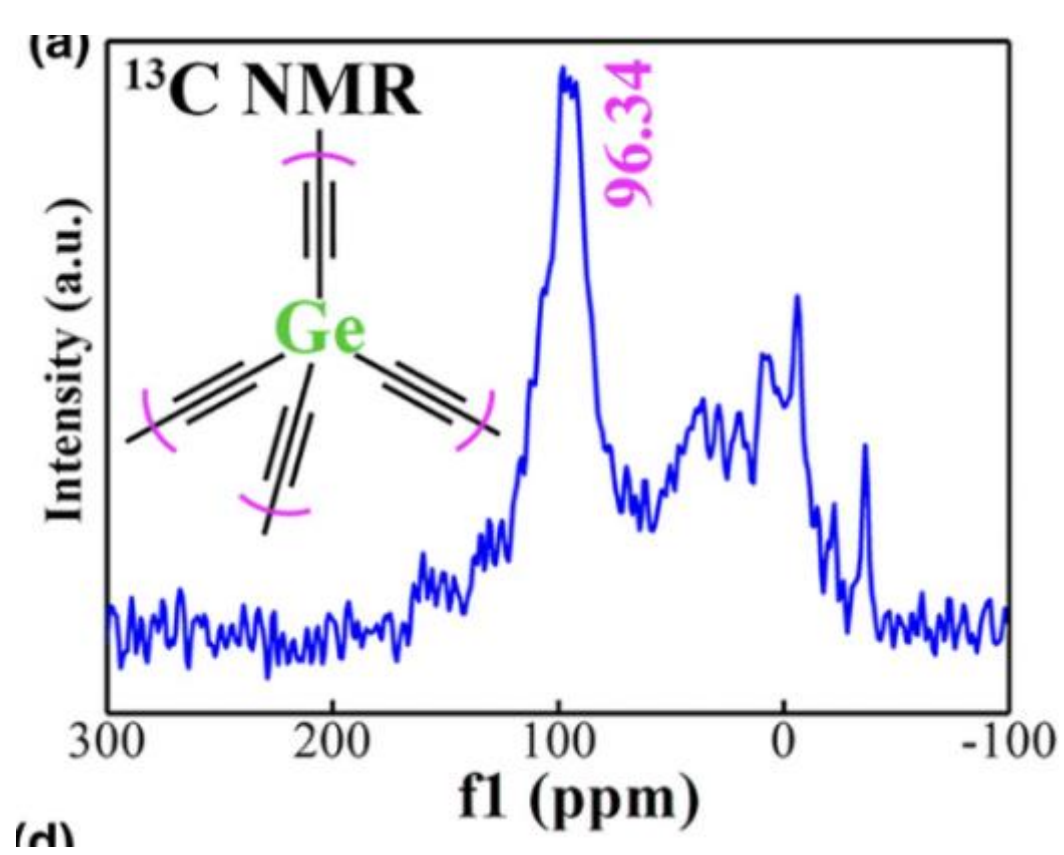

(d) **(e)**

Figure SI.8: Previous work (a) Single-pulse ^{13}C MAS NMR spectra of OSPC-1a and the PTFE NMR rotor reported for OSPC-1a.[1] The peak described as a PTFE rotor is unusual as NMR rotors are usually made out of zirconia, although sometimes a PTFE insert is used. This does not normally show up on ^{13}C NMR spectra as it is not excited via cross polarization, however the OSPC-1a spectra were acquired by direct excitation of ^{13}C nuclei so any PTFE present would

appear in the spectrum. **(b)** Solid state electron paramagnetic resonance (EPR) spectrum of Si-DA, an equivalent of OSPC-1a but with a silicon central sp^3 node rather than a carbon sp^3 node. Note that the synthesis of Si-DA is *via* the Eglinton homocoupling and so radical unreacted end groups are expected. **(c)** The open shell resonance structure proposed by Dong *et al*.[3] **(d)** ^{13}C NMR of Si-DA (red). **(e)** ^{13}C NMR of germanium-carbdiyne (GeCDY).[4]

Table SI.2: Summary of peak positions found for carbon with different hybridisation and potential carbon containing end groups. Details can be found in Table SI.3.

C Hybridisation	Peak Range (ppm)
sp	18-187
sp^2	111-150
sp^3	25-119
Radicals	65-105
Anions	74-161
Ketene	160

Table SI.3: Table of ^{13}C chemical shifts.

C Hybridisation	Chemical Environment	Peak (ppm)	Reference
sp^2	Phenyl (CMP)	124.0 – 139.0	5
sp	Alkyne (CMP)	82.2 - 95.9	
sp^3	Alkyl (CMP)	114.8-119.2	
sp^3	Nanodiamond (ND)	34.9	6
sp^3	Aliphatic (ND)	45	
sp^3	Alcohol (ND)	75	
sp^2	C-OH (GO)	60	7
sp^2	C-O-C (GO)	70	
sp^2	Graphite oxide (GO)	130	
sp^3	Diamond phase	35	8
sp^2	Fullerene shell	111	
sp^2	Activated carbon fibres	131	
sp^2	Graphite	119	
sp^3	Ultra-nanocrystalline diamond	35	
sp^3	C-OH	73	
sp^3	Alkyne	25-55	
sp^2	Pyrene (CMP)	124-138	9
sp^2	Phenyl (CMP)	124-141	10
sp	Alkyne (CMP)	90.2-91.4	
sp	Acetylenic	89.8	11
sp	Acetylenic - Pt	127-130	
sp^2	Phenyl (PAF)	120-150	12
sp^3	Methyl (PAF)	65	
sp^3	Glaser carbon	25	13
sp	Glaser carbon acetylenic	50	
sp^2	Glaser carbon	127	
sp	Glaser carbon Allenic carbyne	18	
sp^2	Glaser phenyl	127	
sp^3	Glaser phenyl	28	
sp	Glaser phenyl carbyne	40 and 85	
sp	Glaser phenyl cumelenic carbyne	187	
sp^2 *radicals*	Phenalenyl	115 – 14 and 140-150	14
sp anion	Acetylide (Pt)	83-92, and 101	15
sp^3 *radicals*	3,4-dimethylenethiophene	65	14
sp^3 *radicals*	3,4-dimethylenethiophene intermediate (terminal carbon)	105	14

sp^3 *anion*	Methanide	73.6	[16]
sp^2 *anion*	Ph-C(-)-N (Na+)	161	[17]
sp^2	Ketenyl (metal coordinated ligands)	210.9<br>236 and 242	[18]<br>[19]
sp^2	Ketene	160	[20]

Section 2. Computational

Ambuild

The Amorphous Builder (Ambuild) code was used to generate our amorphous OSPC models. Ambuild is Python-based, so is able to easily interface with other codes such as HOOMD-blue (which is used as our geometry optimisation engine throughout),[21-23] and Poreblazer,[24, 25] which can be used to generate simulated porosity analysis of structural models. Ambuild is also GPU-based, meaning that we benefit from an increased simulation speed, and it allows us to generate larger structures than a solely CPU-based code within the same timescale. This is especially important when considering amorphous structures such as OSPC-1, where we do not want our simulated models to be tainted by artificial crystallinity that would not occur in the experimental sample. More details about the Ambuild code can be found in references [1, 26-28].

To generate our amorphous OSPC-1 models, we firstly seed one tetraethynylmethane monomer into a cuboid unit cell, to act as a starting point. This monomer is defined such that the terminal carbon atoms are *endGroups*, which will form part of new bonds to other monomers, and the attached hydrogen atoms are *capAtoms*, which are lost on forming a new bond between two suitable *endGroups*. We then set up a loop comprised of: 1. the Ambuild growBlocks methodology, which finds an available *endGroup* within the cell, and bonds a second monomer to it; 2. the Ambuild zipBlocks methodology, which searches for *endGroups* within the existing building blocks that are available to bond by changing the pre-determined bond distance and bond angle margins within which two *endGroups* must be to form a bond. Here we used relatively tight criteria of 2 Å and 60º; 3. a geometry optimisation within HOOMD-blue; and 4. a molecular dynamics step using an NVT ensemble. We continue this loop until we have an OSPC-1 cluster with 442 tetraethynylmethane monomer units. At this point we no longer continue with the growBlocks (step 1) but continue with steps 2, 3, and 4 until no more bonds are possible to form within the specified criteria. This ensures that the maximum number of end groups within the cluster have formed within this strategy. The resulting cluster has 442 tetraethynylmethane nodes and ~60% of end groups remaining that are all positioned on the outer surface of the cluster.

Gaussian

The minimum energy ground-state optimal geometry of each fragment model was calculated (confirmed as such by a harmonic wavenumber analysis) using B3LYP/6-311G calculations in Gaussian 09.[29-34] In the case of the fragment models shown in Figure 2(g) and 2(h), the basis set used for the Cs and Cu atoms was LanL2DZ,[35-38] with 6-311G still being used for the C and H atoms. The NMR spectra of the optimised fragment models were then calculated using the same model chemistry, computing the properties using the CSGT method.[39-41] We also computed the two-step spin–spin coupling constants, which are described in detail by Deng *et al.* in reference [42]. We then shifted the isotropic chemical shielding of each carbon atom relative to the tetramethylsilane (TMS) standard to give the chemical shifts, where:

<u>Carbon NMR:</u>

Chemical shift (ppm) = 189.72 ppm – isotropic chemical shielding (ppm)

Once we had our raw chemical shift values relative to TMS, we then applied a uniform shift to each atom in the fragment models, so that our simulated spectrum of the TMS-protected tetraethynylmethane monomer (which we used as a reference molecule) was in line with the experimental NMR spectrum (please see Figure SI.5 for the spectra). For the Gaussian fragment models, this shift equated to:

Shifted x value = (0.95*x value relative to TMS) – 4.17.

<u>Silicon NMR:</u>

Chemical shift (ppm) = 390.41 ppm – isotropic chemical shielding (ppm)

Shifted x value = (0.95*x value relative to TMS).

CASTEP

The structures of the periodic models were optimised and had their NMR spectra calculated using CASTEP version 8.0[43] on the High-End Computing facility at Lancaster University. The generalised gradient approximation (GGA) Perdew–Burke–Ernzerhof (PBE)[44] was used as the exchange-correlation functional throughout the optimisation and NMR calculations, and ultrasoft pseudopotentials were used to describe the core-valence interactions.[45, 46] The gauge-included projector augmented wave (GIPAW) algorithm was used during the NMR calculations to construct the all-electron wave function in the presence of the magnetic field.[47, 48] A plane wave basis set cut-off energy of 50 Ry and Monkhorst–Pack grid of 1×1×1 (to specify the points in k-space where the Brillouin zone should be sampled)[49] were employed throughout the geometry optimisation and NMR calculations. The elec_energy_tol (for accepting convergence of the total energy) was set to 1×10^{-9} eV. The geom_energy_tol (for accepting convergence of the free energy per atom during the geometry optimisation) was set to 1×10^{-4} eV. The geom_force_tol (for accepting convergence of the ionic forces during the geometry optimisation) was set to 5×10^{-2} eV/Å. The geom_disp_tol (for accepting convergence of the ionic displacement during the geometry optimisation) was set to 1×10^{-3} Å. Finally, the geom_modulus_est (giving an estimate of the bulk modulus of the system) was set to 174 GPa. To ensure that the k-point density and plane-wave cutoff energy were large enough to model our systems with numerical reliability, we undertook convergence tests to show how the final energy and ^{13}C isotropic shielding changed as a function of the number of k-points and plane-wave cutoff energy. These are shown in Table SI.4 and Figure SI.9.

Table SI.4: k-point convergence for the CASTEP models. C1 and C4 are two carbon atoms within different chemical environments in the system, chosen to confirm the convergence of isotropic shielding as a function of k-point density.

**k-point grid size**	1×1×1	2×2×2
**Final energy / eV**	–2882.71	–2882.71
**^{13}C isotropic shielding for C1 / ppm**	146.93	146.98
**^{13}C isotropic shielding for C4 / ppm**	97.62	97.38

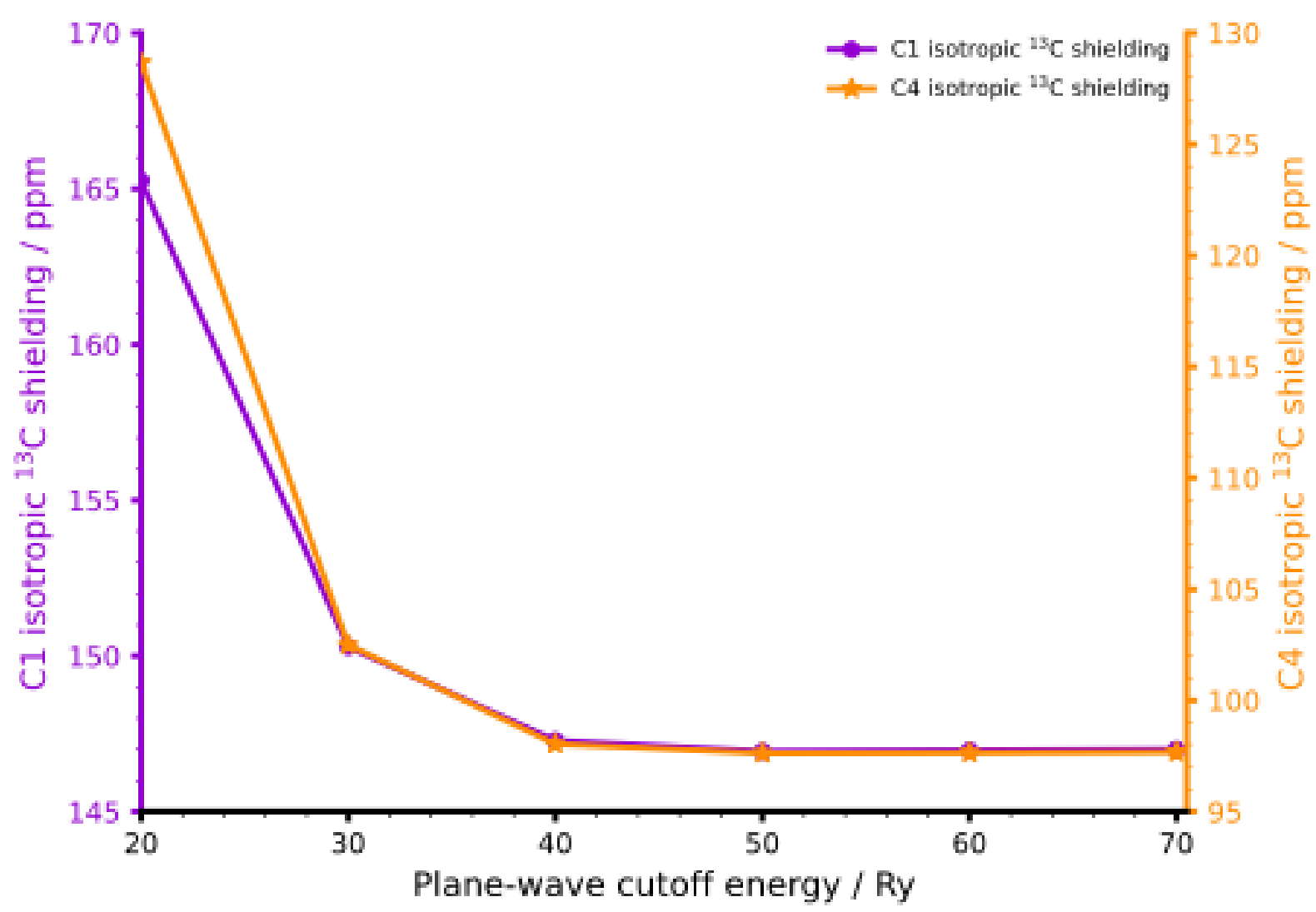

Figure SI.9: Plane-wave cutoff energy convergence for the CASTEP models. C1 (purple) and C4 (orange) are two carbon atoms within different chemical environments in the system, chosen to confirm the convergence of isotropic shielding as a function of plane-wave cutoff energy.

<u>Carbon NMR:</u>

As for the Gaussian fragment models, we shifted the isotropic chemical shieldings relative to TMS (TMS shielding value = 178.92 ppm), and shifted relative to the TMS-protected tetraethynylmethane reference, where, for the CASTEP periodic models, the shift equated to:

Shifted x value = x value relative to TMS – 8.67.

<u>Silicon NMR:</u>

Chemical shift (ppm) = 321.74 ppm – isotropic chemical shielding (ppm).

Section 3. TES validation of method

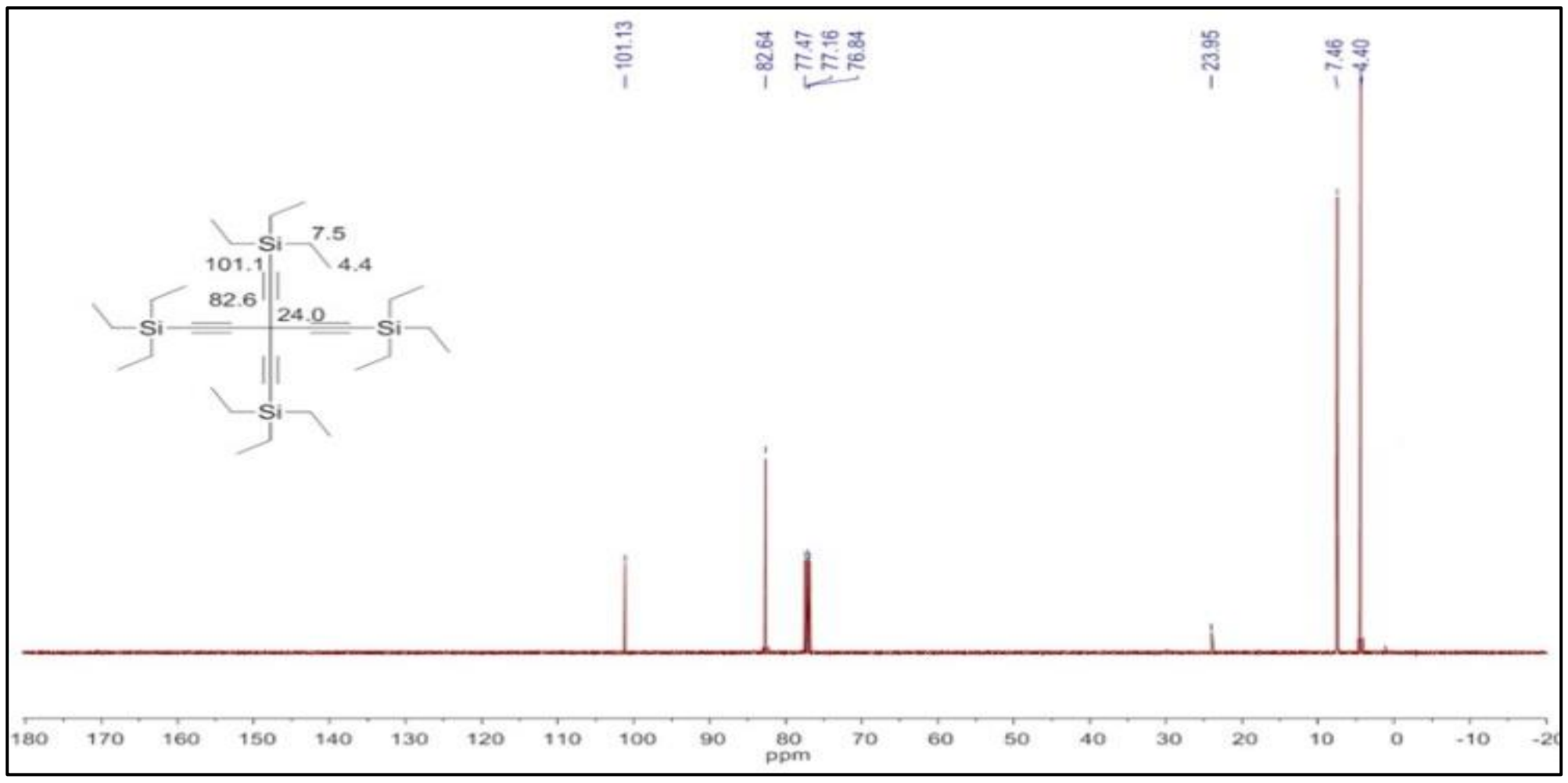

Figure SI.10: NMR spectrum of triethylsilyl (TES)-protected tetraethynylmethane – experiment ($CDCl_3$ solvent).[1]

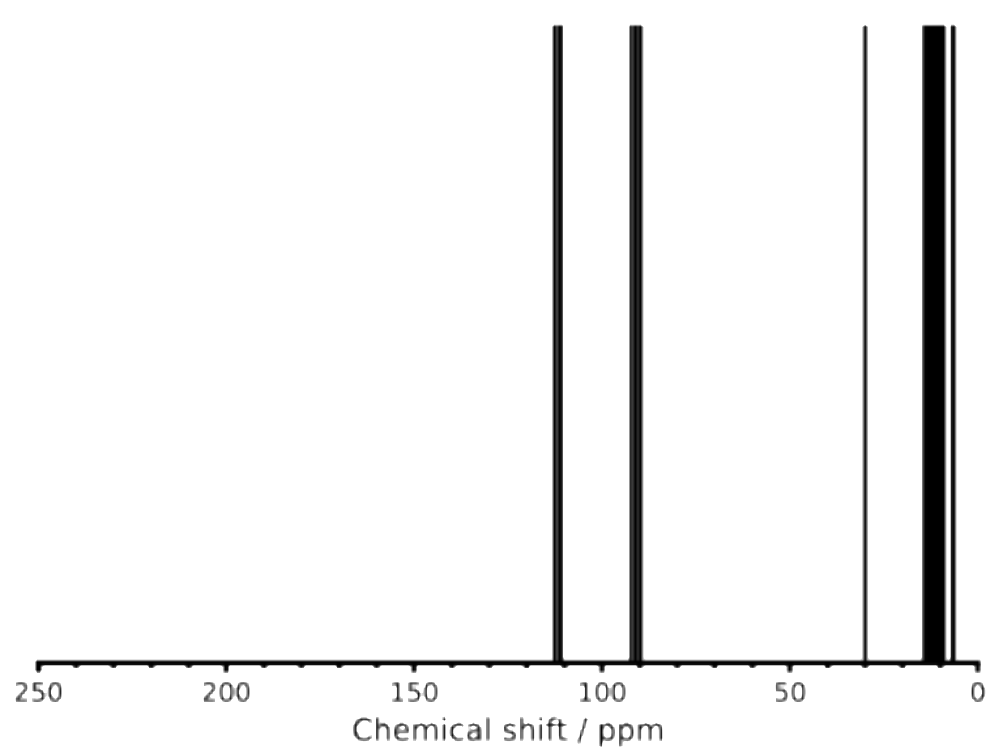

(a)

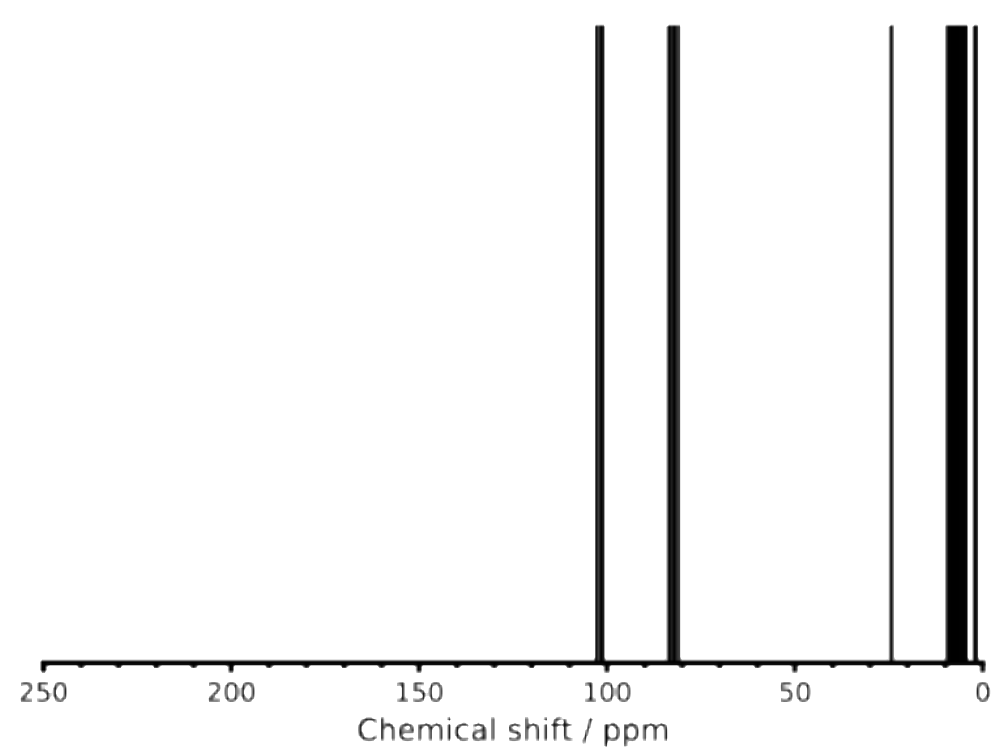

(b)

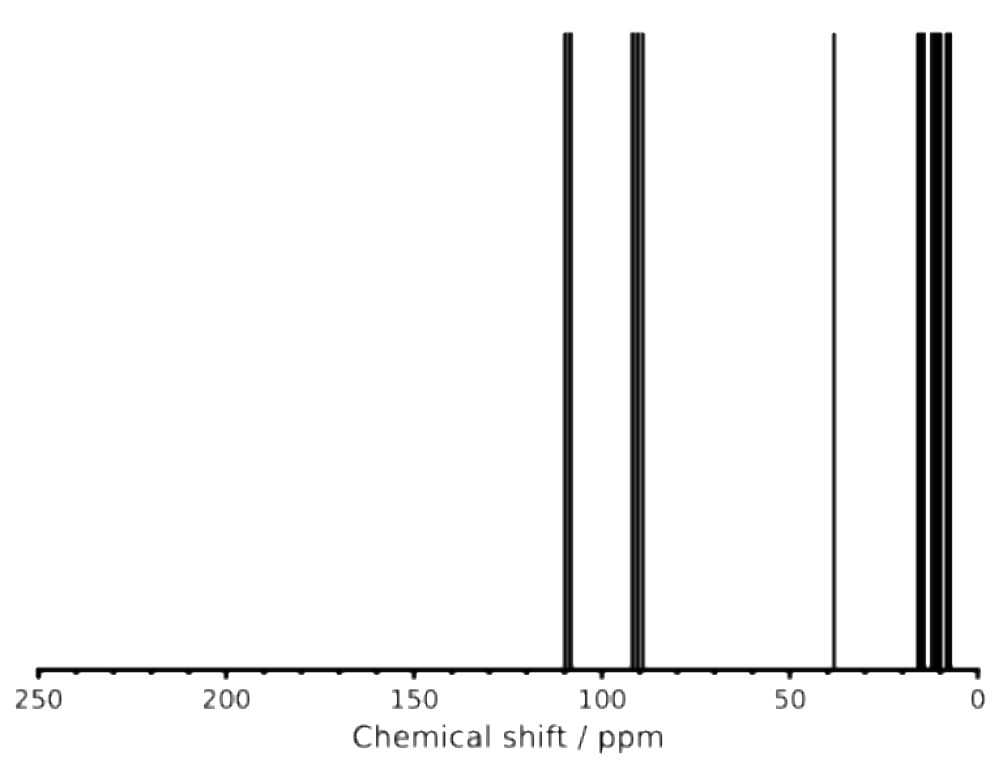

(c)

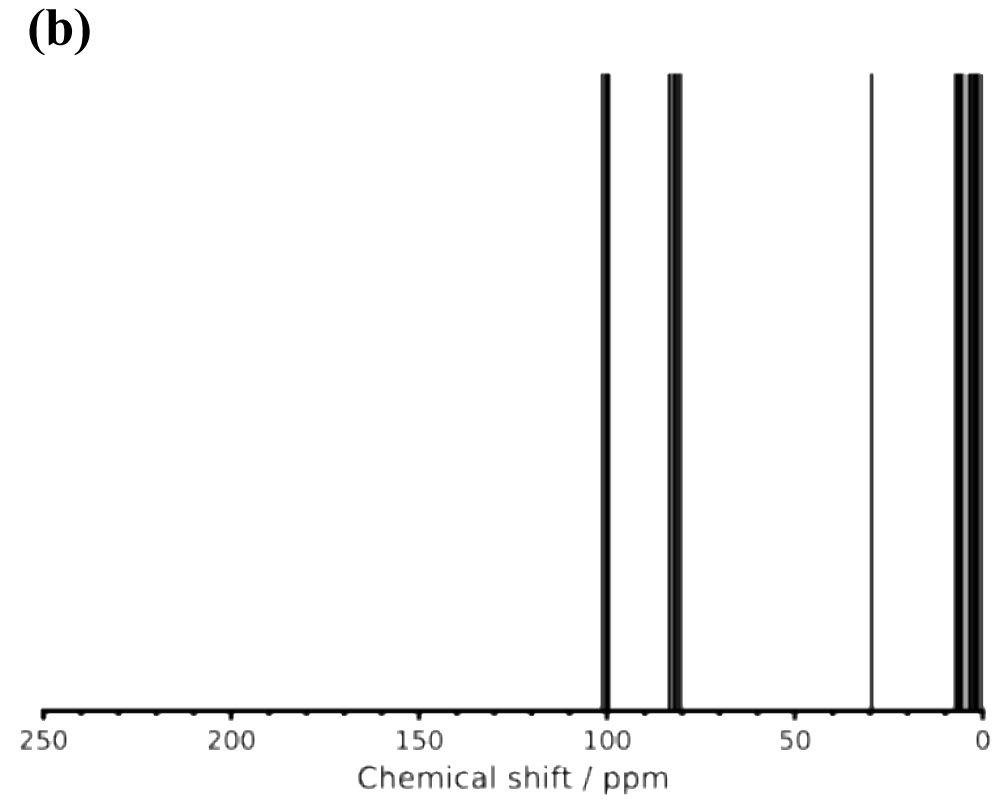

(d)

Figure SI.11: **(a)** NMR spectrum of TES-protected tetraethynylmethane ($CHCl_3$ solvent included) calculated using Gaussian. **(b)** NMR spectrum calculated using Gaussian with the H of CH_x groups averaged and the chemical shift fitted using new shift = (x*0.95) – 4.17. **(c)** NMR spectrum of TES-protected tetraethynylmethane calculated using CASTEP. **(d)**

NMR spectrum of TES protected-tetraethynylmethane calculated using CASTEP with the H of CH_x groups averaged and the chemical shift fitted using new shift = x – 8.67. For **(c)** and **(d)**, the $CHCl_3$ solvent was not included as it made negligible change to the Gaussian models.

Section 4. Structural Fragment Models and their NMR spectra

All fragment spectra are determined using Gaussian and the resulting spectra treated as described above.

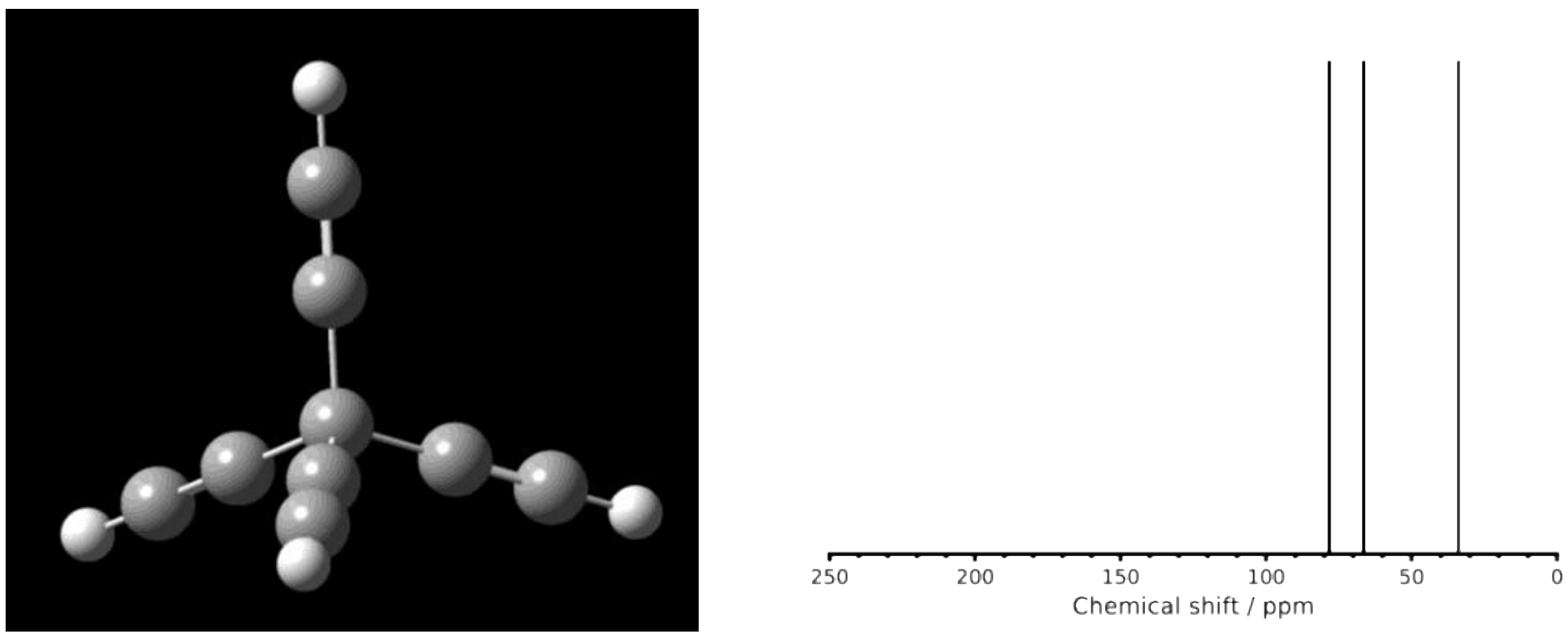

Figure SI.12: Calculated NMR spectrum of tetraethynylmethane.

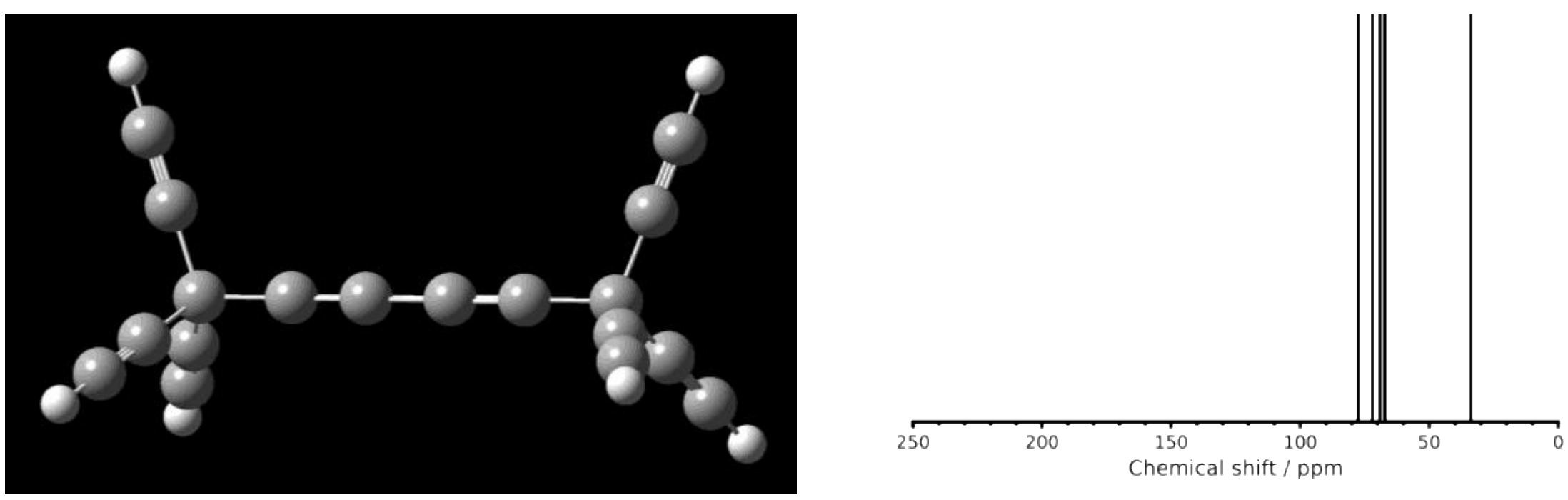

Figure SI.13: Calculated NMR spectrum of a dimer of tetraethynylmethane.

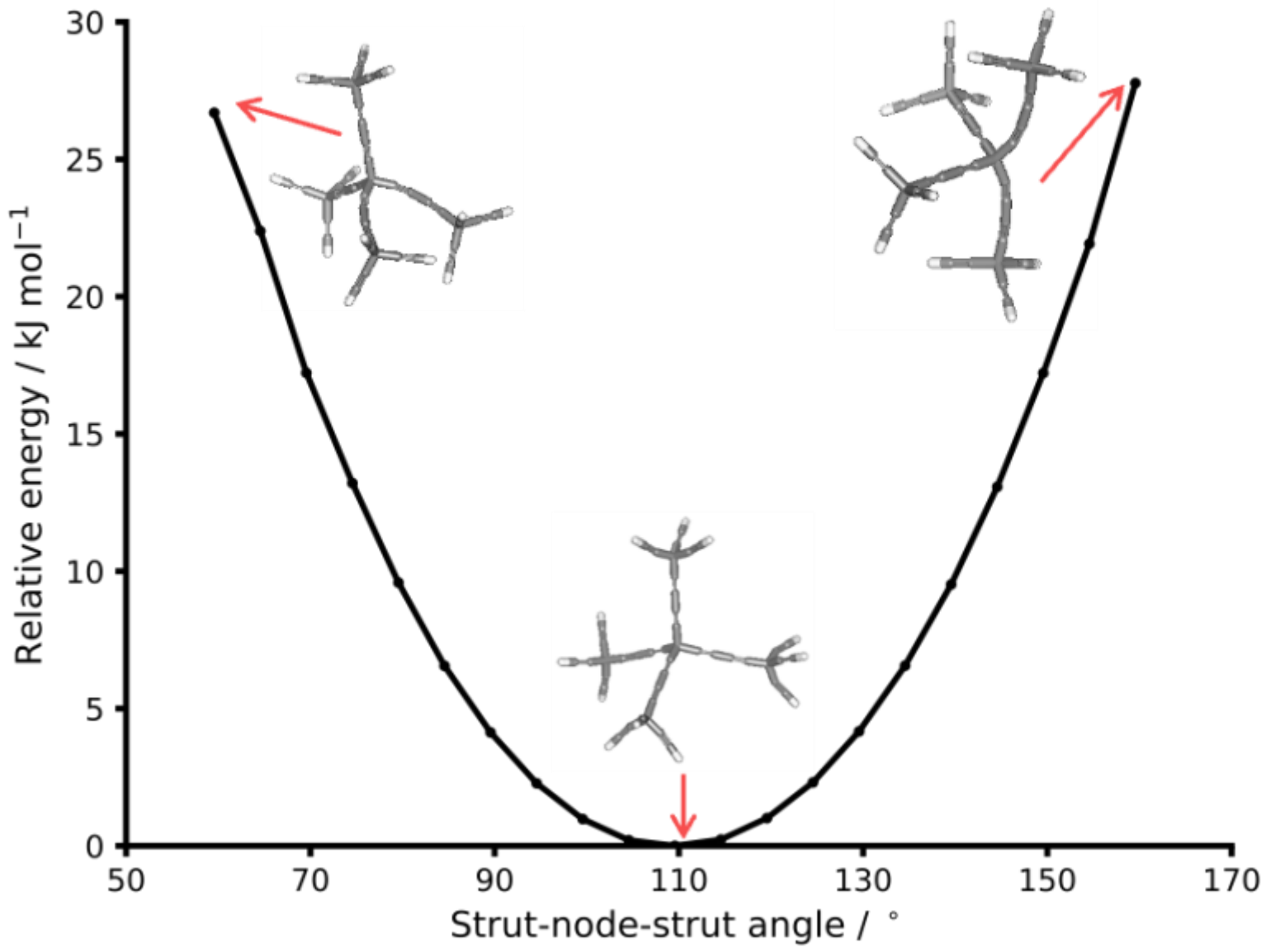

Figure SI.14: Calculated energy of a scan of the strut-node-strut angle of the OSPC-1 fragment, showing the structures of the OSPC-1 model with strut-node-strut angles of 60°, 110° and 160° as insets.

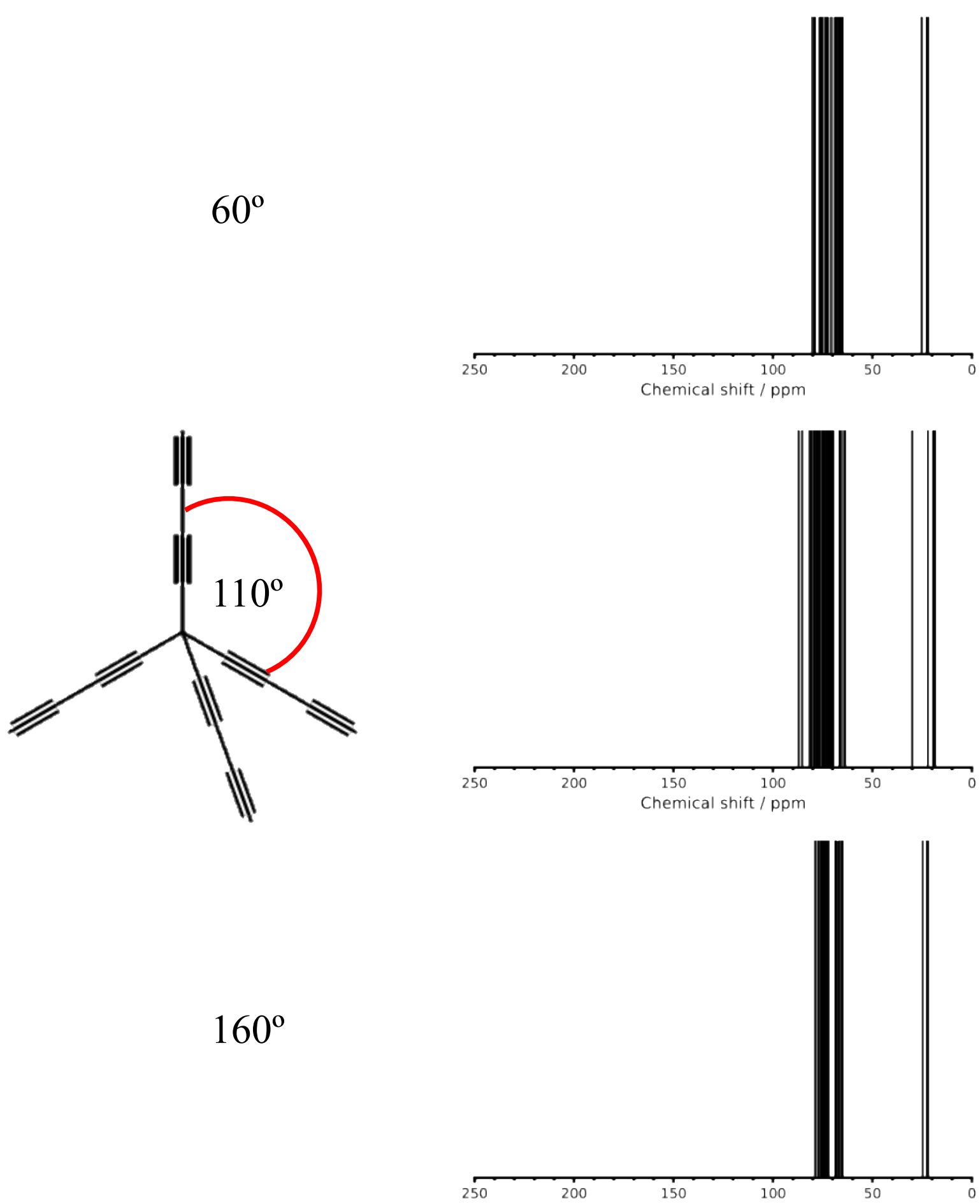

Figure SI.15: Calculated NMR spectra of the tetramer with three different strut-node-strut angles of the OSPC-1 fragment.

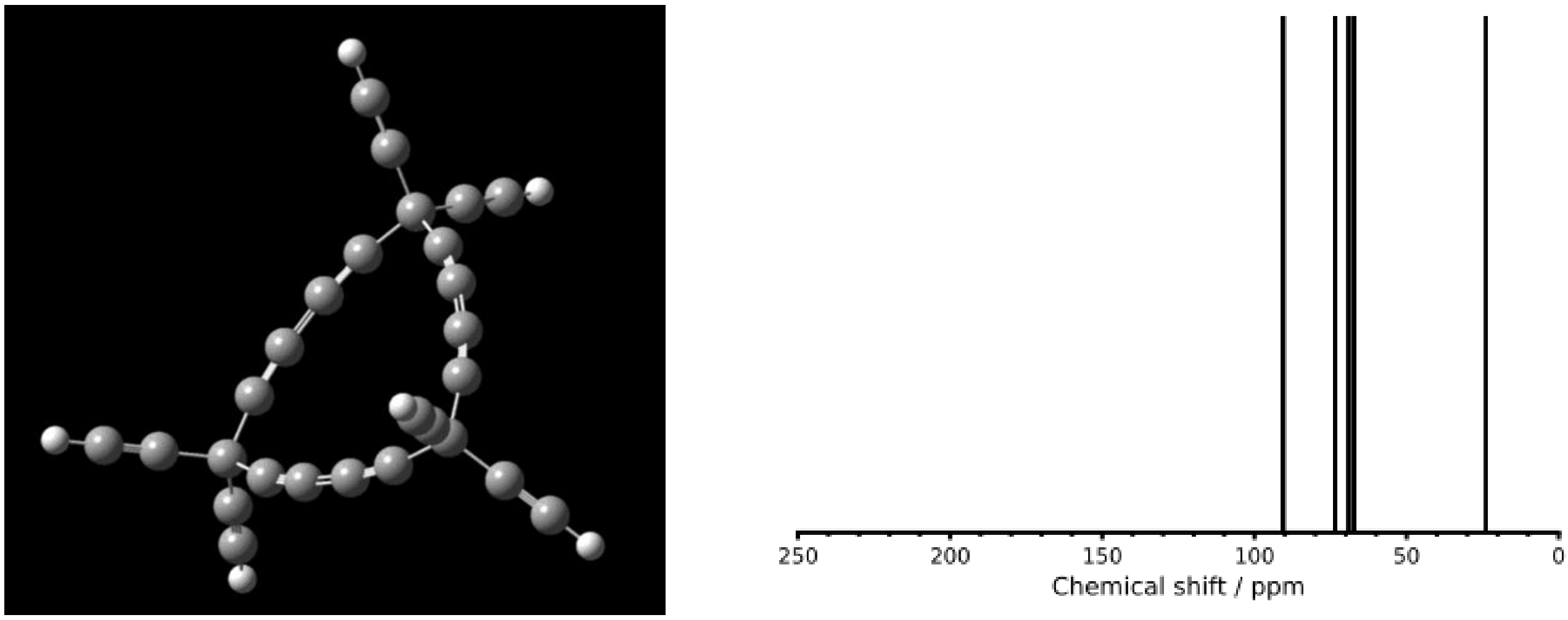

Figure SI.16: Calculated NMR spectrum of the 3-node ring OSPC-1 fragment.

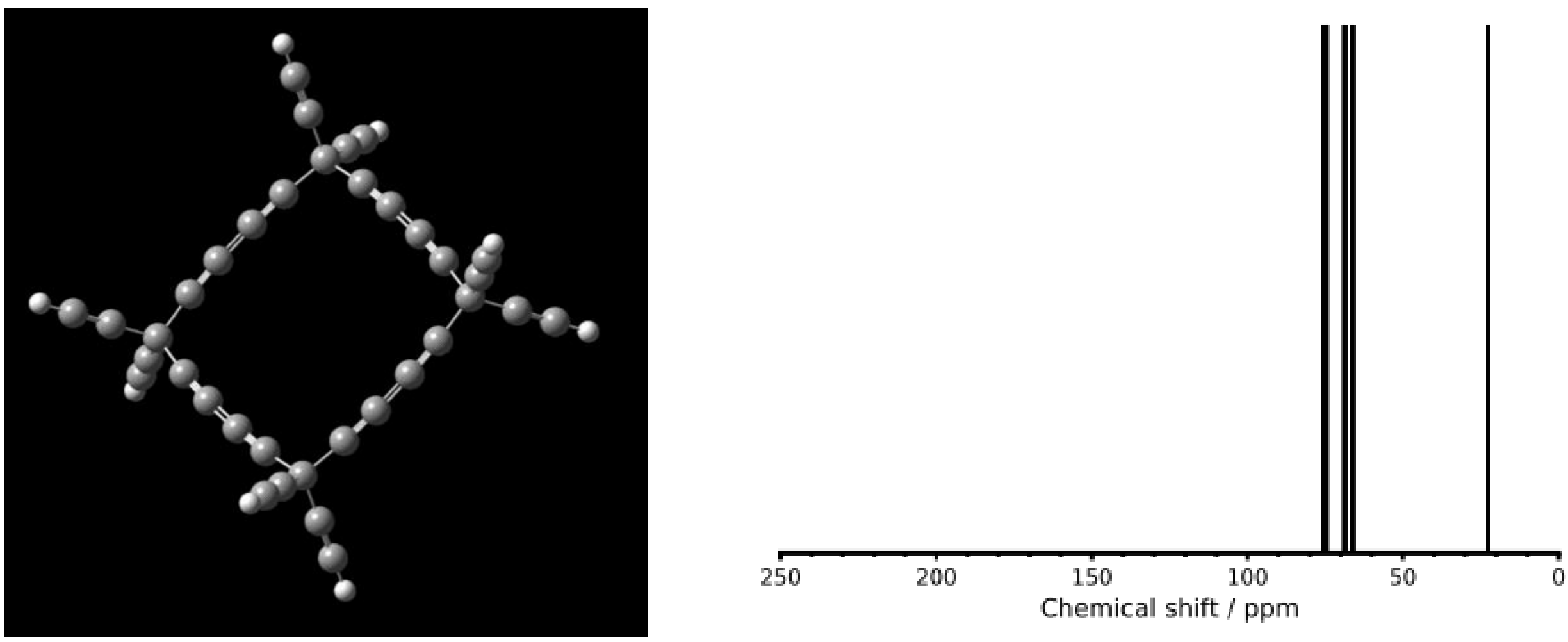

Figure SI.17: Calculated NMR spectrum of the 4-node ring OSPC-1 fragment.

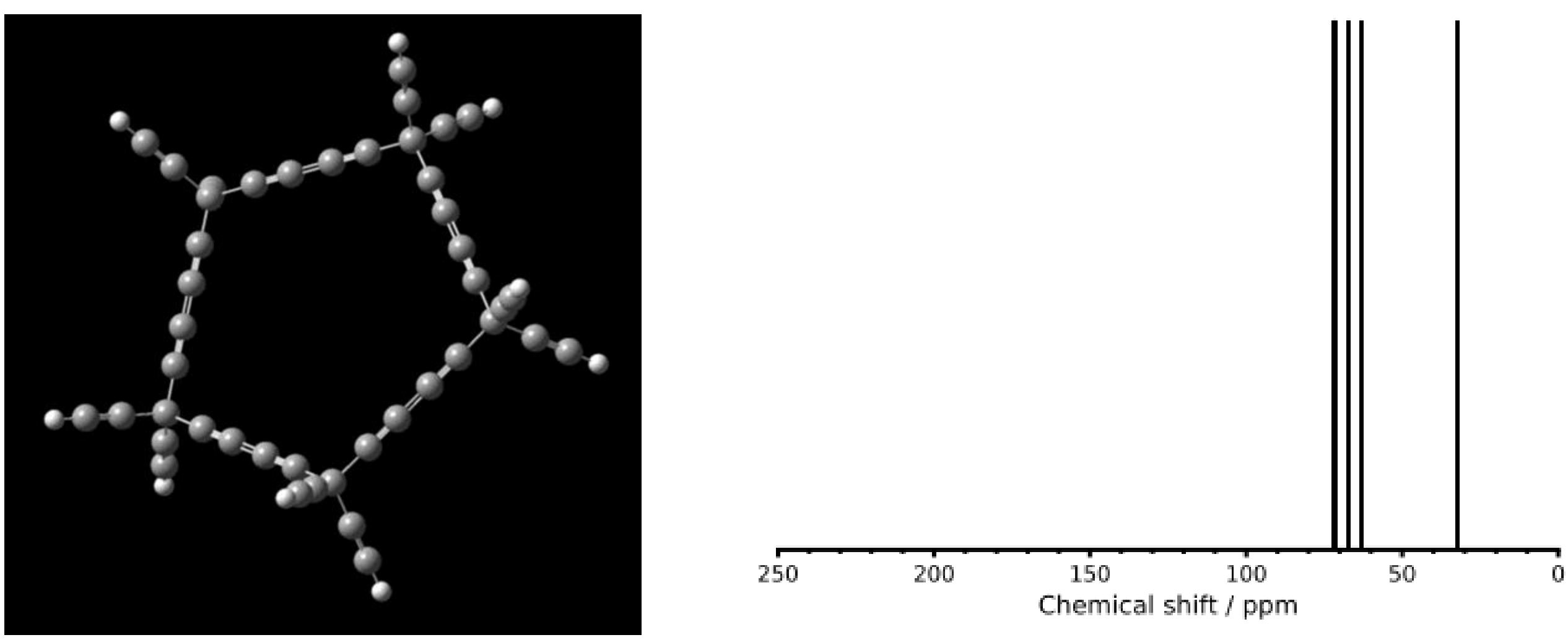

Figure SI.18: Calculated NMR spectrum of the 5-node ring OSPC-1 fragment.

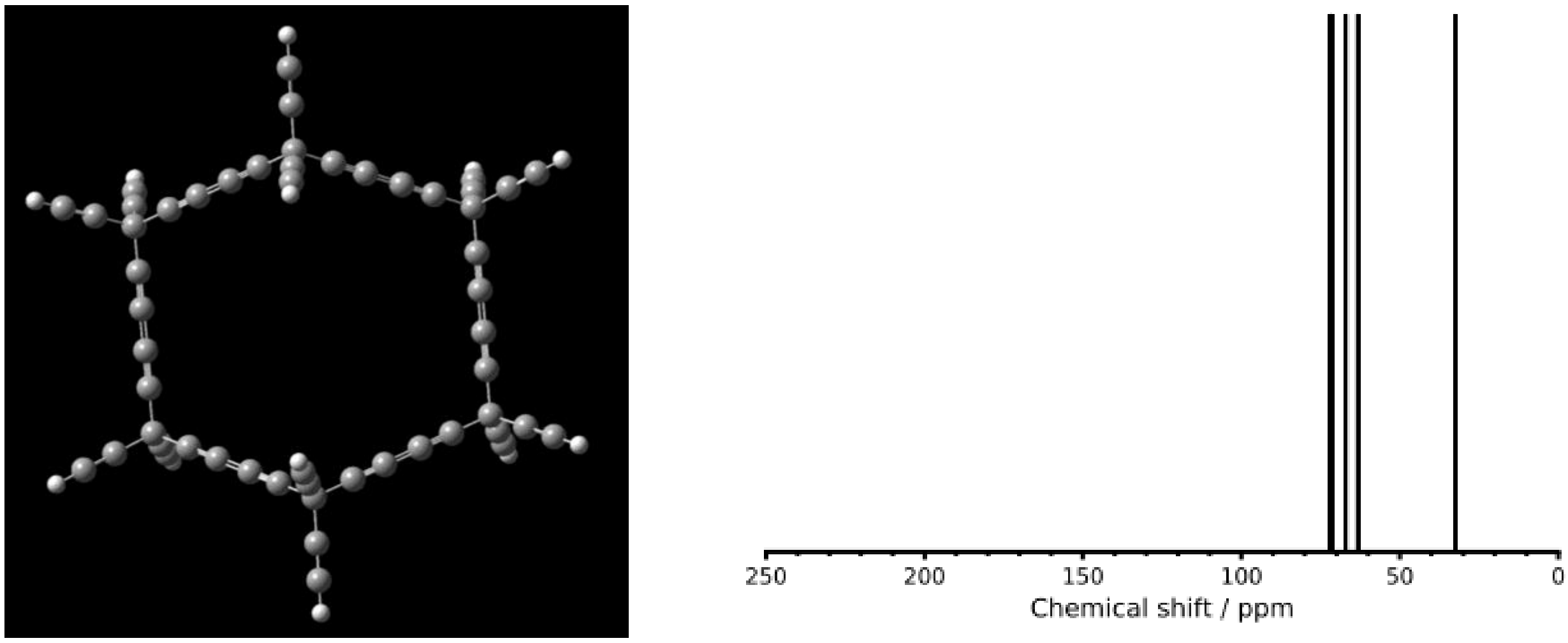

Figure SI.19: Calculated NMR spectrum of the 6-node ring OSPC-1 fragment.

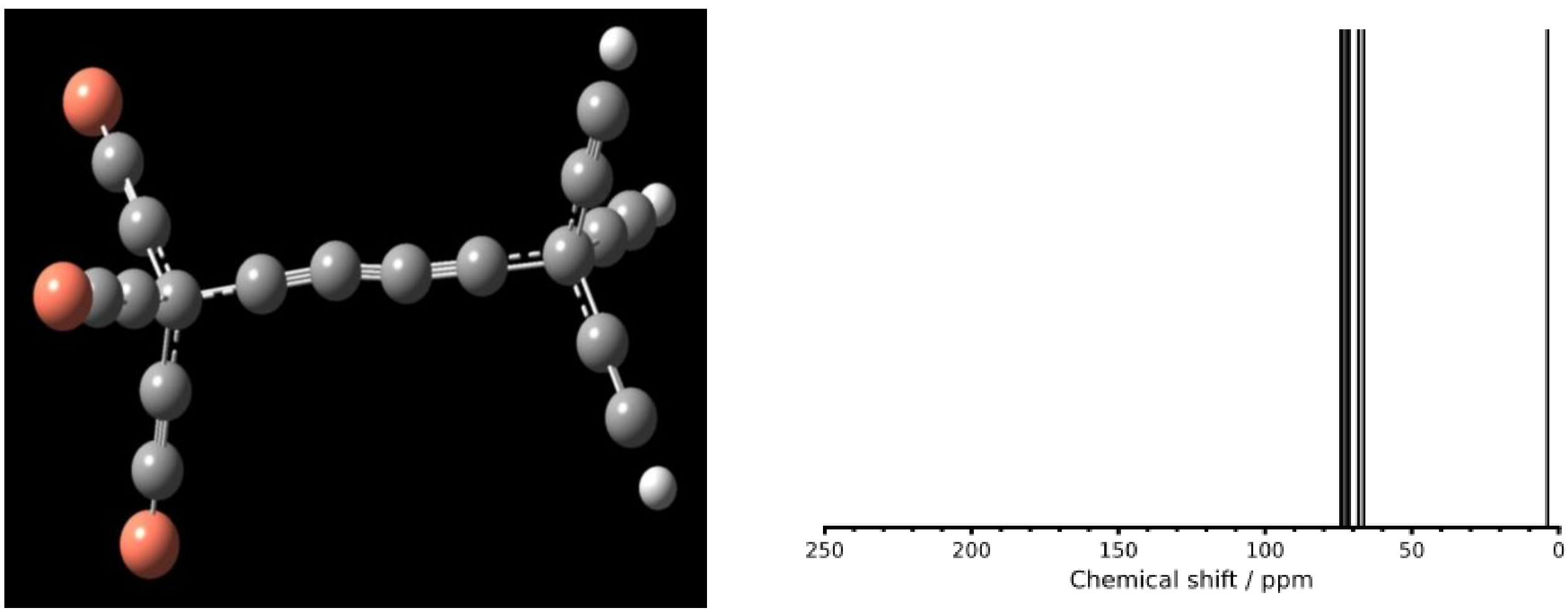

Figure SI.20: Calculated NMR spectrum of the OSPC-1 dimer fragment with a Cu, a potential end group within the synthesis of OSPC-1a.

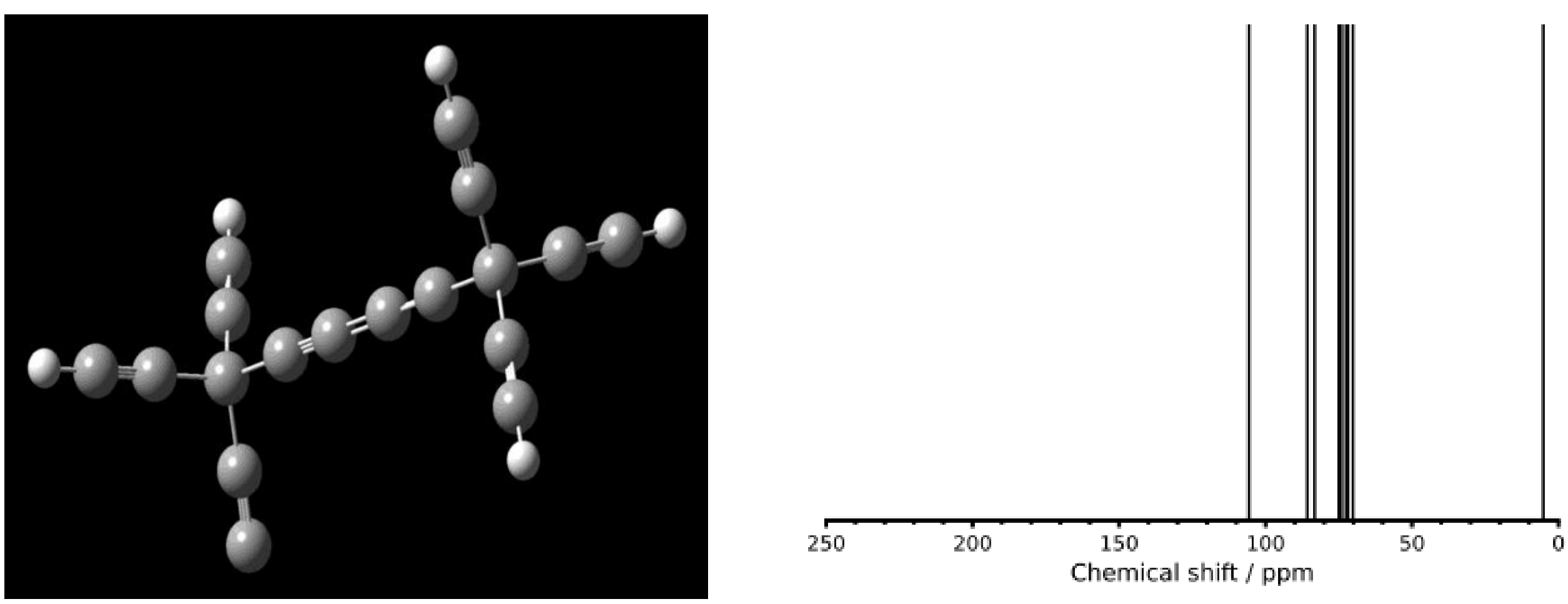

Figure SI.21: Calculated NMR spectrum of the OSPC-1 dimer fragment with a radical, a potential end group within the synthesis of OSPC-1a.

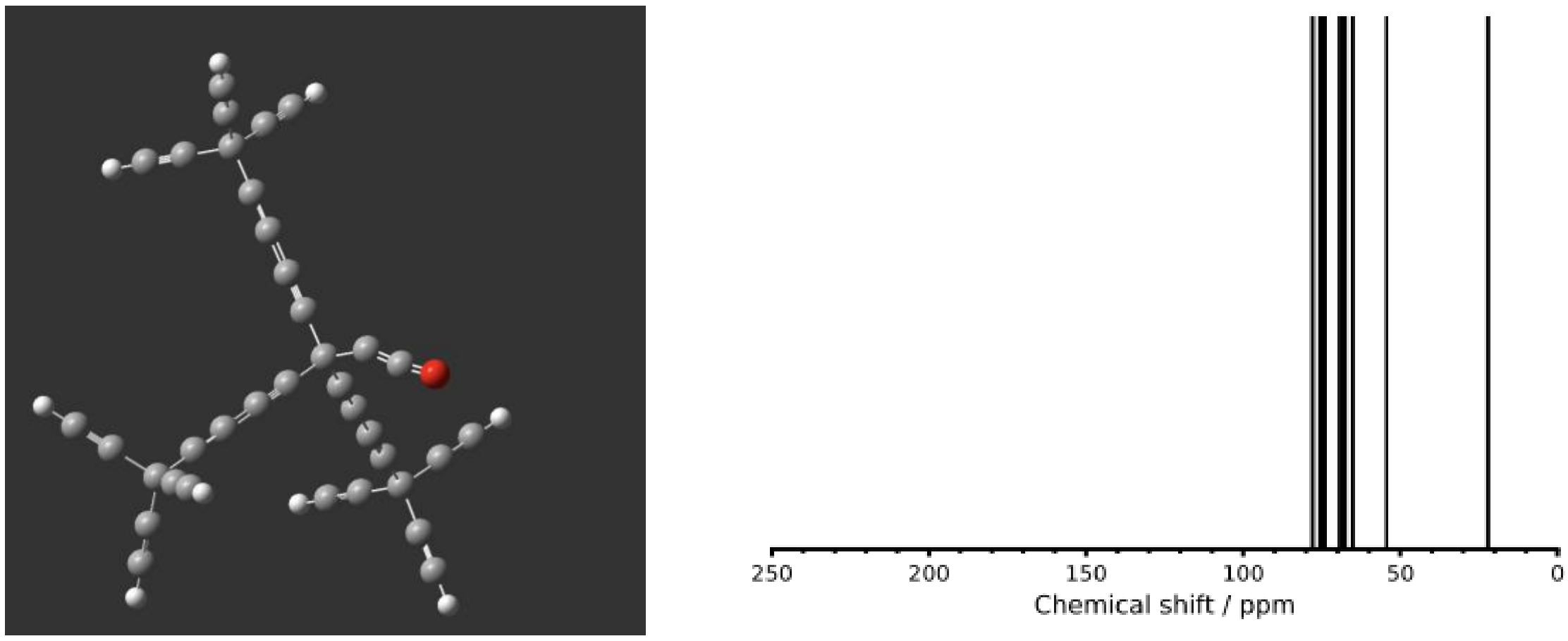

Figure SI.22: Calculated NMR spectrum of the OSPC-1 fragment with a ketenyl radical, a potential end group within the synthesis of OSPC-1a.

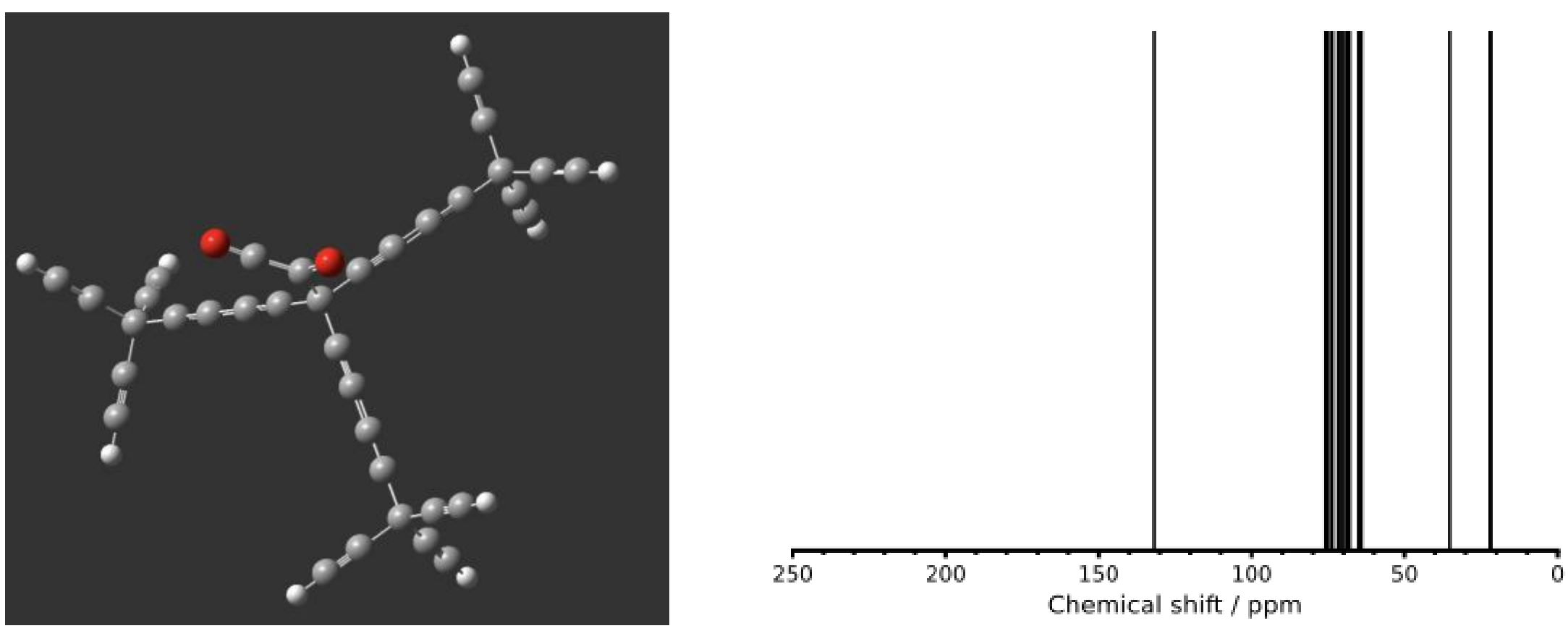

Figure SI.23: Calculated NMR spectrum of the OSPC-1 fragment with a C(O)CO functional group, a potential end group within the synthesis of OSPC-1a.

Section 5. Structural Periodic Models and their NMR spectra

All fragment spectra are determined using CASTEP and the resulting spectra treated as described above.

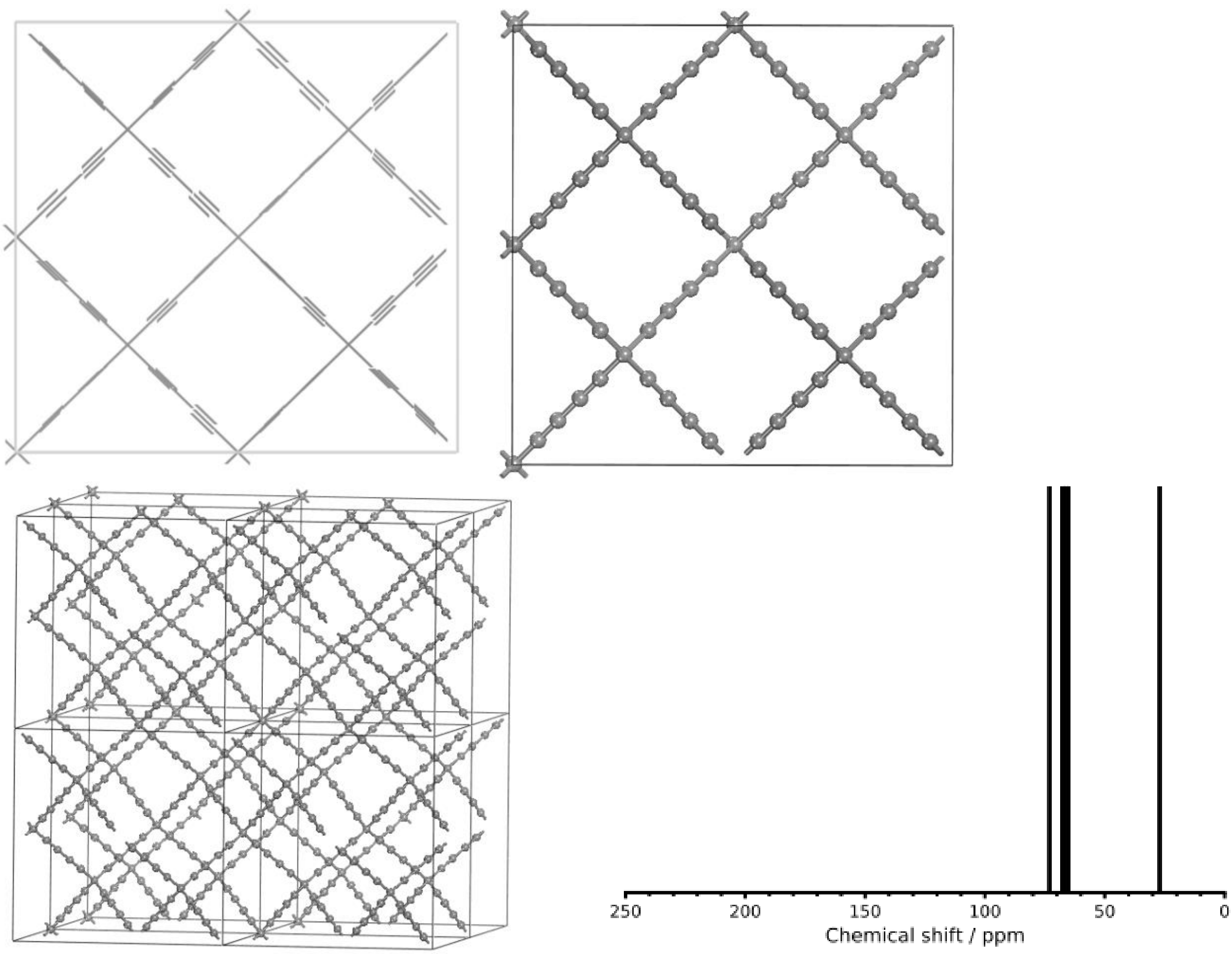

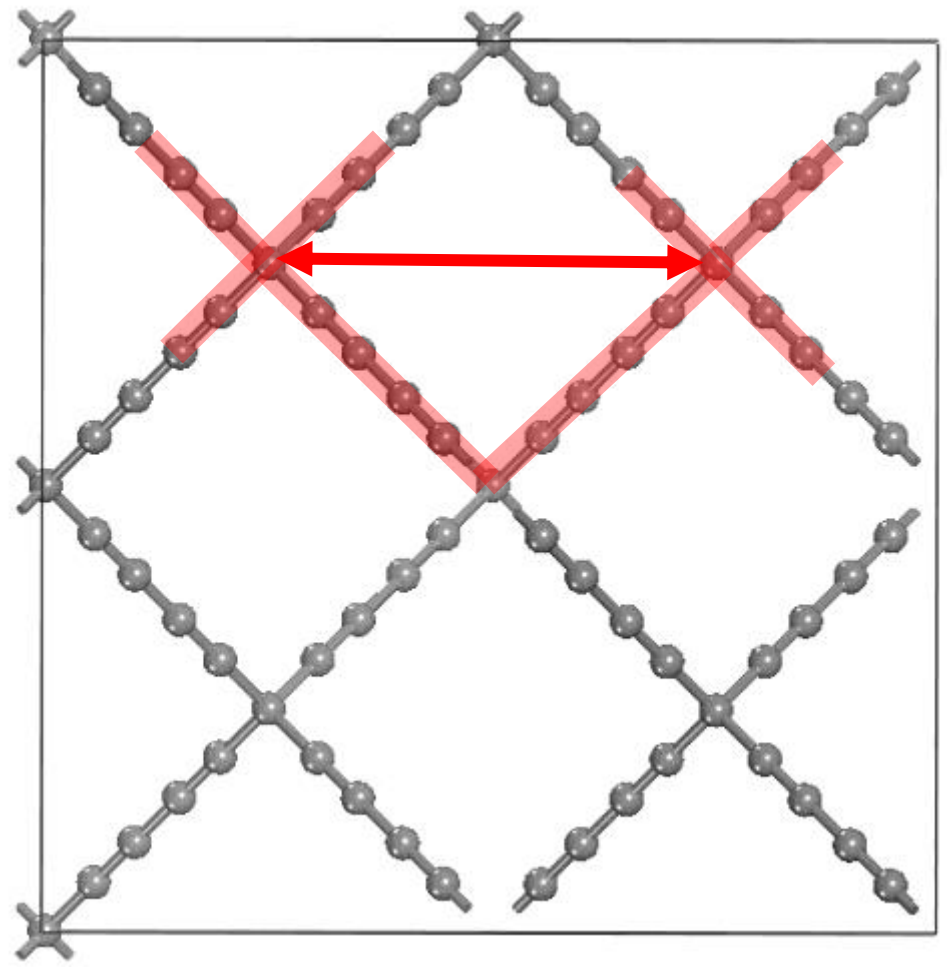

Figure SI.24: Structure and calculated NMR spectrum of OSPC-1 with an idealised *dia* topology. Red lines highlight the structural feature that we propose is present within the pore size distribution resulting in a peak at ~1.2 nm from the distance indicated by the red arrow.

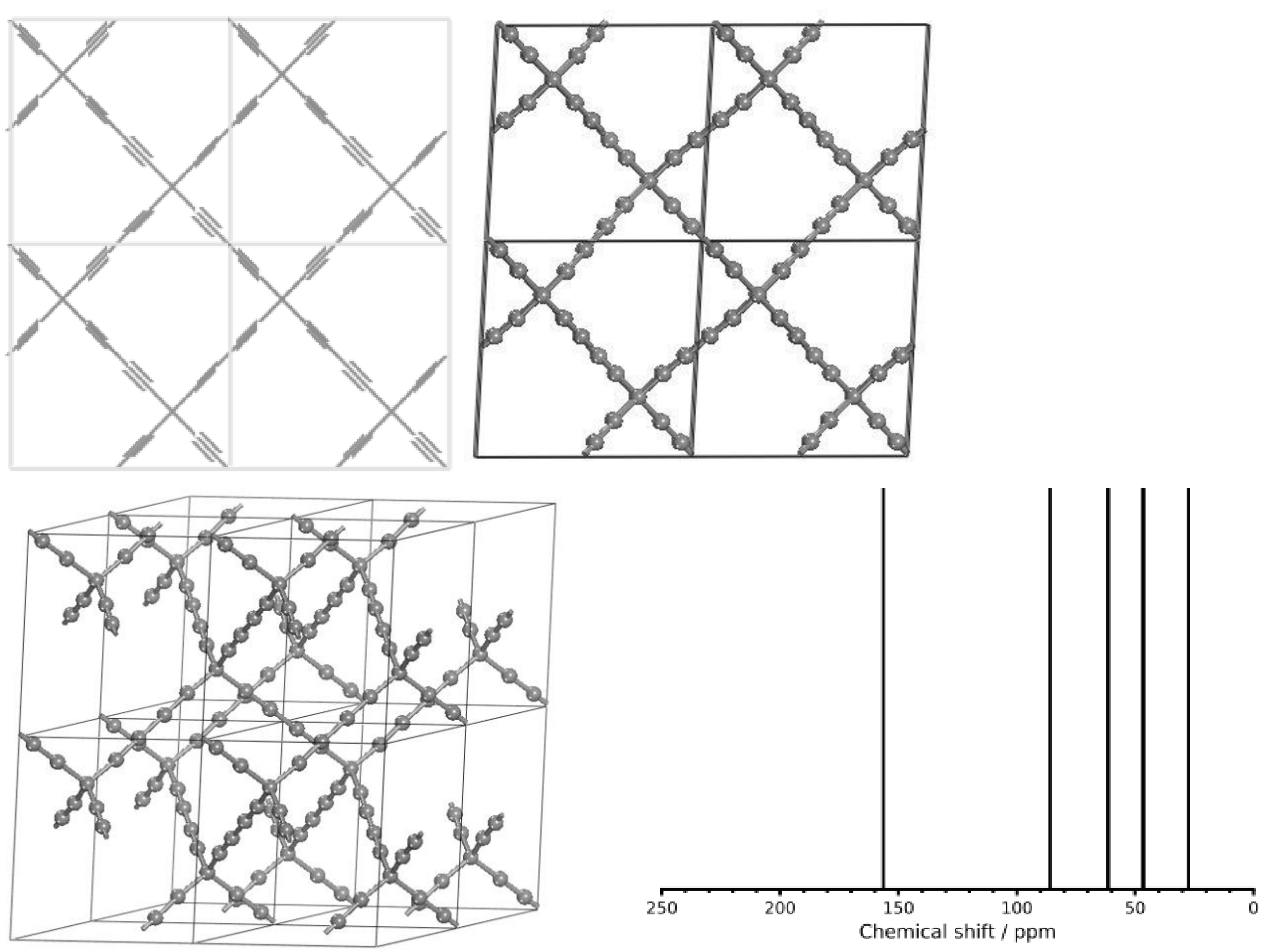

Figure SI.25: Structure and calculated NMR spectrum of OSPC-1 with an idealised *dia-c* topology.

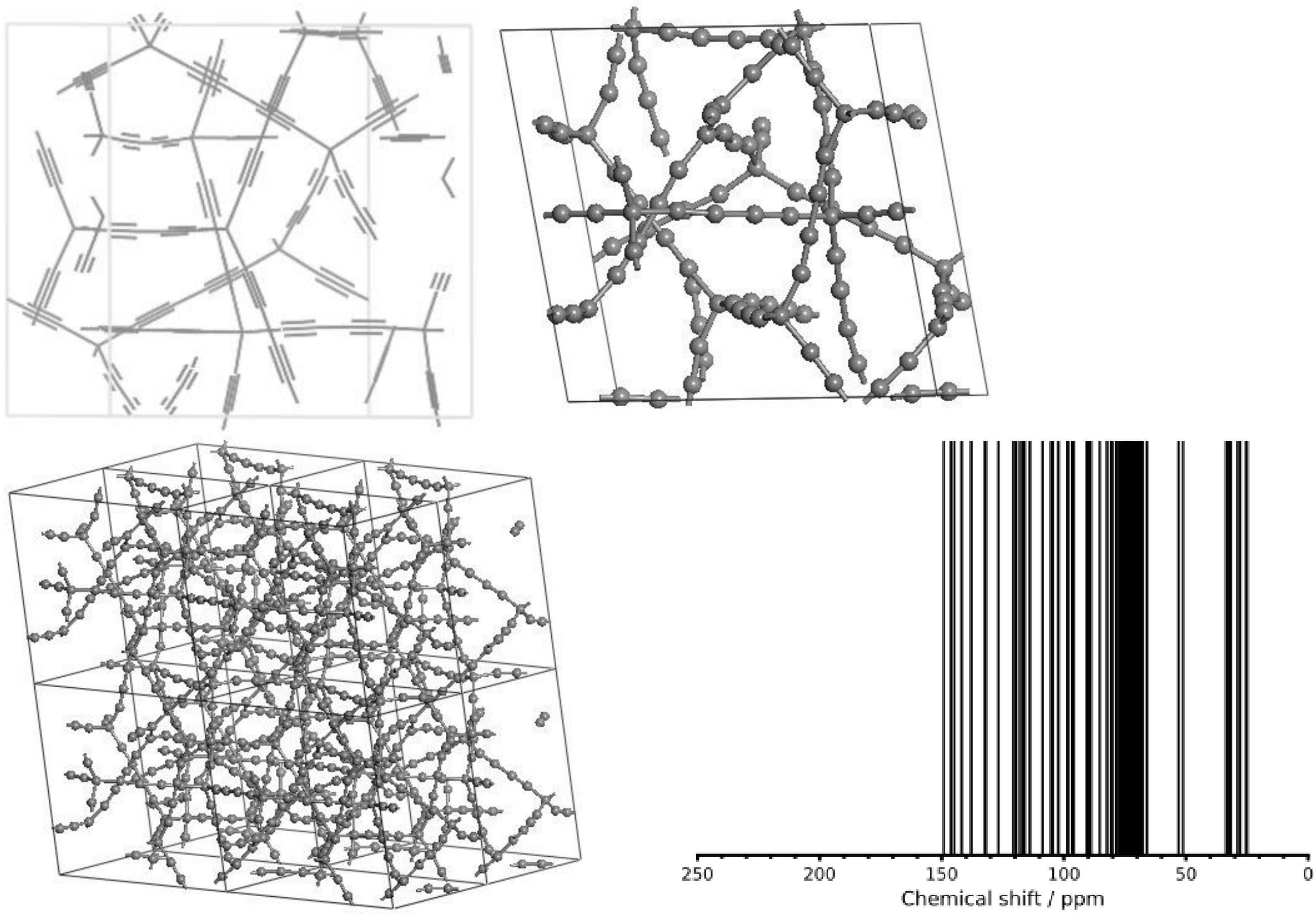

Figure SI.26: Structure and calculated NMR spectrum of OSPC-1 with an idealised *dia-c3* topology.

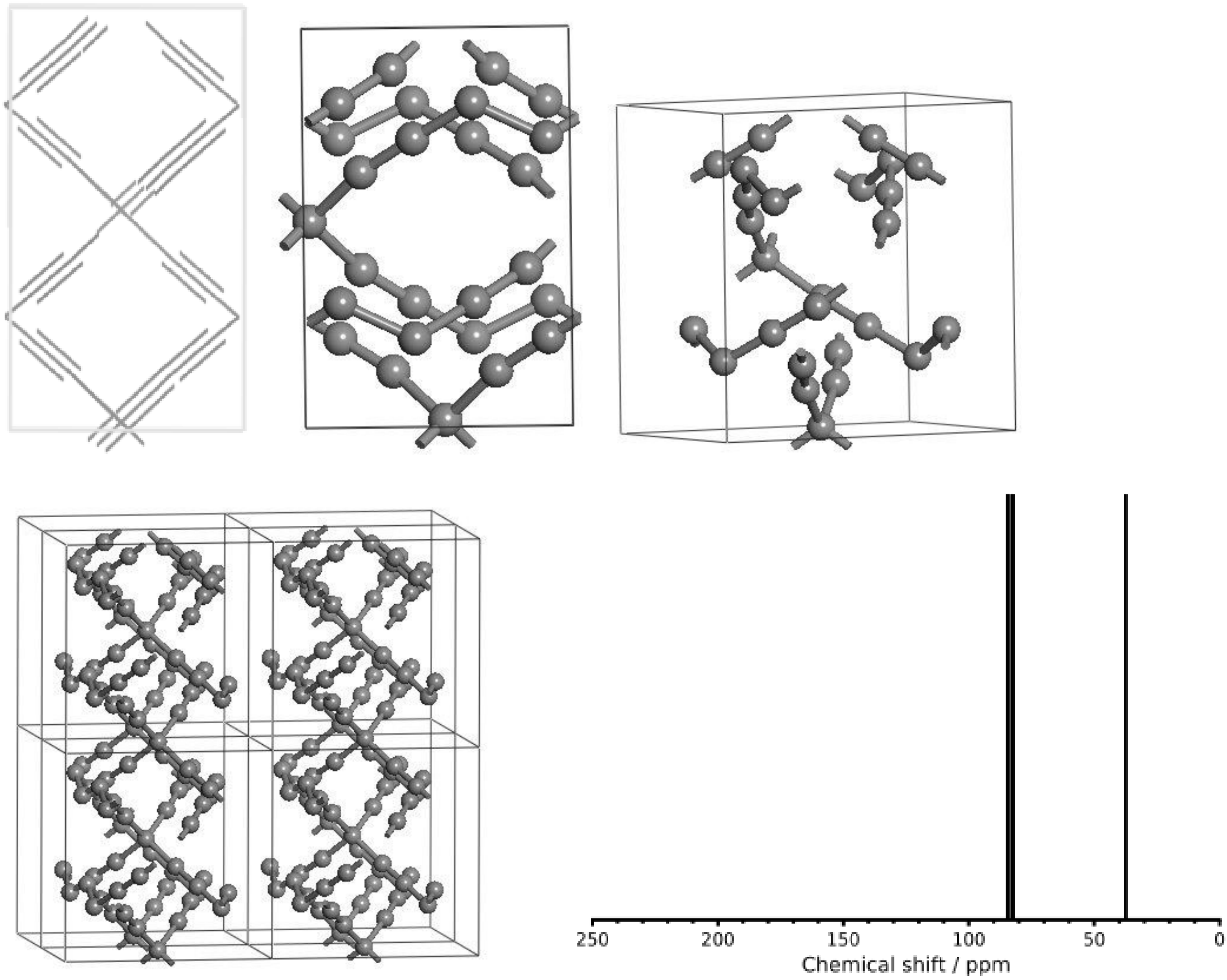

Figure SI.27: Structure and calculated NMR spectrum of OSPC-1 with an idealised *dia-c4* topology.

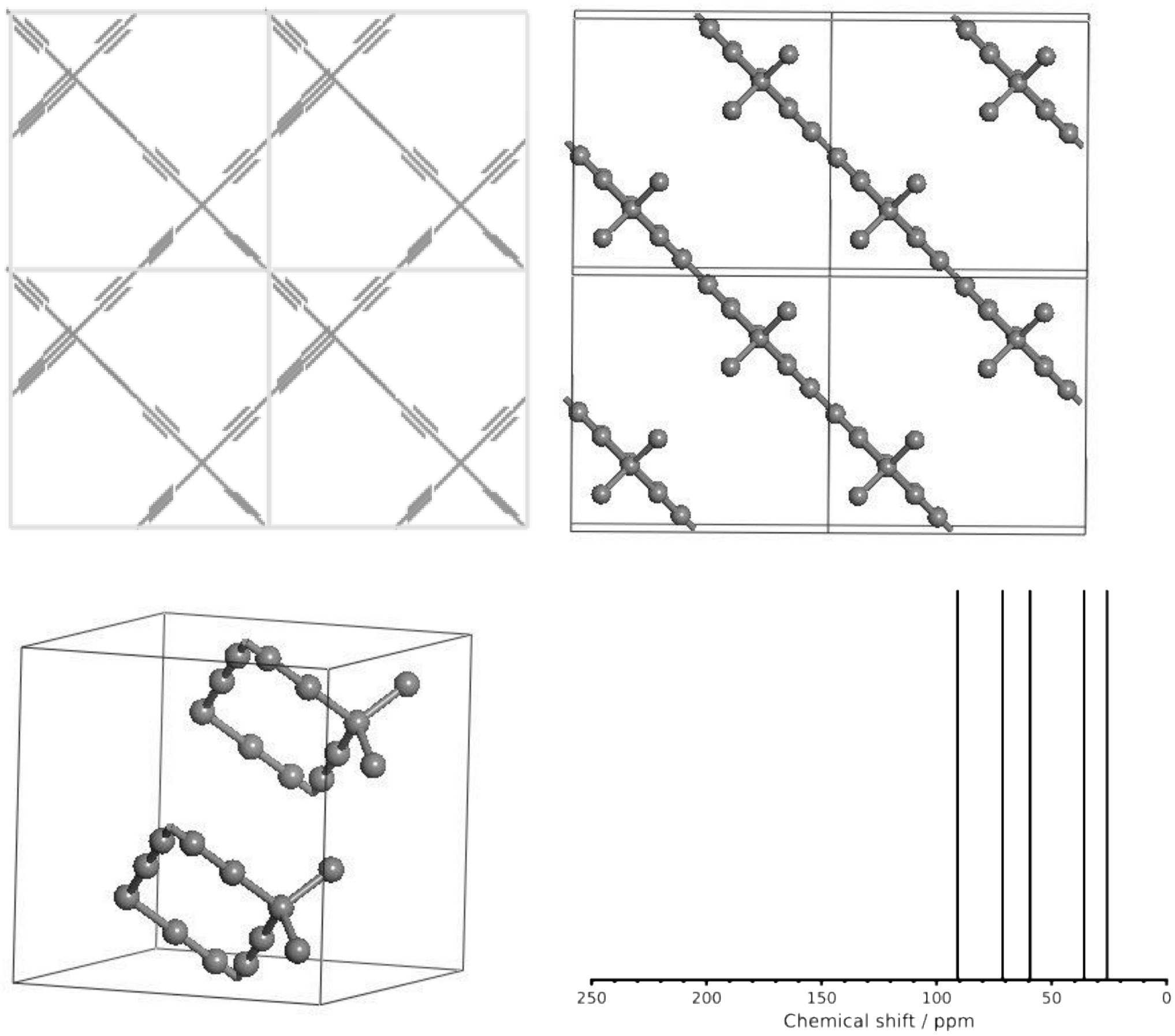

Figure SI.28: Structure and calculated NMR spectrum of OSPC-1 with an idealised *dia-c6* topology.

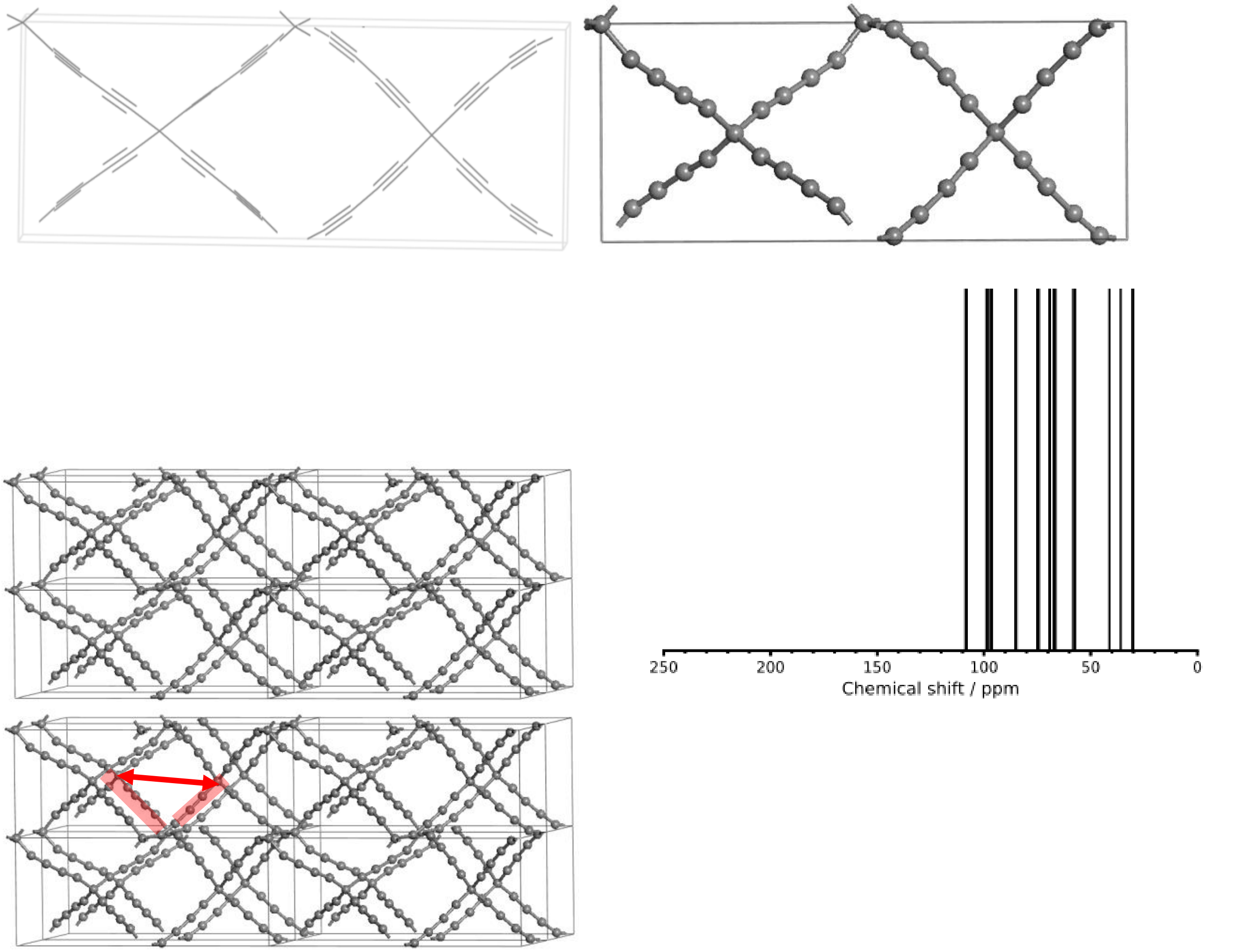

Figure SI.29: Structure and calculated NMR spectrum of OSPC-1 with an idealised *pts-c* topology. Red lines highlight the structural feature that we propose is present within the pore size distribution resulting in a peak at ~0.6 nm from the distance indicated by the red arrow.

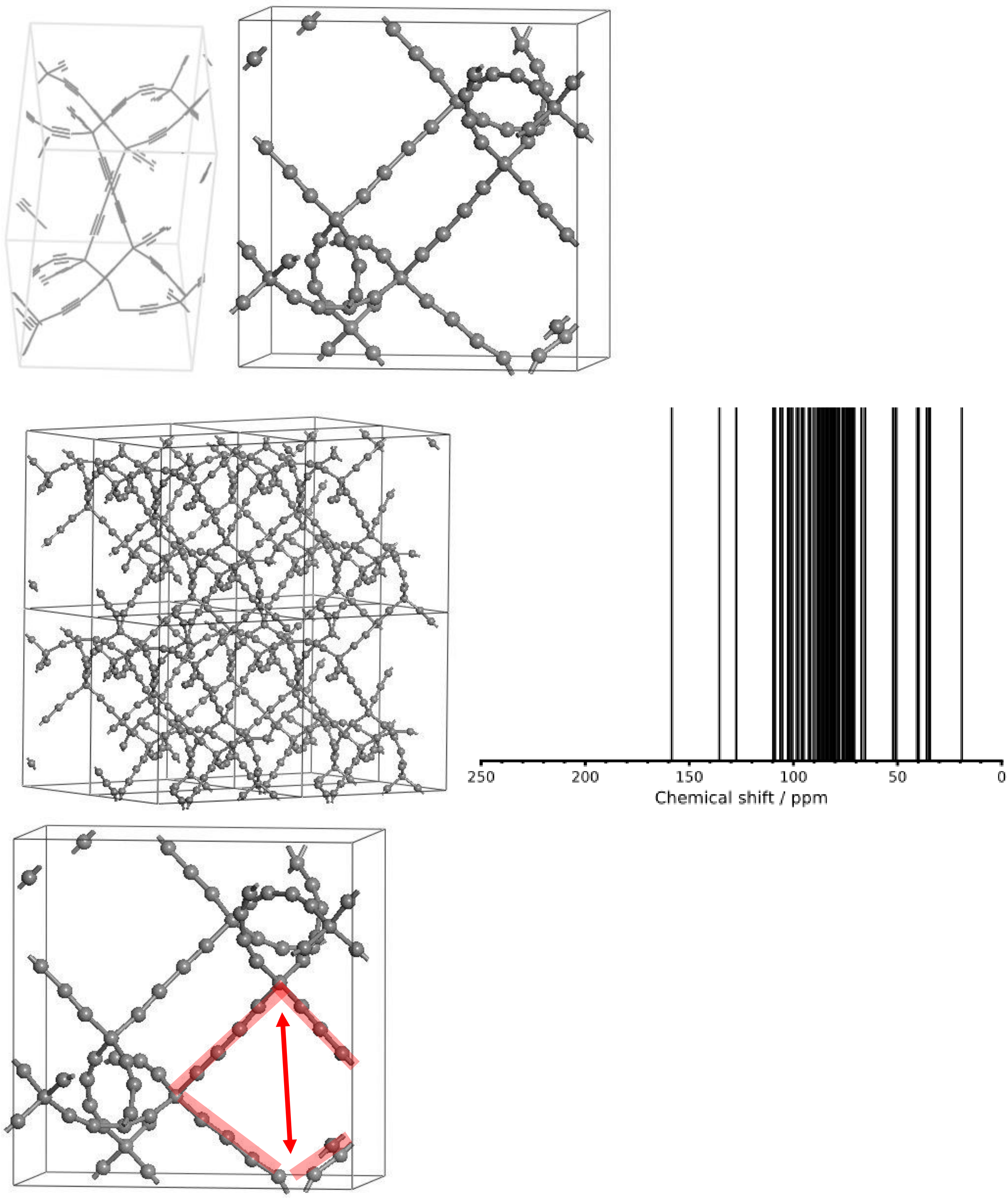

Figure SI.30: Structure and calculated NMR spectrum of OSPC-1 with an idealised *unc-c* topology. Red lines highlight the structural feature that we propose is present within the pore size distribution resulting in a peak at ~0.75 nm from the distance indicated by the red arrow.

Table SI.5: Solvent accessible surface areas and linker C-C distances of the reported OSPC-1 crystalline models given in Figure 4.

Model	Solvent accessible surface area / $m^2\ g^{-1}$	Closest linker C-C distance / Å
dia	6428	11.02
dia-c	0	5.69
pts-c	0	4.79
unc-c	1295	2.76

Section 6. OSPC-1 Chain-Chain Structural Models

The chain-chain structural periodic models were designed to investigate interactions between chains of OSPC-1 as the network interpenetrates. Different models were generated to investigate firstly the effect of changing just one dimension (to simulate either stacking the chains more closely next to each other or on top of one another by changing the y- or z-axis values, respectively, and then to see the effect of changing both the y- and z-axis values. The x-axis value was kept constant throughout, as this is the axis along which the chain lies, and the axis lengths in the y- and z-dimensions were changed in a stepwise manner, where a structure was optimised at one unit cell size, then the optimised structure was used as the input for the next smallest cell, to sequentially decrease the cell size. The structures in Set 12 (given in Table SI.17) were generated by placing two chains (lying along the x- and z-axes) at 90 degrees to one another and then decreasing the y-axis length in a stepwise manner.

Set 1. Table SI.6 y changes from 10.9 to 2.1 Å.
Set 2. Table SI.7 z changes from 10.9 to 5.5 Å.
Set 3. Table SI.8 y =3.4 z changes from 10.9 to 5.5 Å.
Set 4. Table SI.9 y =3.3 z changes from 10.9 to 6.0 Å.
Set 5. Table SI.10 y =3.2 z changes from 10.9 to 6.0 Å.
Set 6. Table SI.11 y =3.18 z changes from 10.9 to 6.0 Å.
Set 7. Table SI.12 y =3.16 z changes from 10.9 to 6.0 Å.
Set 8. Table SI.13 y =3.14 z changes from 10.9 to 6.0 Å.
Set 9. Table SI.14 y =3.12 z changes from 10.9 to 6.0 Å.
Set 10. Table SI.15 y =3.10 z changes from 10.9 to 6.0 Å.
Set 11. Table SI.16 y changes from 3.12 to 2.0, z = 6.0 Å.
Set 12. Table SI.17 chain in the x and z direction. y changes from 21.8392 to 6.443 Å.
Set 13. Table SI.18 OSPC-1 model (2x Br end groups) y and z change from 15.0 to 5.7 Å.
Set 14. Table SI.19 OSPC-1 model (1x Br, 1x TMS end groups) y changes from 15.0 to 5.5 Å and z changes from 20.0 to 9.5 Å.

OSPC-1 with ethynyl end groups

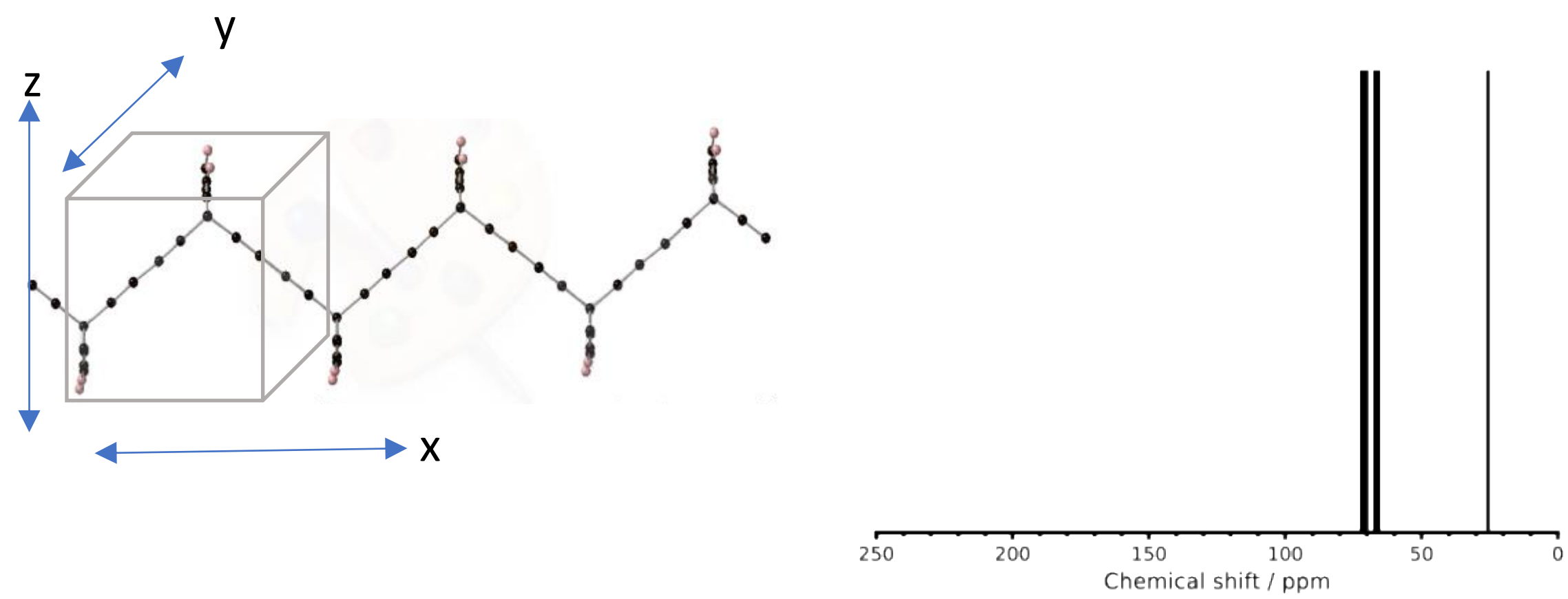

Figure SI.31: Structure and calculated NMR spectrum of an infinite in the x direction chain of OSPC-1 with periodic cell size of a=10.914 Å, b=10.914 Å, and c=10.914 Å. Model A in Table SI.4.

Model	Cell length (Å)		
	x	y	z
A	10.919594	10.919594	10.919594
B	10.919594	9.919594	10.919594
C	10.919594	8.919594	10.919594
D	10.919594	7.919594	10.919594
E	10.919594	6.919594	10.919594
F	10.919594	5.919594	10.919594
FF	10.919594	5.500000	10.919594
FG	10.919594	4.919594	10.919594
FH	10.919594	4.500000	10.919594
FI	10.919594	3.919594	10.919594
FJ	10.919594	3.500000	10.919594
FM	10.919594	3.400000	10.919594
FN	10.919594	3.300000	10.919594
FO	10.919594	3.200000	10.919594
FR	10.919594	3.190000	10.919594
FS	10.919594	3.180000	10.919594
FT	10.919594	3.170000	10.919594
FU	10.919594	3.160000	10.919594
FV	10.919594	3.150000	10.919594
FW	10.919594	3.140000	10.919594
FX	10.919594	3.130000	10.919594
FY	10.919594	3.120000	10.919594
FZ	10.919594	3.110000	10.919594
FP	10.919594	3.100000	10.919594
FQ	10.919594	3.000000	10.919594
FK	10.919594	2.919594	10.919594
PY	10.919594	2.700000	10.919594
QT	10.919594	2.650000	10.919594
QU	10.919594	2.600000	10.919594
QV	10.919594	2.550000	10.919594
FL	10.919594	2.500000	10.919594
QW	10.919594	2.450000	10.919594
QX	10.919594	2.400000	10.919594
QY	10.919594	2.350000	10.919594
PZ	10.919594	2.300000	10.919594
QZ	10.919594	2.250000	10.919594
RA	10.919594	2.200000	10.919594
RB	10.919594	2.150000	10.919594
QA	10.919594	2.100000	10.919594

Table SI.6: Set 1

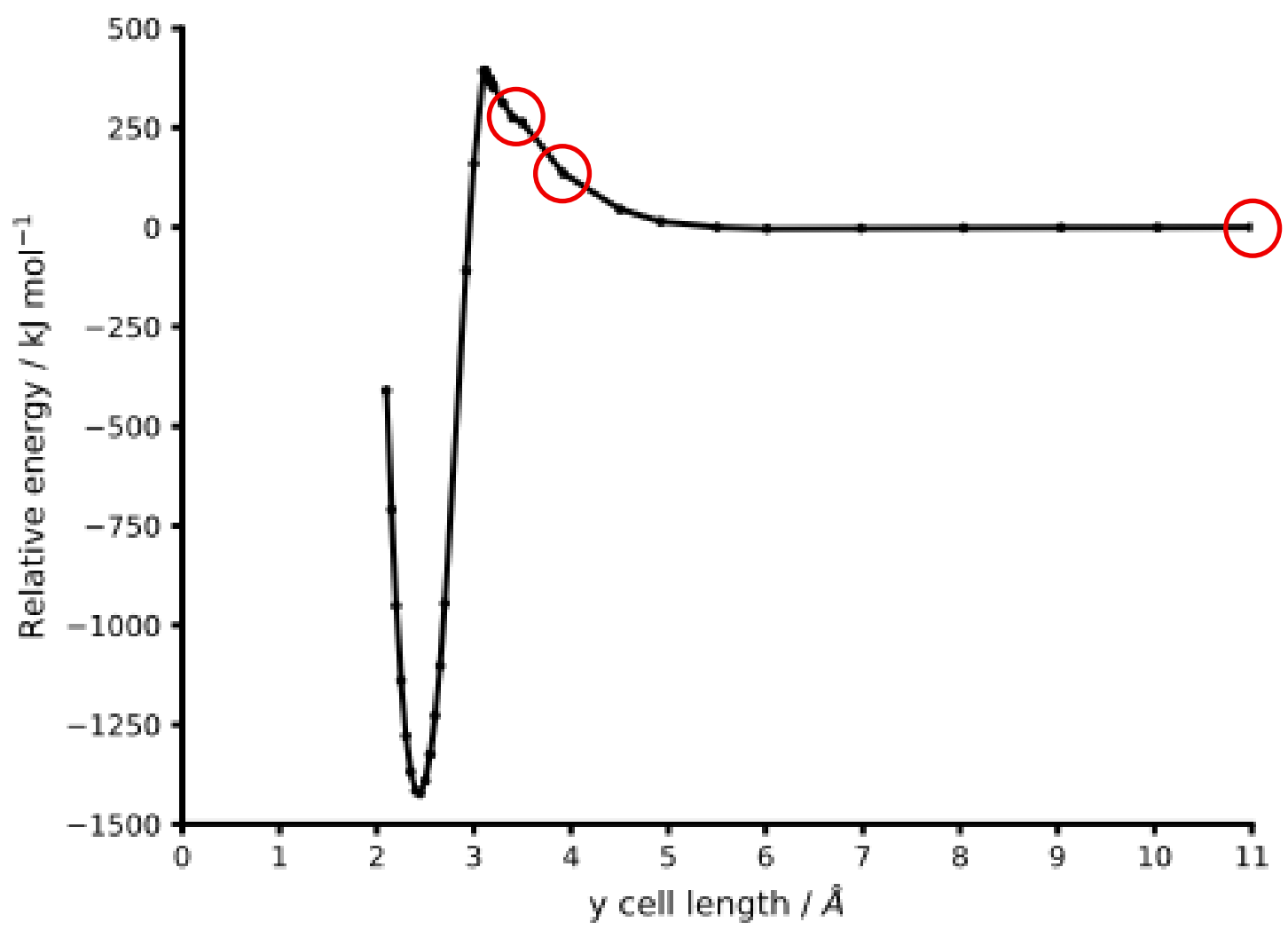

Figure SI.32: Energy of the optimised chain structure models from Set 1 where the y-cell length changes. Energies are relative to model A.

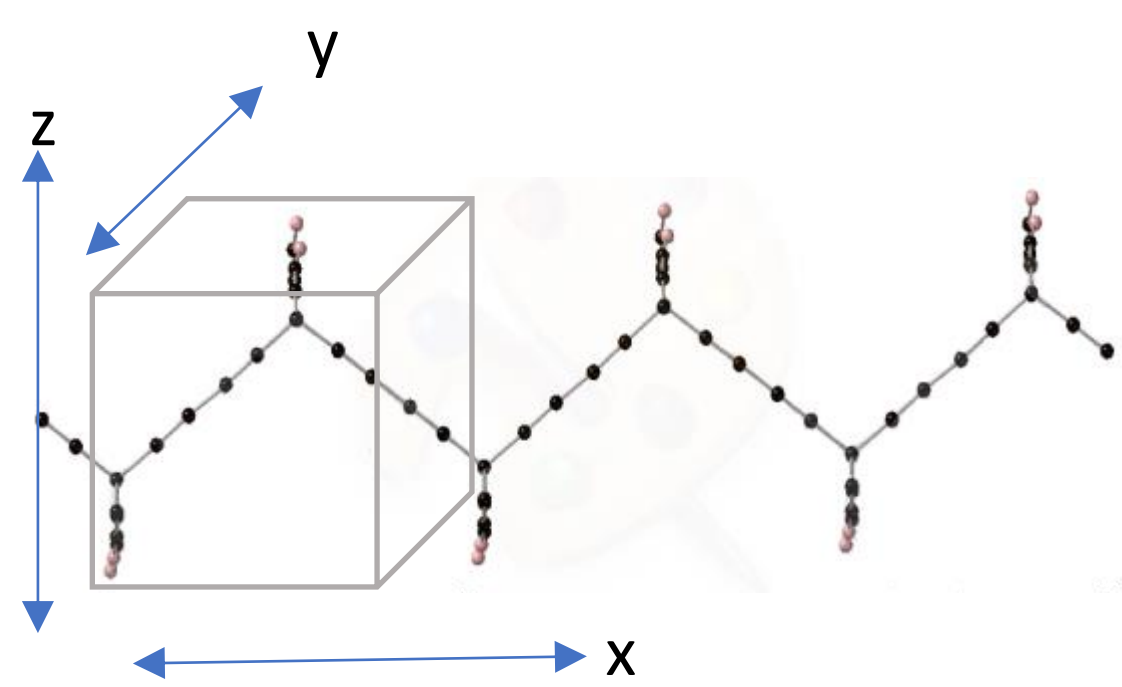

Model	Cell length (Å)		
	x	y	z
FJ	10.919594	3.500000	10.919594
IL	10.919594	3.500000	10.500000
IM	10.919594	3.500000	9.919594
IN	10.919594	3.500000	9.500000
IO	10.919594	3.500000	8.919594
IP	10.919594	3.500000	8.500000
IQ	10.919594	3.500000	7.919594
IR	10.919594	3.500000	7.500000
IS	10.919594	3.500000	6.919594
IT	10.919594	3.500000	6.500000
IU	10.919594	3.500000	6.000000
IV	10.919594	3.500000	5.500000

Table SI.7: Set 2

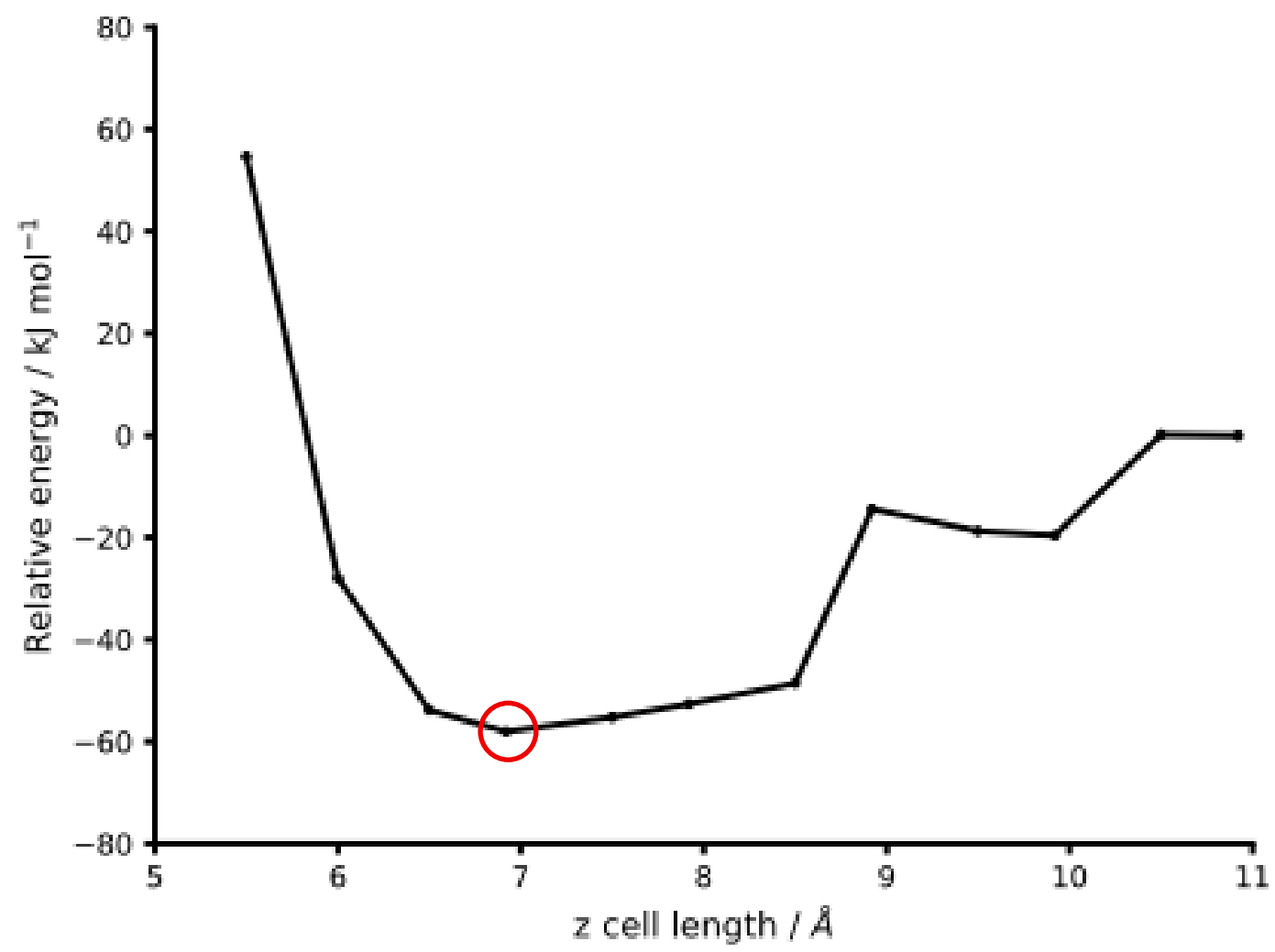

Figure SI.33: Energy of the optimised chain structure models from Set 2 where the z-cell length changes. Energies are relative to model FJ.

Model	Cell length (Å)		
	x	y	z
FM	10.919594	3.400000	10.919594
JE	10.919594	3.400000	10.500000
JF	10.919594	3.400000	9.919594
JG	10.919594	3.400000	9.500000
JH	10.919594	3.400000	8.919594
JI	10.919594	3.400000	8.500000
JJ	10.919594	3.400000	7.919594
JK	10.919594	3.400000	7.500000
JL	10.919594	3.400000	6.919594
JM	10.919594	3.400000	6.500000
JN	10.919594	3.400000	6.000000
JO	10.919594	3.400000	5.500000

Table SI.8: Set 3

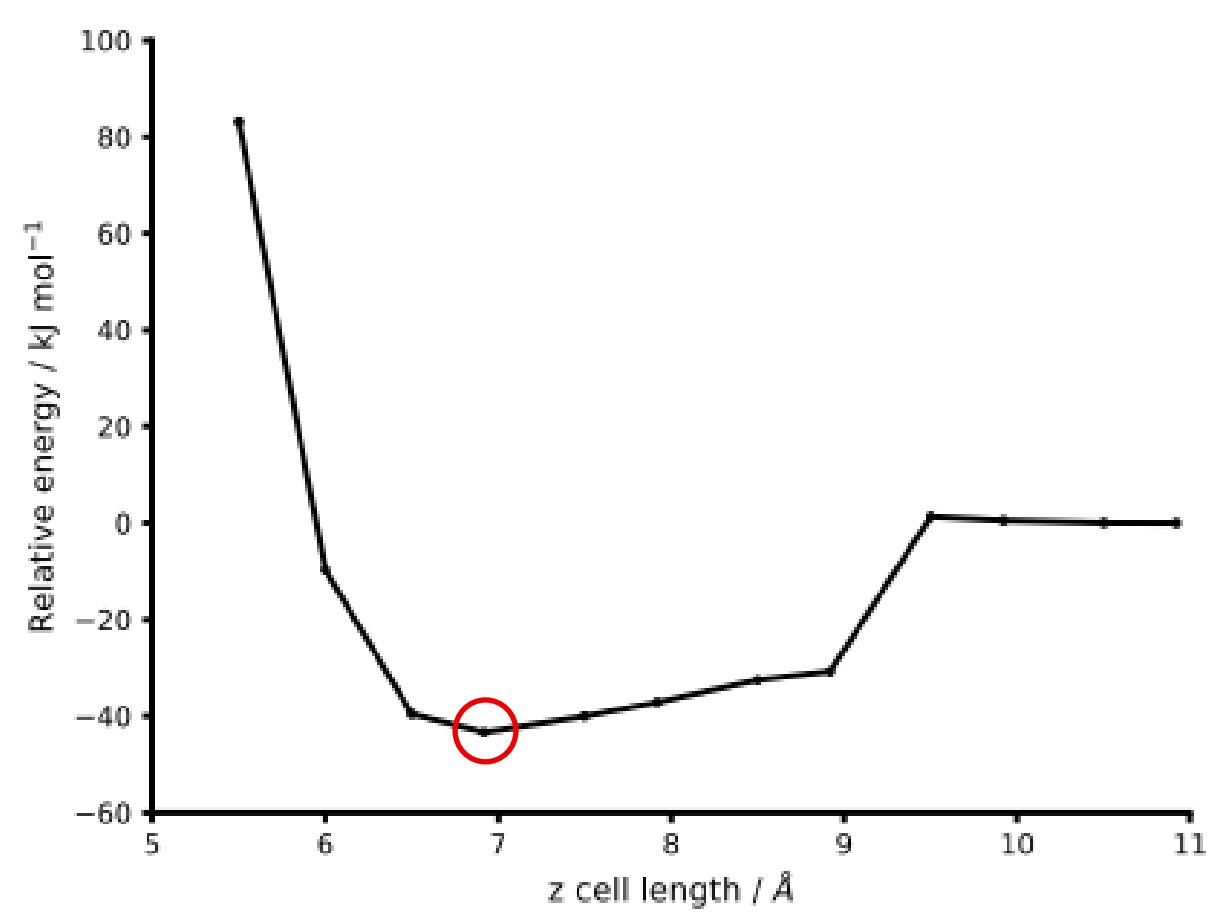

Figure SI.34: Energy of the optimised chain structure models from Set 3 where the z-cell length changes. Energies are relative to model FM.

Model	Cell length (Å)		
	x	y	z
FN	10.919594	3.300000	10.919594
JQ	10.919594	3.300000	10.500000
JR	10.919594	3.300000	9.919594
JS	10.919594	3.300000	9.500000
JT	10.919594	3.300000	8.919594
JU	10.919594	3.300000	8.500000
JV	10.919594	3.300000	7.919594
JW	10.919594	3.300000	7.500000
JX	10.919594	3.300000	6.919594
JY	10.919594	3.300000	6.500000
JZ	10.919594	3.300000	6.000000

Table SI.9: Set 4

Model	Cell length (Å)		
	x	y	z
FO	10.919594	3.200000	10.919594
KC	10.919594	3.200000	10.500000
KD	10.919594	3.200000	9.919594
KE	10.919594	3.200000	9.500000
KF	10.919594	3.200000	8.919594
KG	10.919594	3.200000	8.500000
KH	10.919594	3.200000	7.919594
KI	10.919594	3.200000	7.500000
KJ	10.919594	3.200000	6.919594
KK	10.919594	3.200000	6.500000
KL	10.919594	3.200000	6.000000

Table SI.10: Set 5

Model	Cell length (Å)		
	x	y	z
FS	10.919594	3.180000	10.919594
KO	10.919594	3.180000	10.500000
KP	10.919594	3.180000	9.919594
KQ	10.919594	3.180000	9.500000
KR	10.919594	3.180000	8.919594
KS	10.919594	3.180000	8.500000
KT	10.919594	3.180000	7.919594
KU	10.919594	3.180000	7.500000
KV	10.919594	3.180000	6.919594
KW	10.919594	3.180000	6.500000
KX	10.919594	3.180000	6.000000

Table SI.11: Set 6

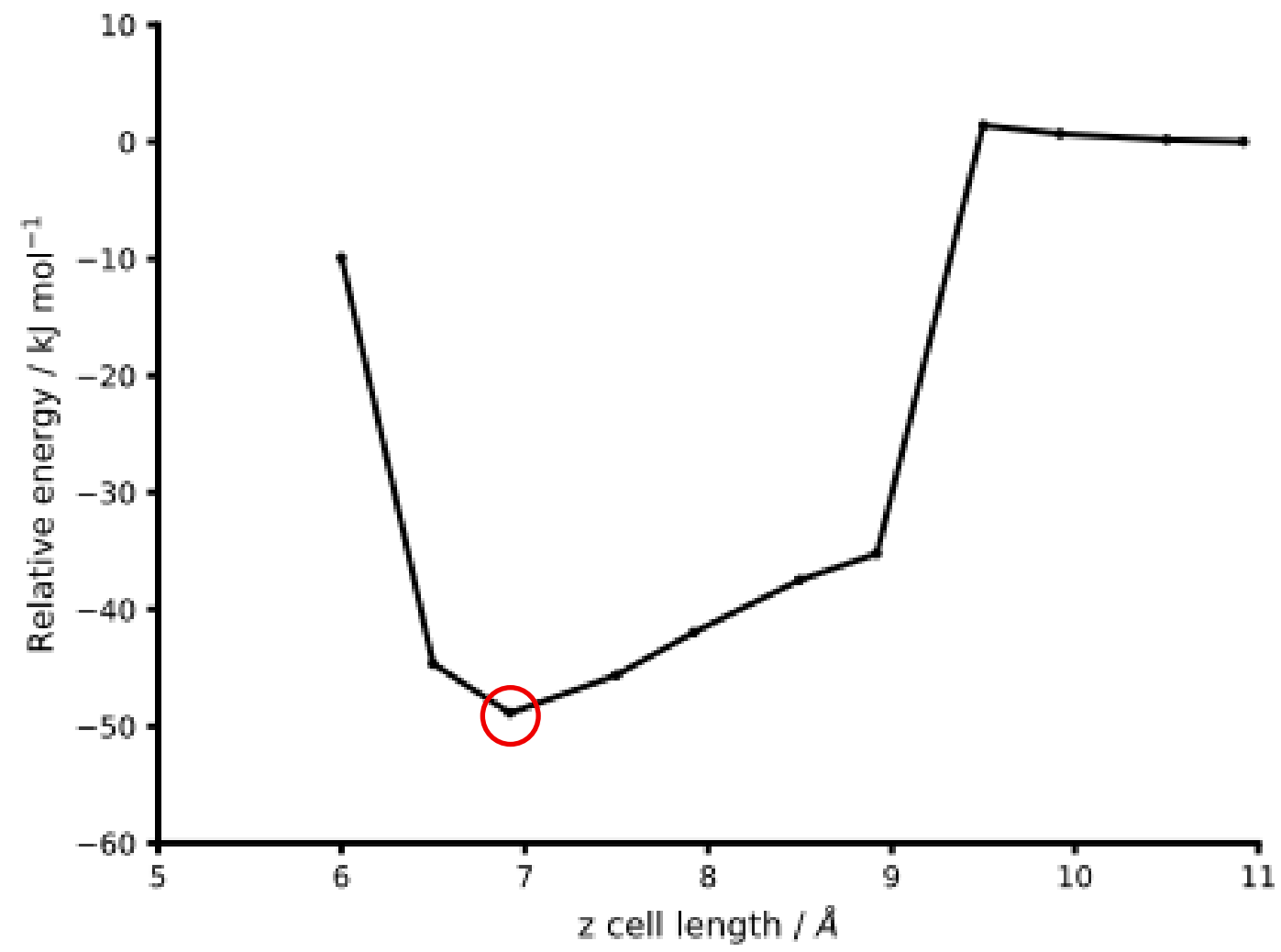

Figure SI.35: Energy of the optimised chain structure models from Set 4 where the z-cell length changes. Energies are relative to model FN.

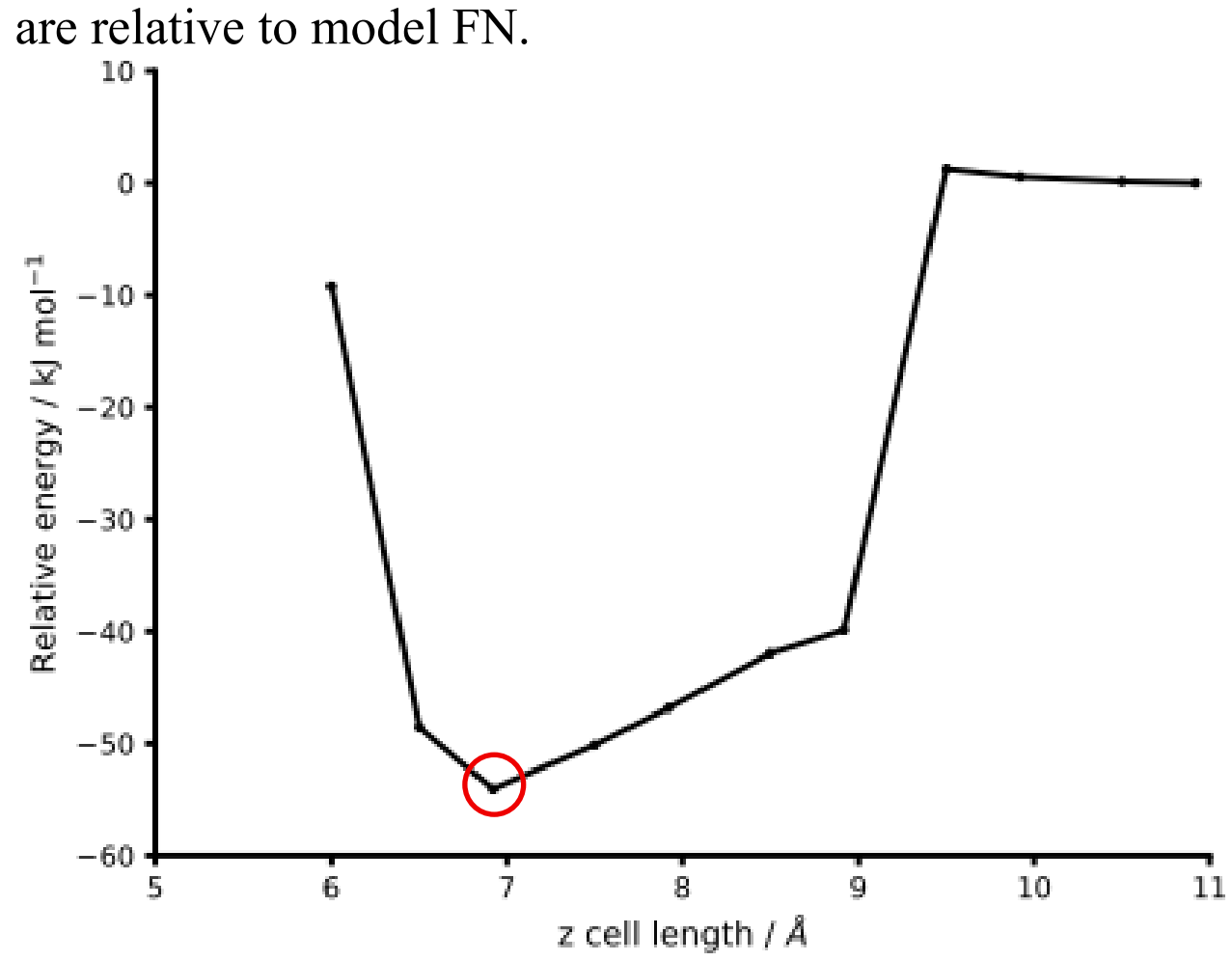

Figure SI.36: Energy of the optimised chain structure models from Set 5 where the z-cell length changes. Energies are relative to model FO.

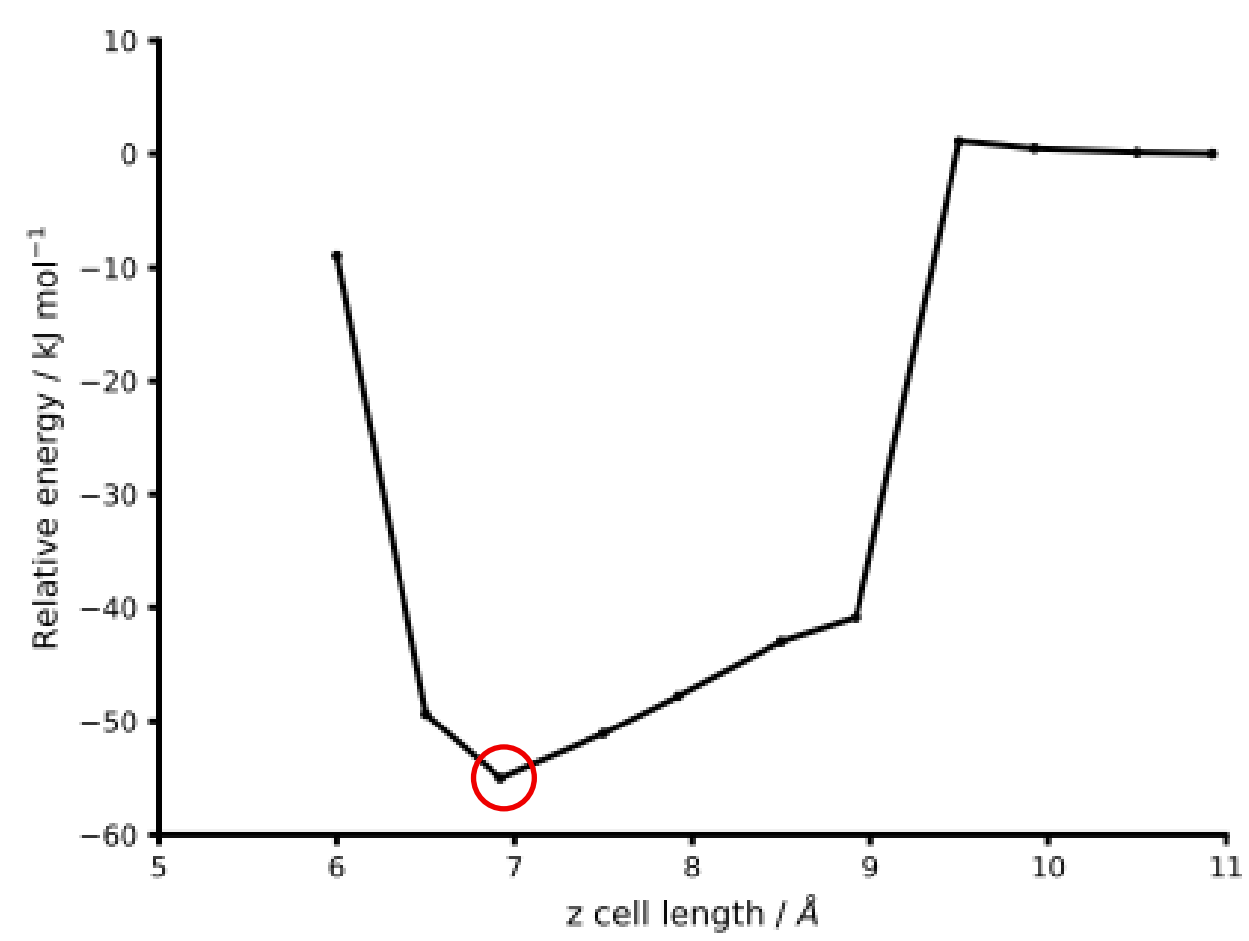

Figure SI.37: Energy of the optimised chain structure models from Set 6 where the z-cell length changes. Energies are relative to model FS.

Model	Cell length (Å)		
	x	y	z
FU	10.919594	3.160000	10.919594
LA	10.919594	3.160000	10.500000
LB	10.919594	3.160000	9.919594
LC	10.919594	3.160000	9.500000
LD	10.919594	3.160000	8.919594
LE	10.919594	3.160000	8.500000
LF	10.919594	3.160000	7.919594
LG	10.919594	3.160000	7.500000
LH	10.919594	3.160000	6.919594
LI	10.919594	3.160000	6.500000
LJ	10.919594	3.160000	6.000000

Table SI.12: Set 7

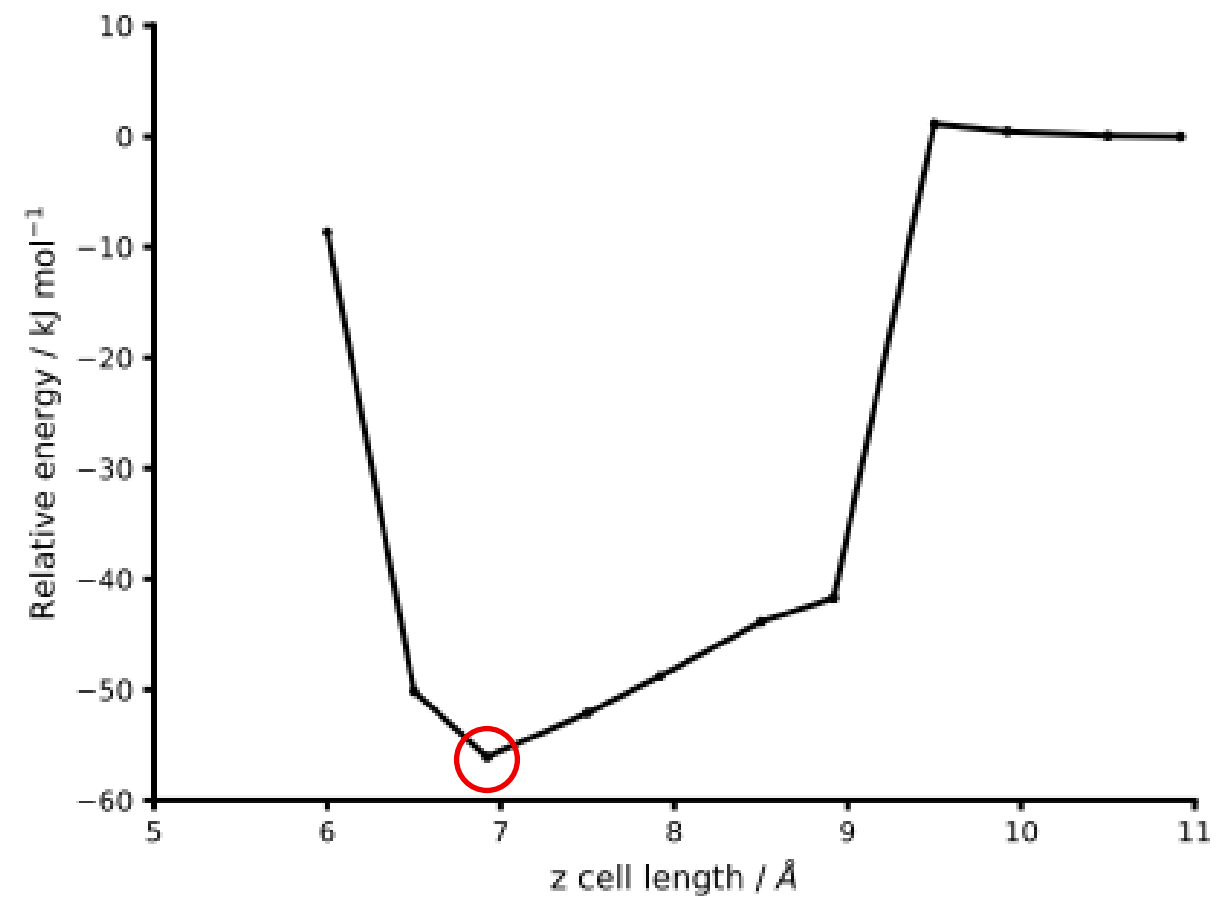

Figure SI.38: Energy of the optimised chain structure models from Set 7 where the z-cell length changes. Energies are relative to model FU.

Model	Cell length (Å)		
	x	y	z
FW	10.919594	3.140000	10.919594
LM	10.919594	3.140000	10.500000
LN	10.919594	3.140000	9.919594
LO	10.919594	3.140000	9.500000
LP	10.919594	3.140000	8.919594
LQ	10.919594	3.140000	8.500000
LR	10.919594	3.140000	7.919594
LS	10.919594	3.140000	7.500000
LT	10.919594	3.140000	6.919594
LU	10.919594	3.140000	6.500000
LV	10.919594	3.140000	6.000000

Table SI.13: Set 8

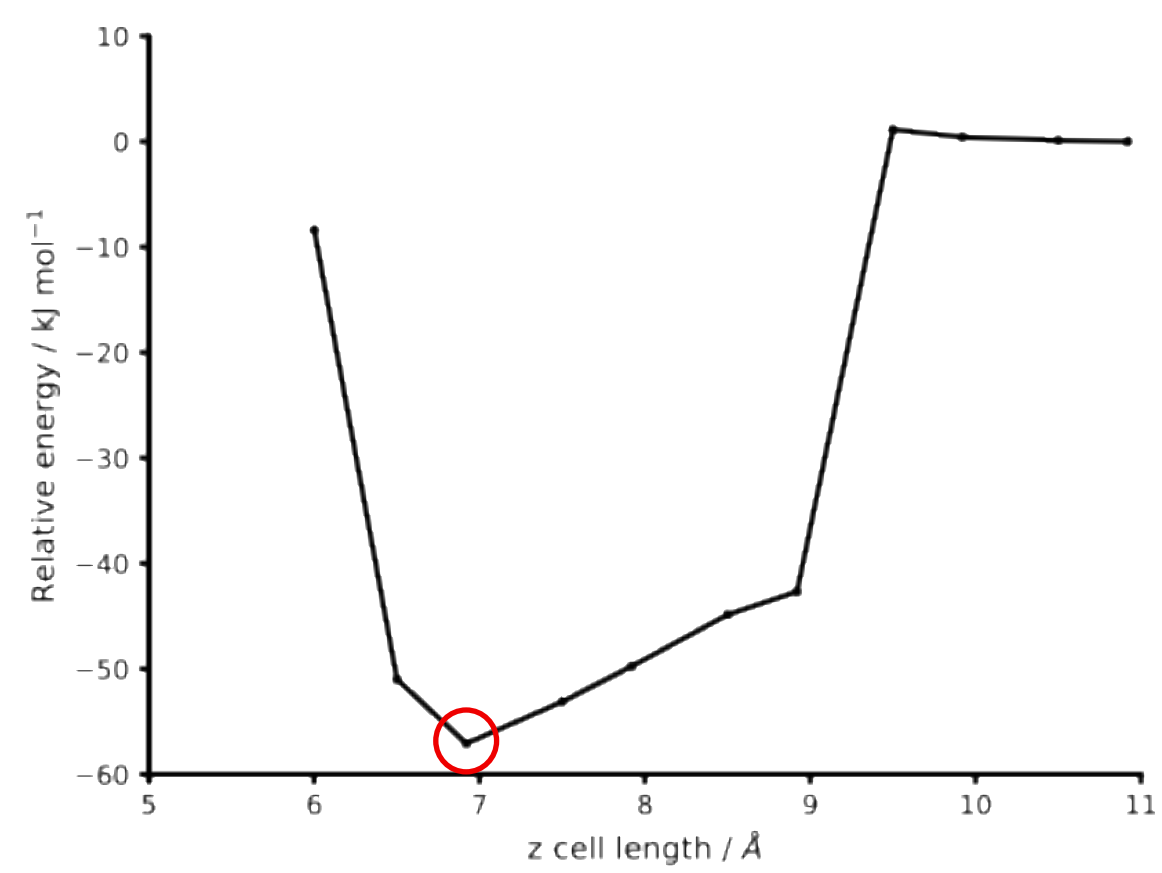

Figure SI.39: Energy of the optimised chain structure models from Set 8 where the z-cell length changes. Energies are relative to model FW.

Model	Cell length (Å)		
	x	y	z
FY	10.919594	3.120000	10.919594
LY	10.919594	3.120000	10.500000
LZ	10.919594	3.120000	9.919594
MA	10.919594	3.120000	9.500000
MB	10.919594	3.120000	8.919594
MC	10.919594	3.120000	8.500000
MD	10.919594	3.120000	7.919594
ME	10.919594	3.120000	7.500000
MF	10.919594	3.120000	6.919594
MG	10.919594	3.120000	6.500000
MH	10.919594	3.120000	6.000000

Table SI.14: Set 9

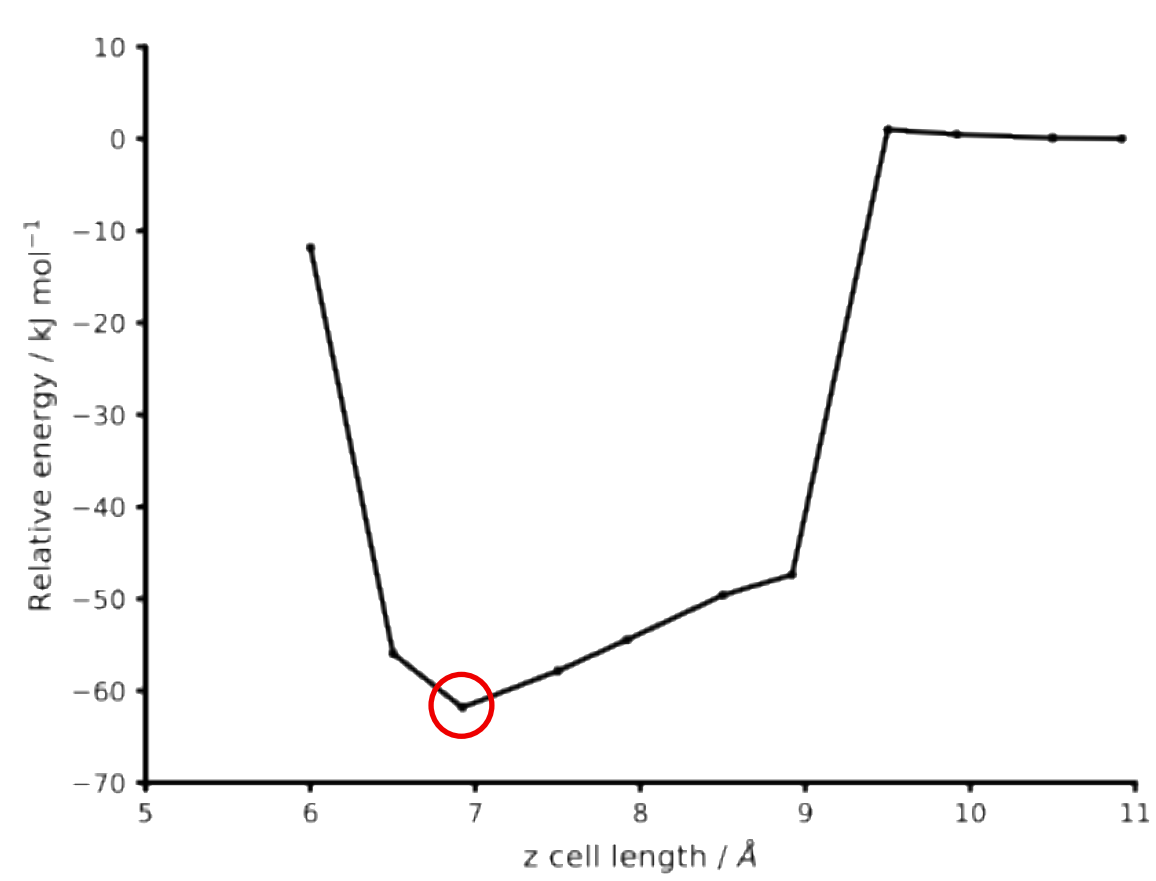

Figure SI.40: Energy of the optimised chain structure models from Set 9 where the z-cell length changes. Energies are relative to model FY.

Model	Cell length (Å)		
	x	y	z
FP	10.919594	3.100000	10.919594
MK	10.919594	3.100000	10.500000
ML	10.919594	3.100000	9.919594
MM	10.919594	3.100000	9.500000
MN	10.919594	3.100000	8.919594
MO	10.919594	3.100000	8.500000
MP	10.919594	3.100000	7.919594
MQ	10.919594	3.100000	7.500000
MR	10.919594	3.100000	6.919594
RQ	10.919594	3.100000	6.900000
RR	10.919594	3.100000	6.800000
RS	10.919594	3.100000	6.700000
RT	10.919594	3.100000	6.600000
MS	10.919594	3.100000	6.500000
RU	10.919594	3.100000	6.400000
RV	10.919594	3.100000	6.300000
RW	10.919594	3.100000	6.200000
RX	10.919594	3.100000	6.100000
MT	10.919594	3.100000	6.000000

Table SI.15: Set 10

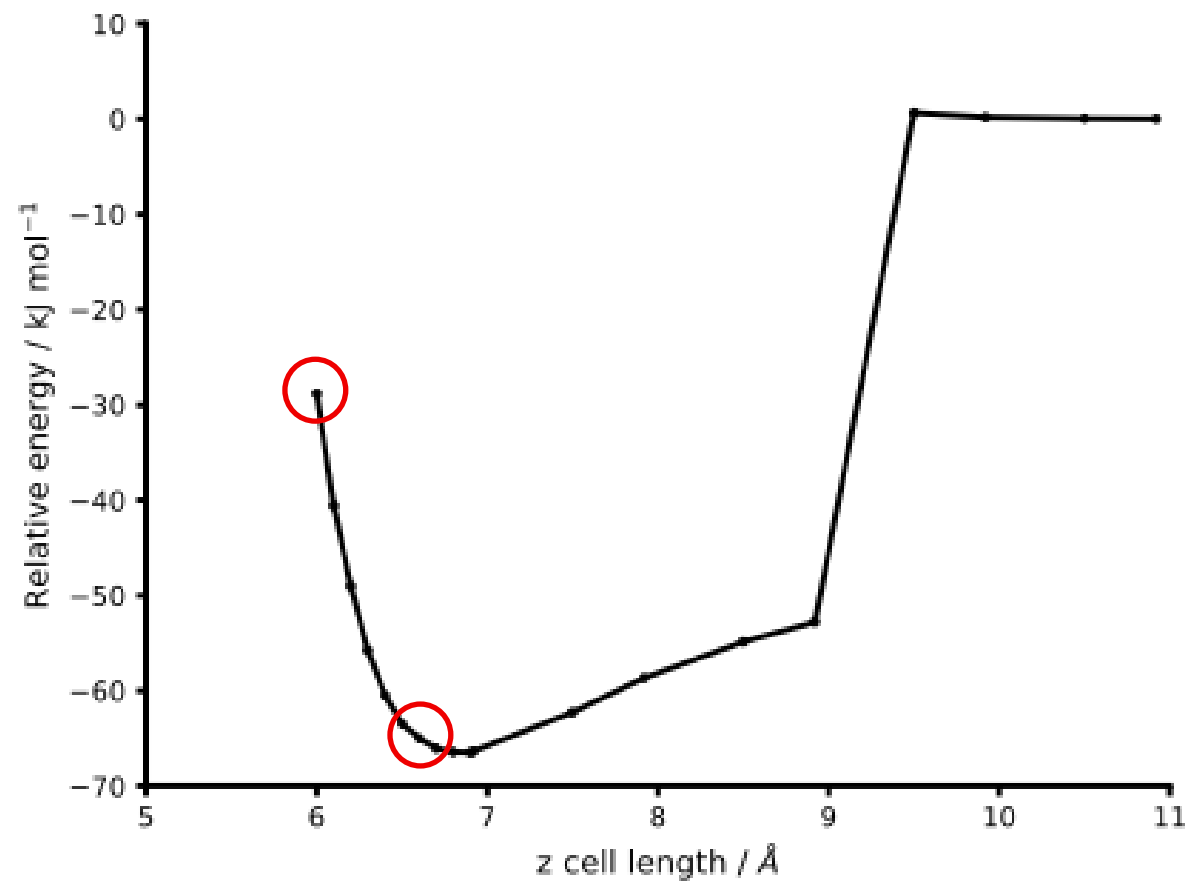

Figure SI.41: Energy of the optimised chain structure models from Set 10 where the z-cell length changes. Energies are relative to model FP.

Model	Cell length (Å)		
	x	y	z
MH	10.919594	3.120000	6.000000
PP	10.919594	3.116000	6.000000
PQ	10.919594	3.112000	6.000000
PR	10.919594	3.108000	6.000000
PS	10.919594	3.104000	6.000000
MT	10.919594	3.100000	6.000000
QB	10.919594	3.000000	6.000000
QC	10.919594	2.900000	6.000000
QD	10.919594	2.800000	6.000000
QE	10.919594	2.700000	6.000000
QF	10.919594	2.600000	6.000000
QG	10.919594	2.500000	6.000000
QO	10.919594	2.400000	6.000000
QP	10.919594	2.300000	6.000000
QQ	10.919594	2.200000	6.000000
QR	10.919594	2.100000	6.000000
QS	10.919594	2.000000	6.000000

Table SI.16: Set 11

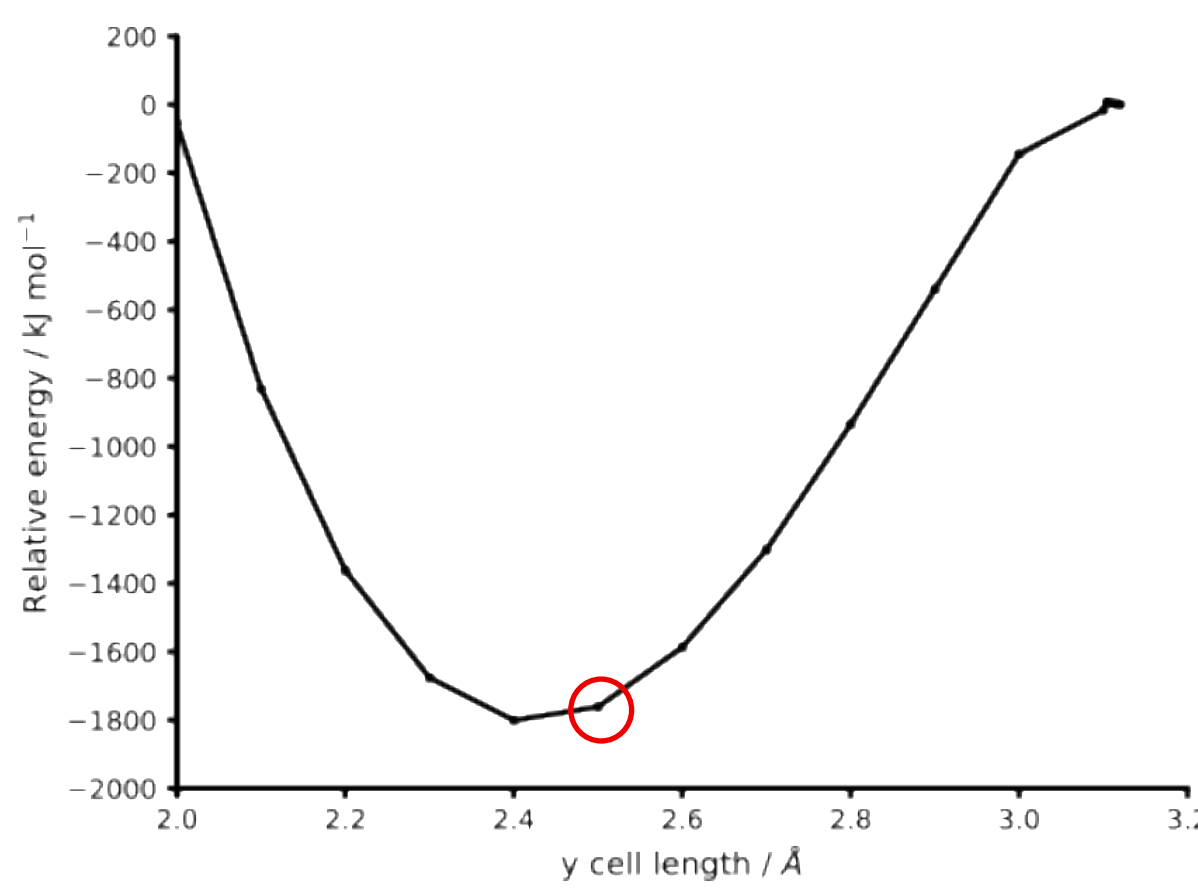

Figure SI.42: Energy of the optimised chain structure models from Set 11 where the y-cell length changes. Energies are relative to model MH.

Chain in the x and z direction

Model	Cell length (Å)		
	x	y	z
NM	21.8392	21.8392	21.8392
NN	21.8392	21.4196	21.8392
NO	21.8392	20.8392	21.8392
NP	21.8392	20.4196	21.8392
NQ	21.8392	19.8392	21.8392
NR	21.8392	19.4196	21.8392
NS	21.8392	18.8392	21.8392
NT	21.8392	18.4196	21.8392
NU	21.8392	17.8392	21.8392
NV	21.8392	17.4196	21.8392
NW	21.8392	16.8392	21.8392
NX	21.8392	16.4196	21.8392
NY	21.8392	15.8392	21.8392
NZ	21.8392	15.4196	21.8392
OA	21.8392	14.8392	21.8392
OB	21.8392	14.4196	21.8392
OC	21.8392	13.8392	21.8392
OD	21.8392	13.4196	21.8392
OE	21.8392	12.8392	21.8392
OF	21.8392	12.4196	21.8392
OG	21.8392	11.8392	21.8392
OH	21.8392	11.4196	21.8392
OI	21.8392	10.8392	21.8392
OJ	21.8392	10.4196	21.8392
OR	21.8392	9.8392	21.8392
OS	21.8392	9.4196	21.8392
OT	21.8392	8.8392	21.8392
OU	21.8392	8.4196	21.8392
OV	21.8392	7.8392	21.8392
OW	21.8392	7.4196	21.8392
OX	21.8392	6.8392	21.8392
OY	21.8392	6.4196	21.8392
OZ	21.8392	5.8392	21.8392
PA	21.8392	5.4196	21.8392
PB	21.8392	4.8392	21.8392
PD	21.8392	4.7343	21.8392
PF	21.8392	4.7243	21.8392

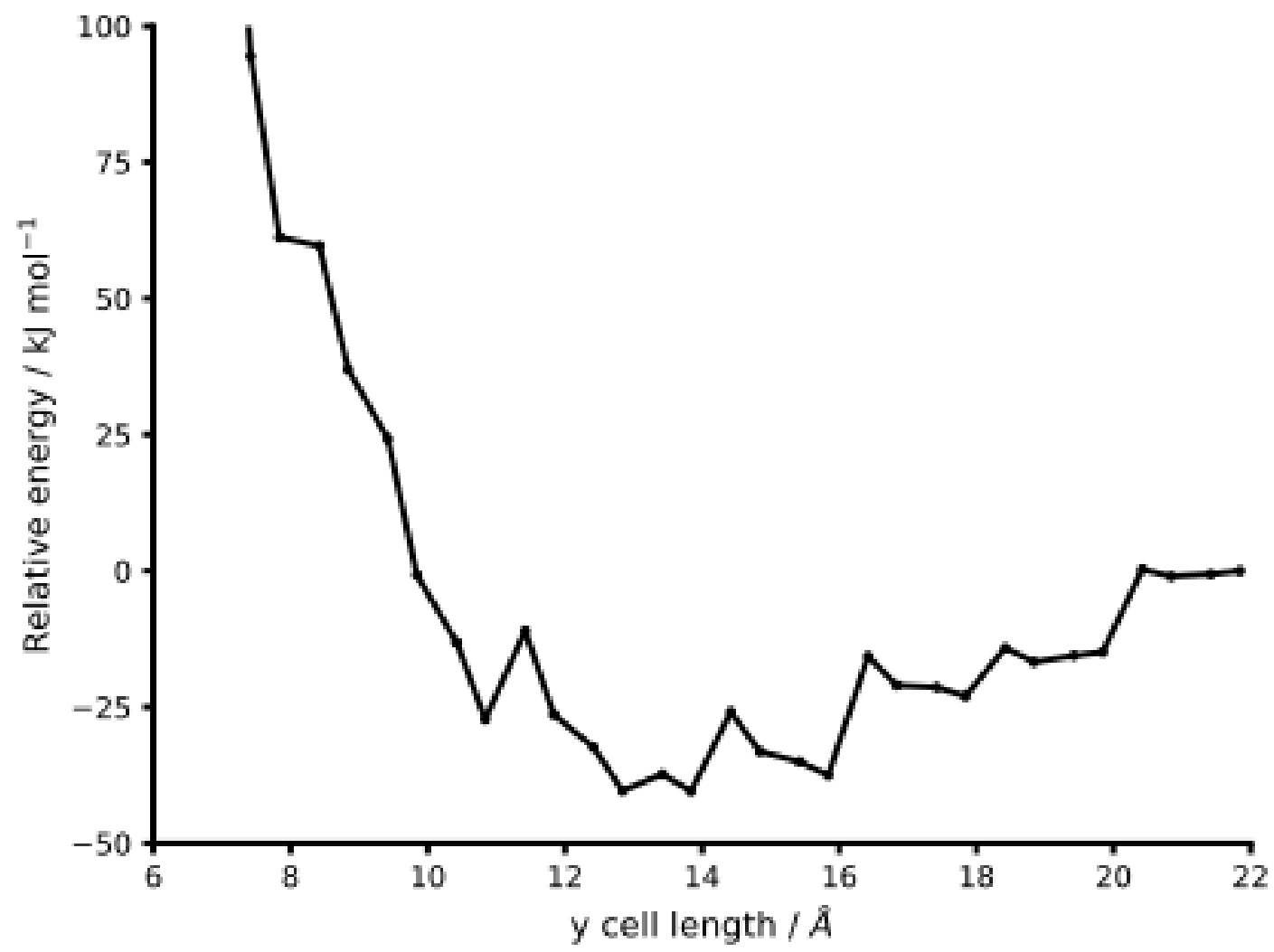

Figure SI.43: Energy of the optimised chain structure model with chains in the x and z directions from Set 12 where the y-cell length changes. Energies are relative to model NM.

PG	21.8392	4.7143	21.8392
PH	21.8392	4.7043	21.8392
PI	21.8392	4.6943	21.8392
PJ	21.8392	4.6843	21.8392
PK	21.8392	4.6743	21.8392
PL	21.8392	4.6643	21.8392
PM	21.8392	4.6543	21.8392
PN	21.8392	4.6443	21.8392

Table SI.17: Set 12. Chains in the x and z directions at 90 degrees.

Section 7. OSPC-1 Chain-Chain Structural Models with different end groups
Key: C – black, H – pink, Br – brown, Si – blue.

OSPC-1 with 2xBr end groups

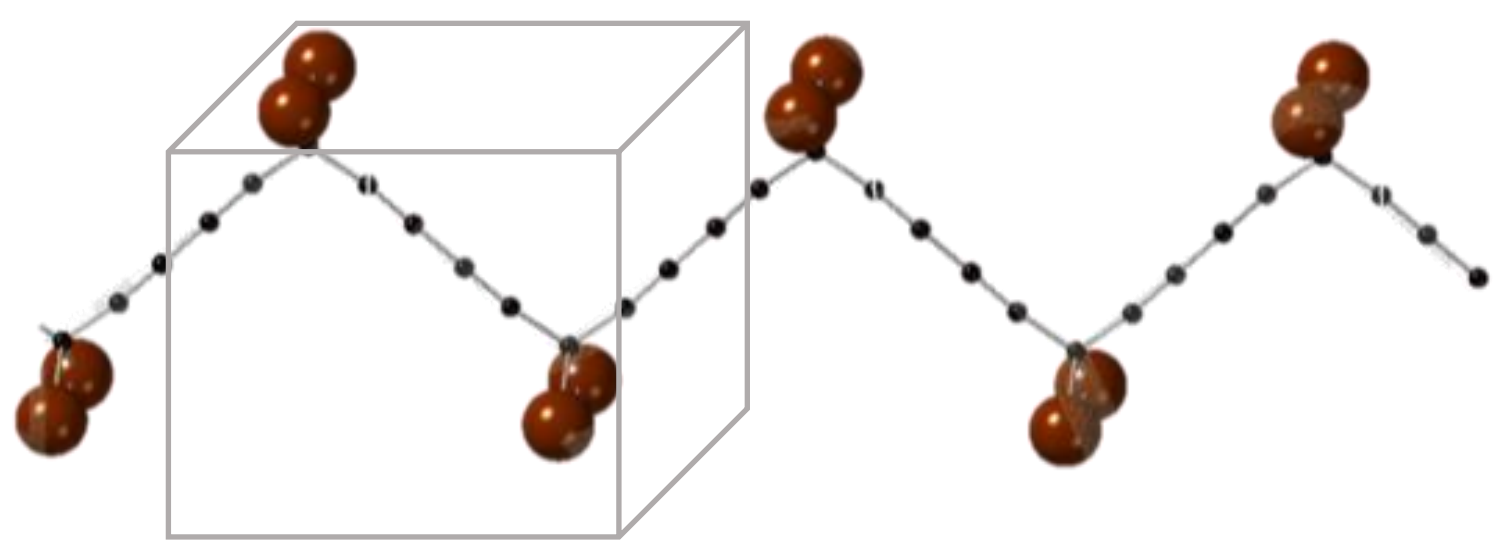

AT – largest cell dimensions

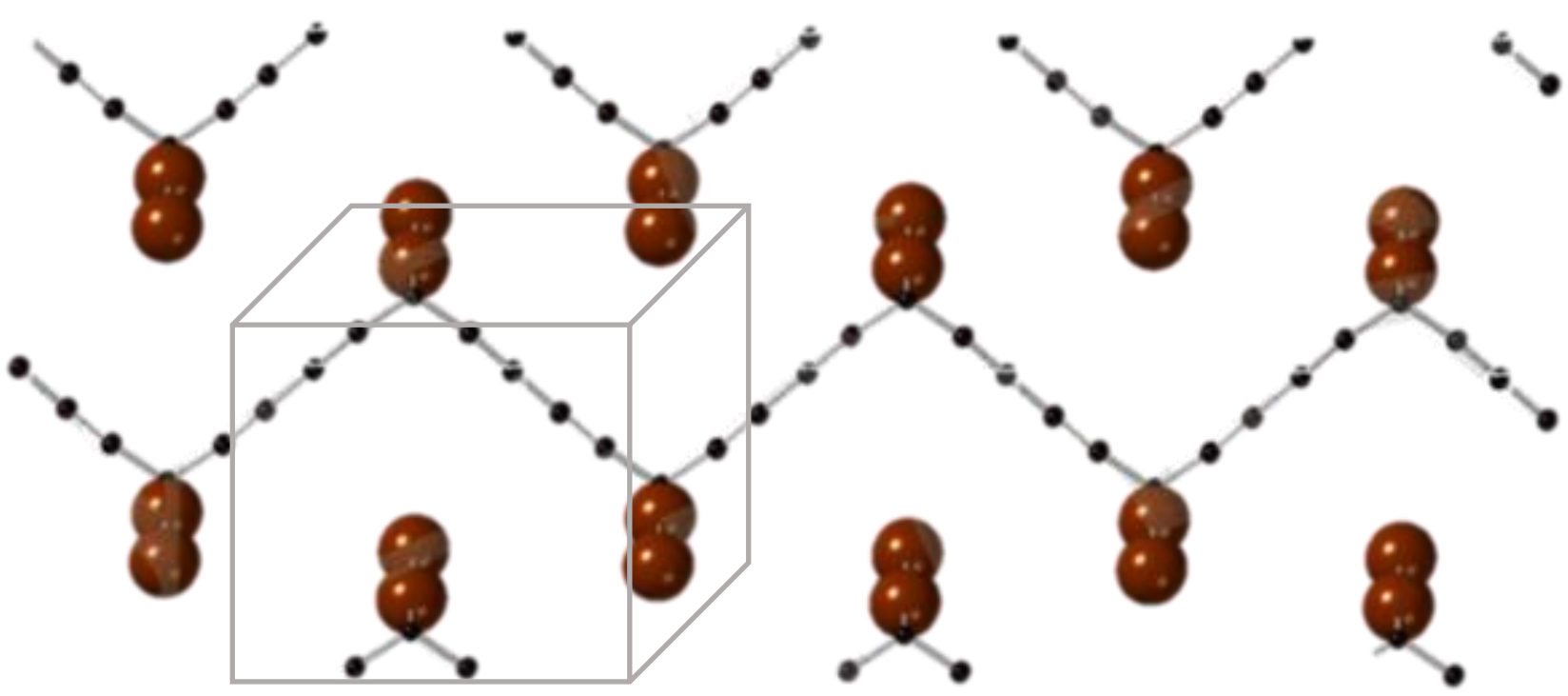

BB – lowest energy structure

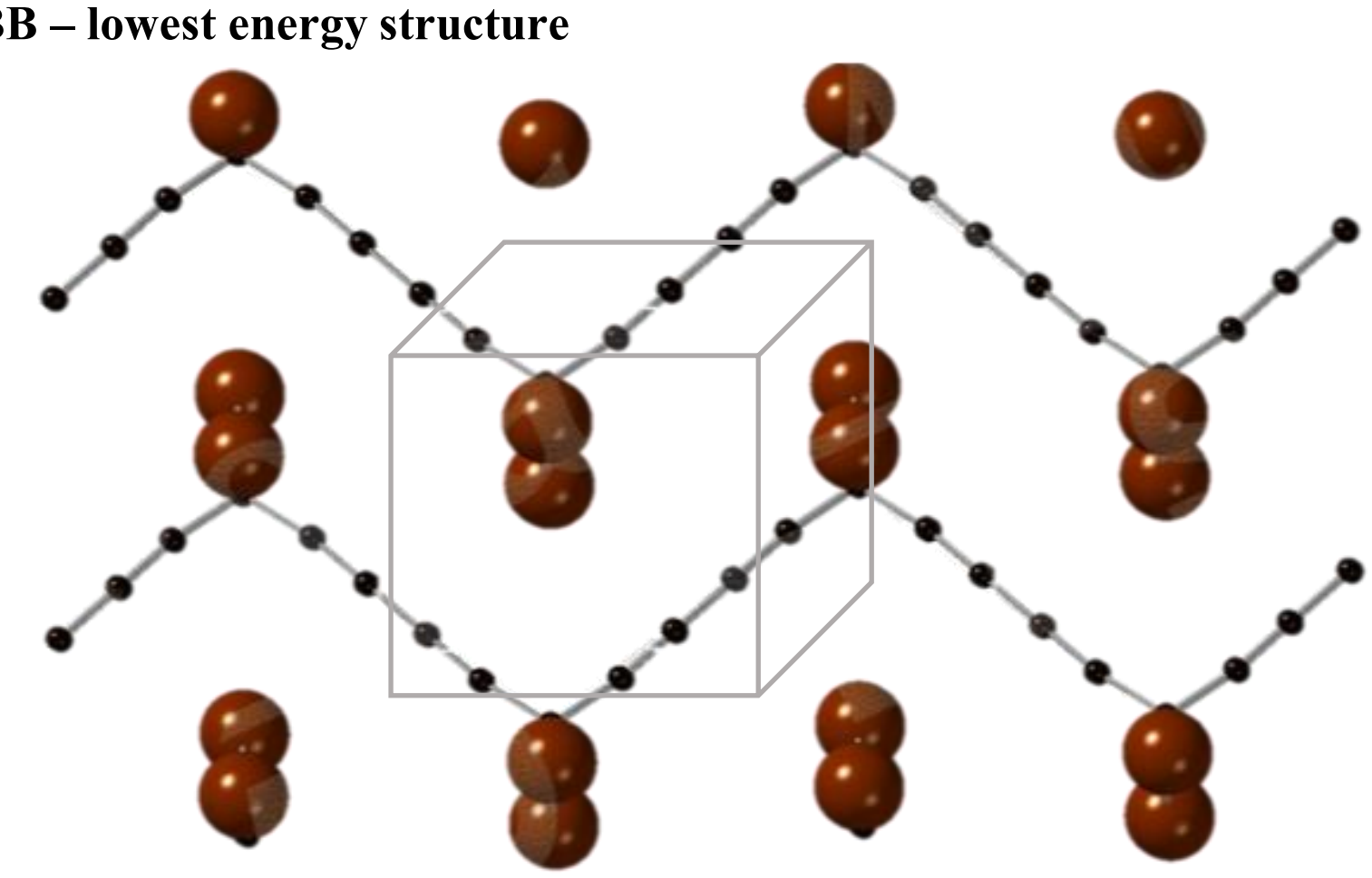

BF – highest energy structure

OSPC-1 with 1xBr and 1xTMS end groups (two views shown of each structure)

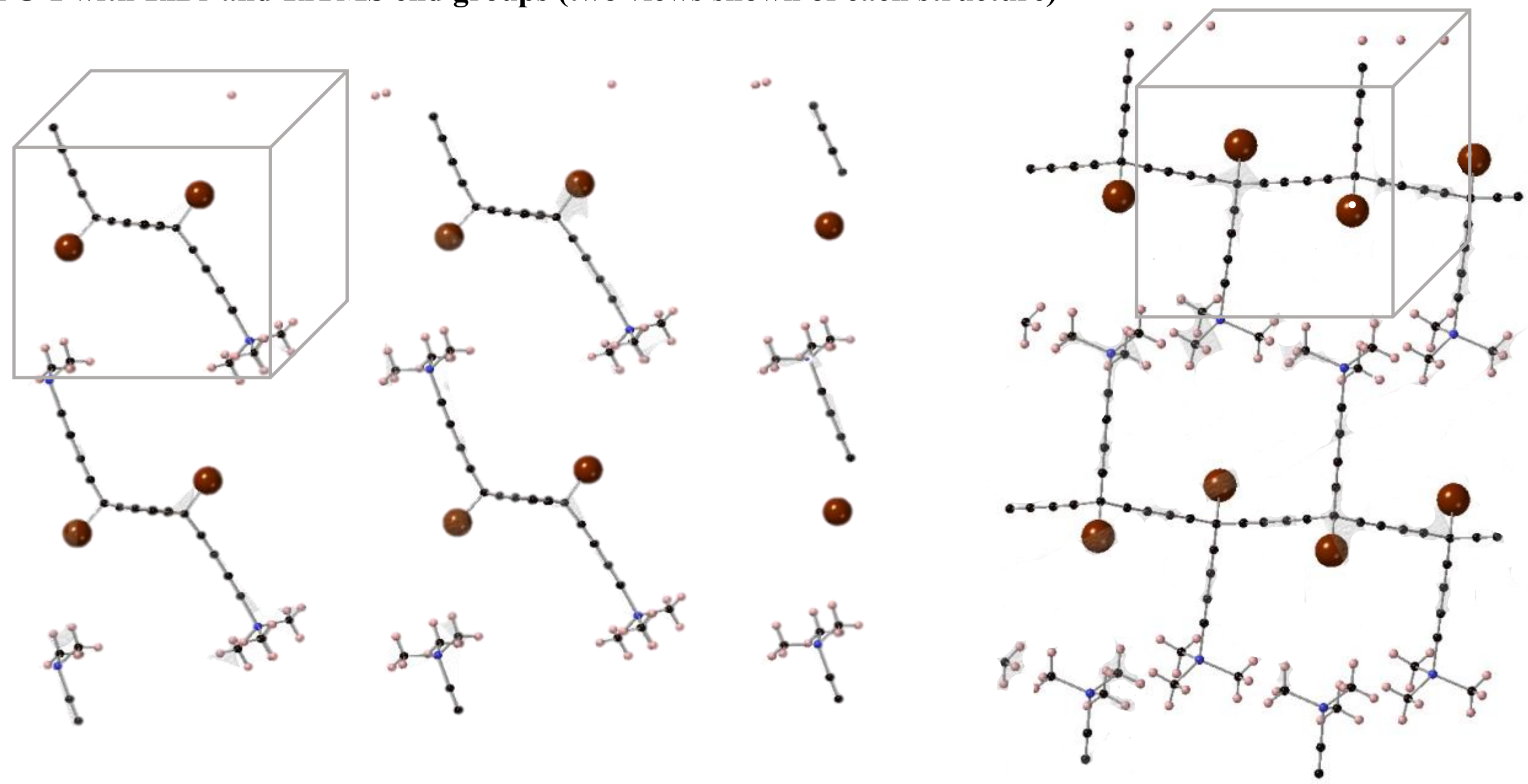

BG – largest cell dimensions

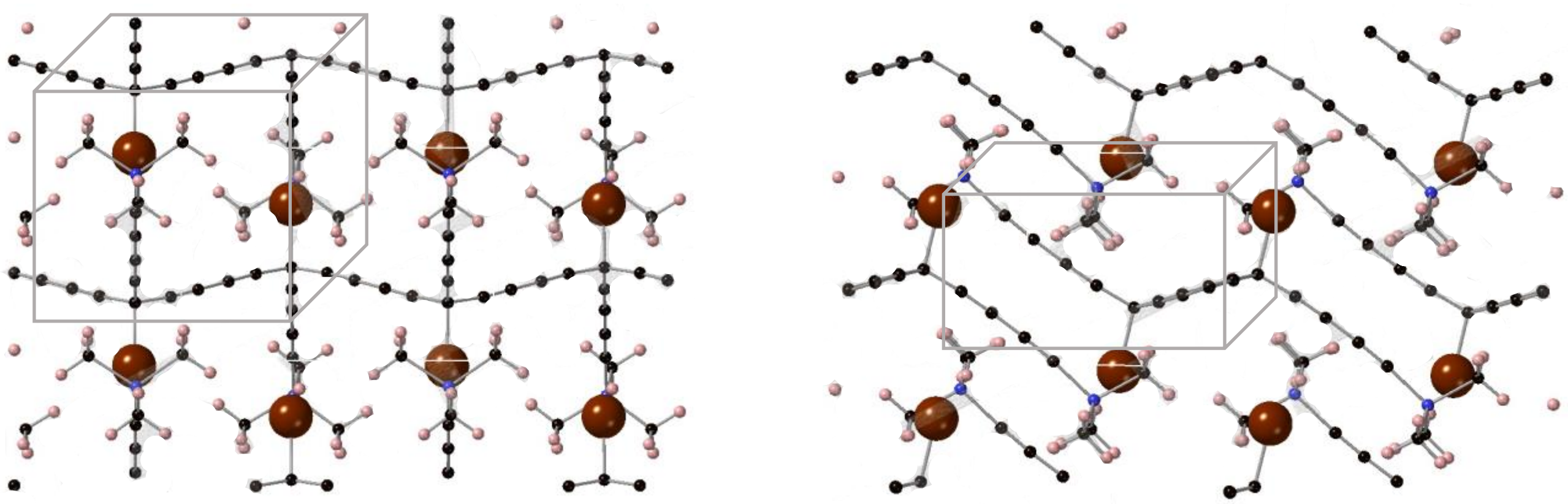

BO – lowest energy structure

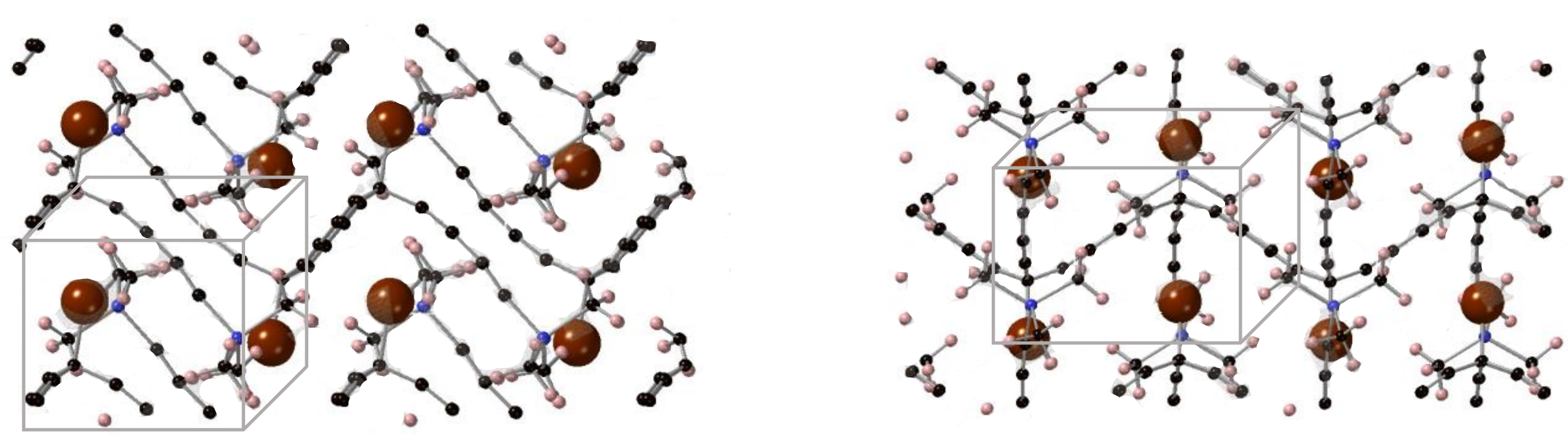

BQ – highest energy structure

Model	Cell length (Å)		
	x	y	z
AT	10.3130	15.0000	15.0000
AU	10.3130	14.0000	14.0000
AV	10.3130	13.0000	13.0000
AW	10.3130	12.0000	12.0000
AX	10.3130	11.0000	11.0000
AY	10.3130	10.0000	10.0000
AZ	10.3130	9.0000	9.0000
BA	10.3130	8.0000	8.0000
BB	10.3130	7.0000	7.0000
BC	10.3130	6.0000	6.0000
BD	10.3130	5.9000	5.9000
BE	10.3130	5.8000	5.8000
BF	10.3130	5.7000	5.7000

Table SI.18: Set 13. OSPC-1 chain (2x Br end groups).

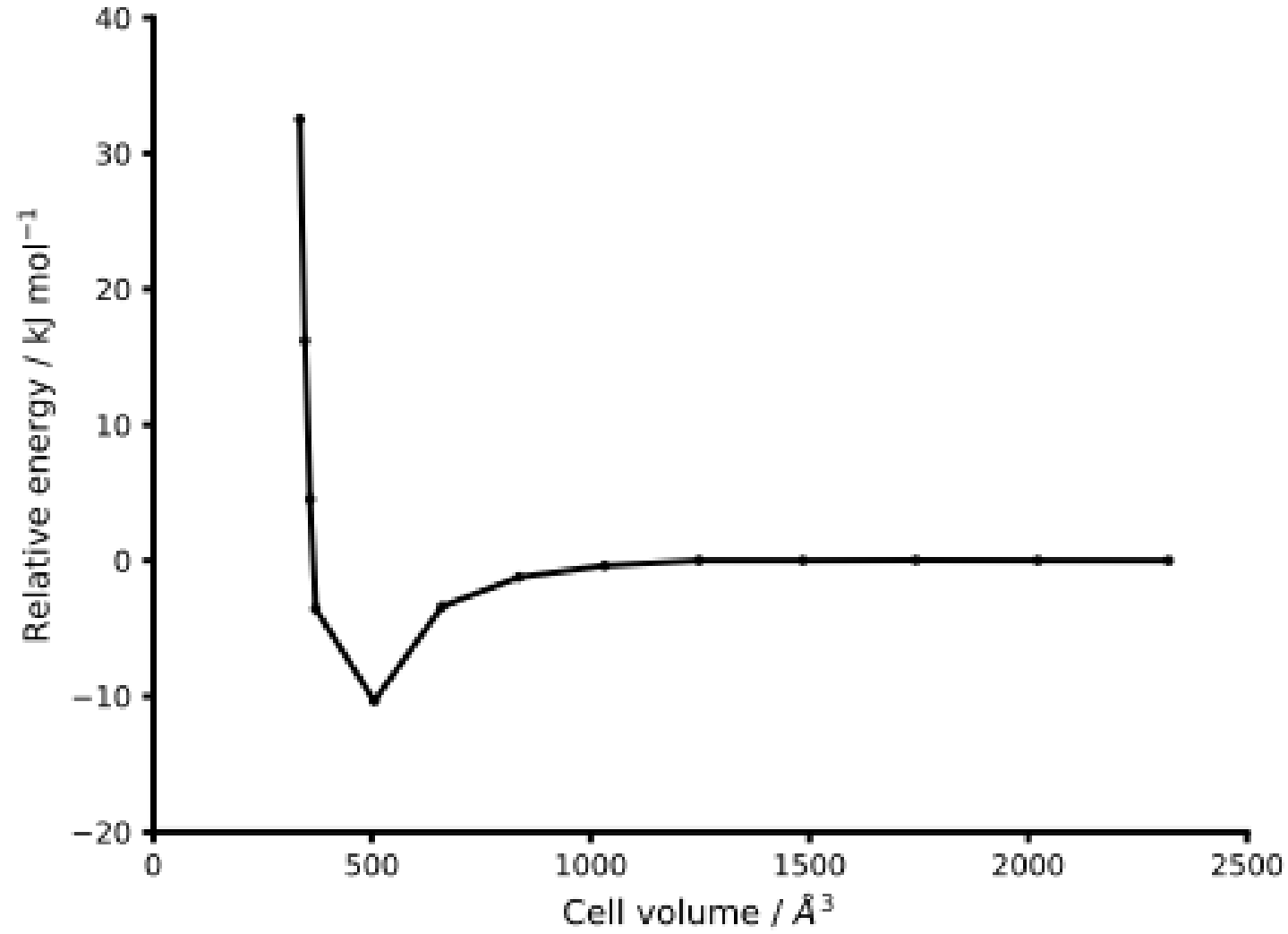

Figure SI.44: Energy of the OSPC-1 (2x Br end groups) optimised chain structure model with a chain in the x direction from Set 13 where the y- and z-cell length changes. Energies are relative to model AT.

Model	Cell length (Å)		
	x	y	z
BG	10.3130	15.0000	20.0000
BH	10.3130	14.0000	19.0000
BI	10.3130	13.0000	18.0000
BJ	10.3130	12.0000	16.0000
BK	10.3130	11.0000	15.0000
BL	10.3130	10.0000	14.0000
BM	10.3130	9.0000	13.0000
BN	10.3130	8.0000	12.0000
BO	10.3130	7.0000	11.0000
BP	10.3130	6.0000	10.0000
BQ	10.3130	5.5000	9.5000

Table SI.19: Set 14. OSPC-1 chain (1x Br, 1x TMS end groups), changing y and z values.

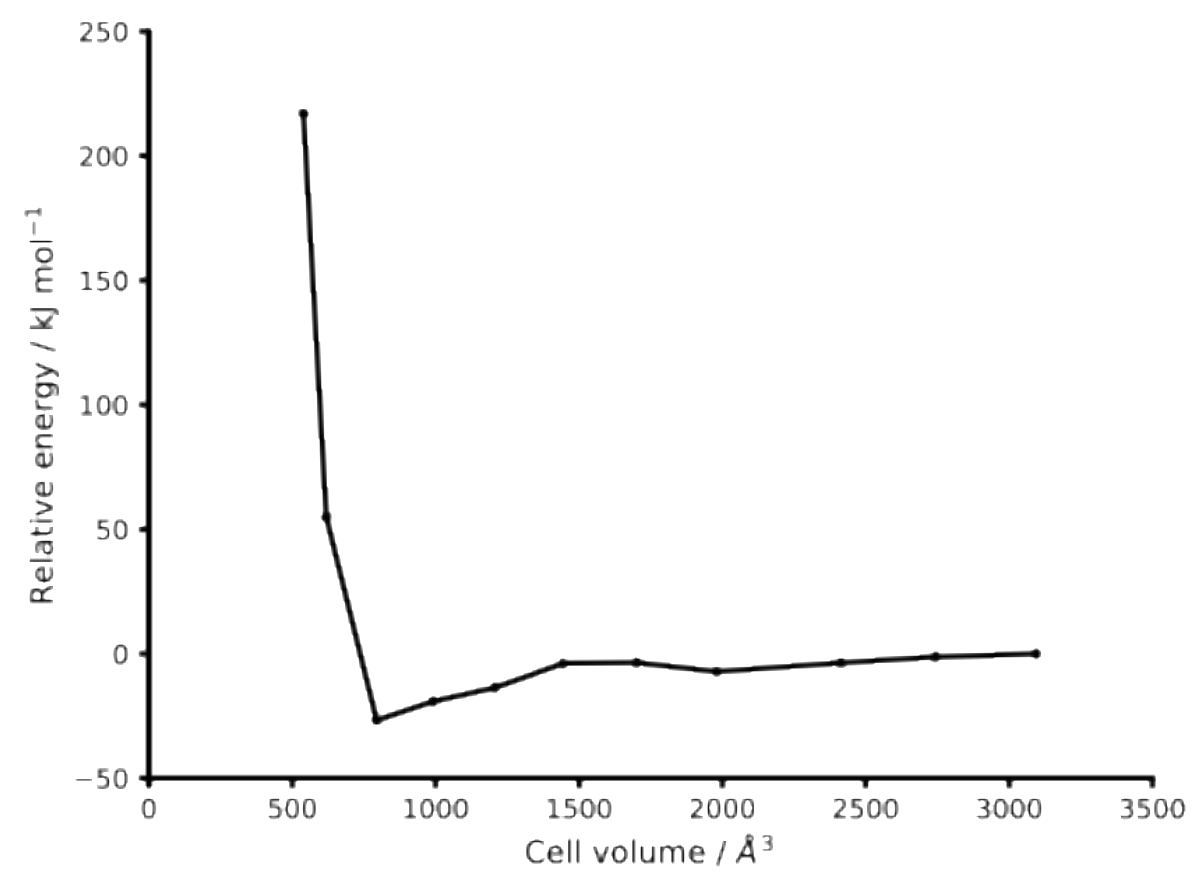

Figure SI.45: Energy of the OSPC-1 (1x Br, 1x TMS end groups) optimised chain structure model with a chain in the x direction from Set 14 where the y- and z-cell length changes. Energies are relative to model BG.

Section 8. Energy Mapping

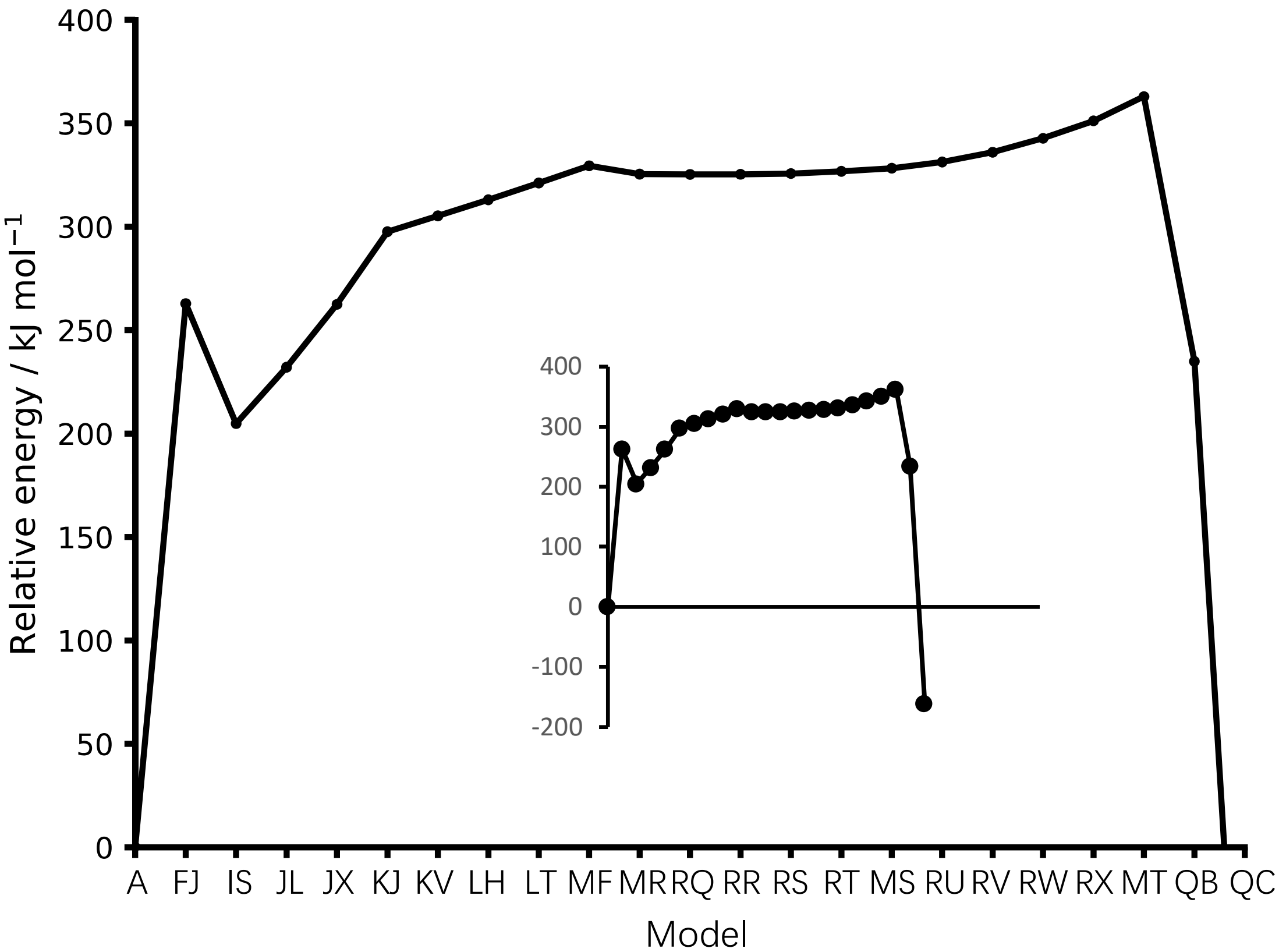

Figure SI.46: Energy pathway to the denser models for a single chain of OSPC-1 in the x direction from model A (10.9 x 10.9 x 10.9 Å) to model QC (10.9 x 2.9 x 6.0 Å). All models used are circled in red in the respective energy versus cell length plots.

(a)

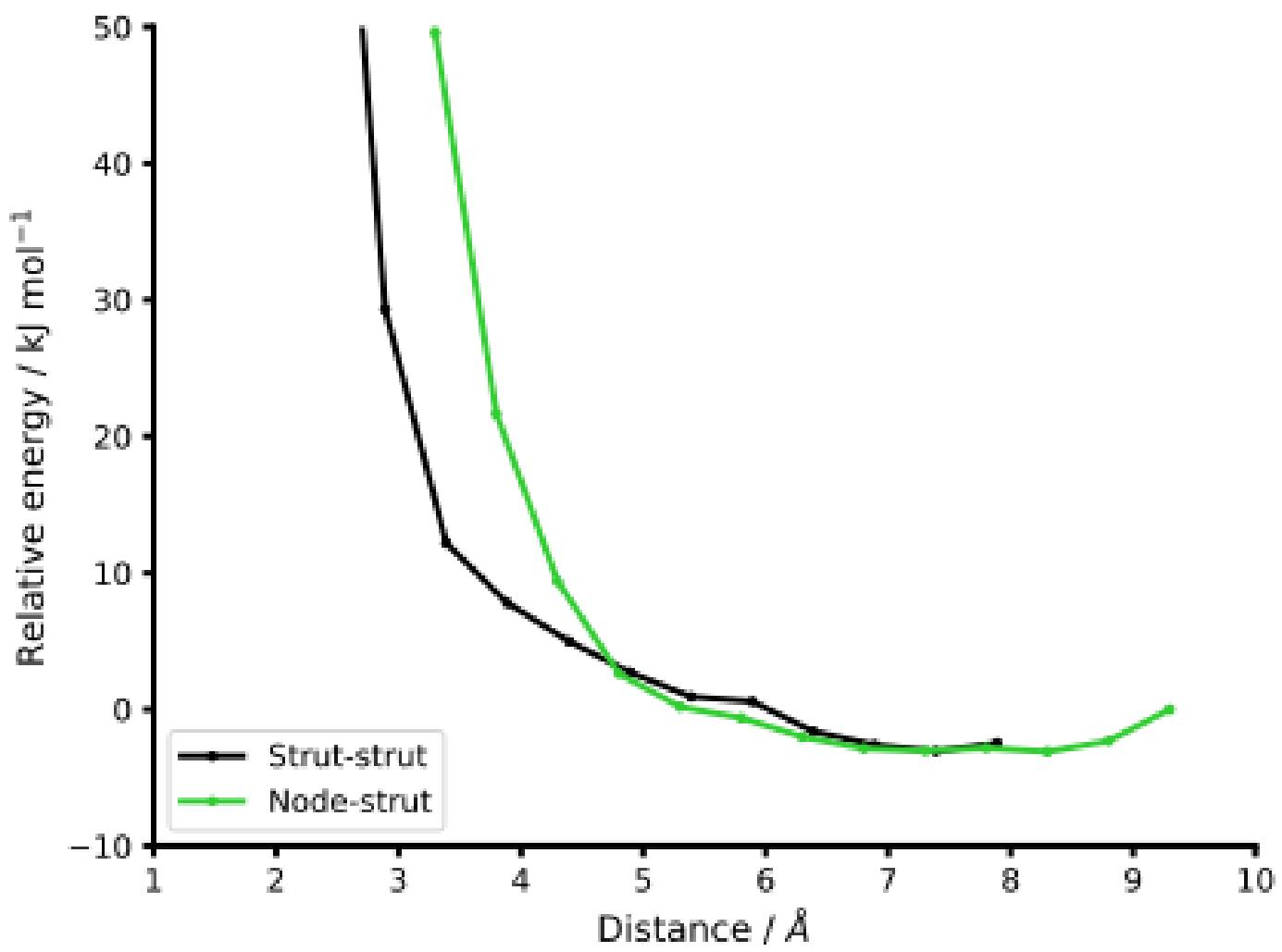

(b)

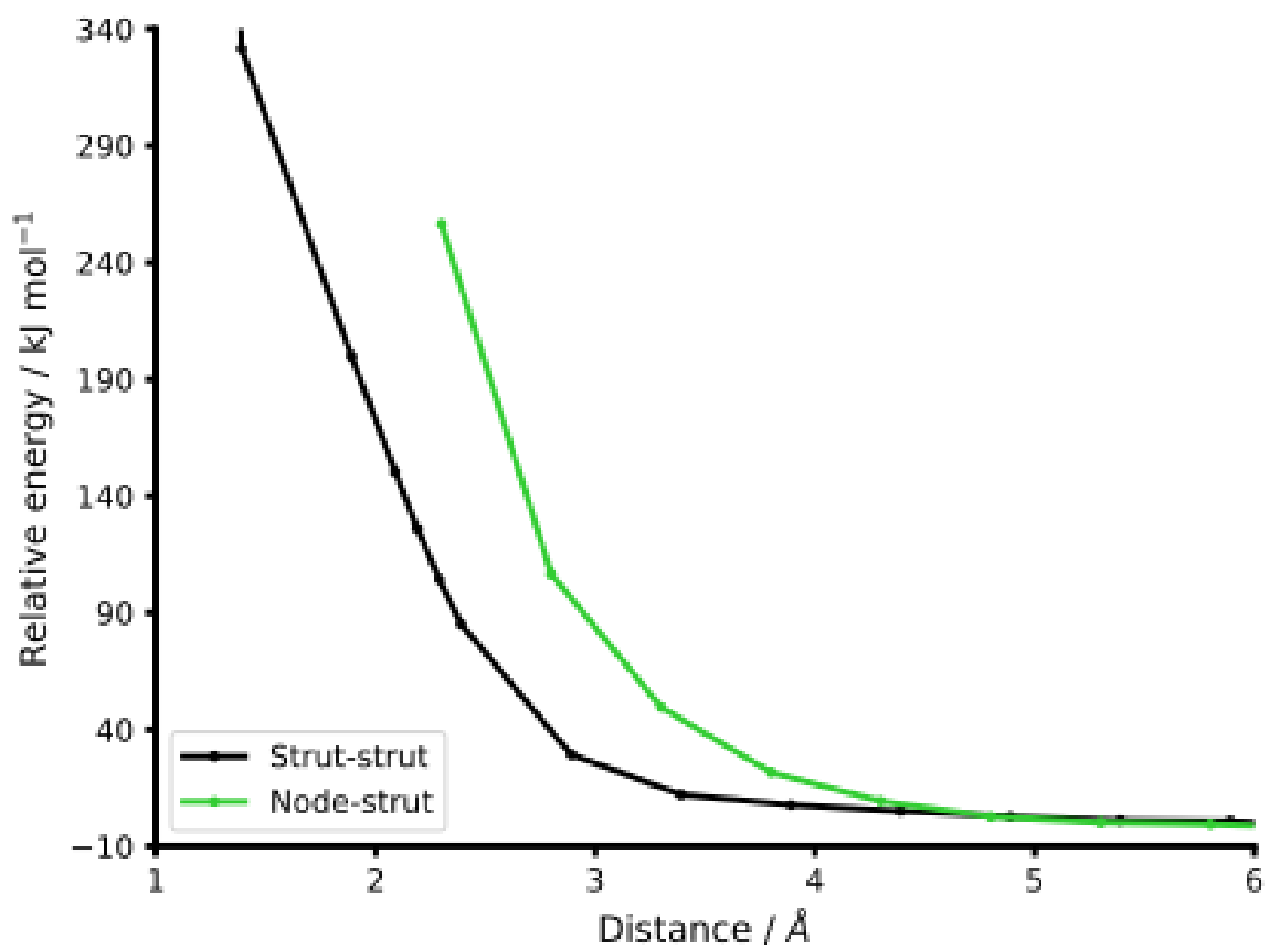

Figure SI.47: (a) Energy of dimer-dimer interaction as a function of distance. **(b)** zoomed in section. Black – the distance between two central *sp* carbon atoms, green – the distance between the central *sp* carbon atom of one dimer and the *sp*^{3} node of the other.

(a)

(b)

(c)

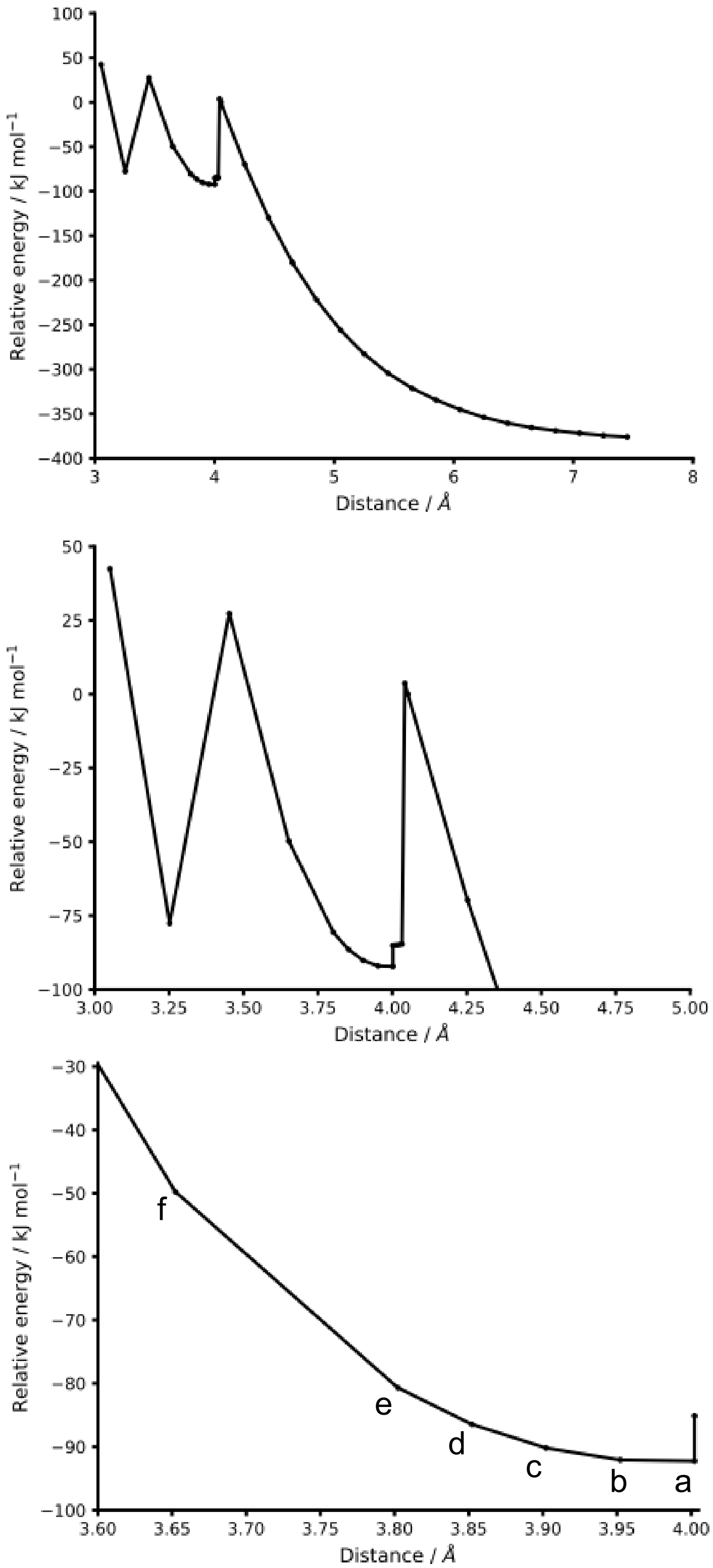

Figure SI.48: (a) Energy of dimer-dimer-dimer interaction as a function of distance between two central *sp* carbon atoms of the outer dimers. **(b)** Zoomed in section showing the energy barriers between 3.0 Å and 4.0 Å. **(c)** Zoomed in section showing the energy well and model name assignments.

Section 9. NMR Spectra

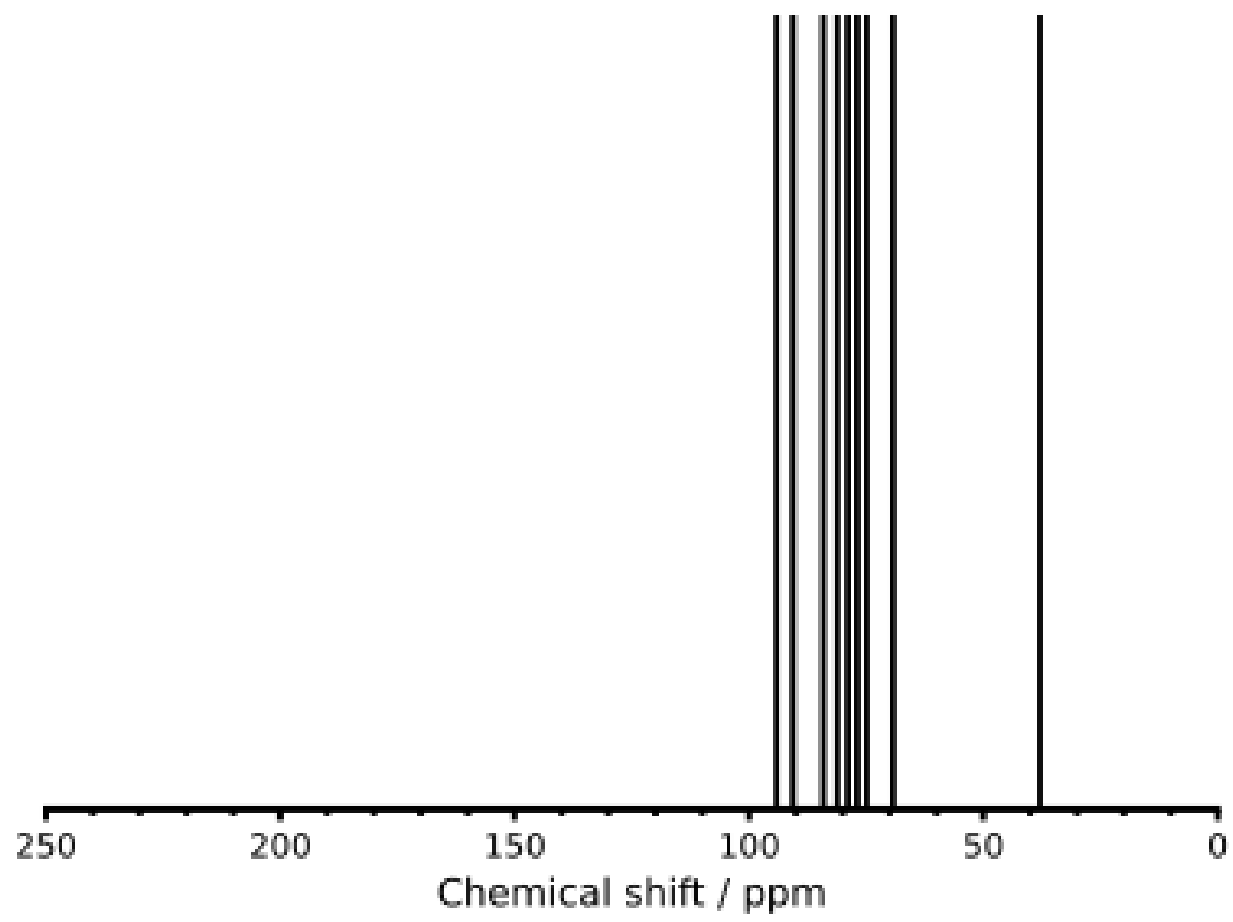

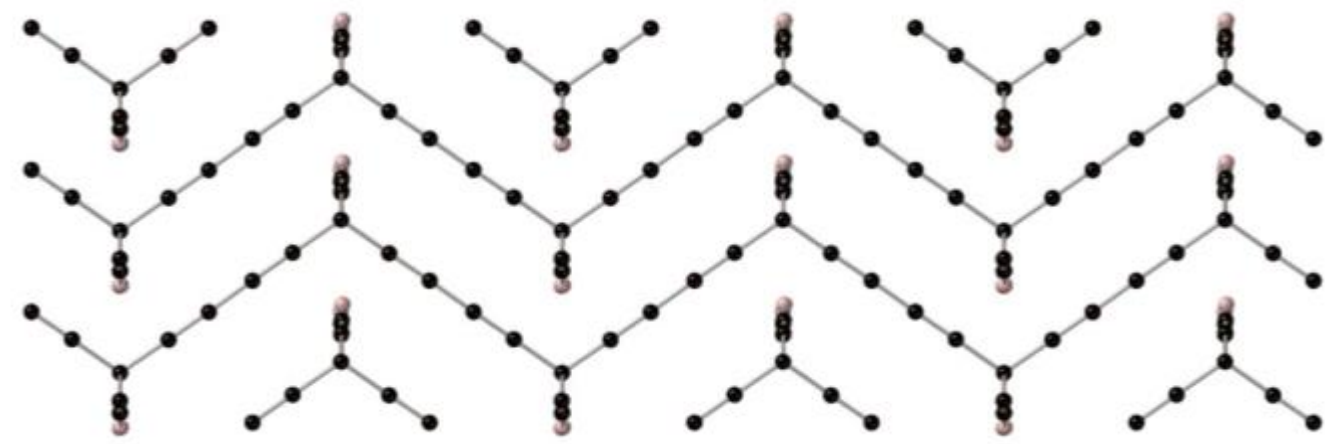

FJ

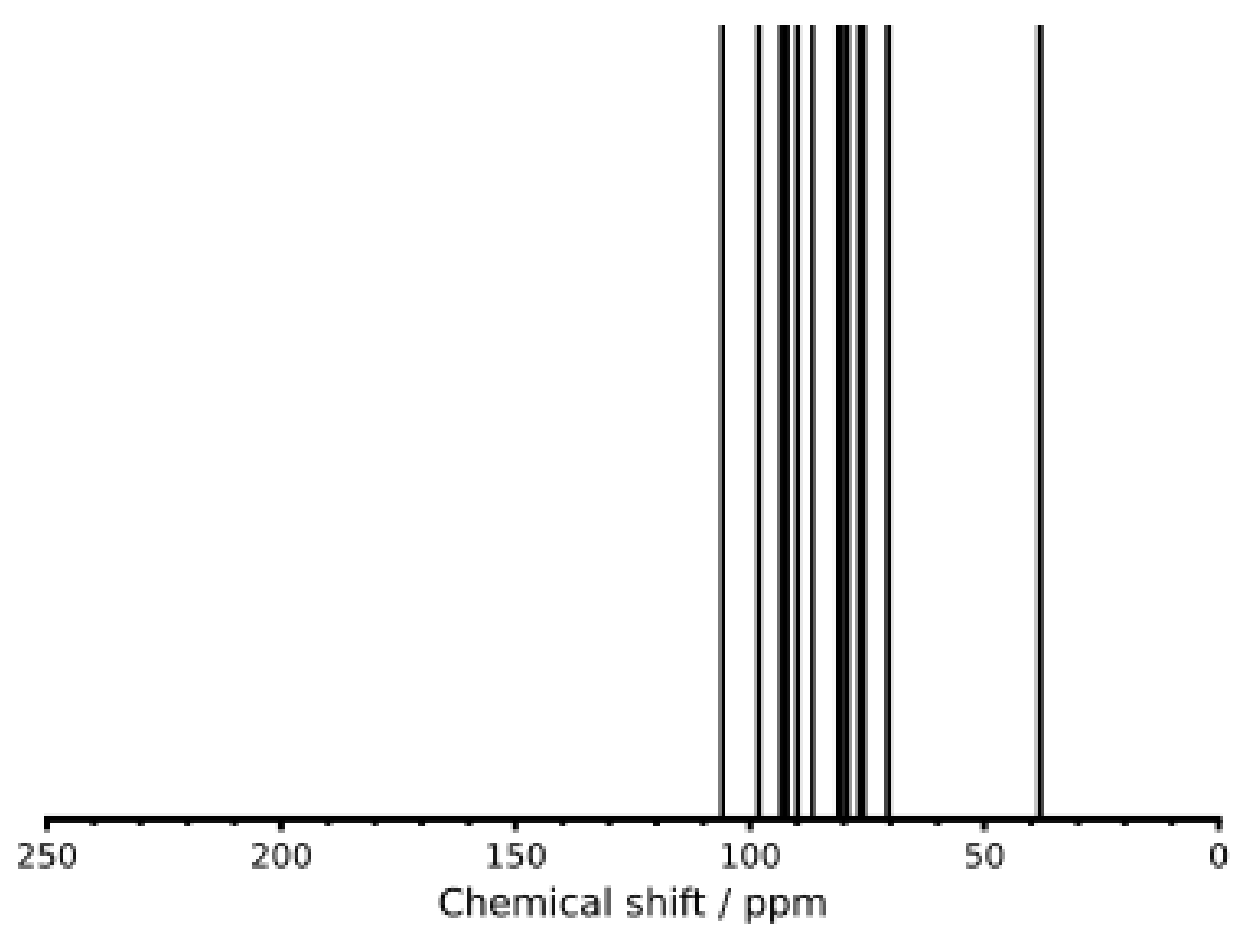

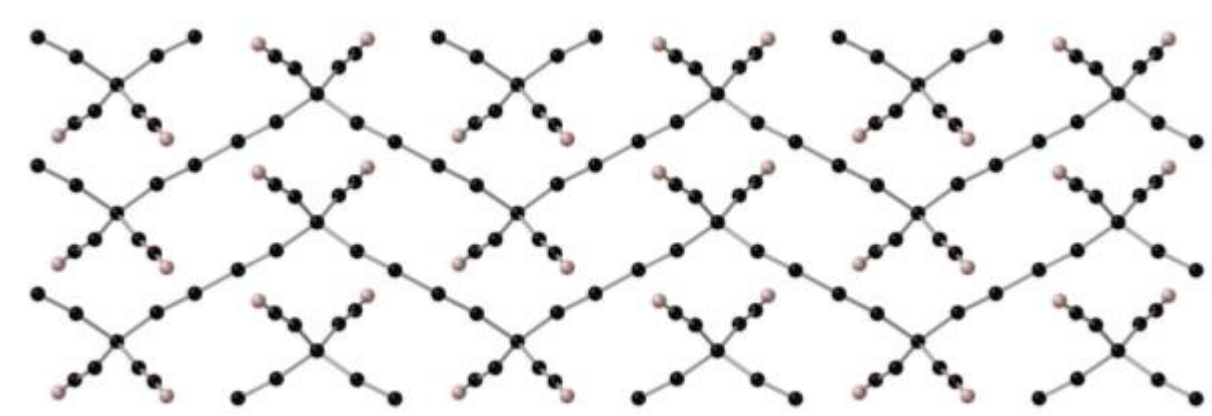

IS

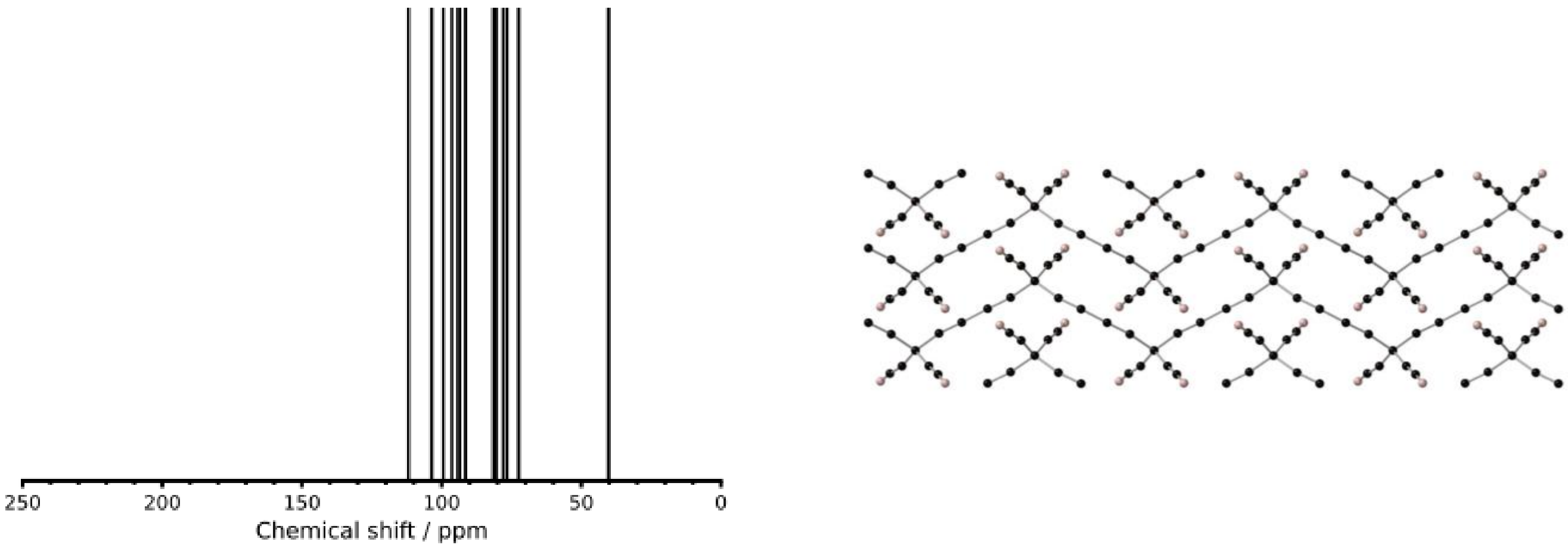
250 200 150 100 50 0
Chemical shift / ppm

JL

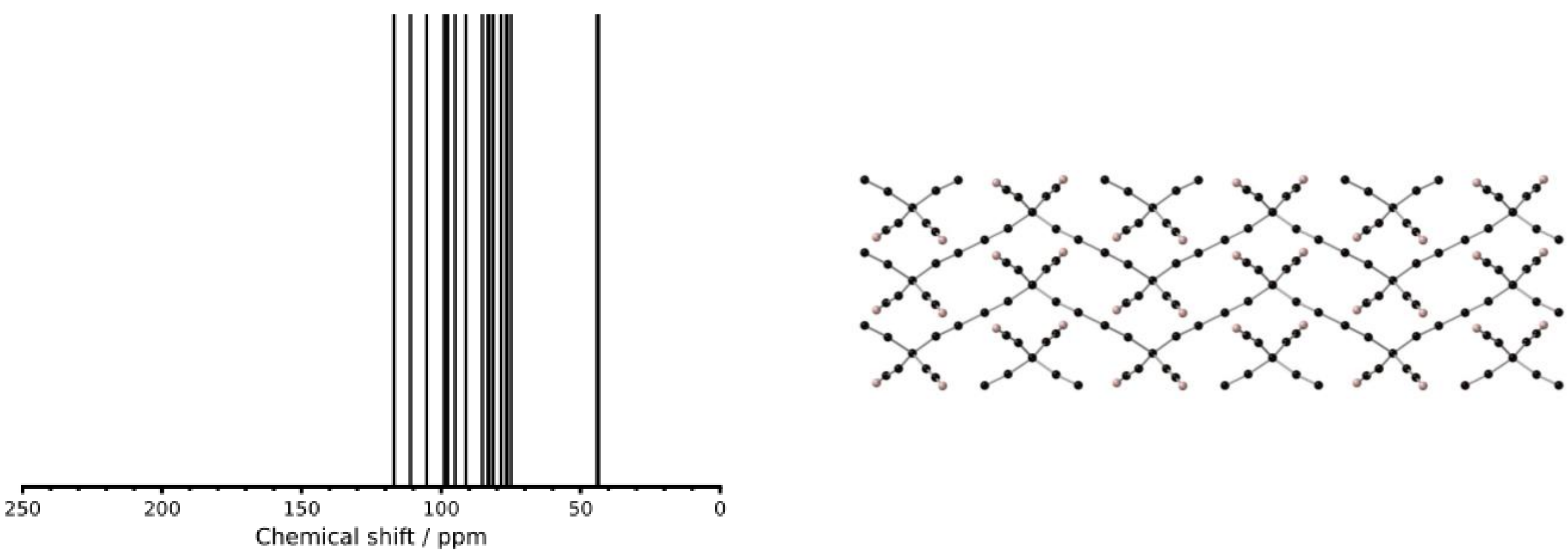
250 200 150 100 50 0
Chemical shift / ppm

JX

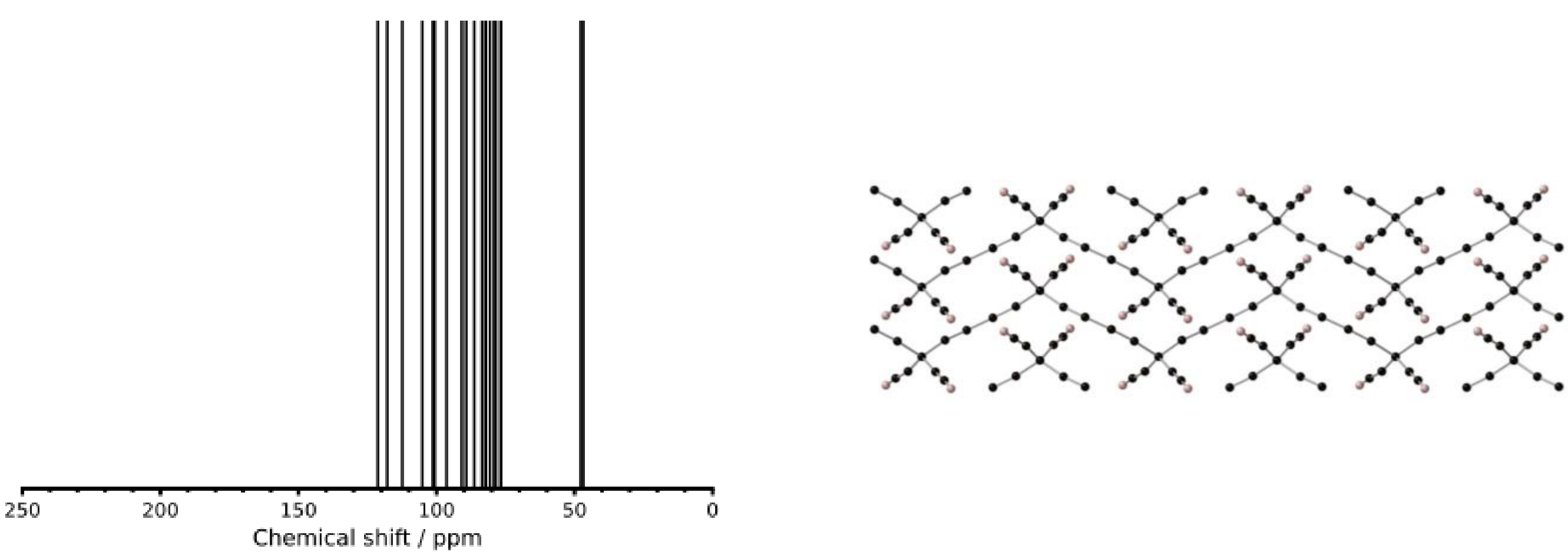
250 200 150 100 50 0
Chemical shift / ppm

KJ

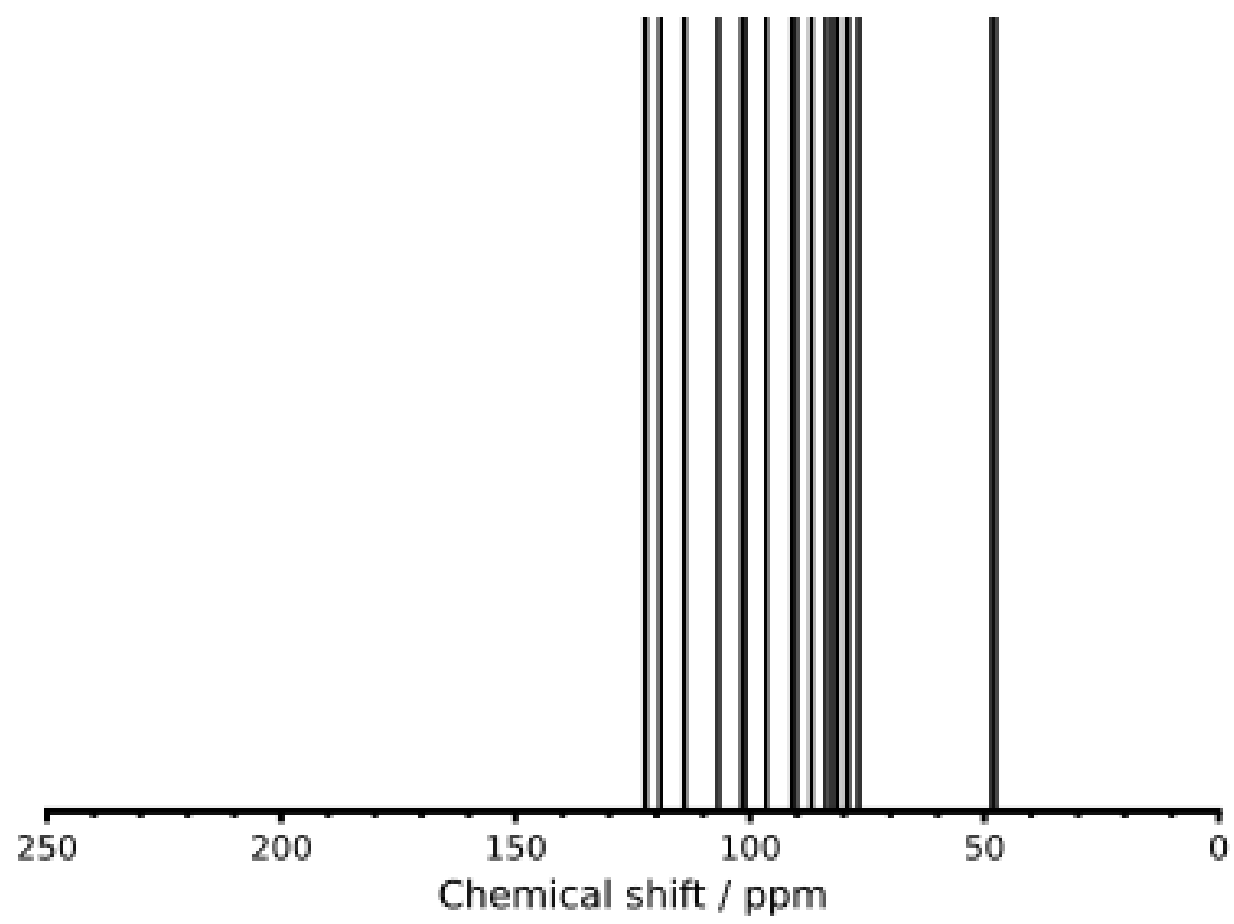
250
200
150
100
50
0
Chemical shift / ppm

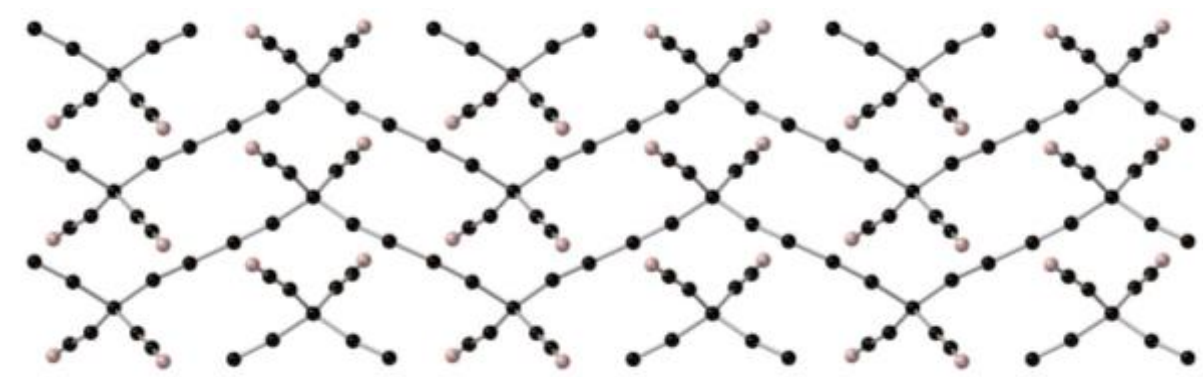

KV

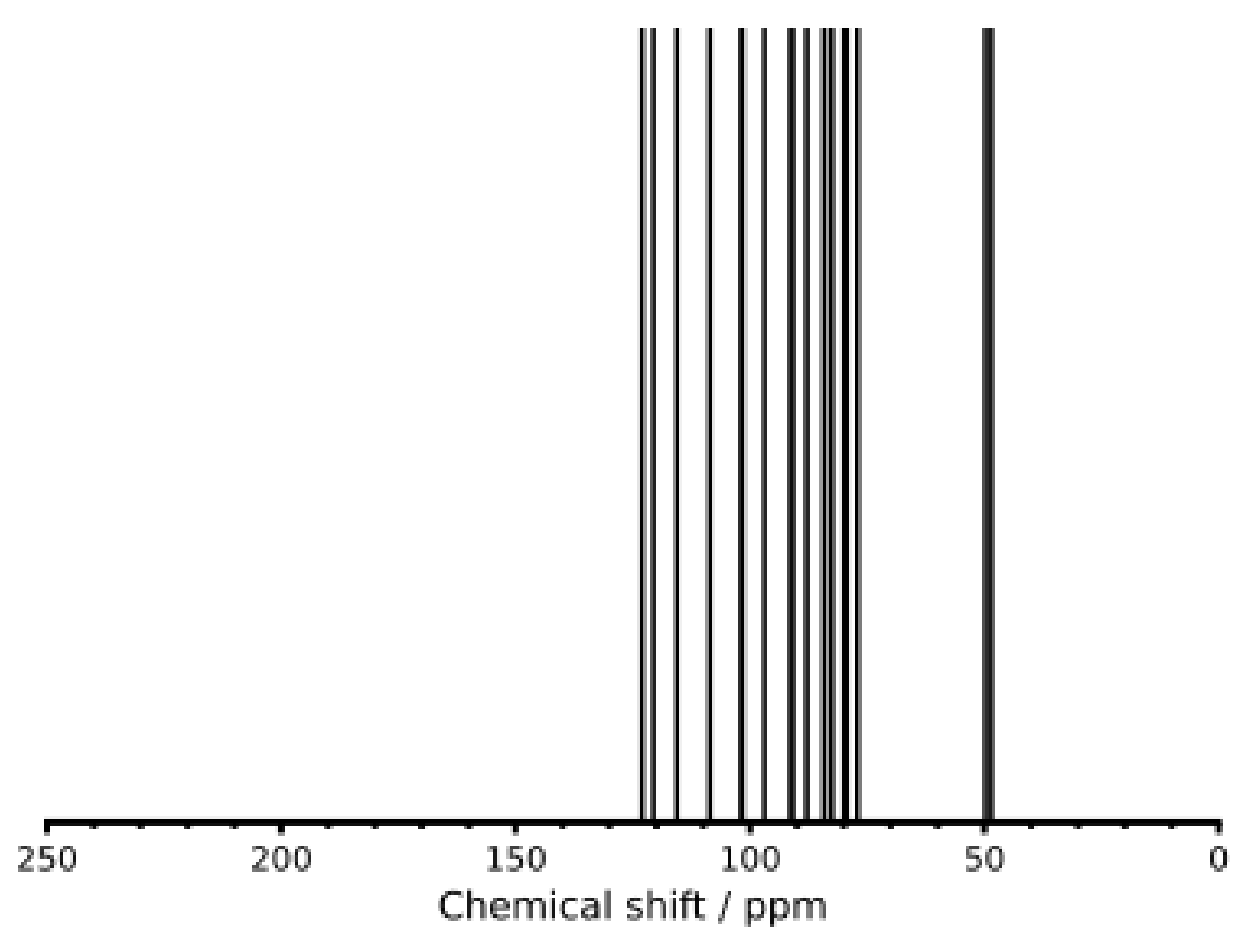
250
200
150
100
50
0
Chemical shift / ppm

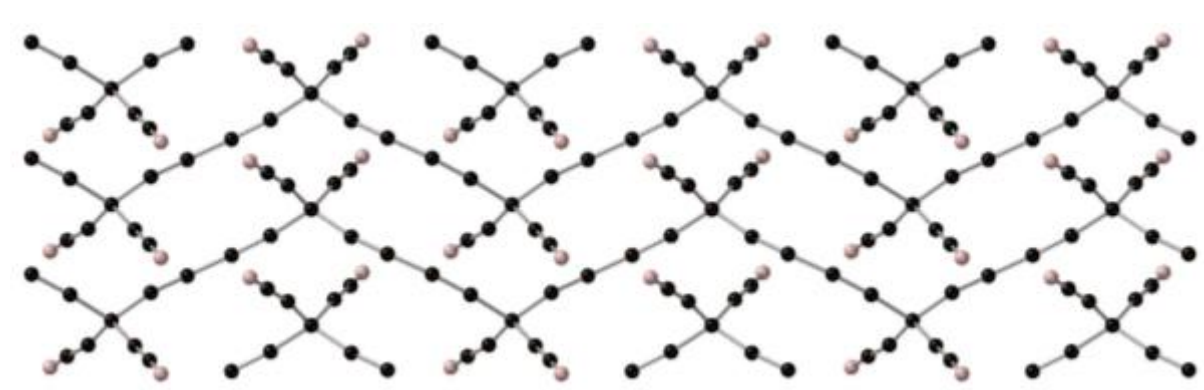

LH

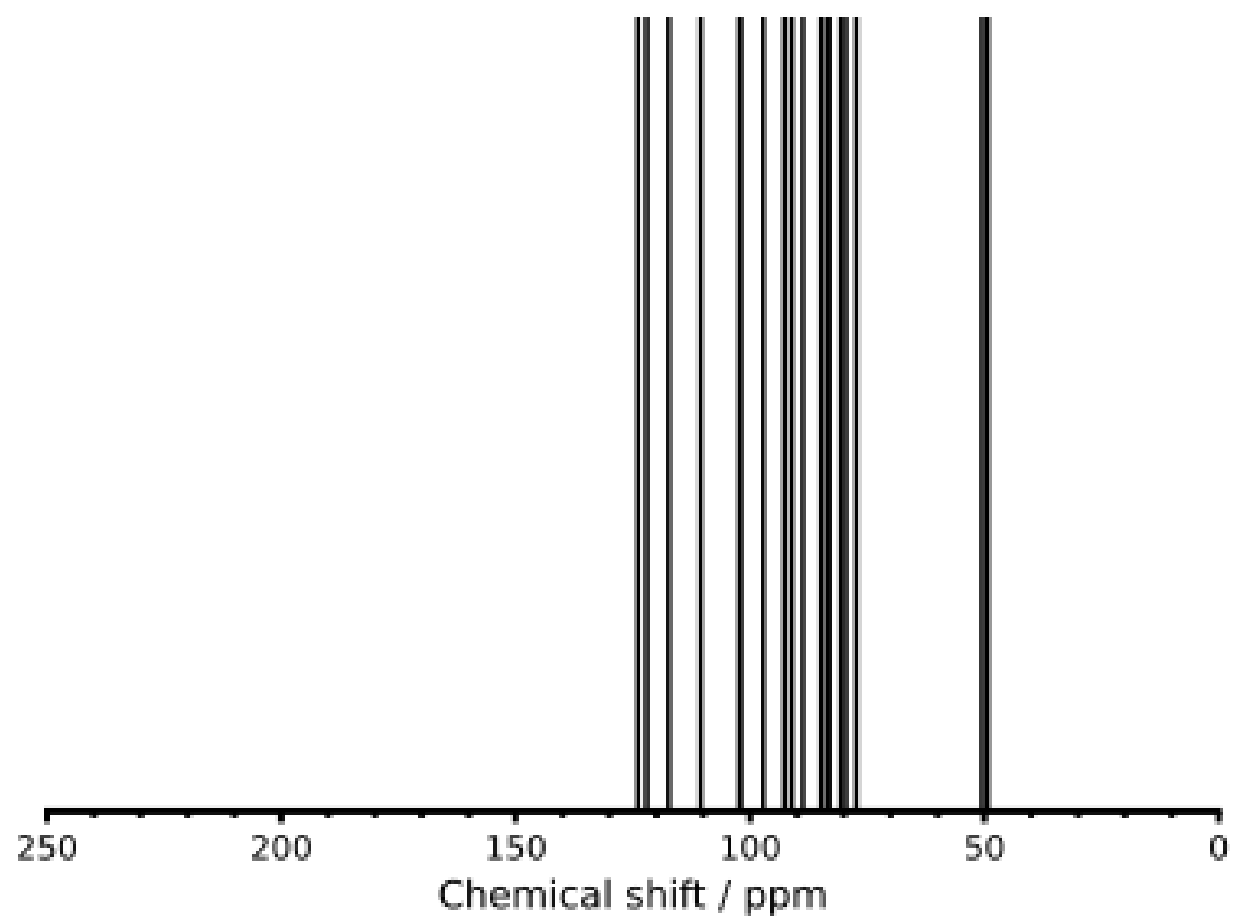

250
200
150
100
50
0
Chemical shift / ppm

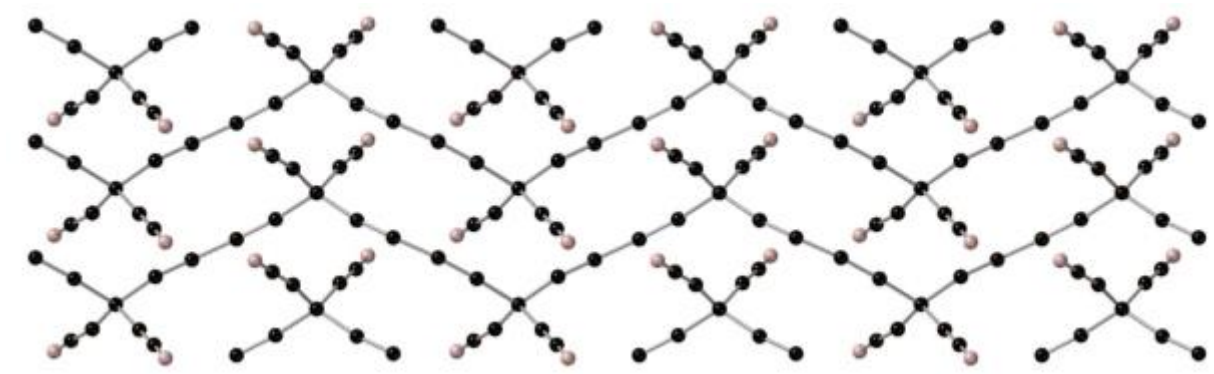

LT

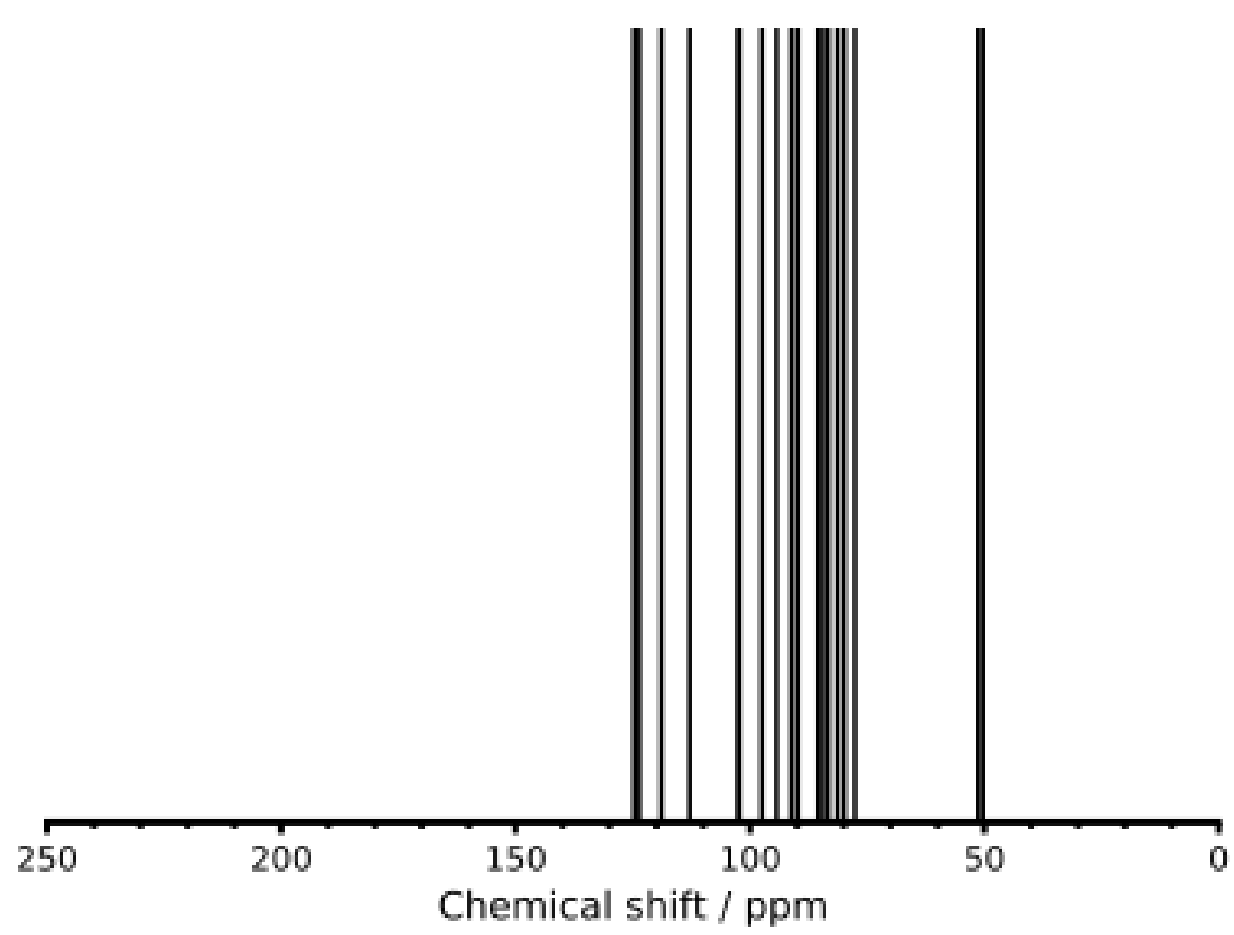

250
200
150
100
50
0
Chemical shift / ppm

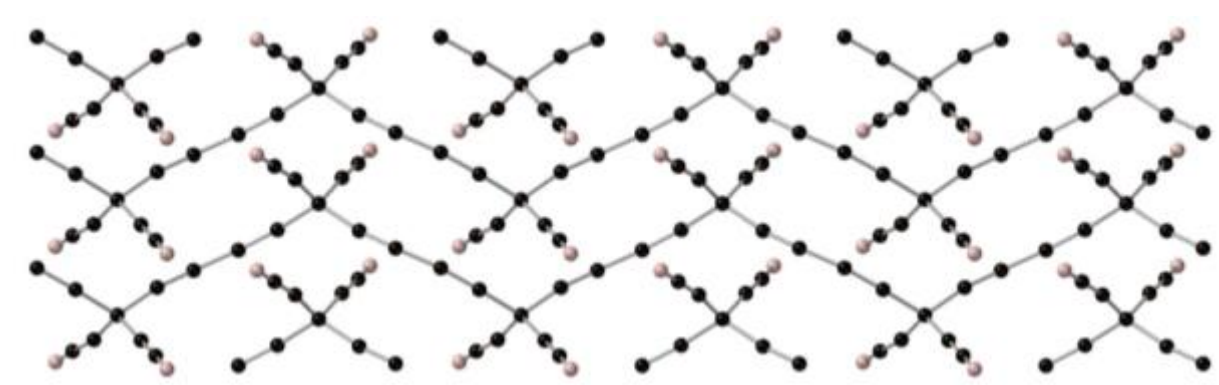

MF

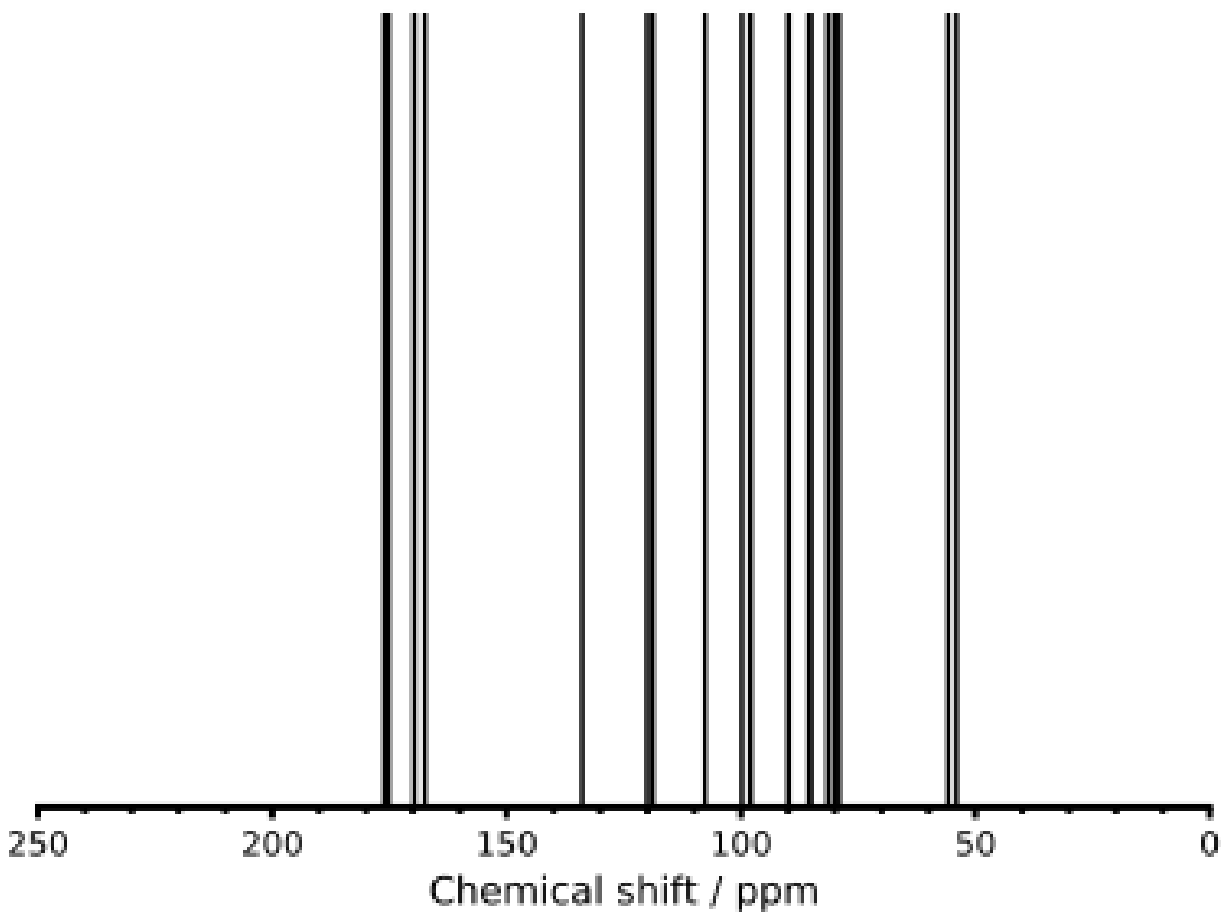
250
200
150
100
50
0
Chemical shift / ppm
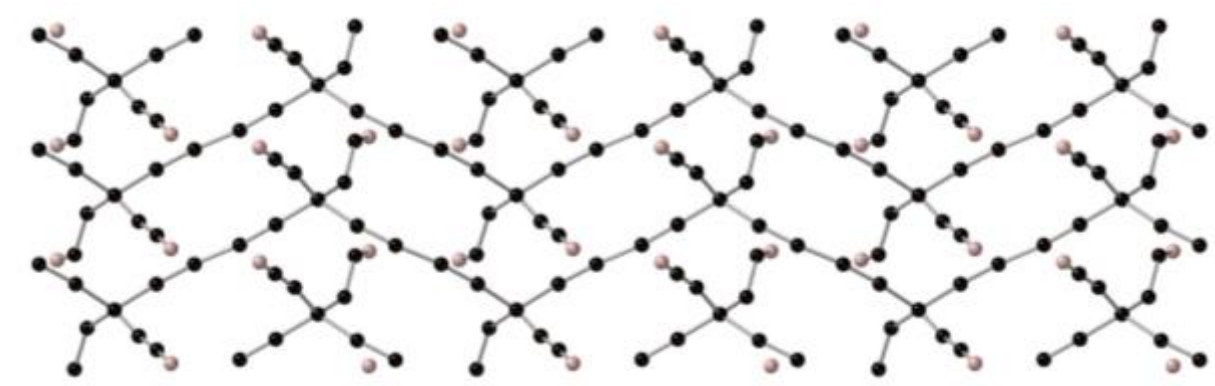

MR

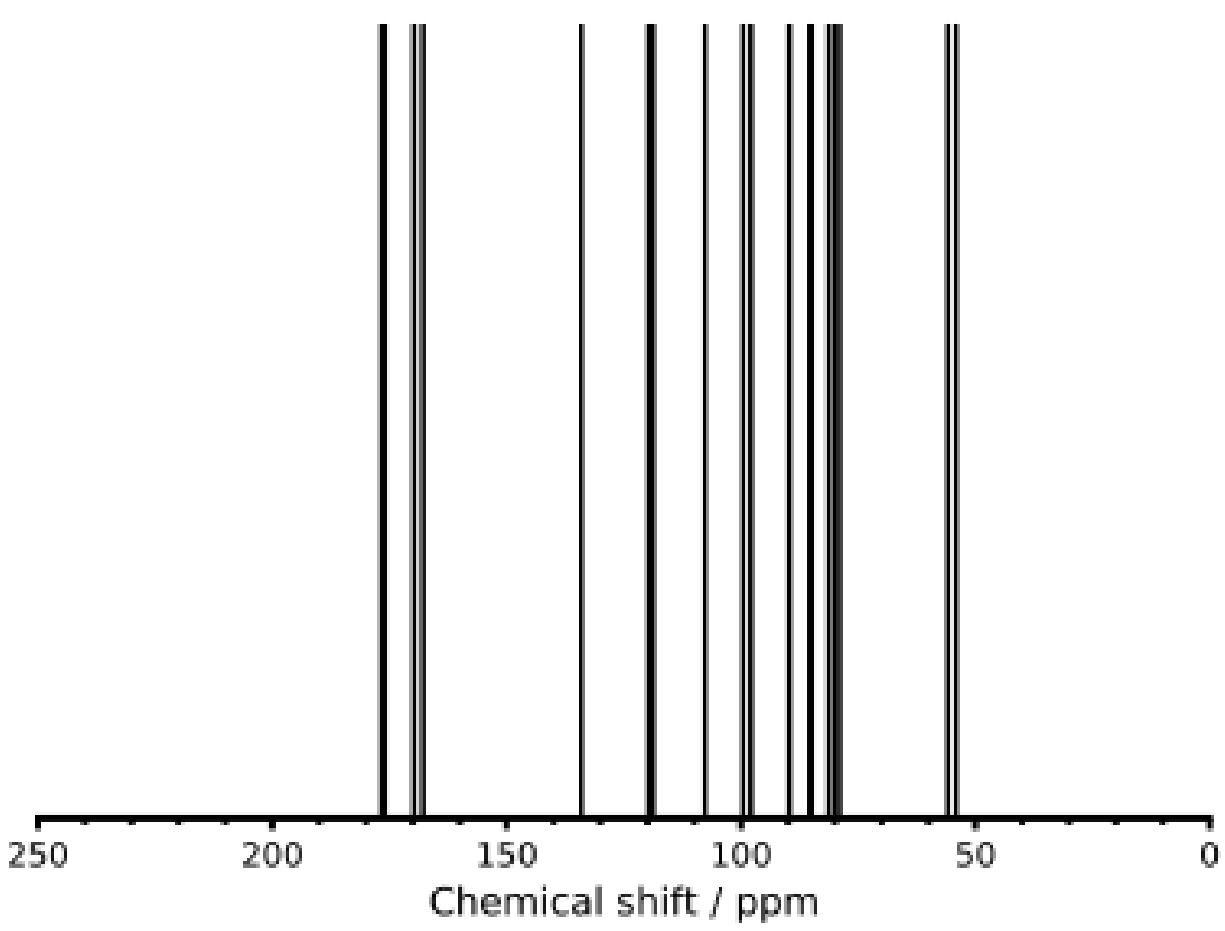
250
200
150
100
50
0
Chemical shift / ppm
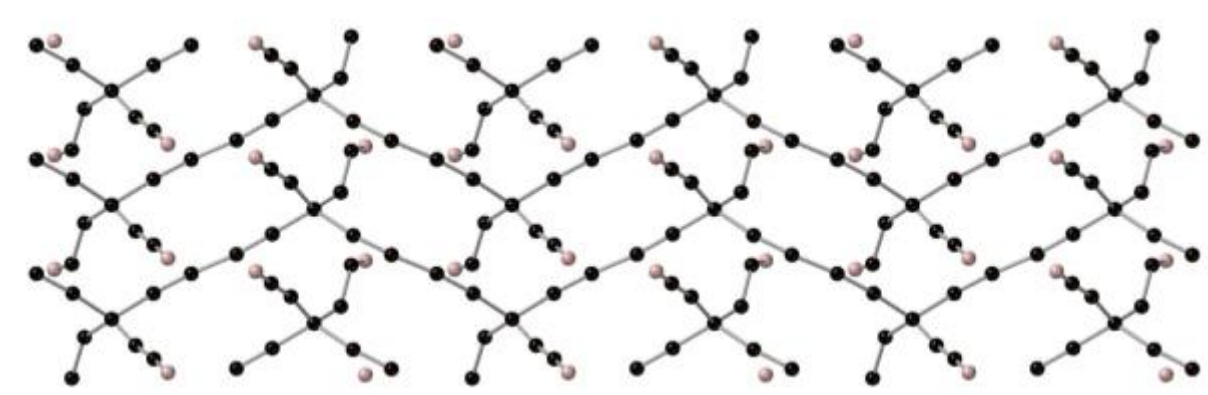

RQ

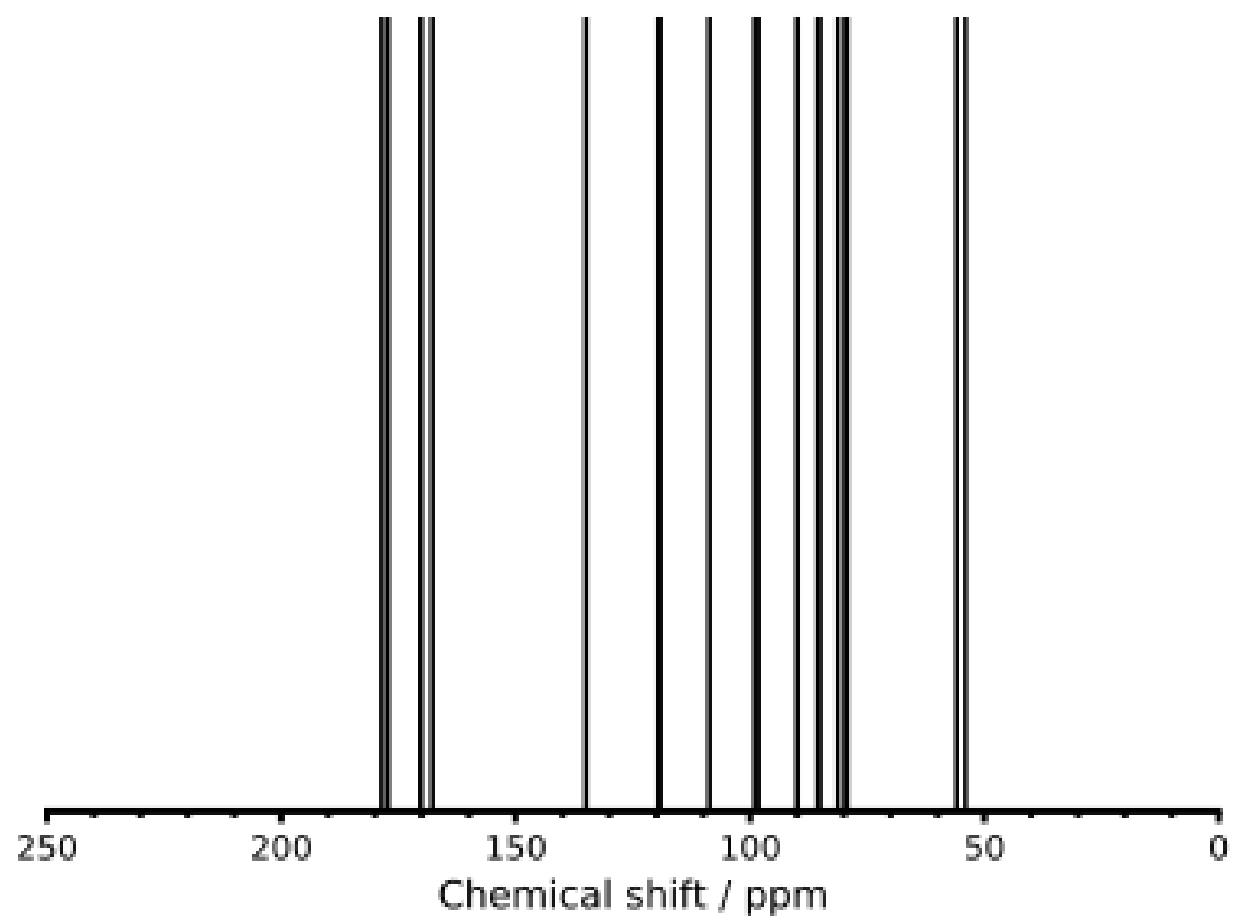
250
200
150
100
50
0
Chemical shift / ppm

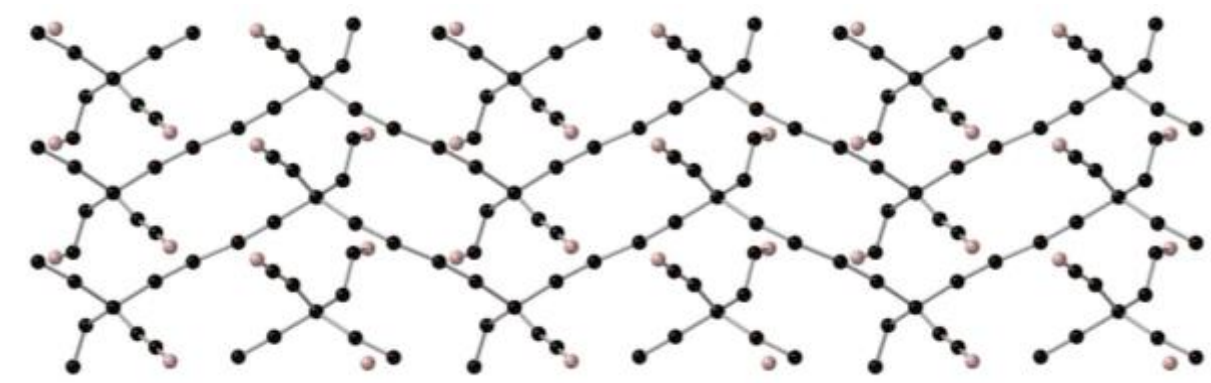

RR

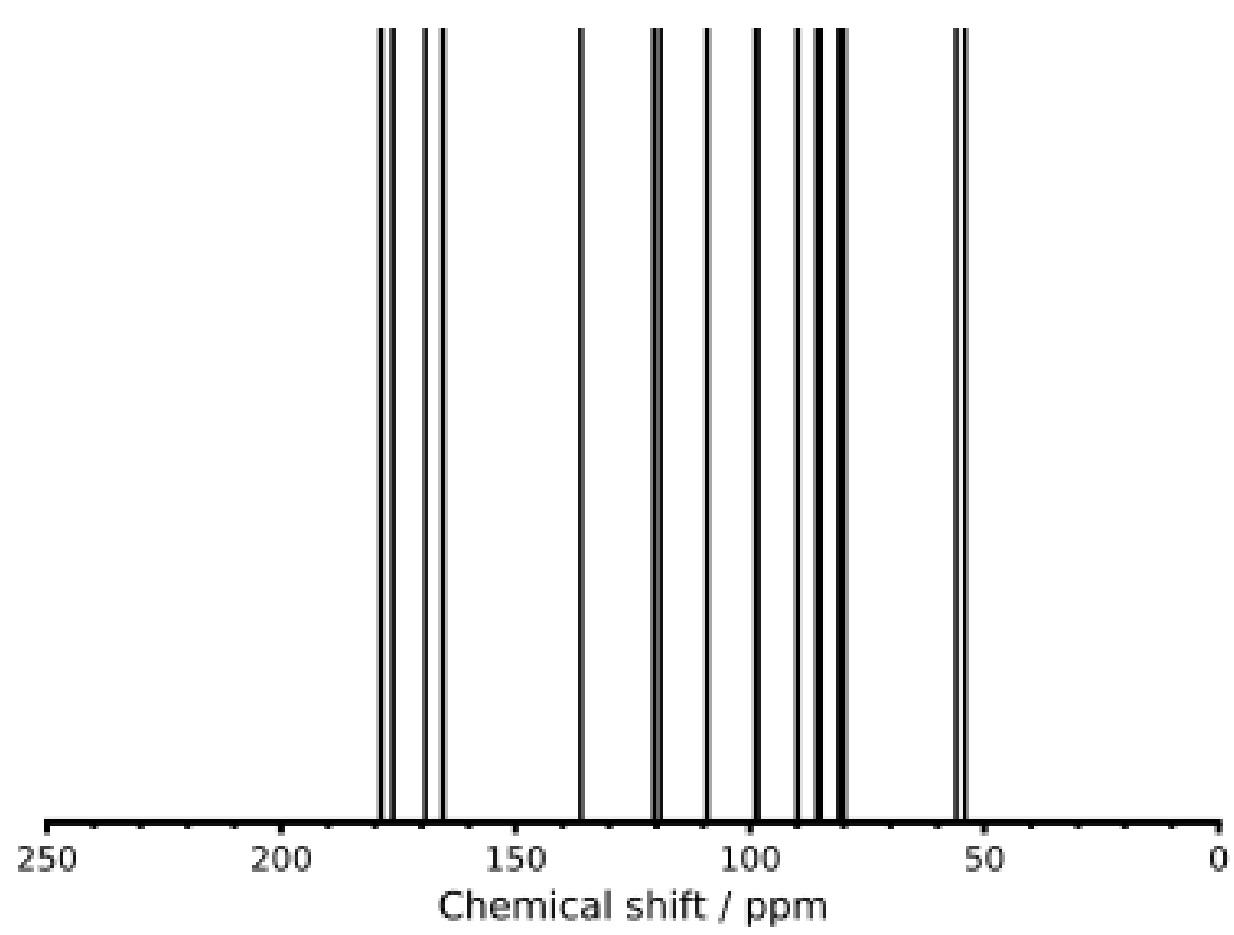
250
200
150
100
50
0
Chemical shift / ppm

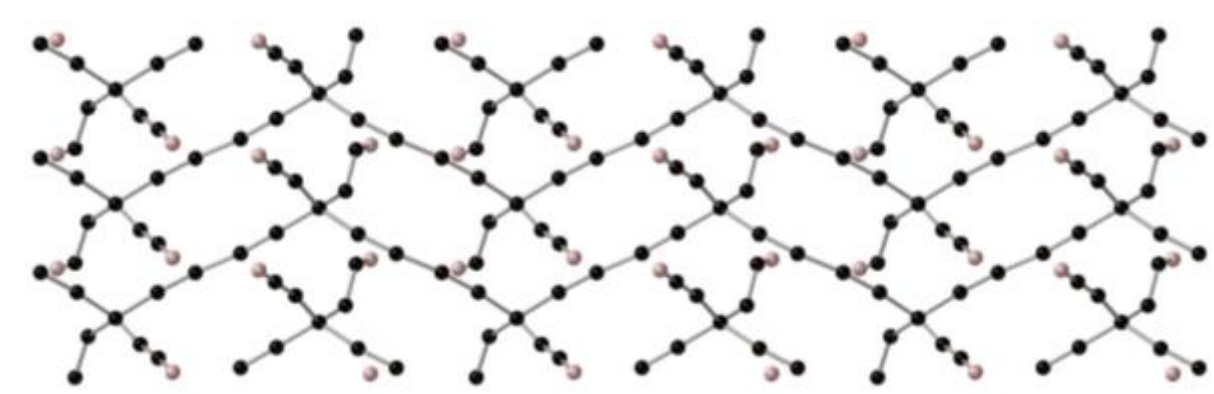

RS

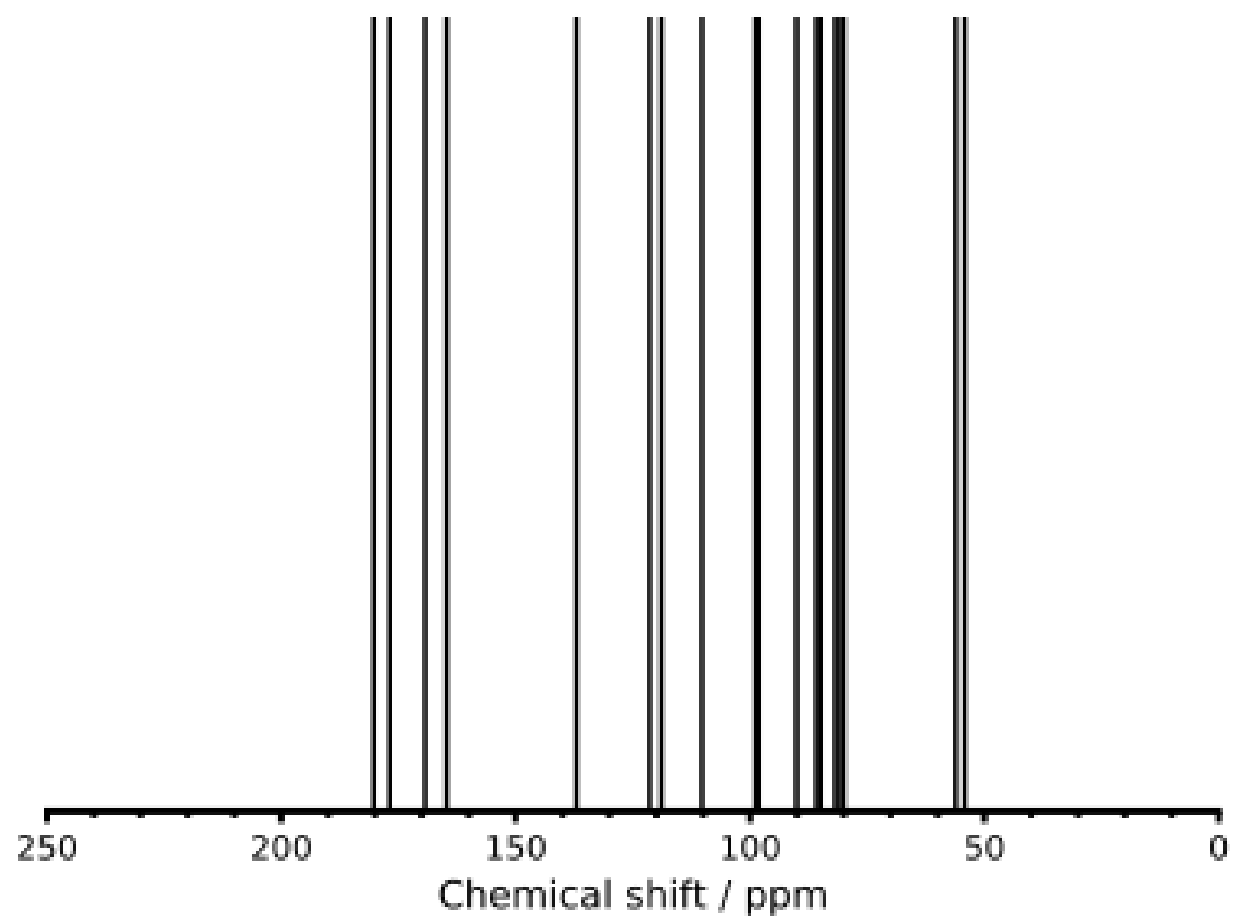
250
200
150
100
50
0
Chemical shift / ppm

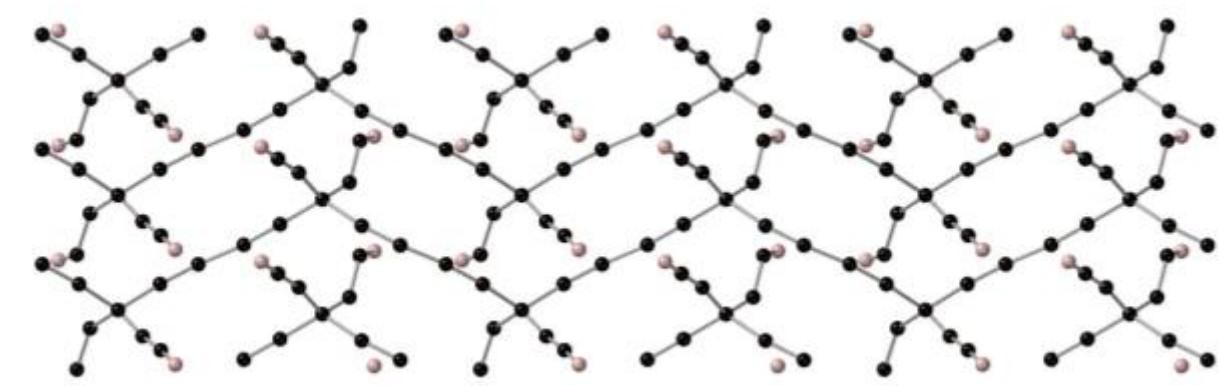

RT

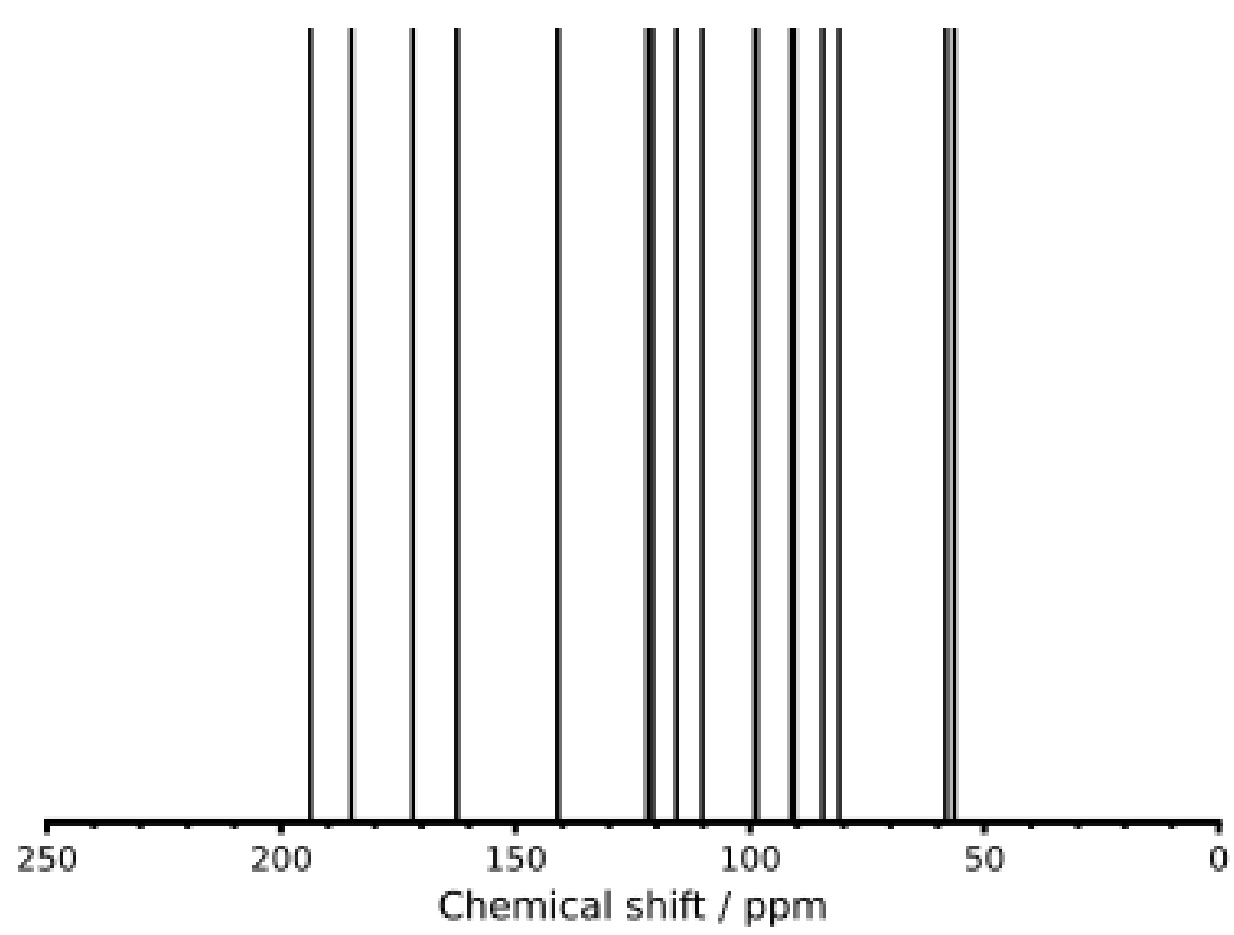
250
200
150
100
50
0
Chemical shift / ppm

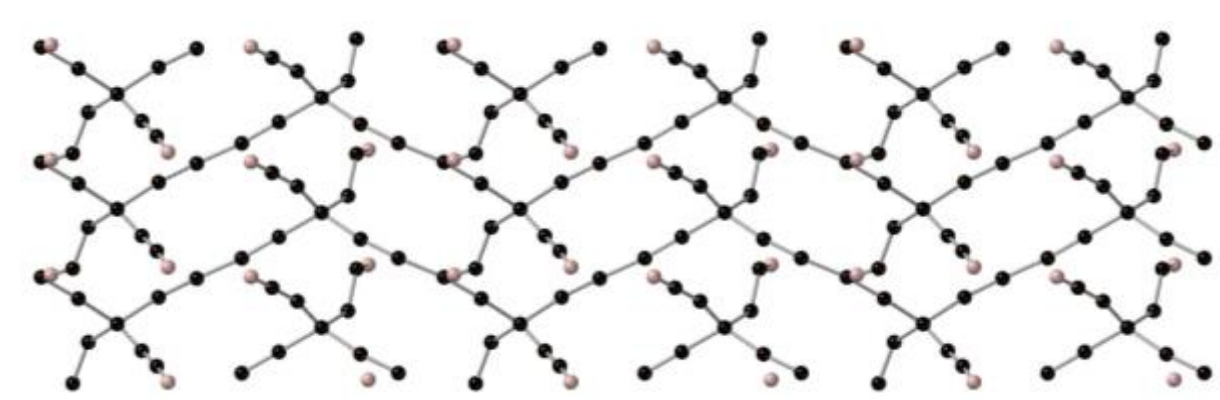

MT

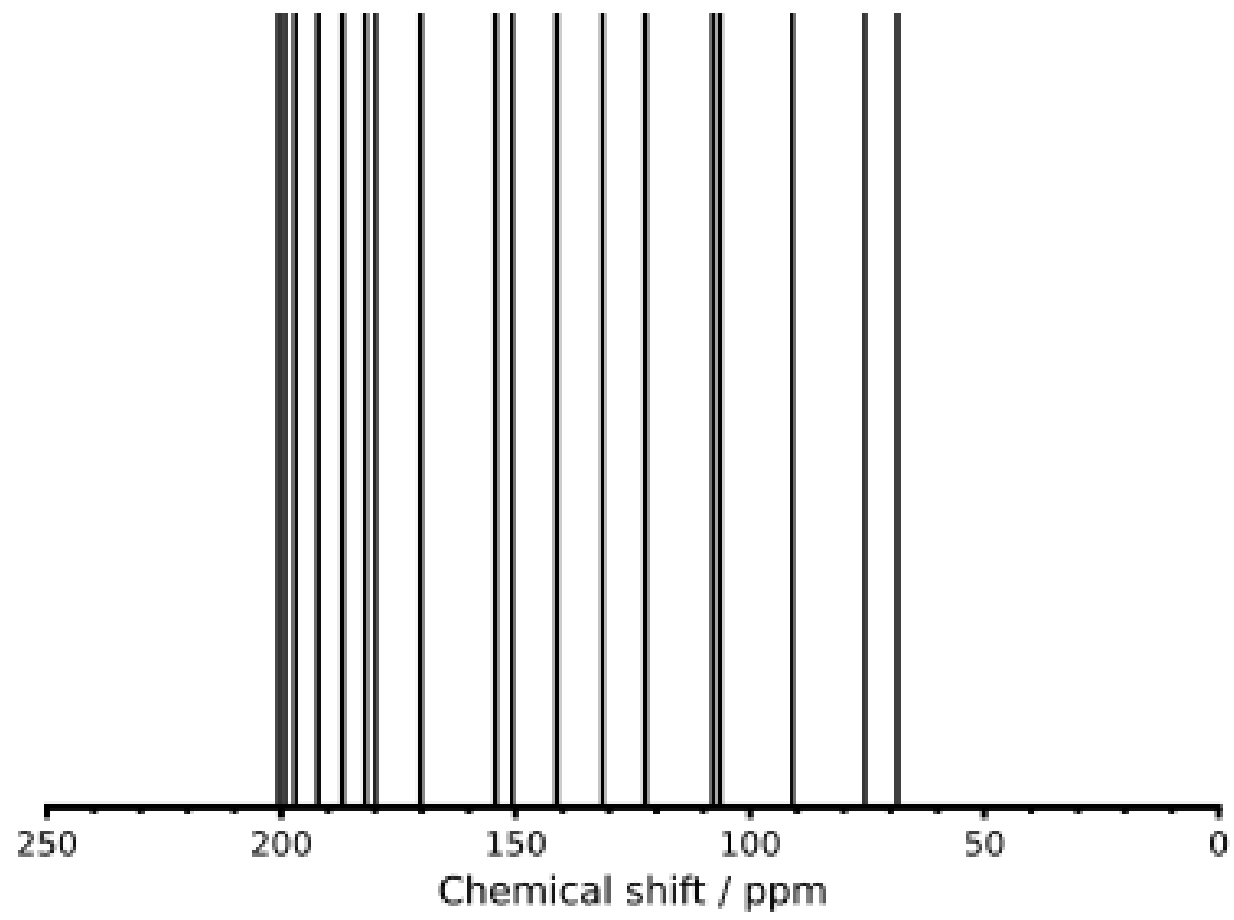
250
200
150
100
50
0
Chemical shift / ppm

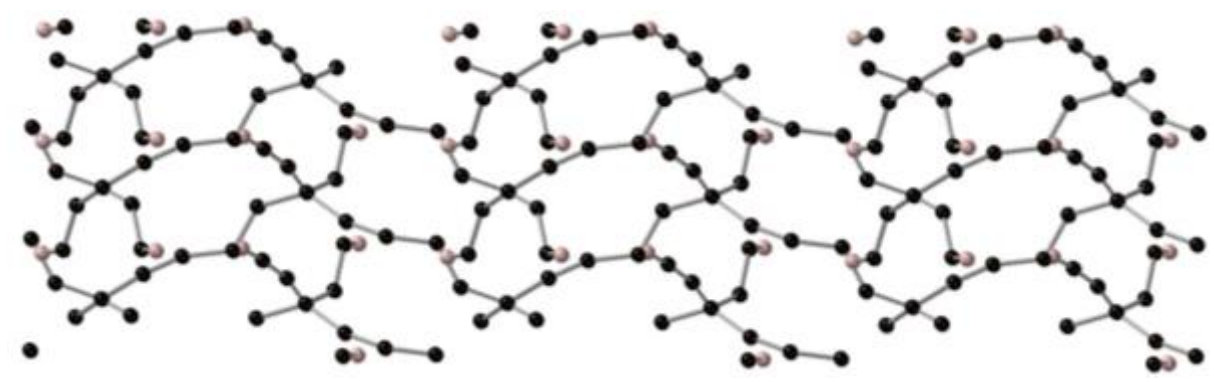

QB

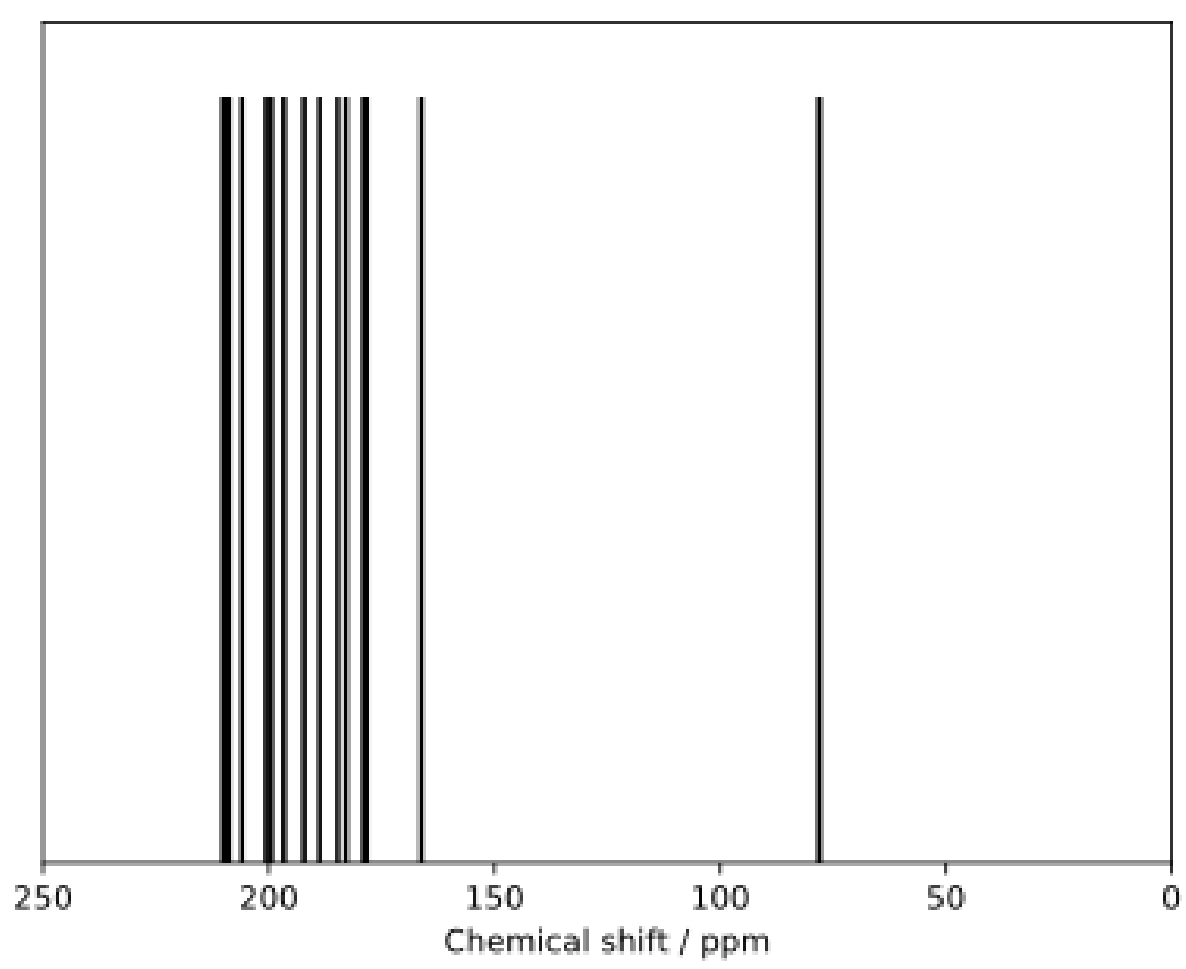
250
200
150
100
50
0
Chemical shift / ppm

QC

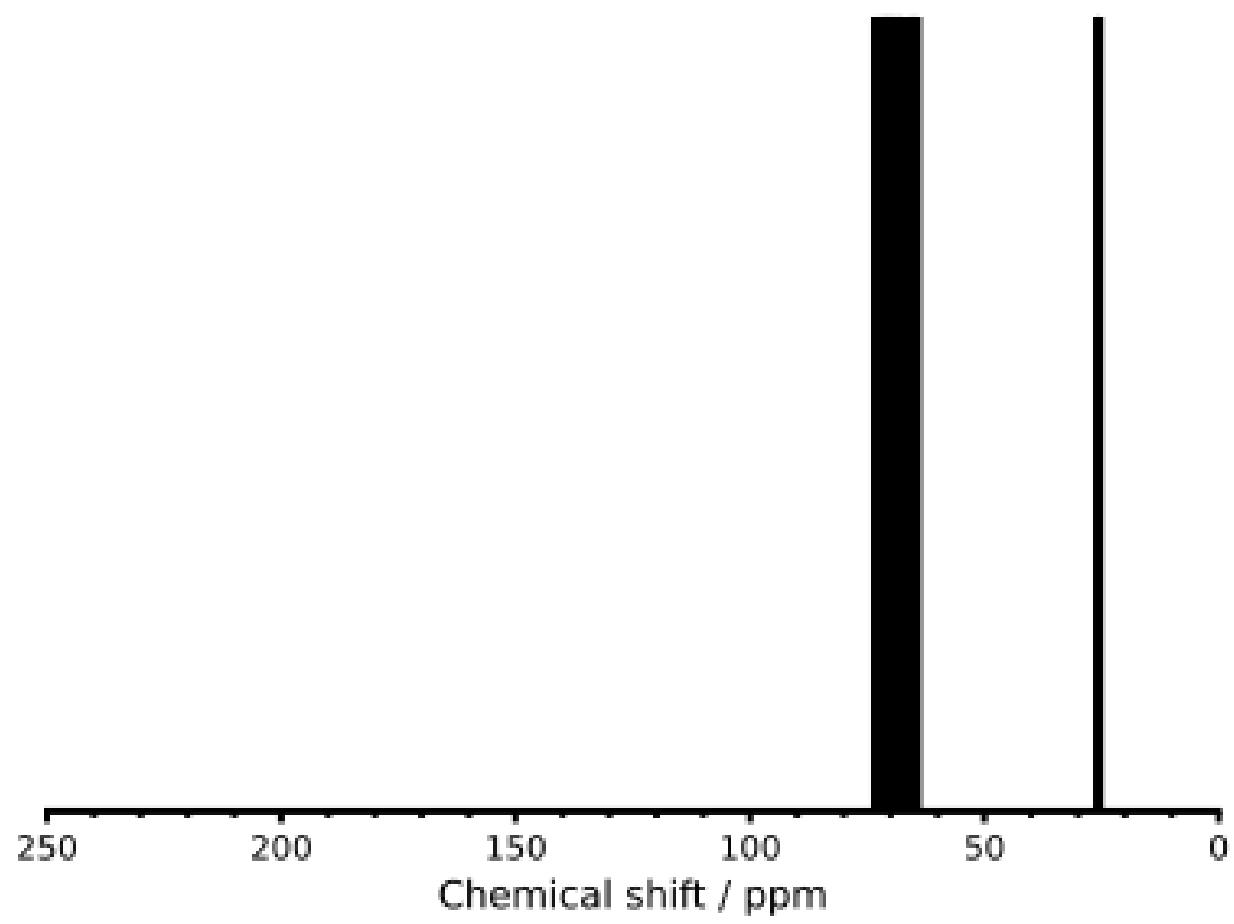
250
200
150
100
50
0
Chemical shift / ppm
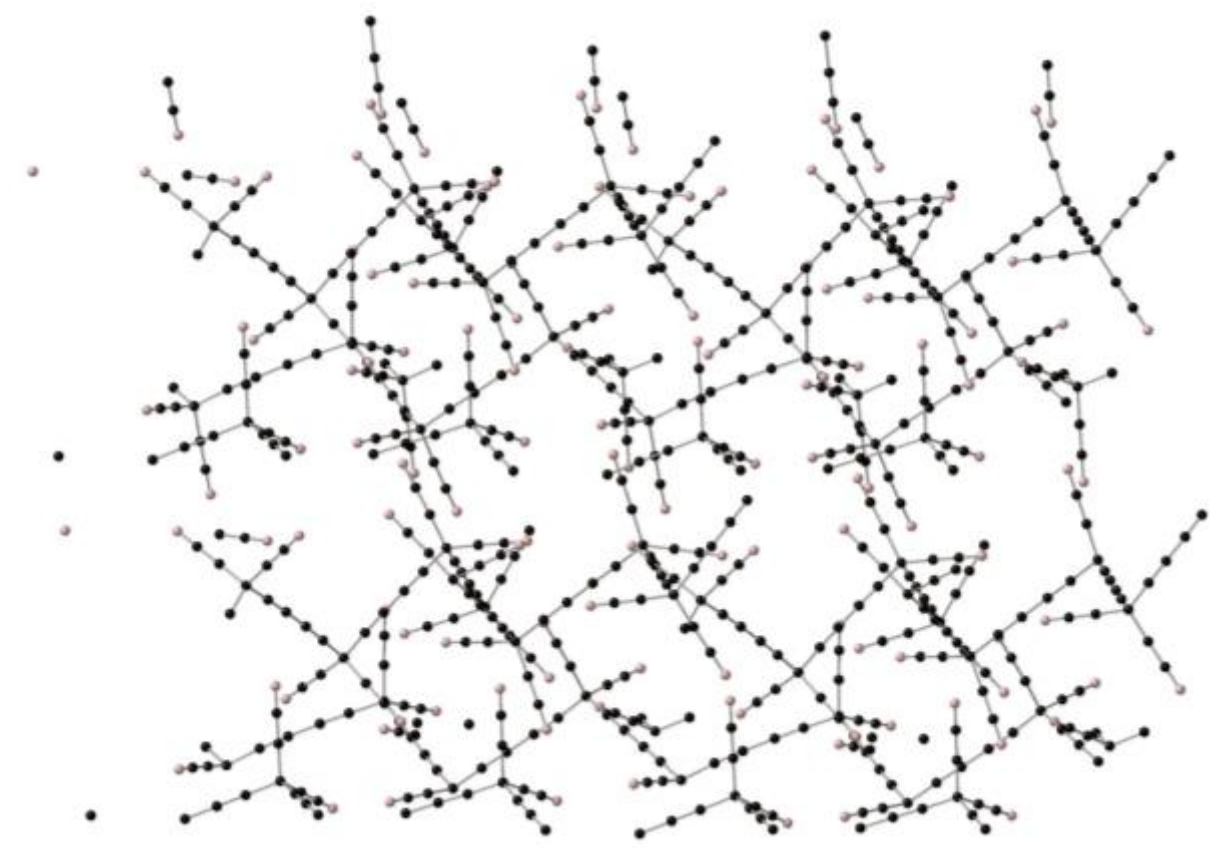

OB

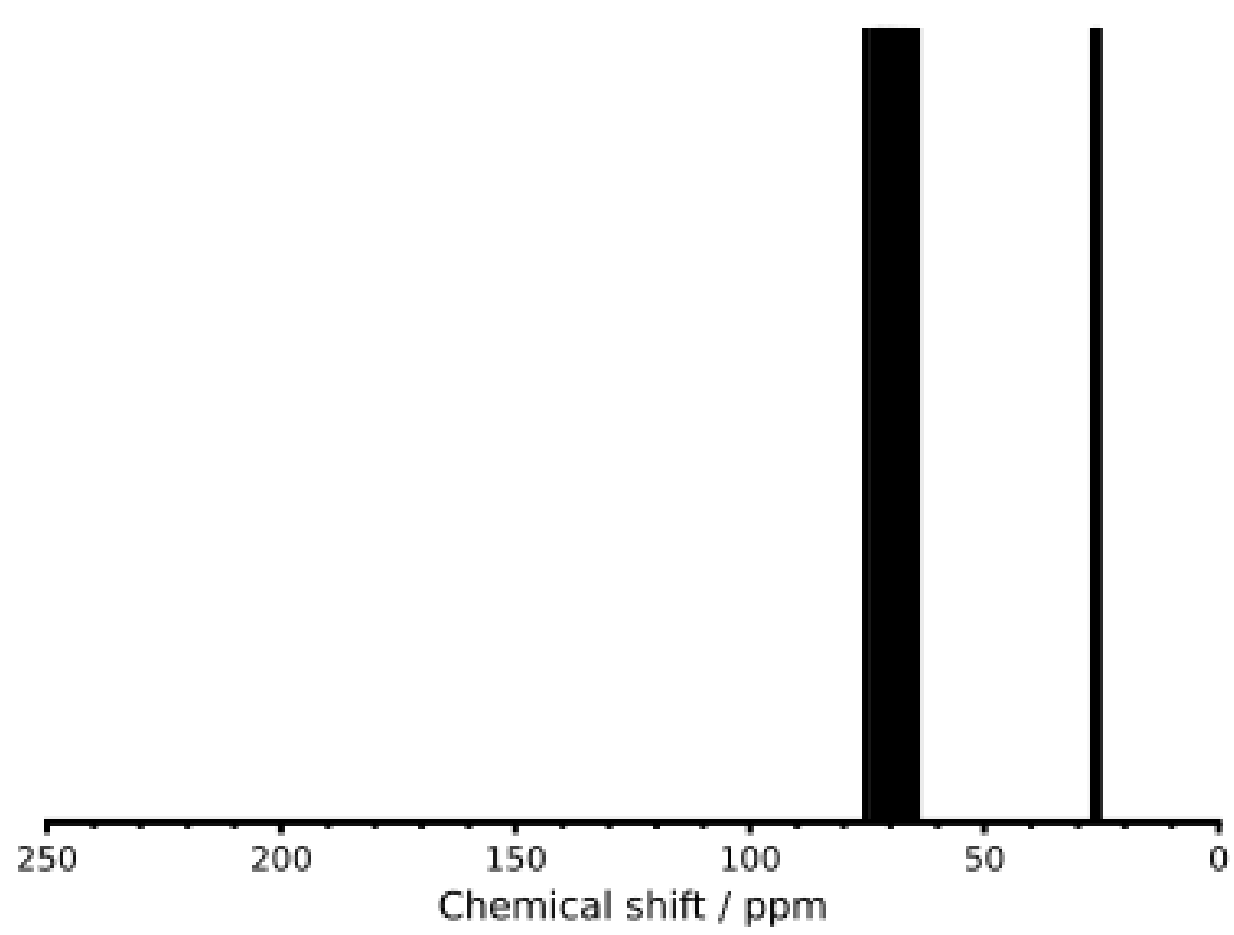
250
200
150
100
50
0
Chemical shift / ppm
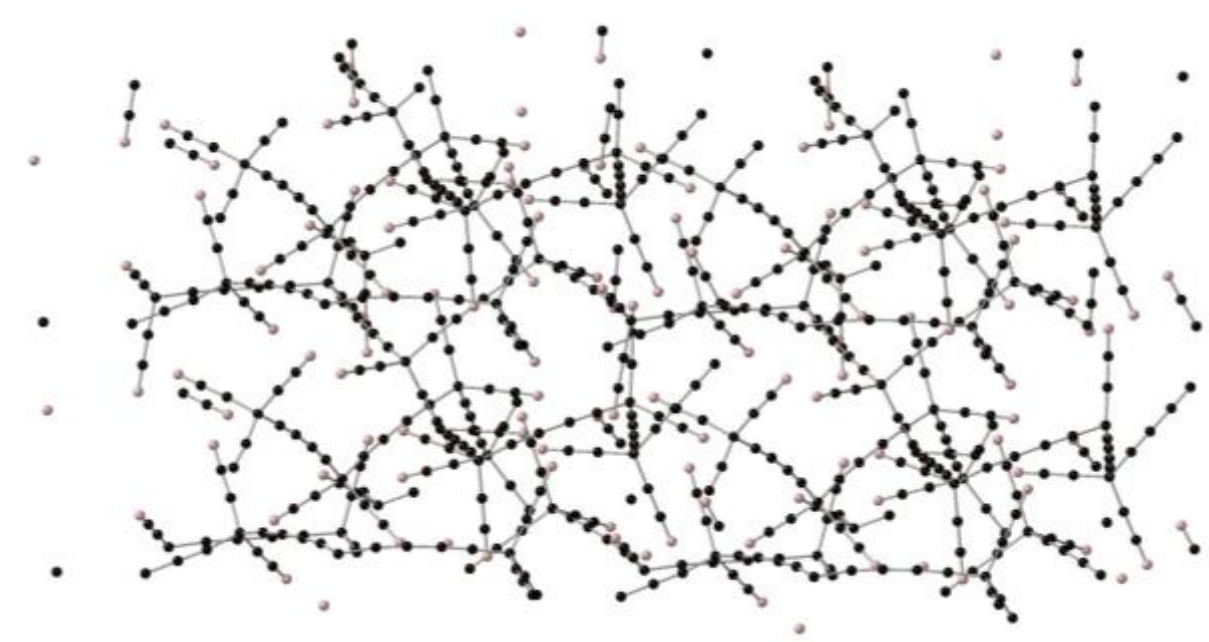

OR

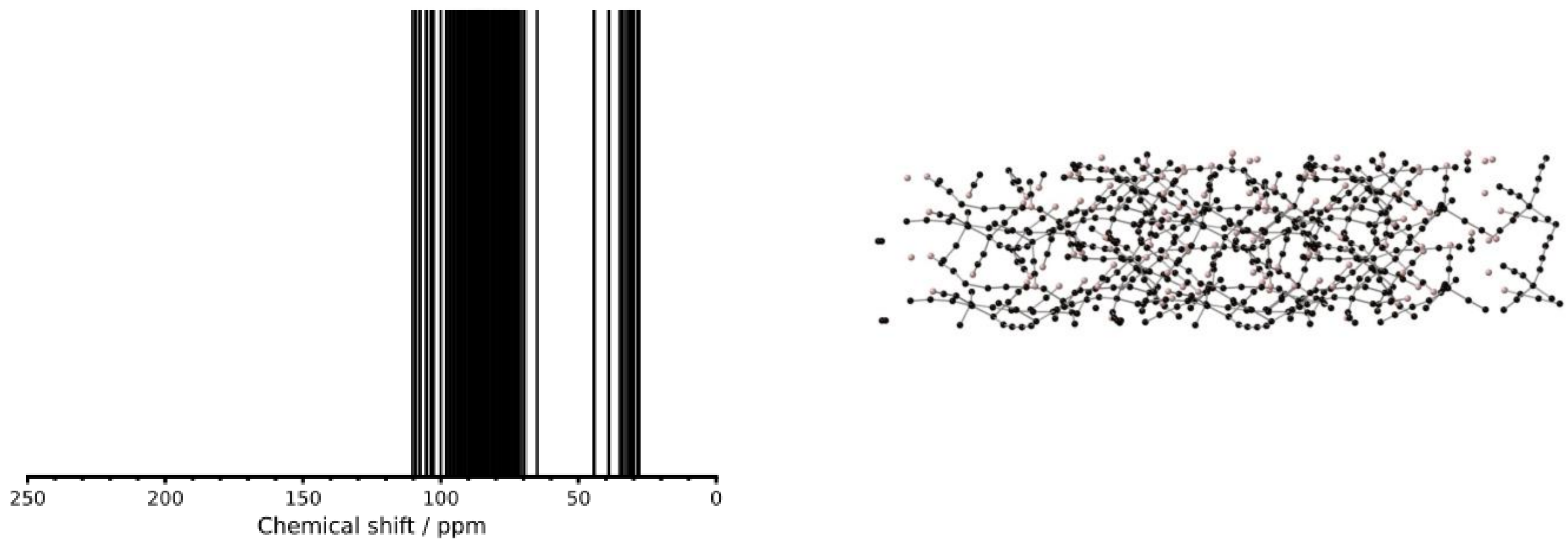
250
200
150
100
50
0
Chemical shift / ppm

PB

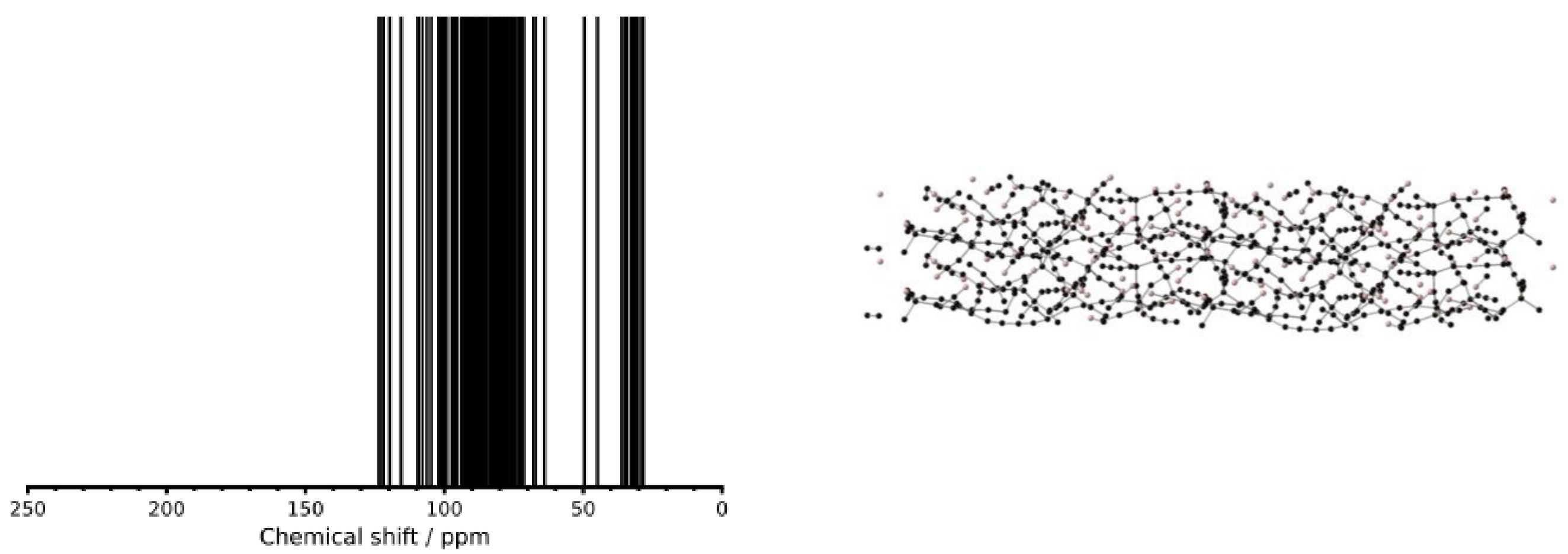
250
200
150
100
50
0
Chemical shift / ppm

PN

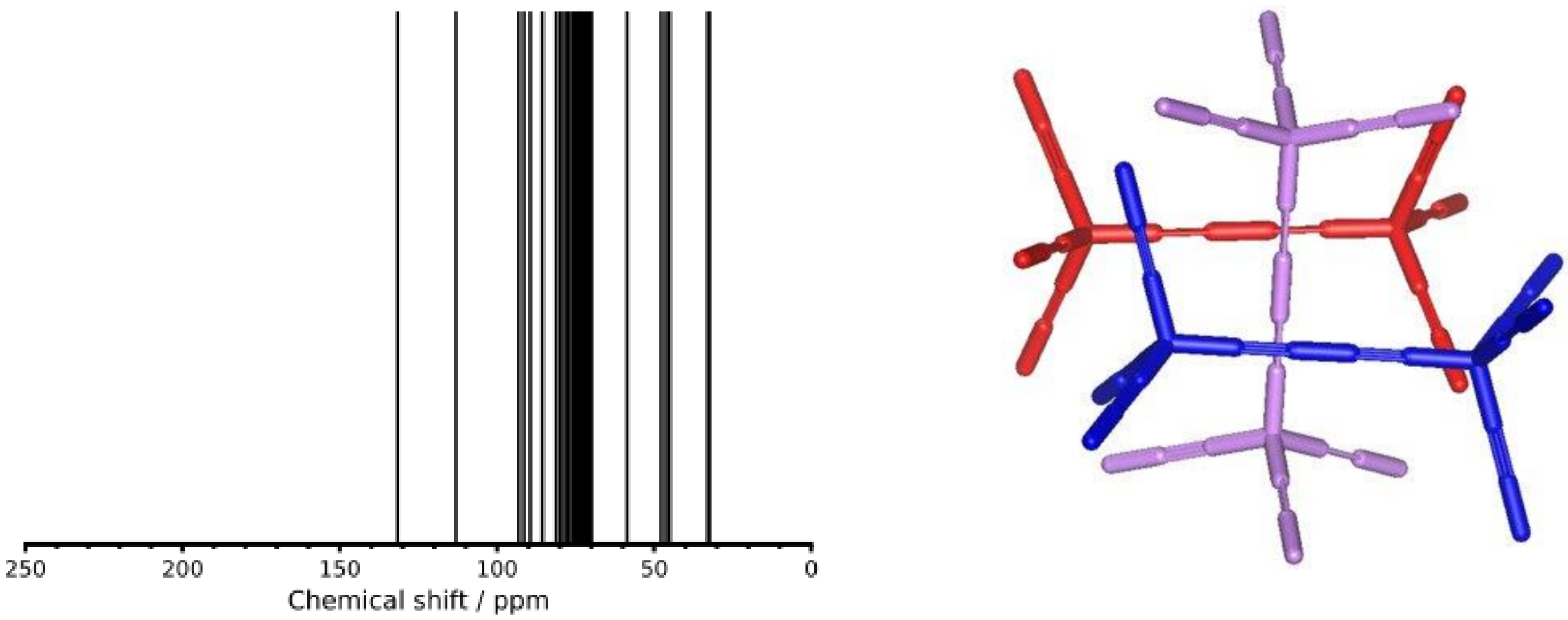
250
200
150
100
50
0
Chemical shift / ppm

a

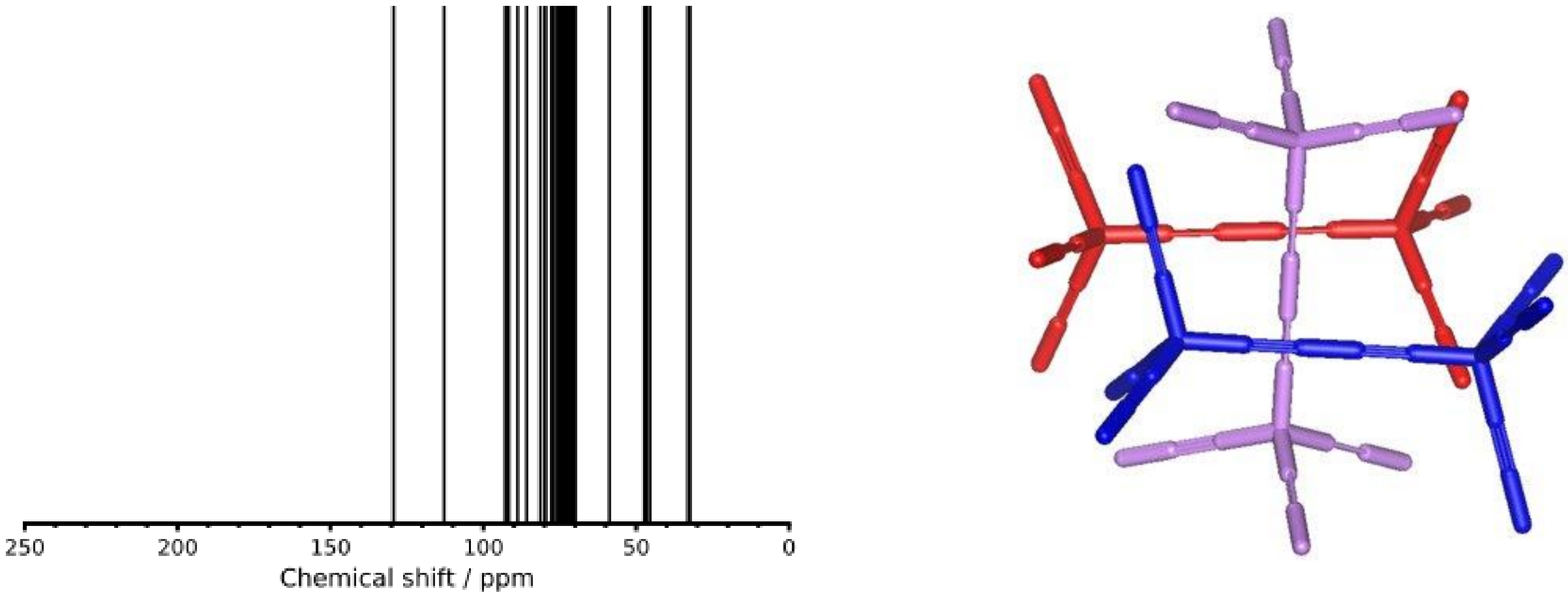
250
200
150
100
50
0
Chemical shift / ppm

b

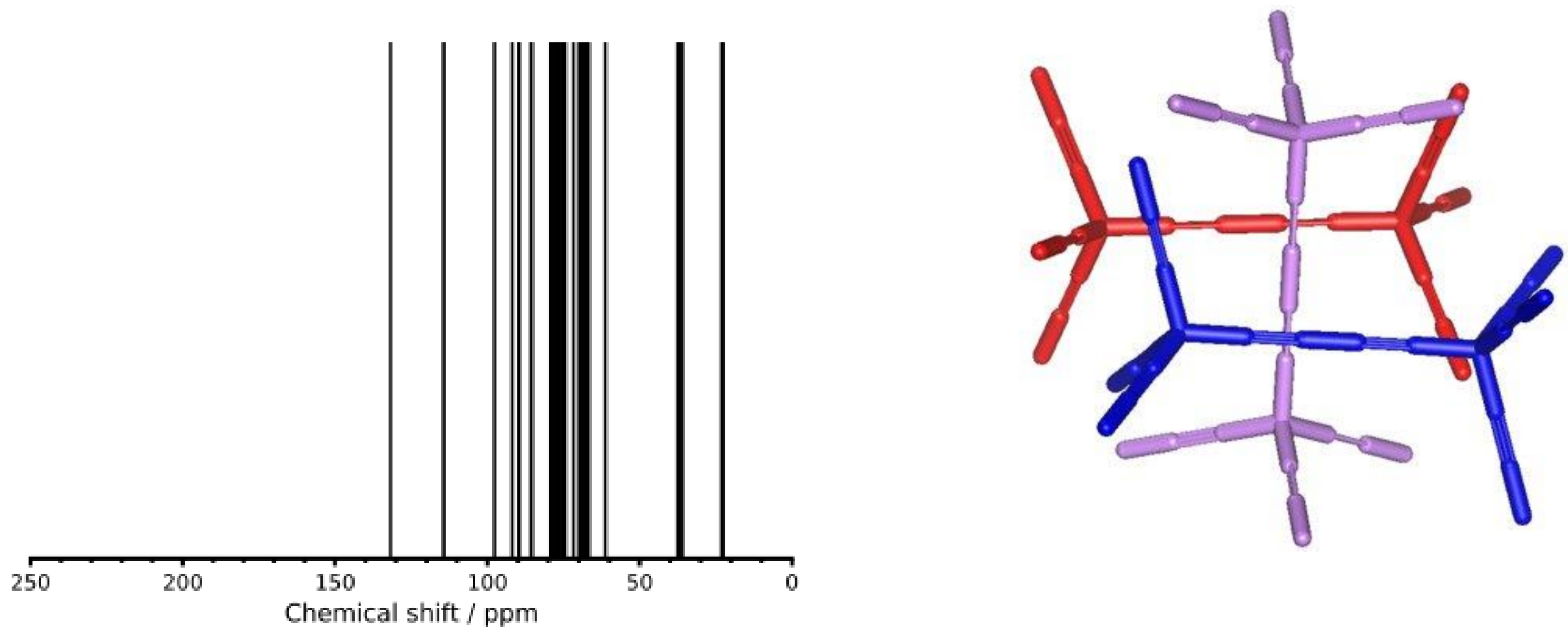
250
200
150
100
50
0
Chemical shift / ppm

c

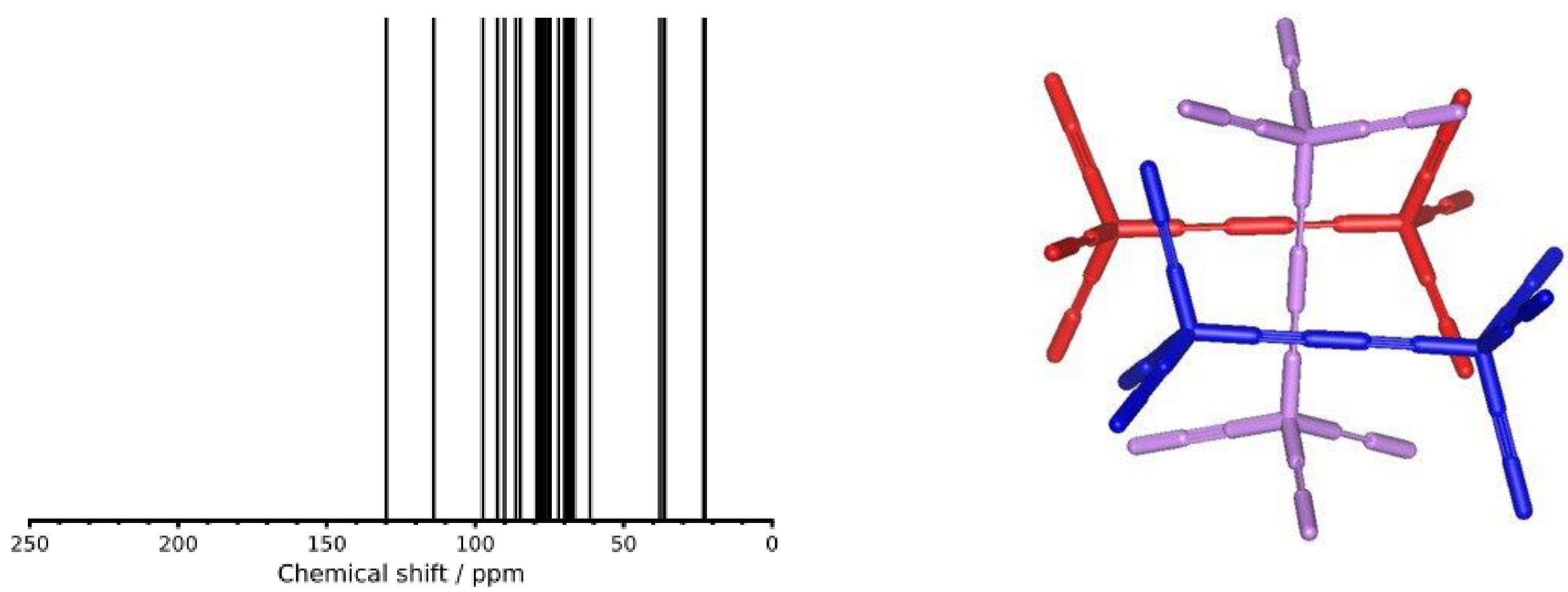
250
200
150
100
50
0
Chemical shift / ppm

d

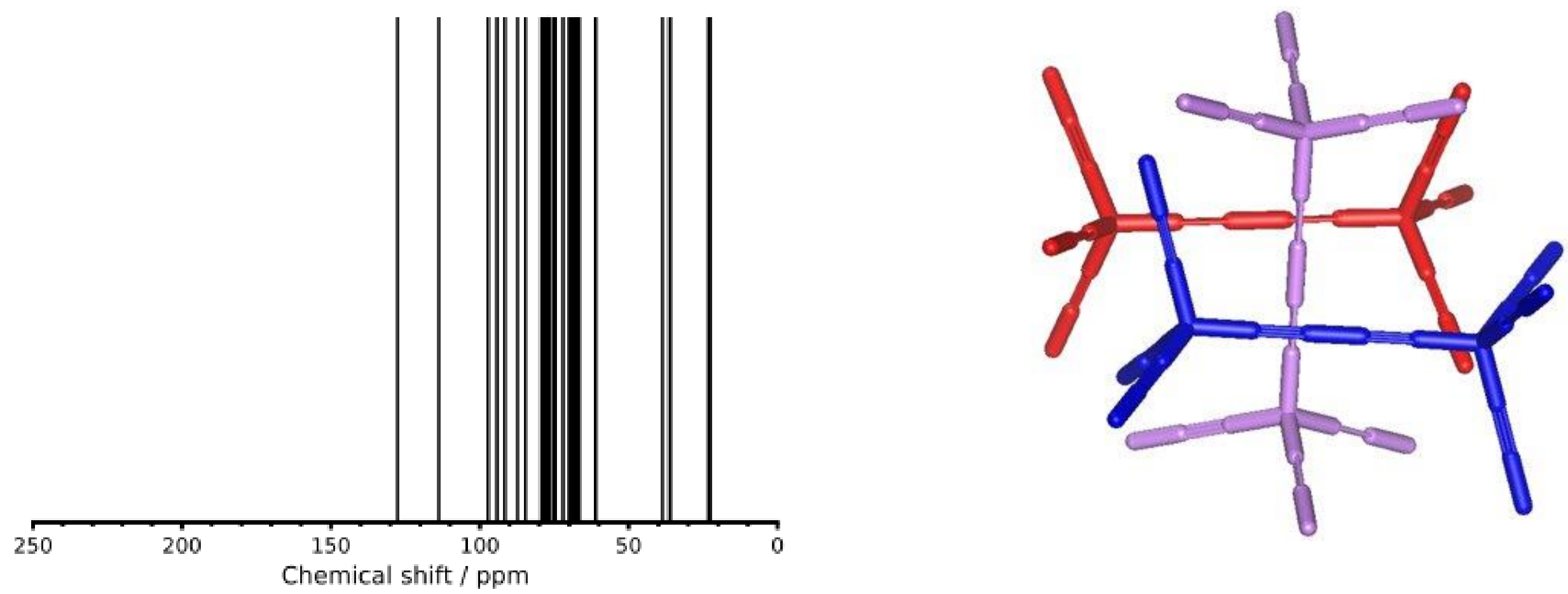
250 200 150 100 50 0
Chemical shift / ppm

e

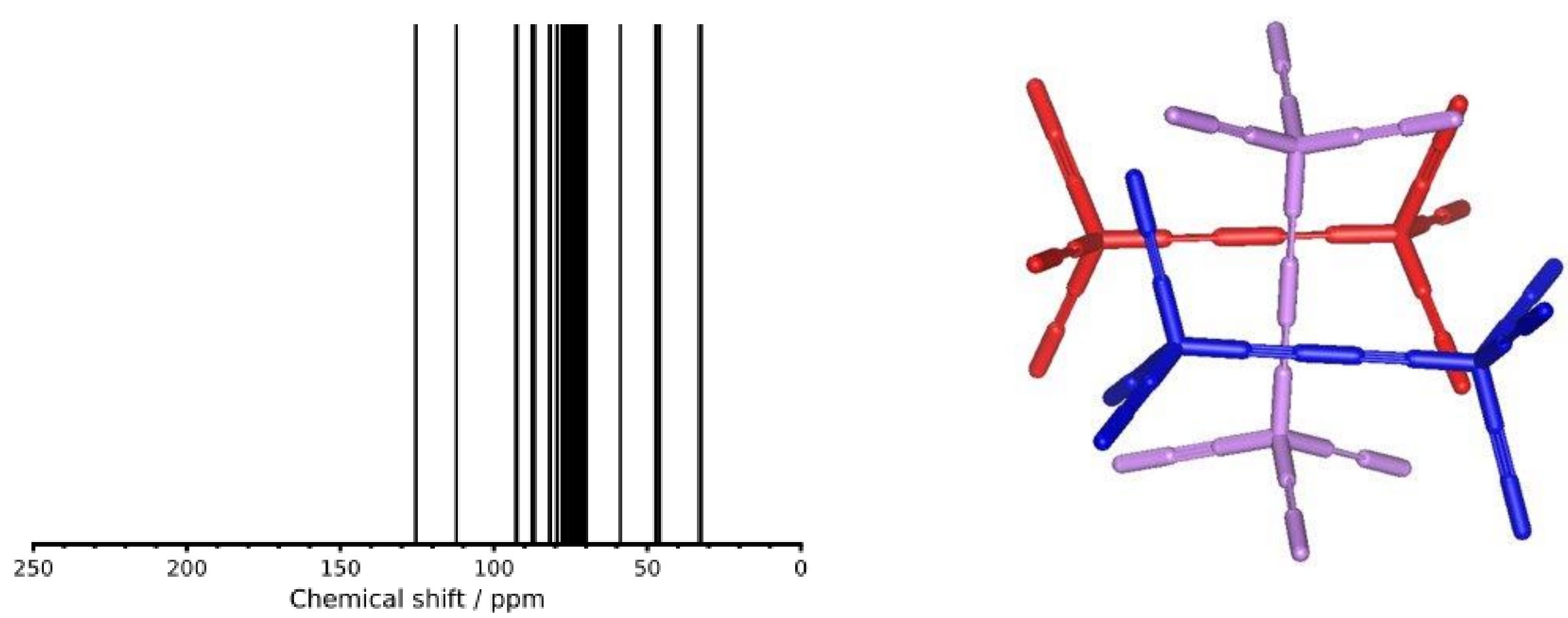
250 200 150 100 50 0
Chemical shift / ppm

f

OSPC-1 spectra with 2xBr end groups

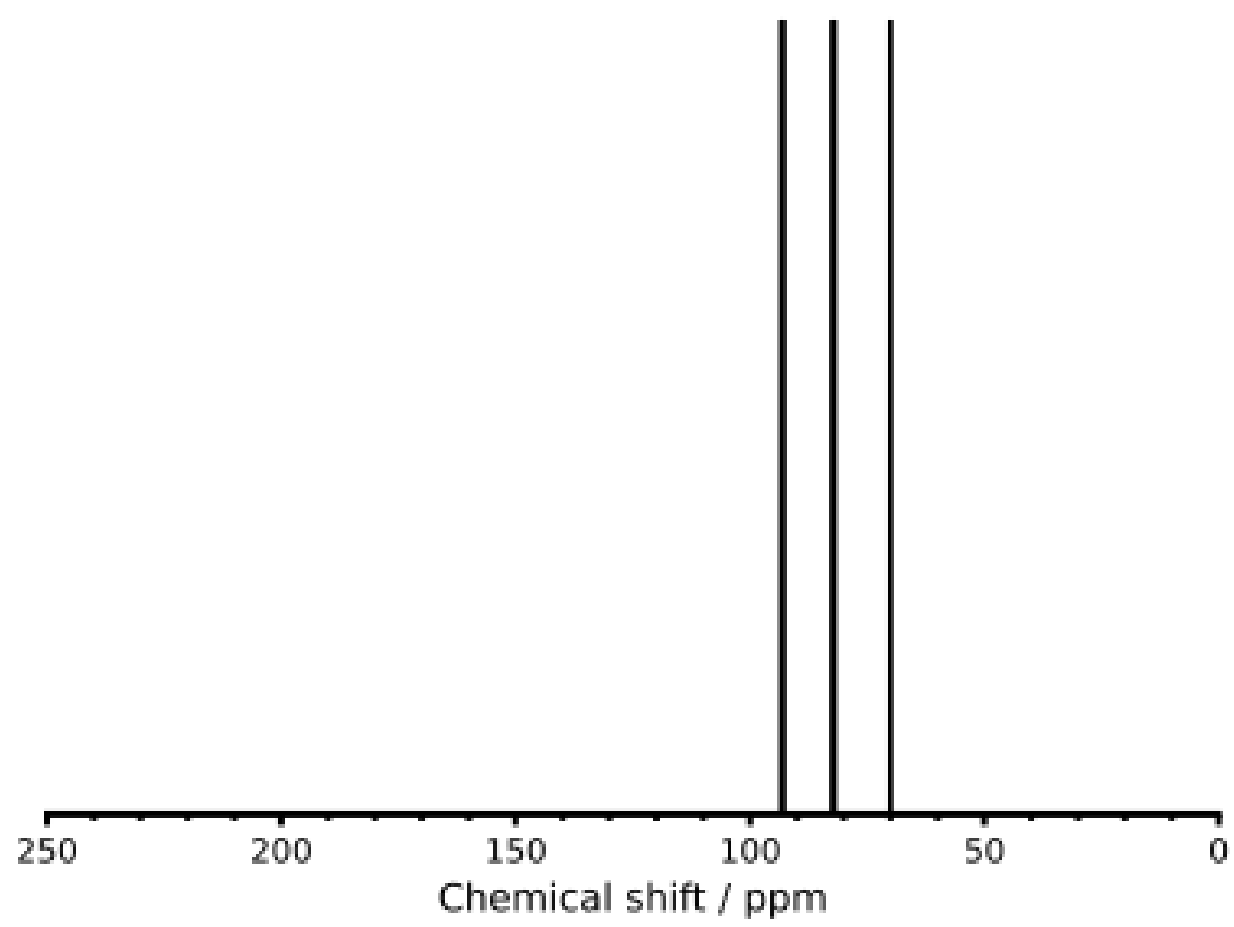

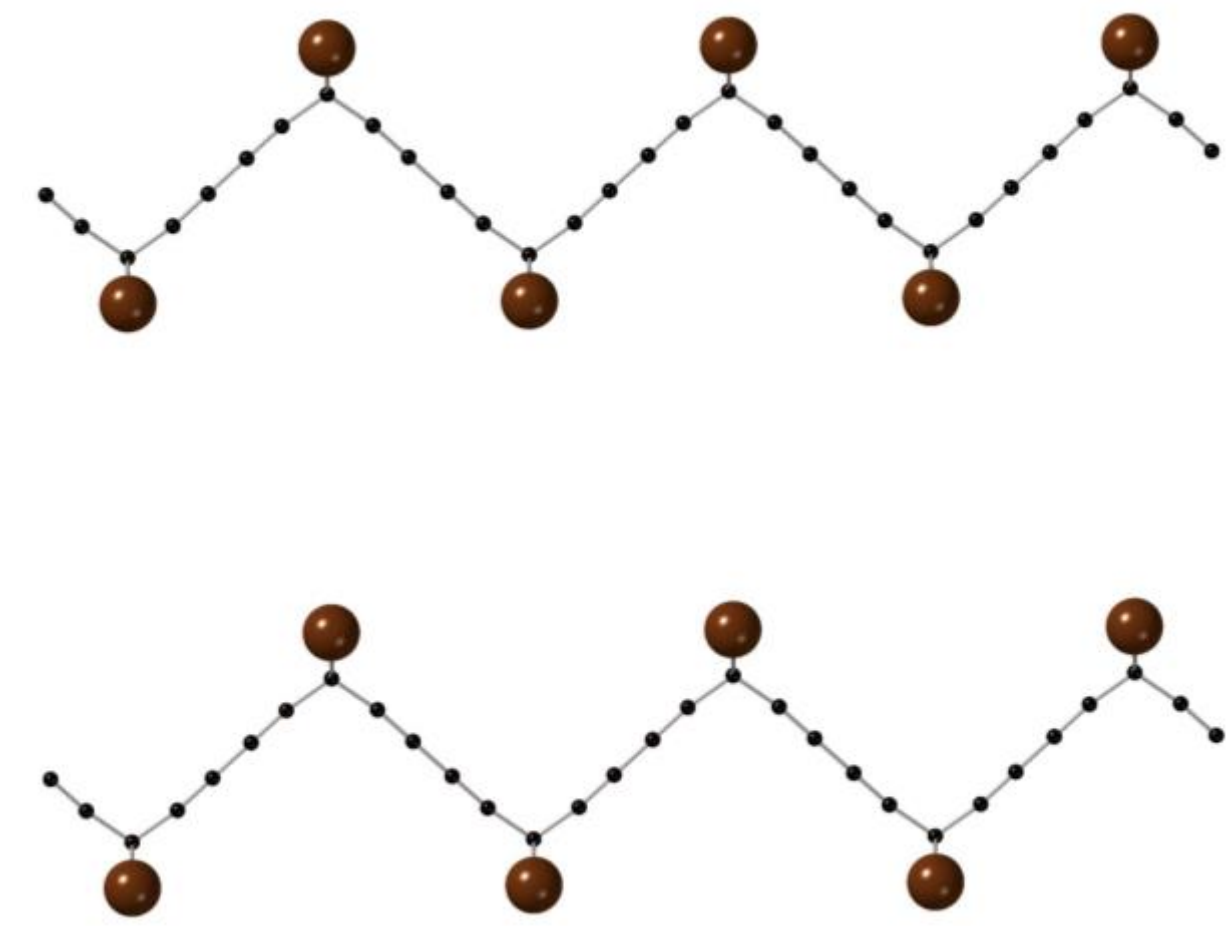

AT

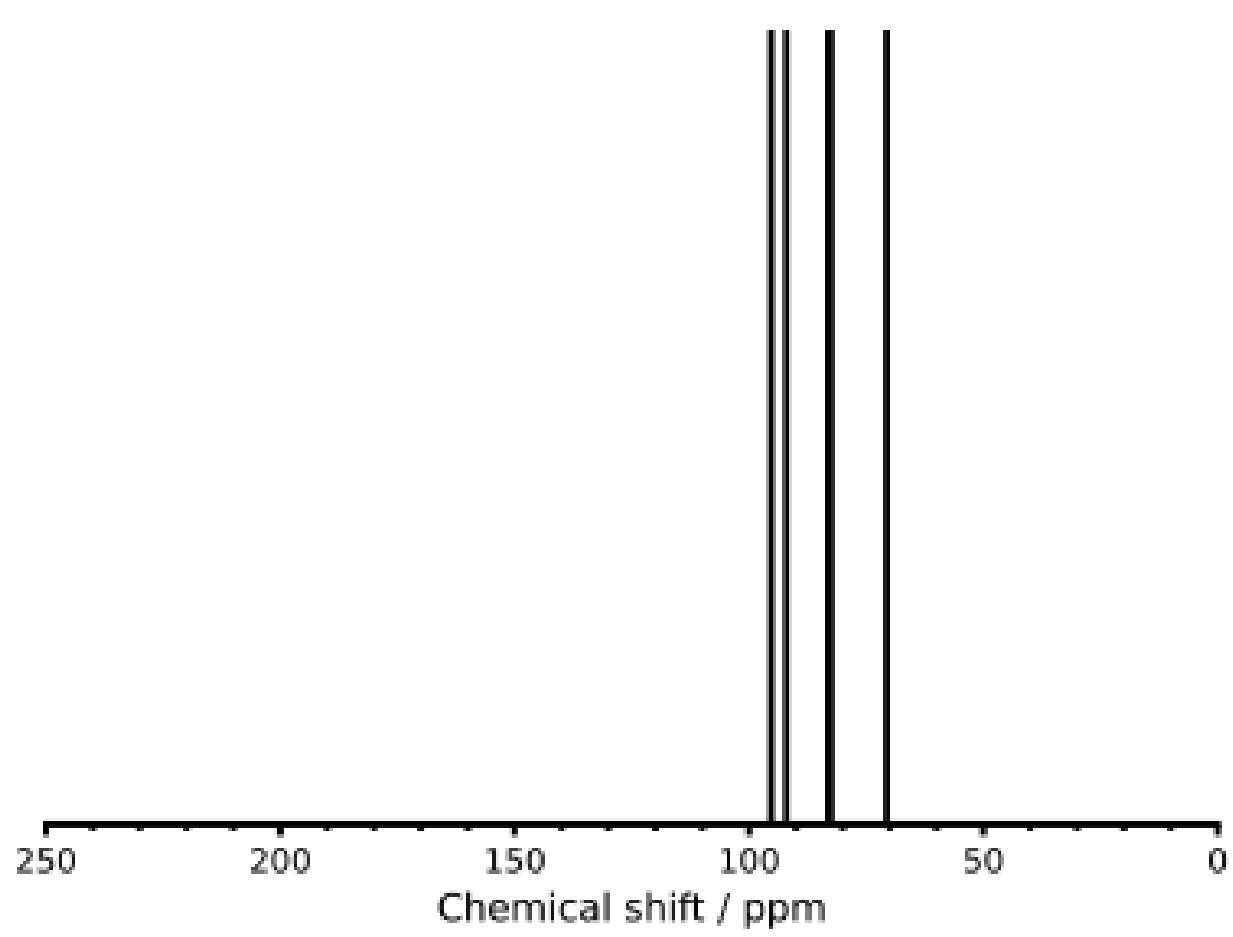

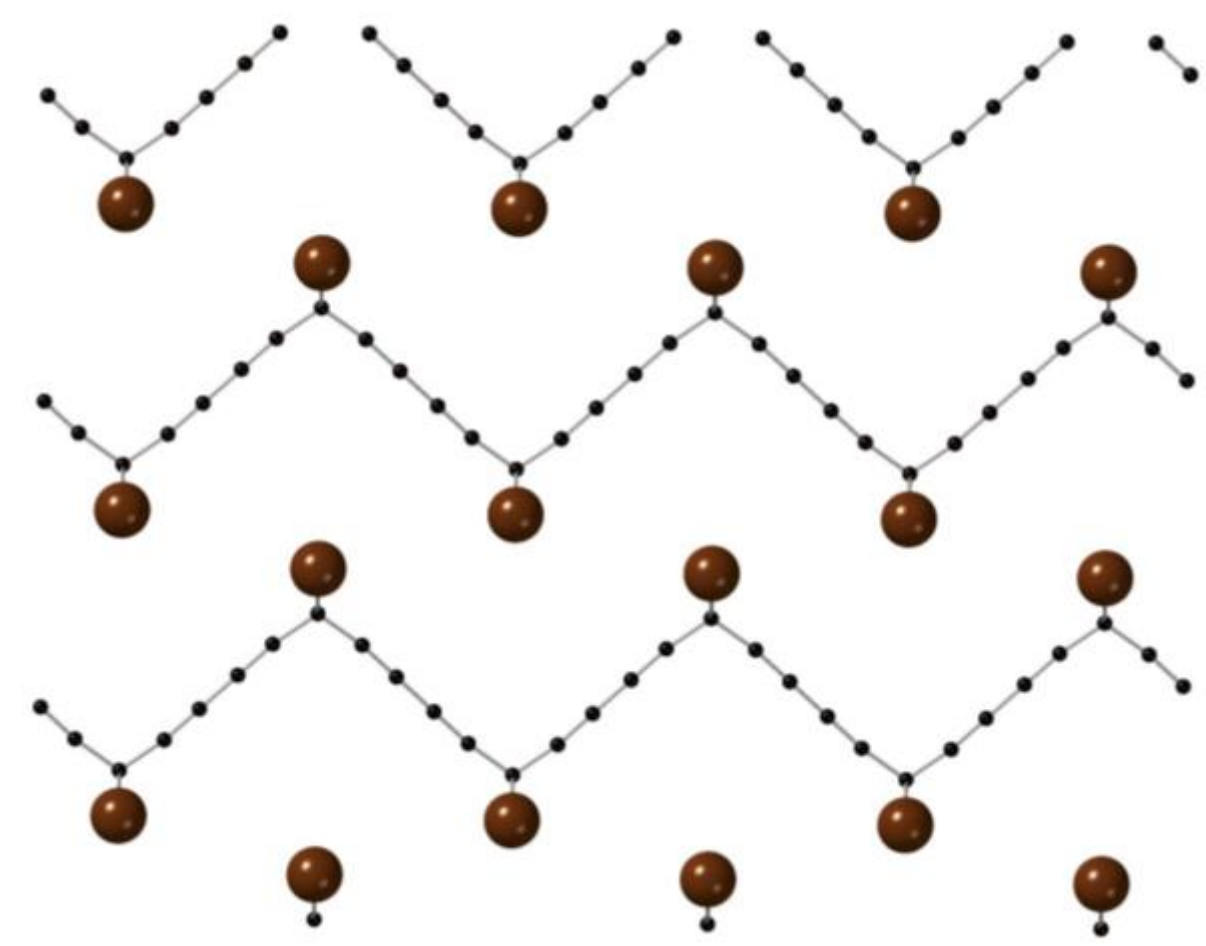

BA

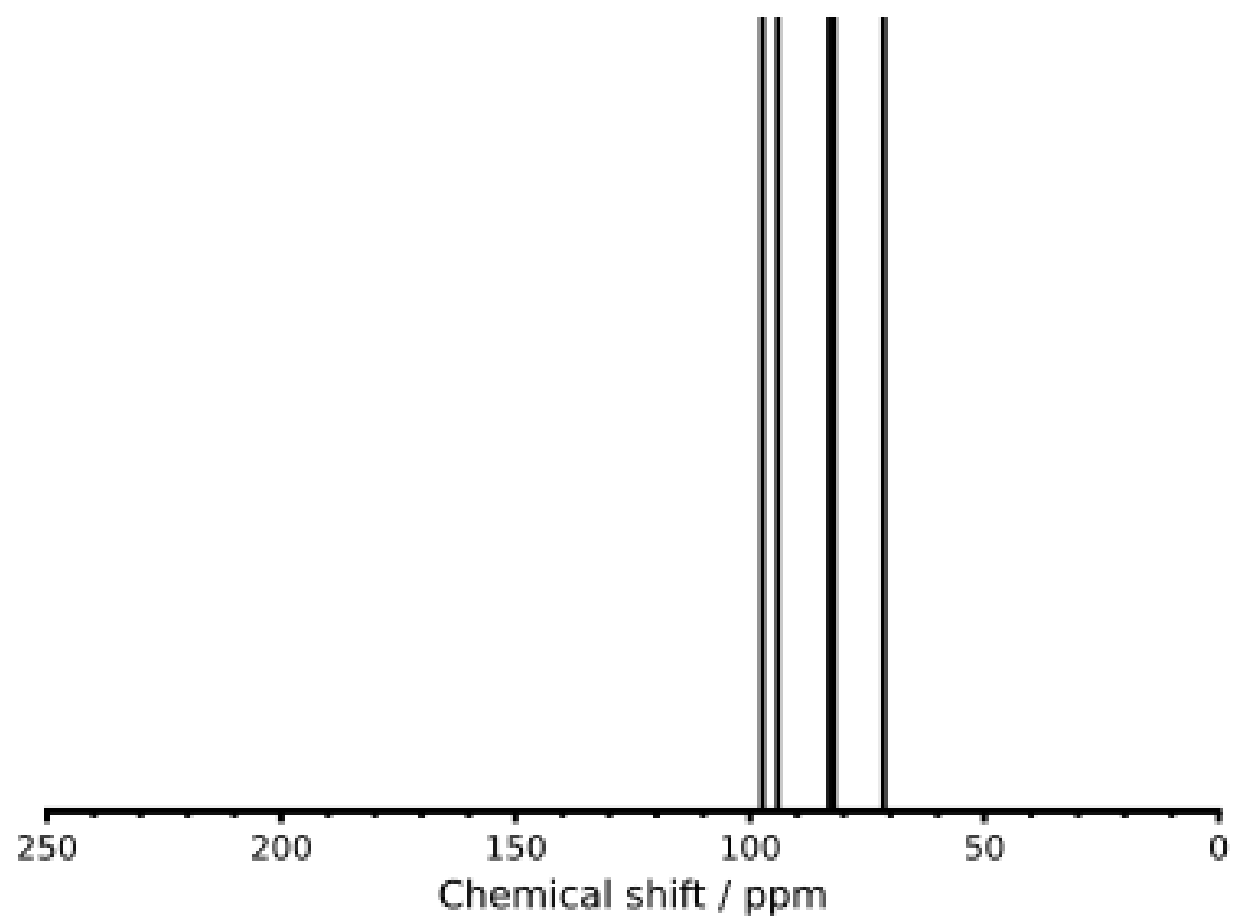
250
200
150
100
50
0
Chemical shift / ppm

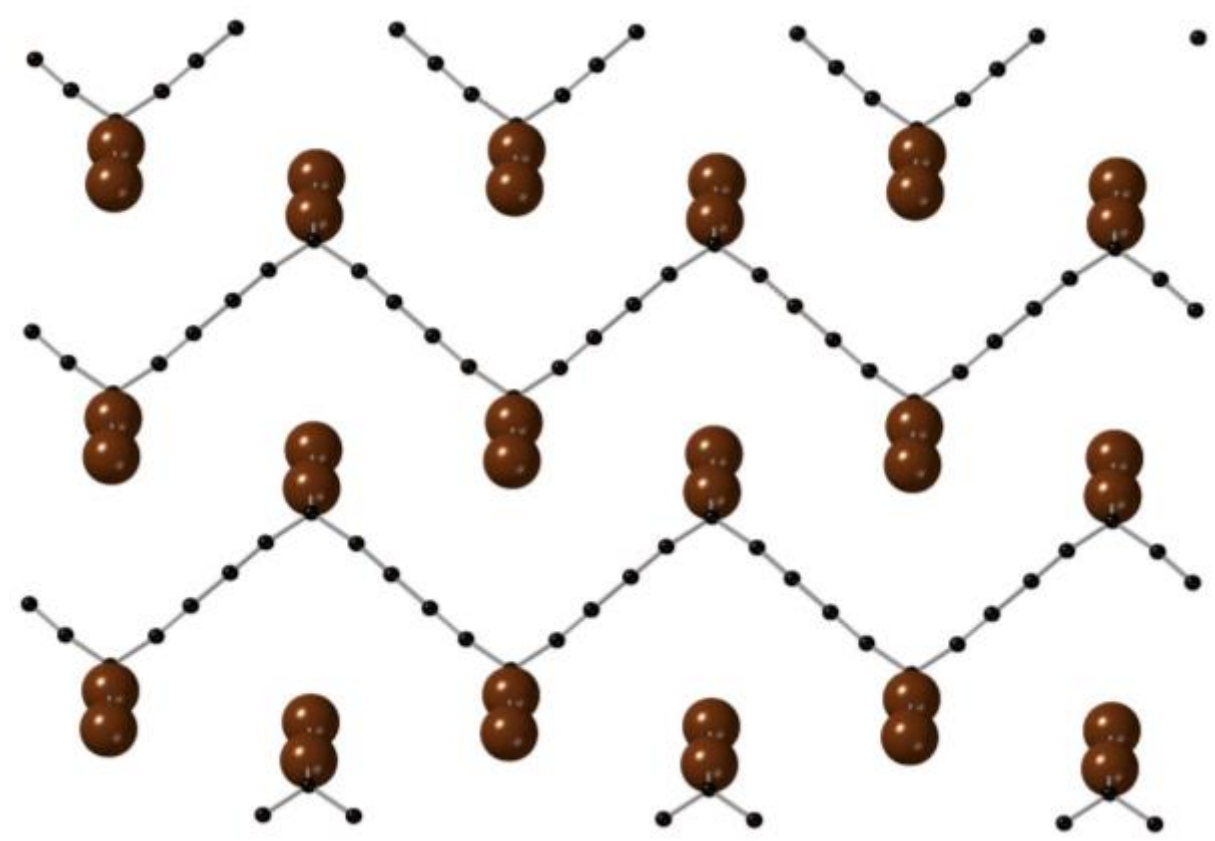

BB

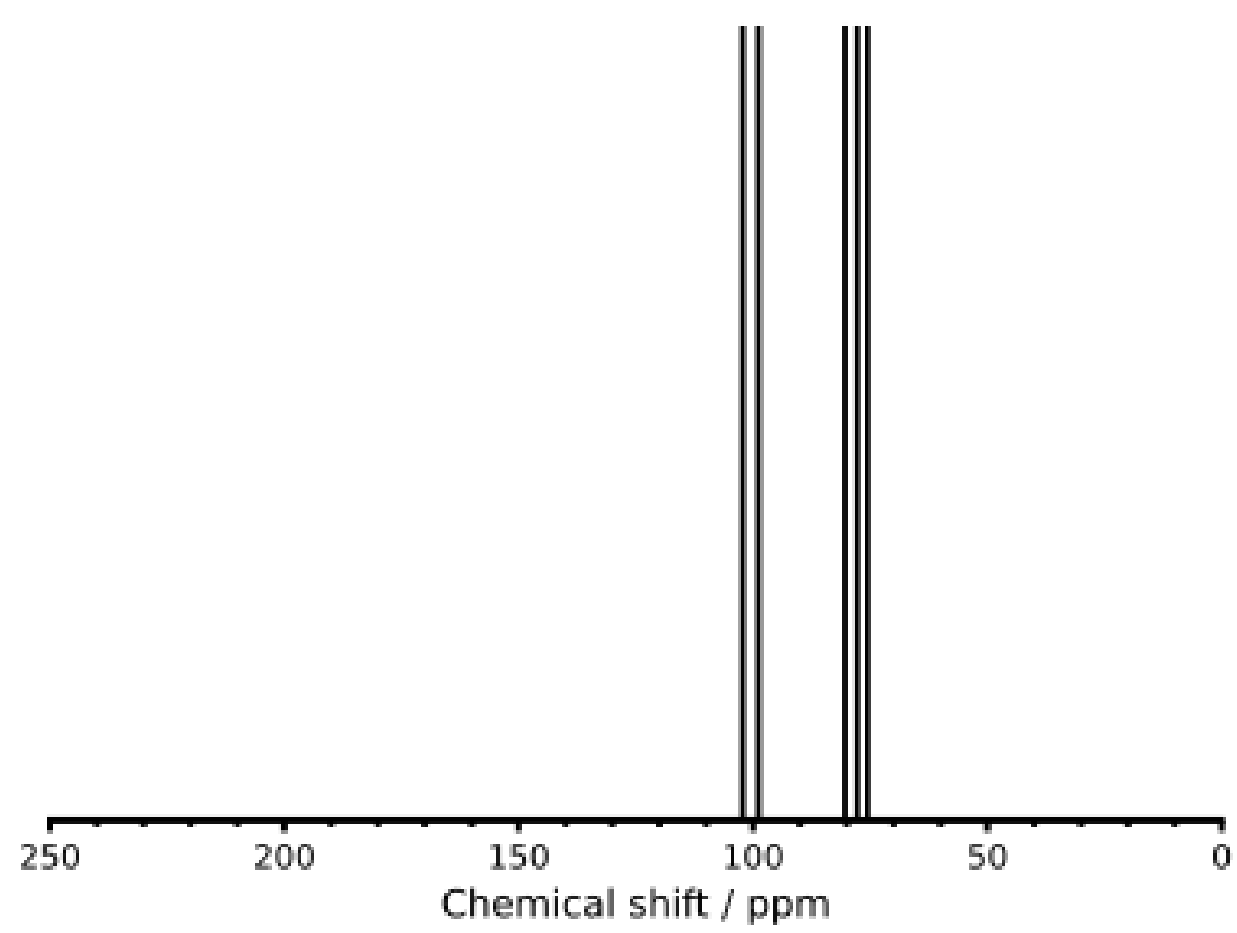
250
200
150
100
50
0
Chemical shift / ppm

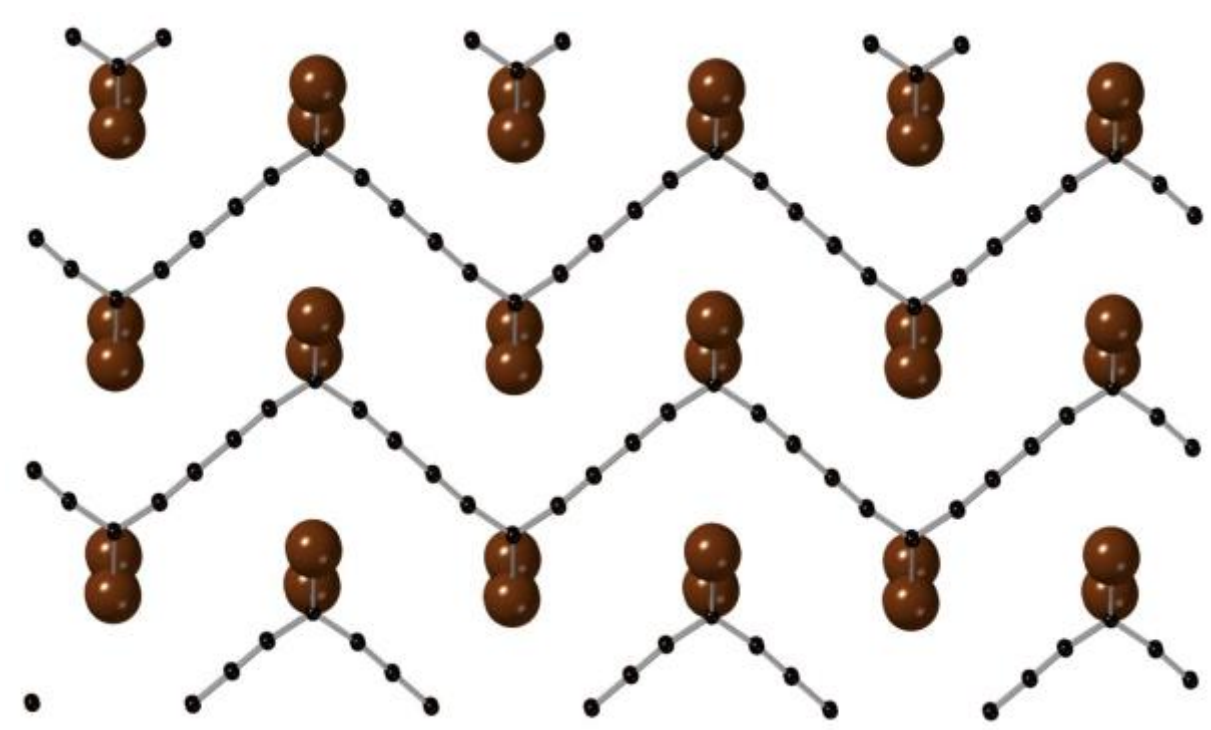

BC

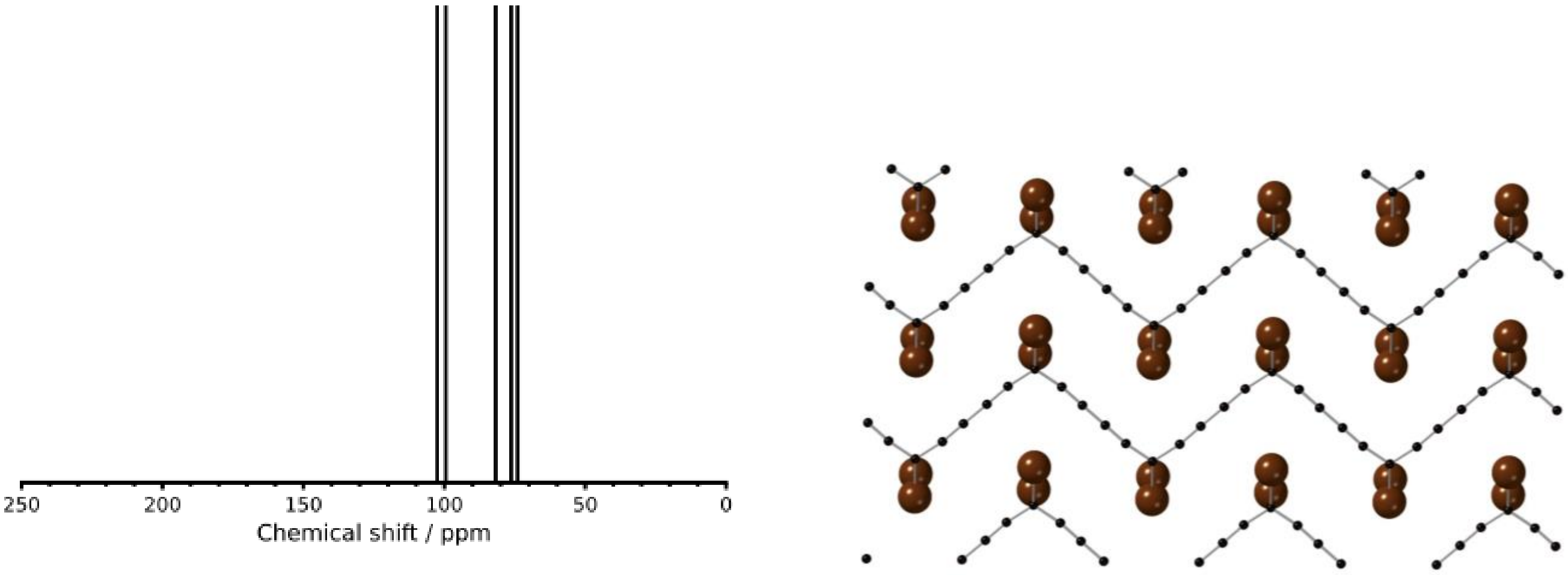
250 200 150 100 50 0
Chemical shift / ppm

BD

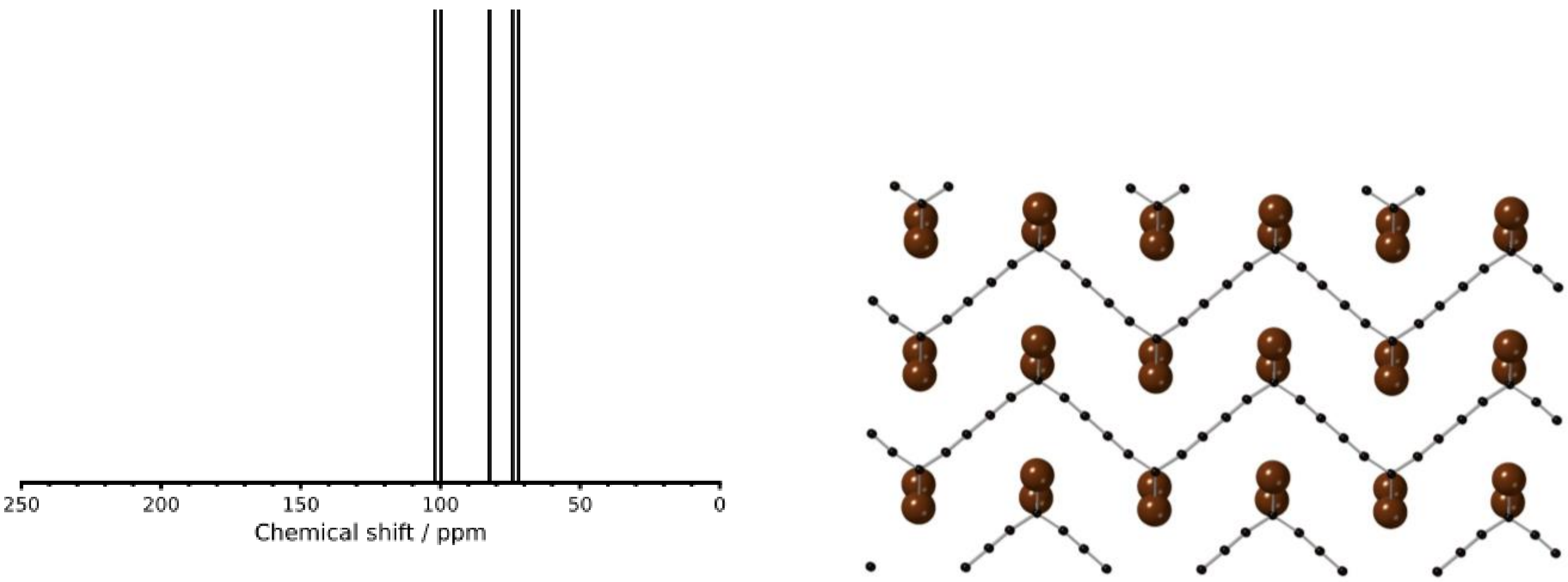
250 200 150 100 50 0
Chemical shift / ppm

BE

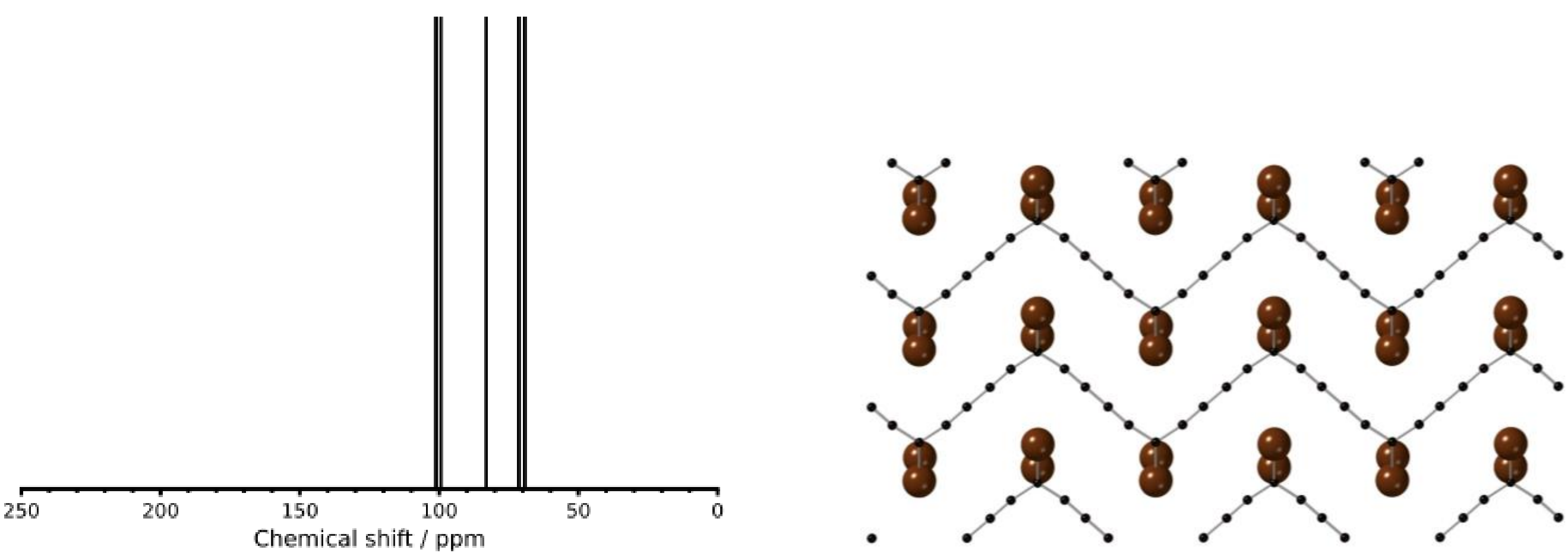
250 200 150 100 50 0
Chemical shift / ppm

BF

OSPC-1 spectra with 1x Br and 1x TMS end groups

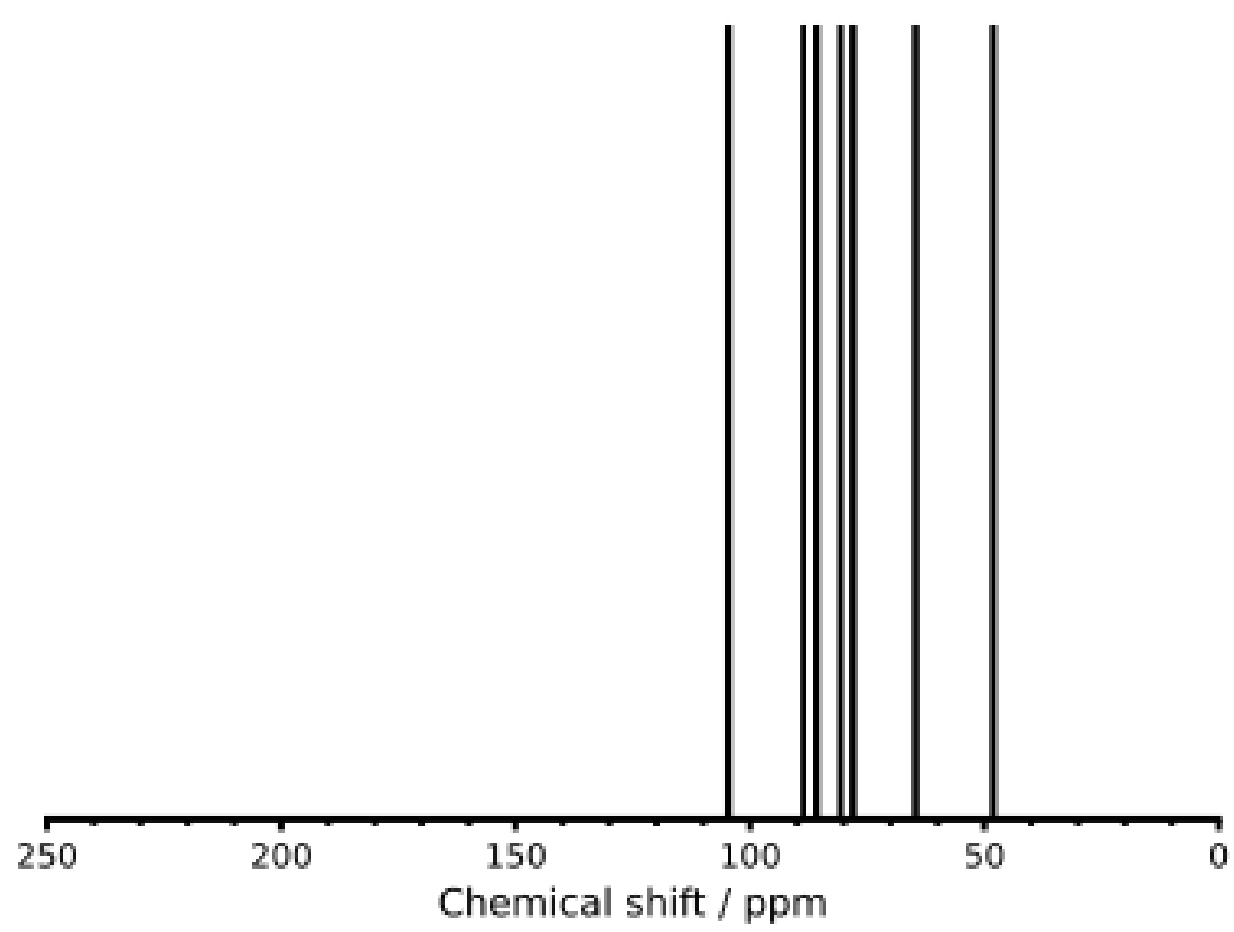

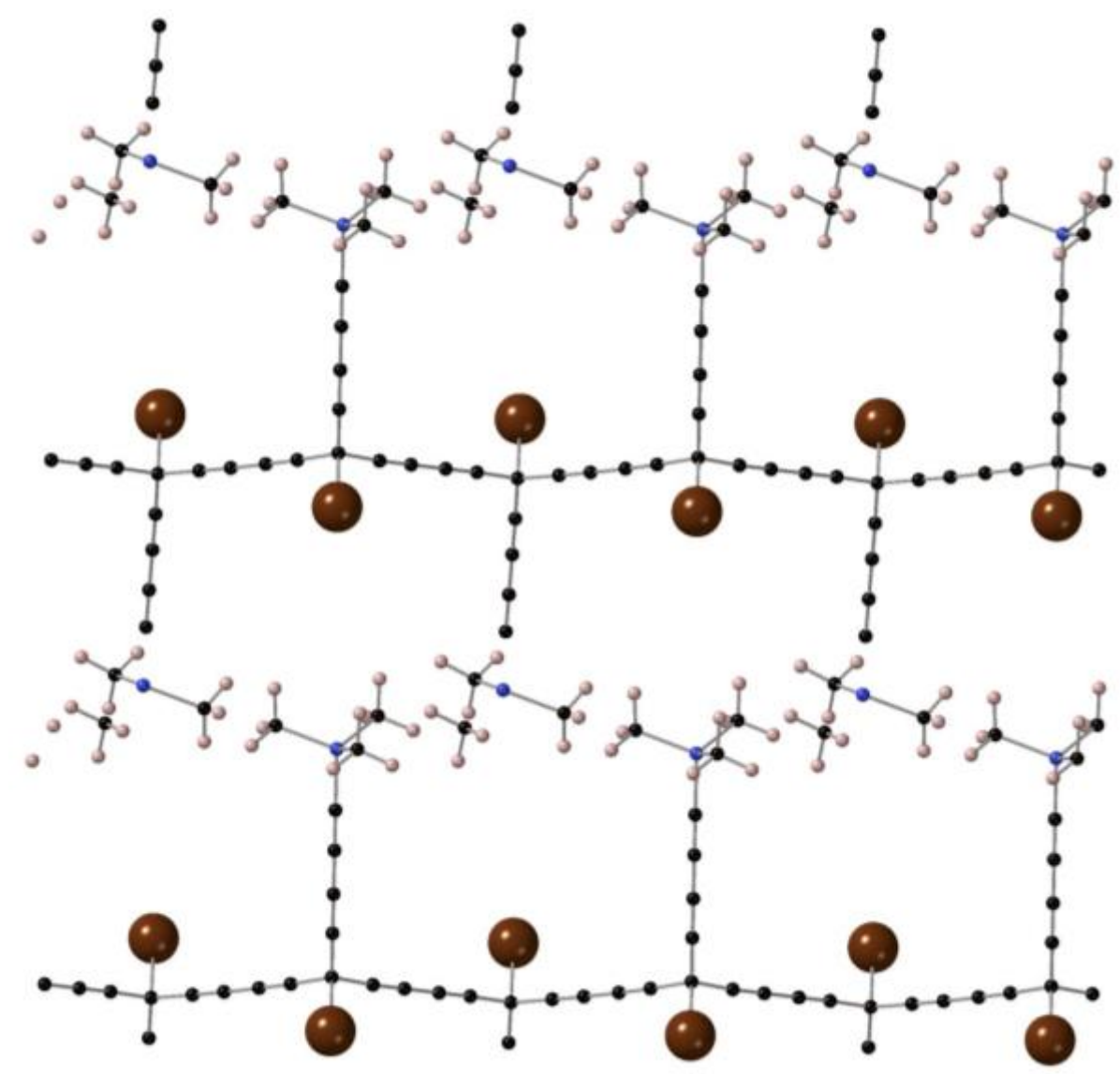

BG

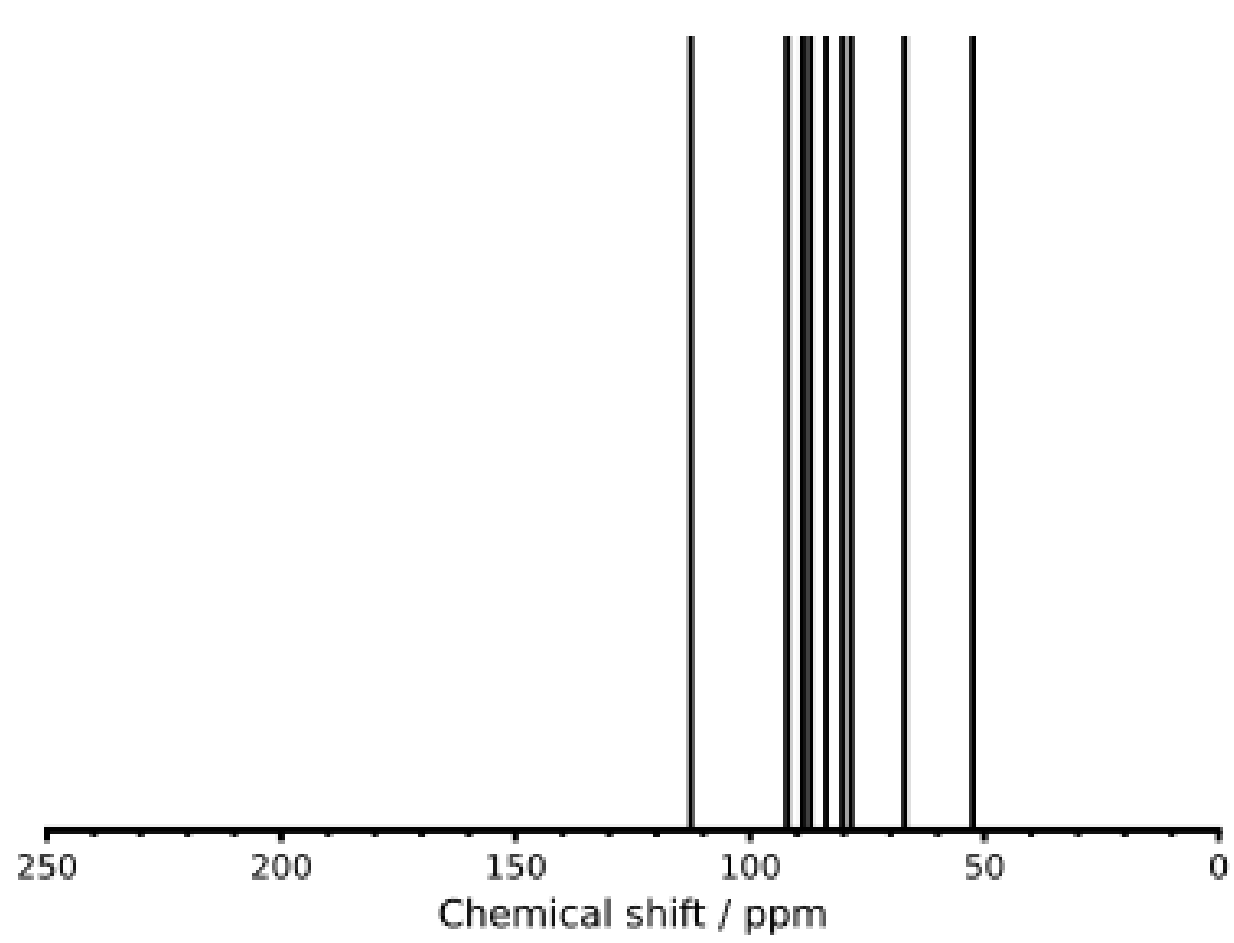

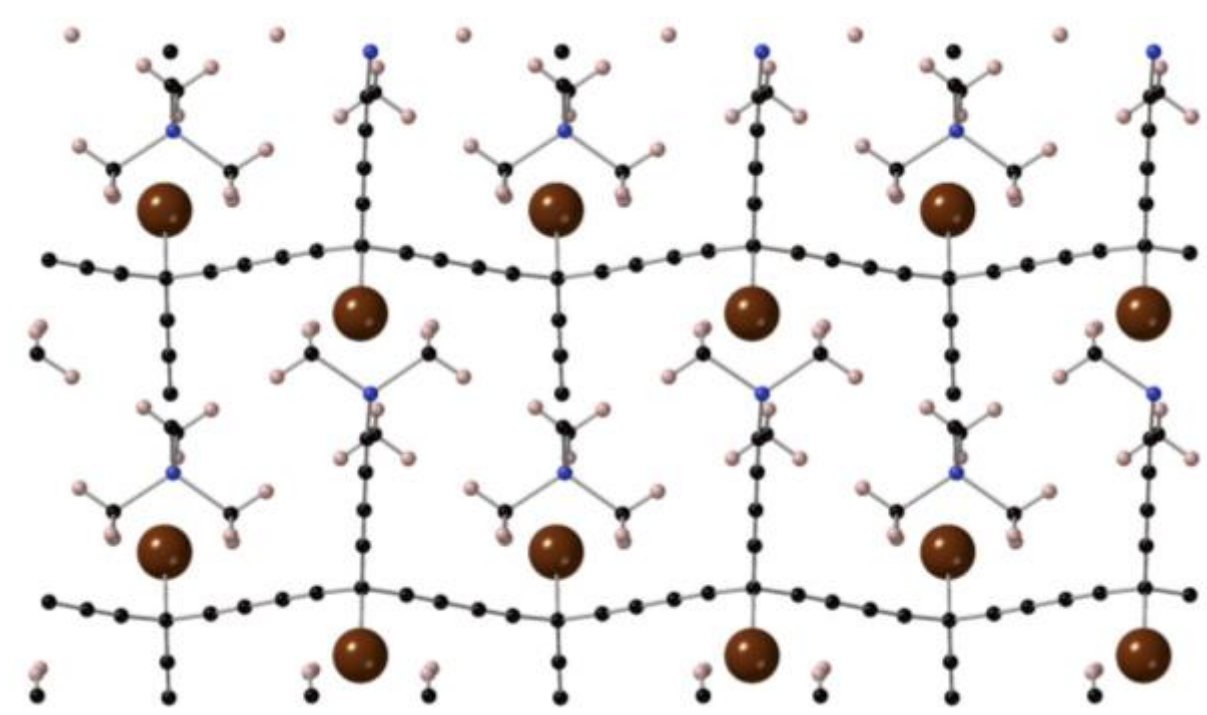

BM

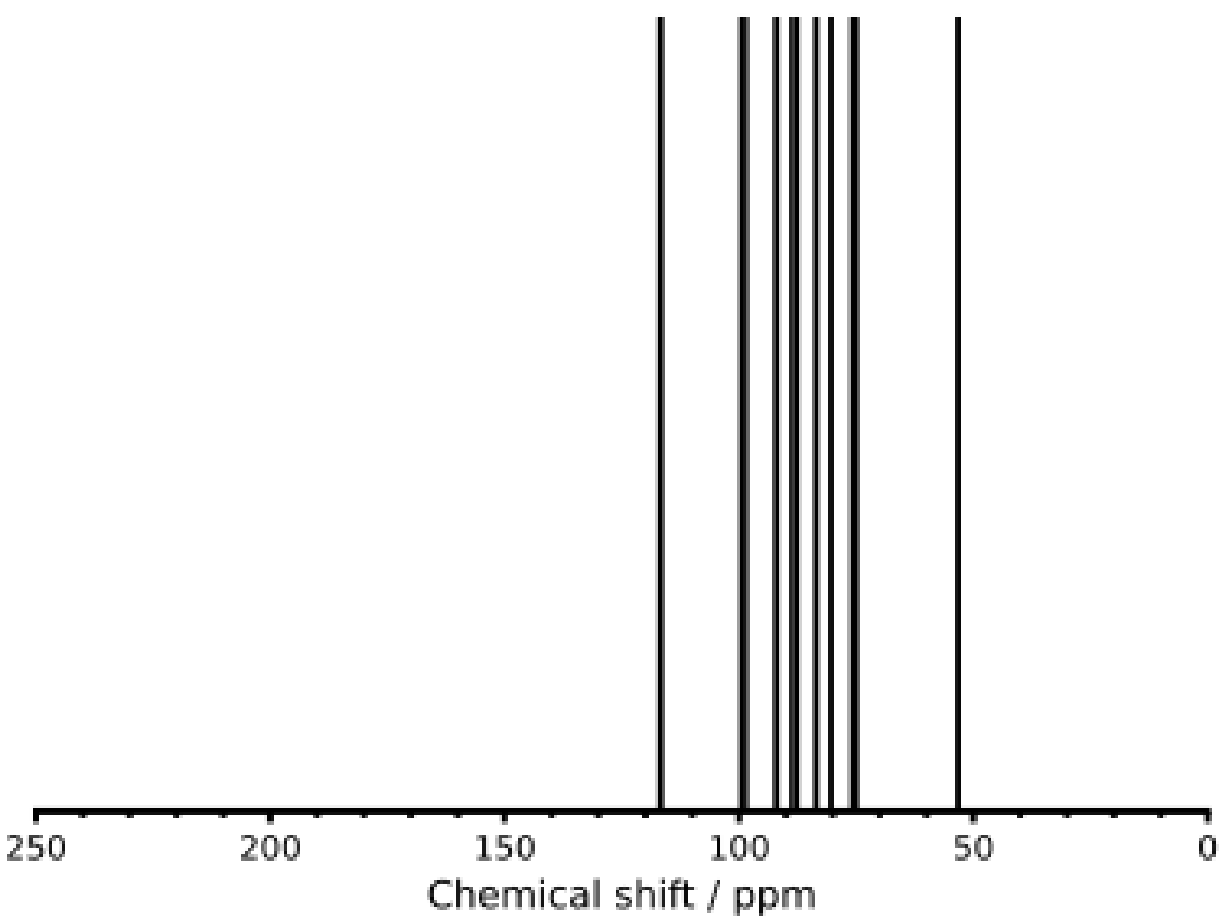
250
200
150
100
50
0
Chemical shift / ppm

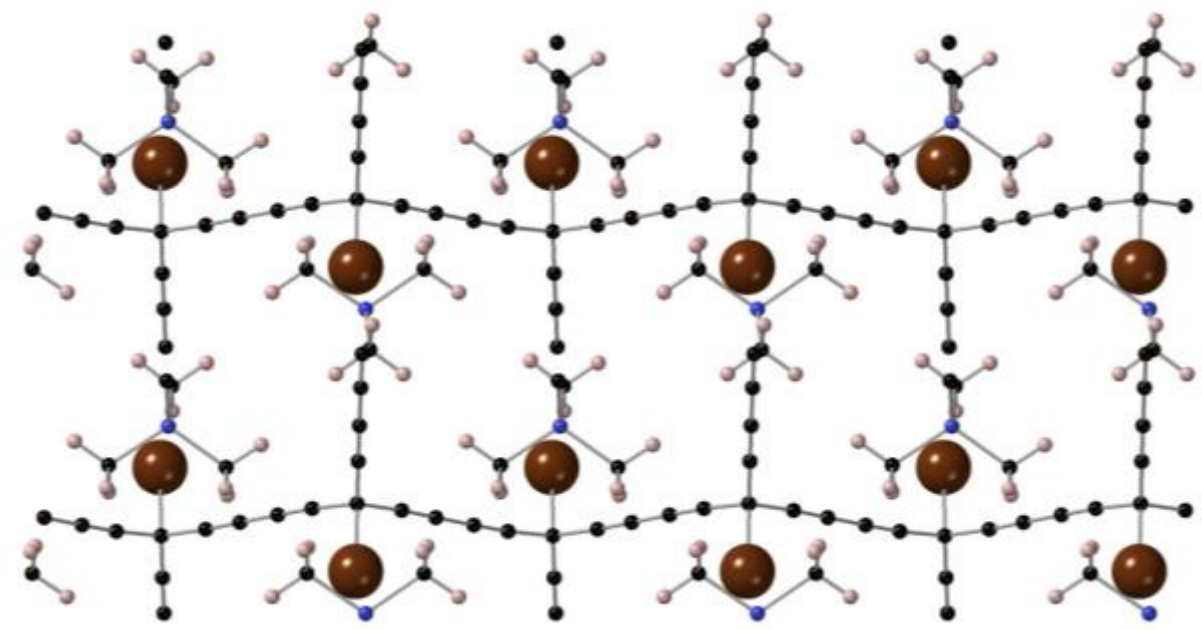

BN

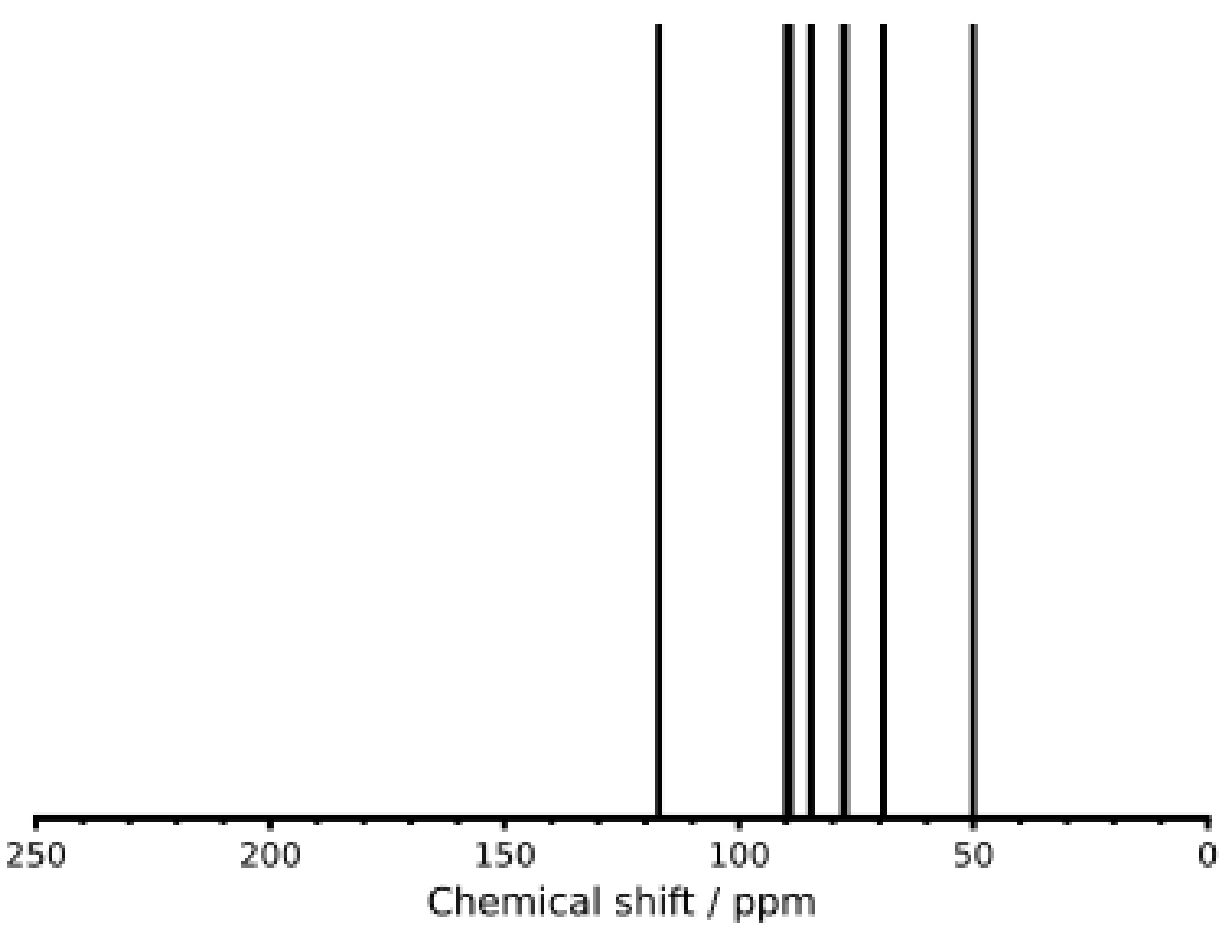
250
200
150
100
50
0
Chemical shift / ppm

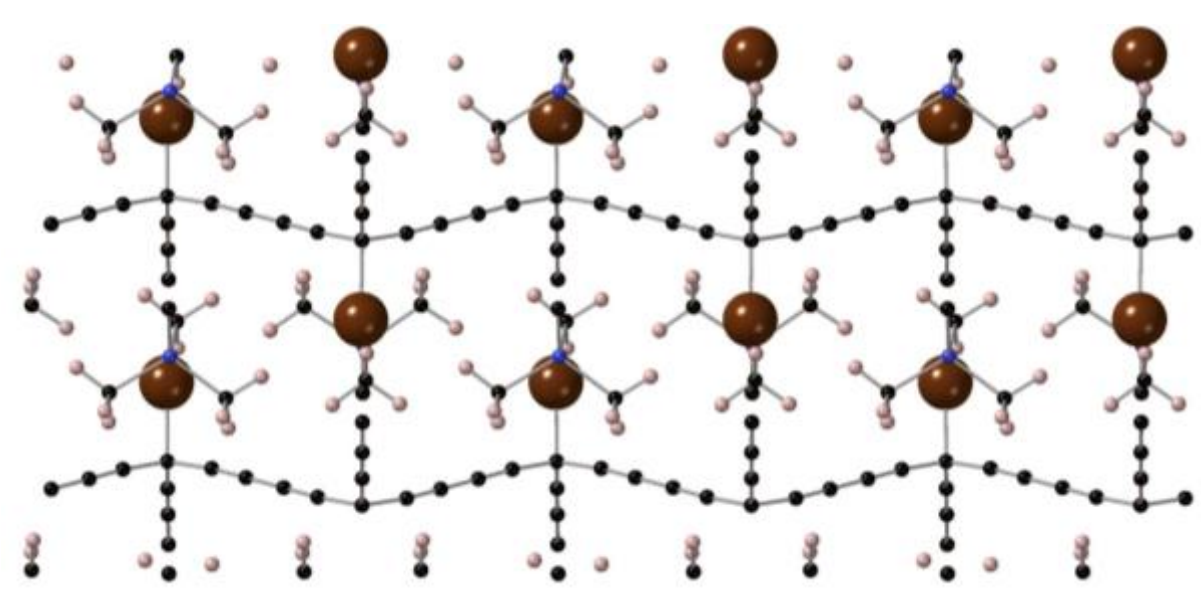

BO

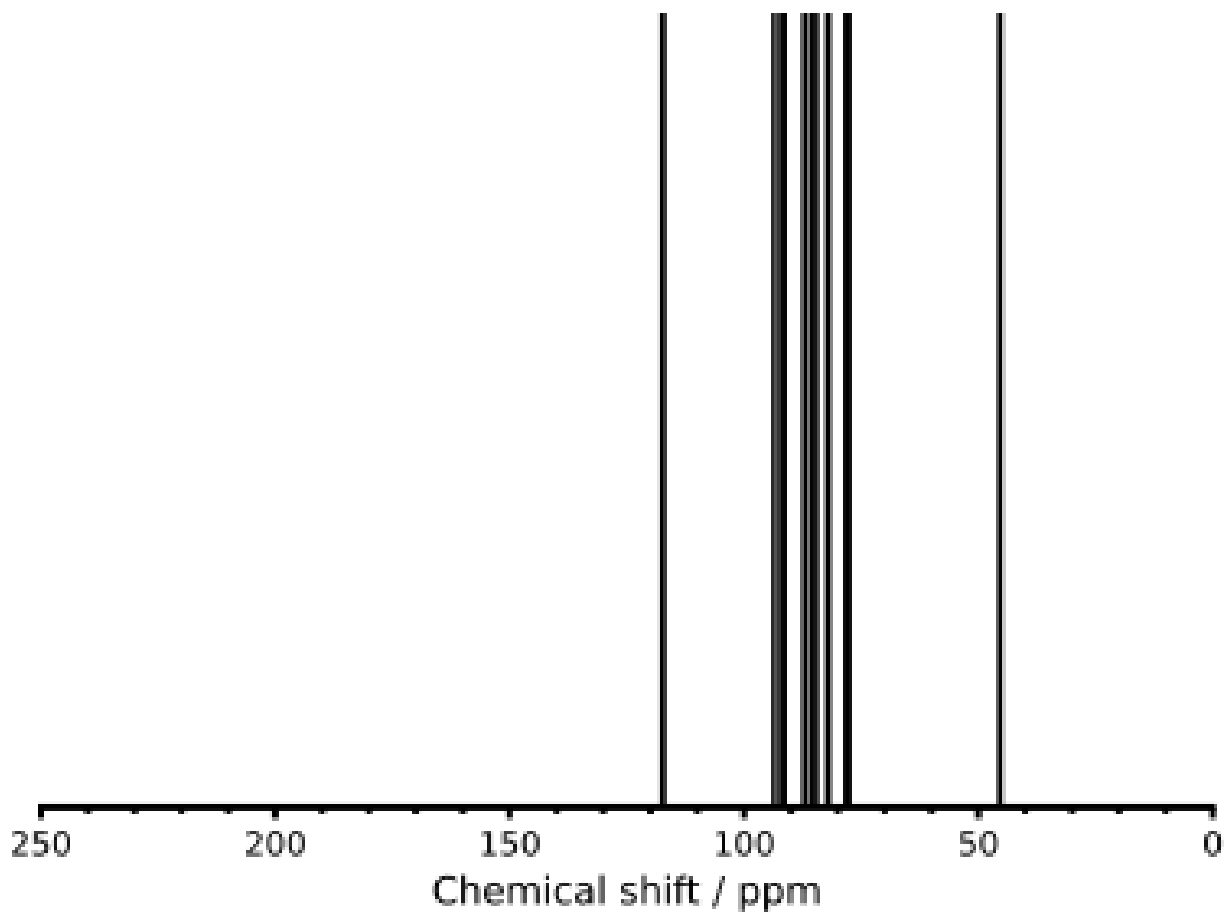
250
200
150
100
50
0
Chemical shift / ppm

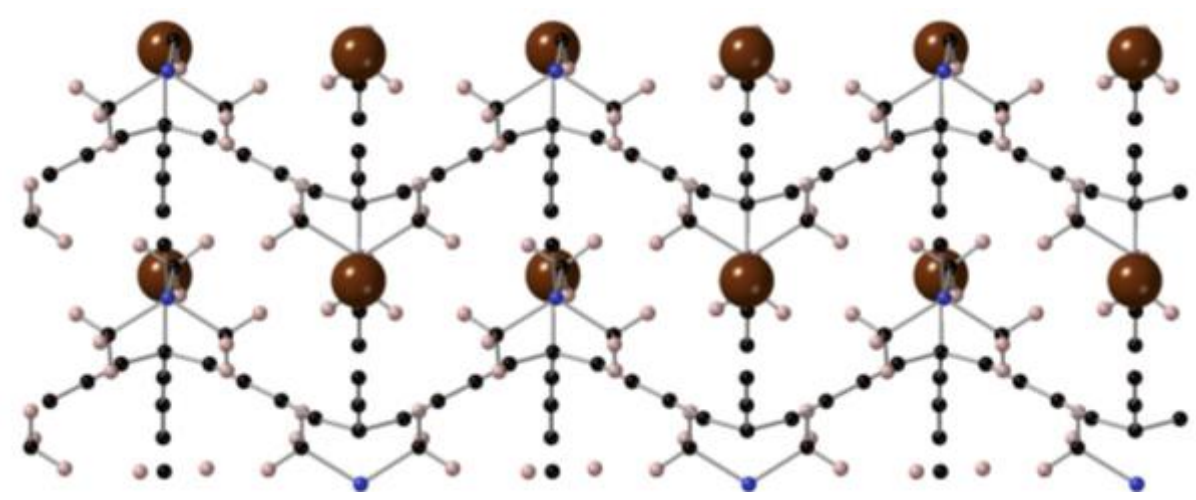

BP

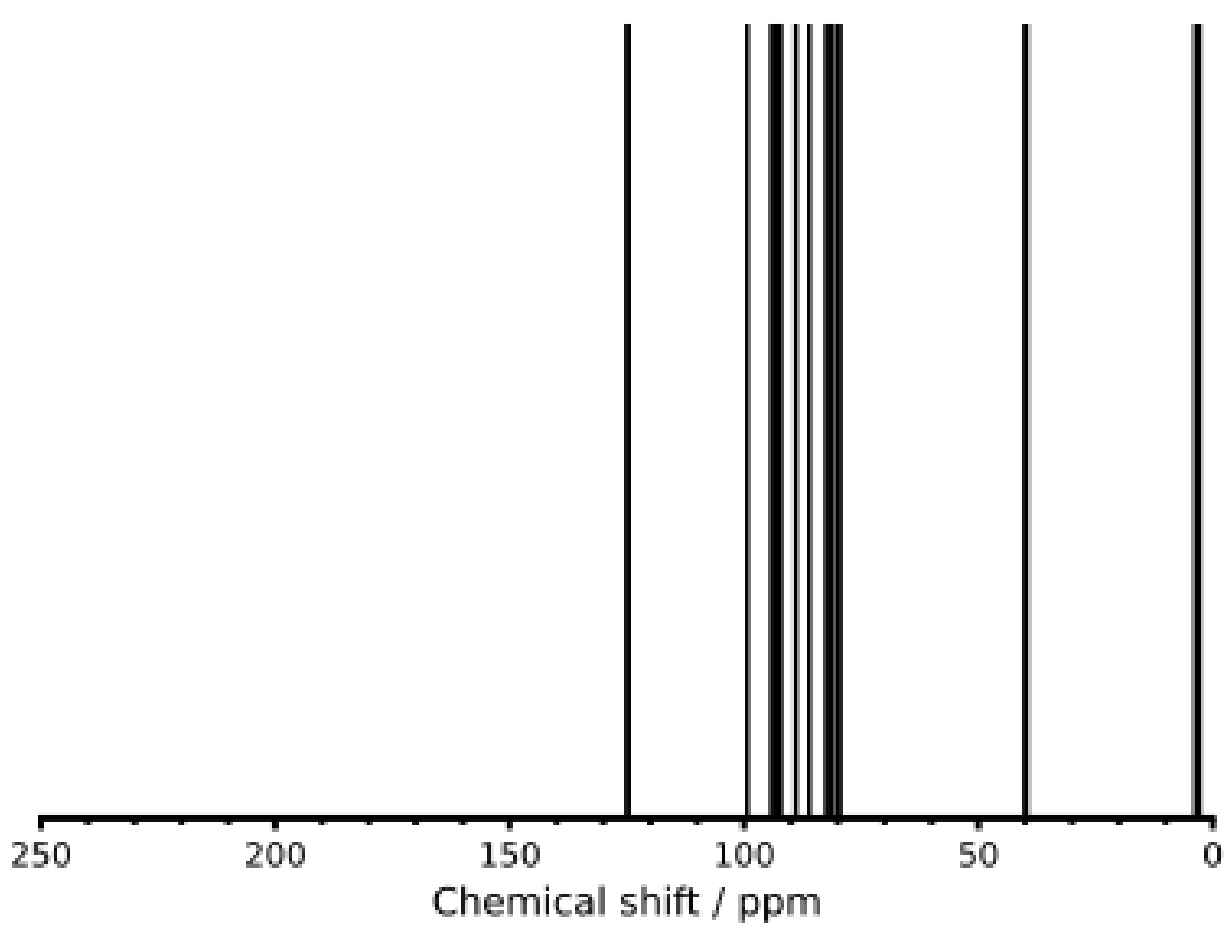
250
200
150
100
50
0
Chemical shift / ppm

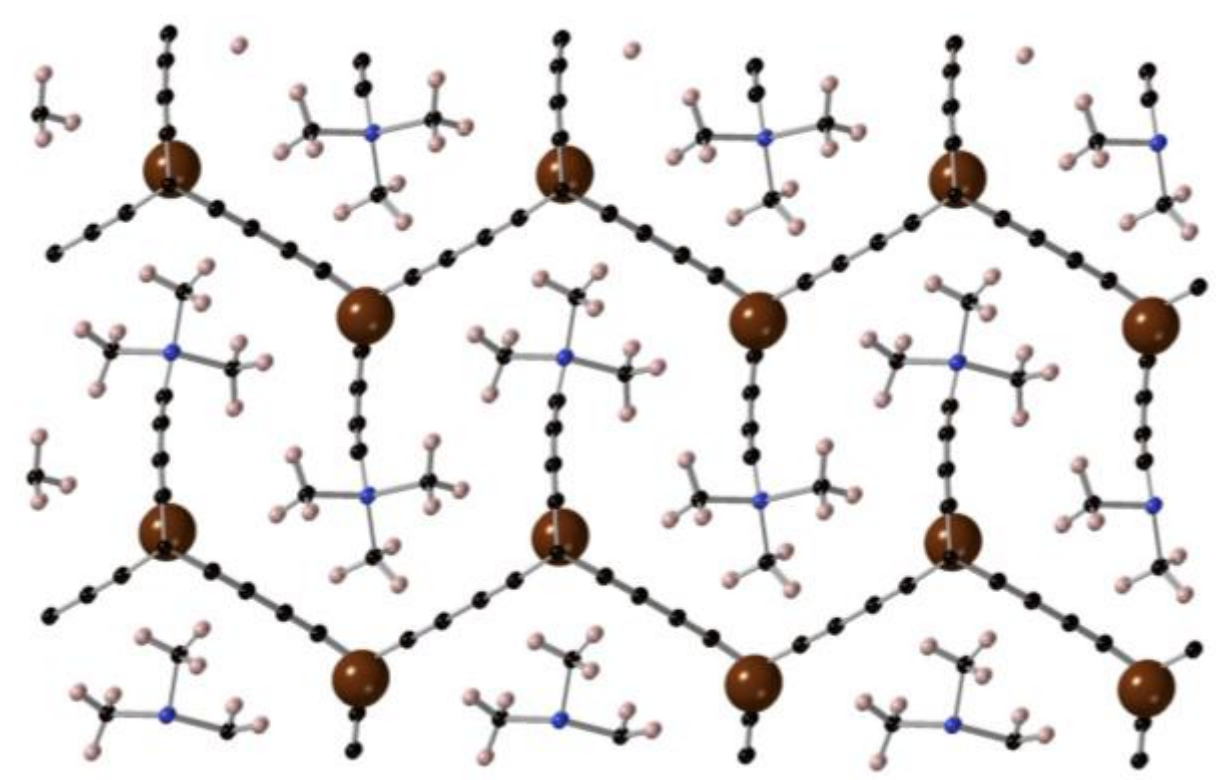

BQ

Section 10. Peak assignments

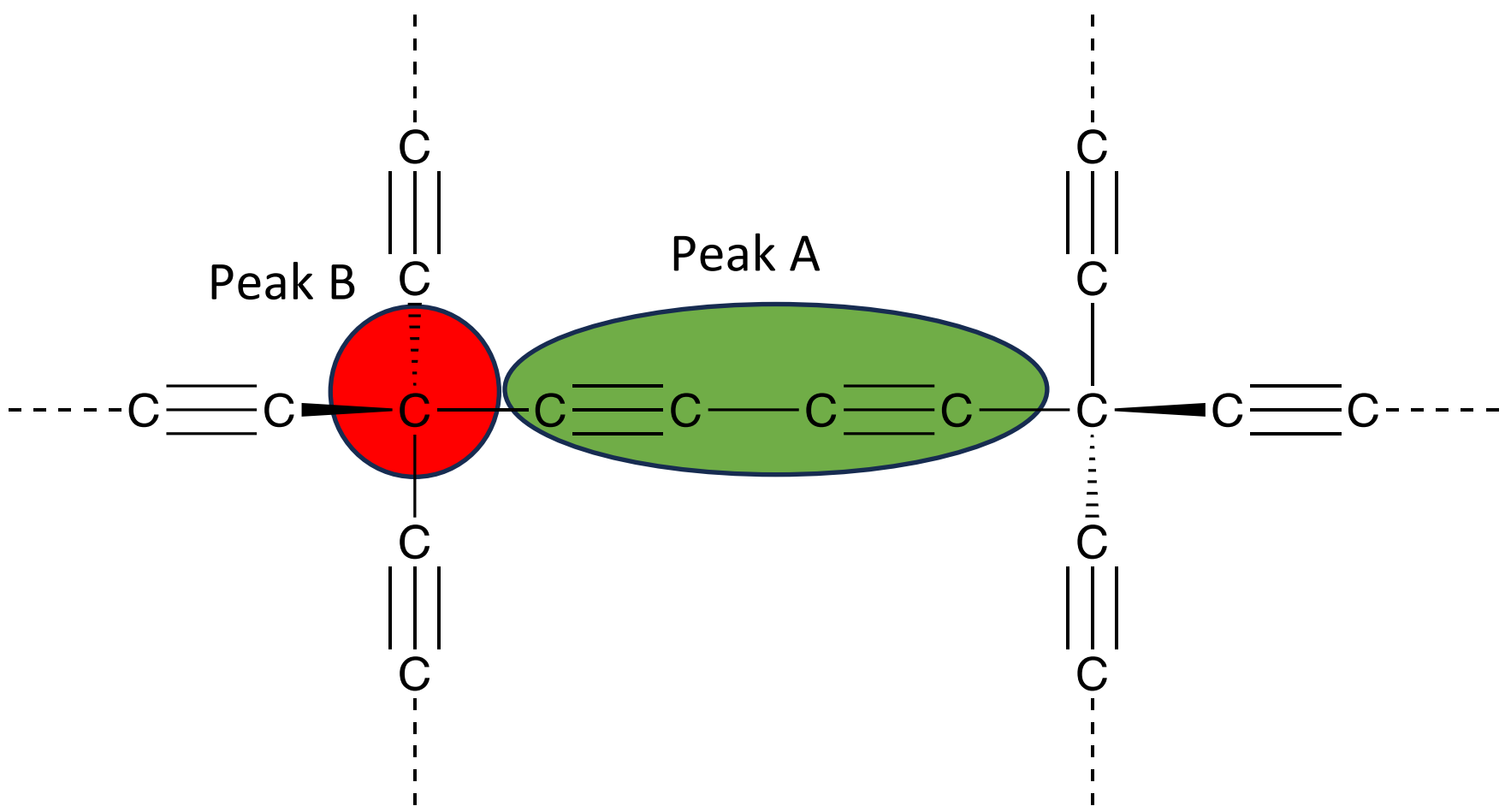

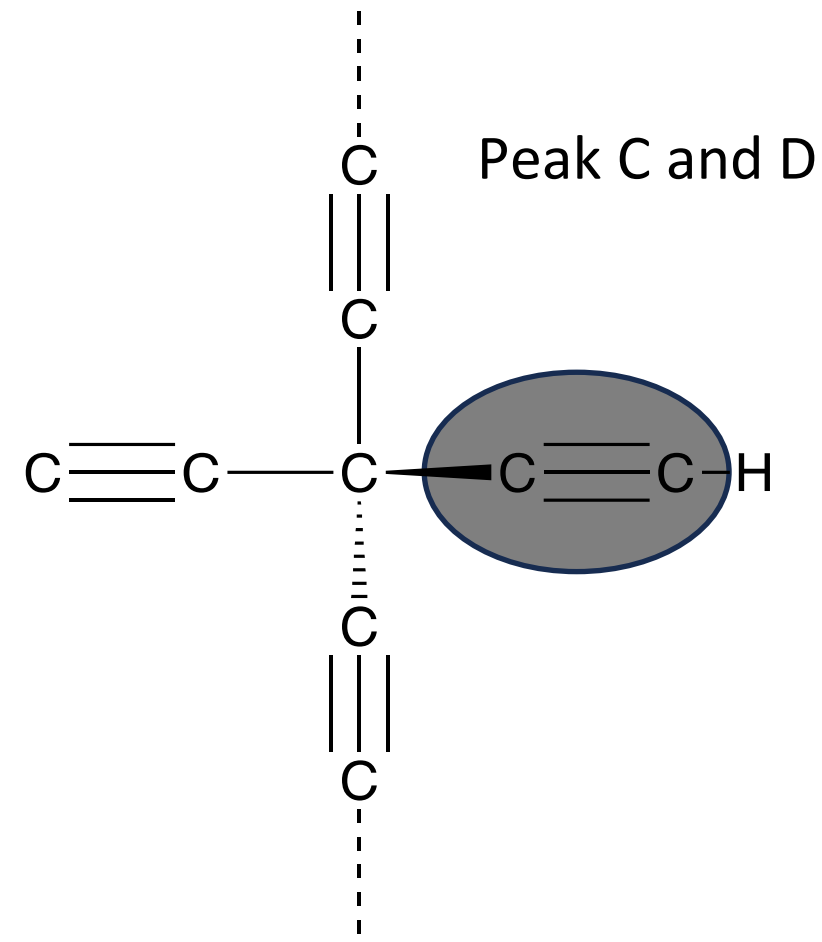

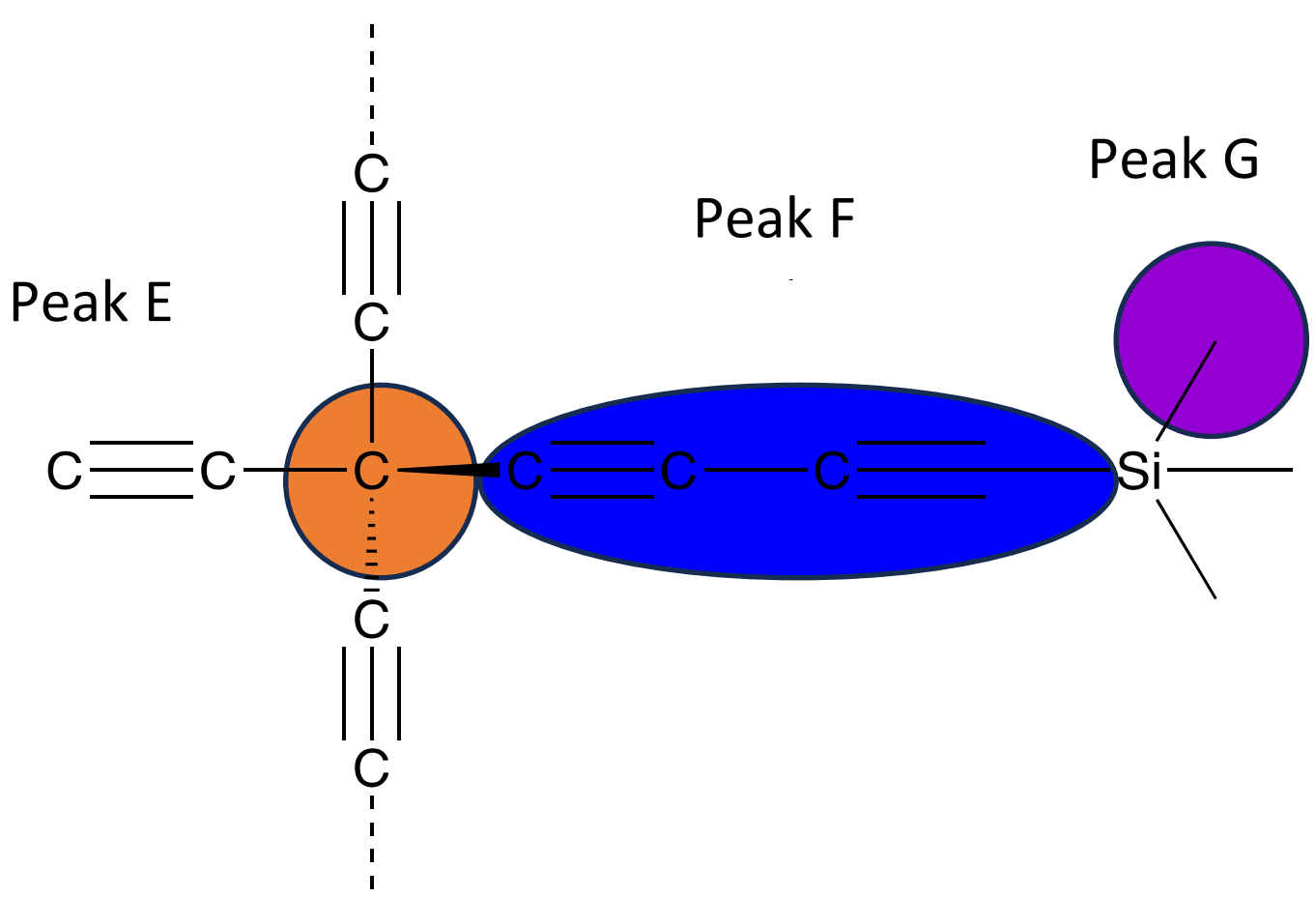

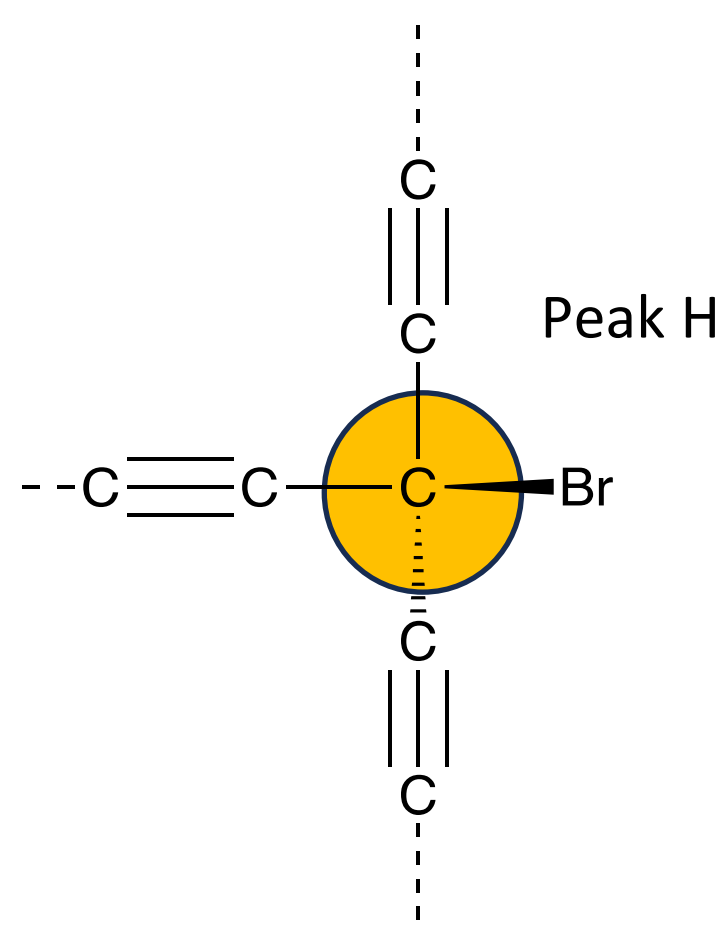

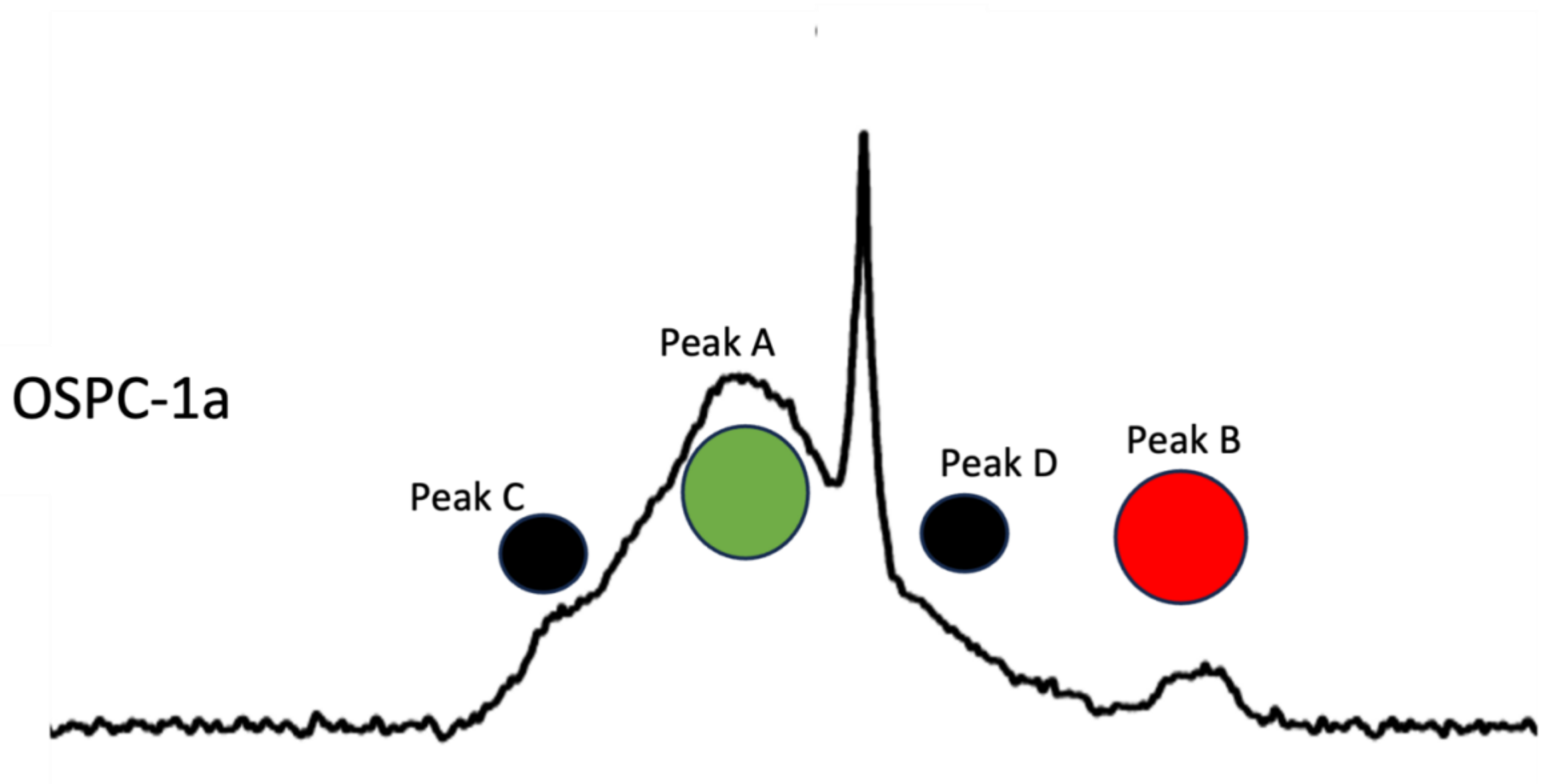

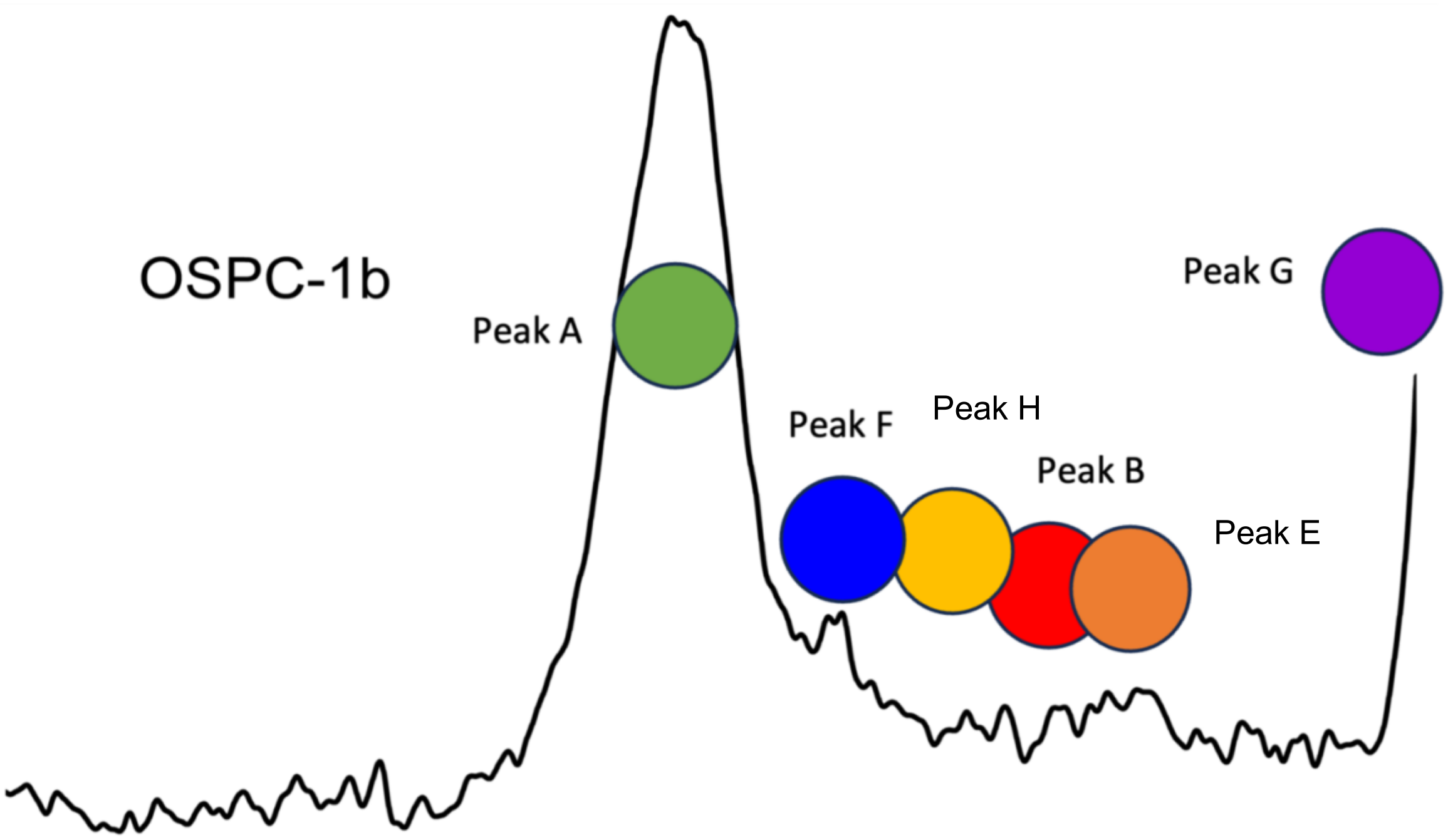

Table SI.20: Ratios of the OSPC-1a peak environments.

Peak	Environment	Area	~Ratio (All)
A	OSPC *sp*	338.4	8.4
B	OSPC sp^3	40.1	1
C	Ethynyl end group	35.1	1
D	Ethynyl end group	35.1	1

Table SI.21: Ratios of the OSPC-1b peak environments.

Peak	Environment	Area	~Ratio (All)	~Ratio (A:B)	~Ratio (B:E:H)
**A**	**OSPC *sp***	**501.3**	**33**	**9.5**	
**B**	**OSPC *sp*^{3}**	**52.6**	**3.5**	**1**	**3.5**
**E**	**C–Br**	**25.1**	**2**		**2**
**F**	**Ethynyl-TMS end group**	**100.3**	**7**		
**G**	**TMS**	**97.5**	**6.5**		
**H**	**C-ethynyl-TMS *sp*^{3}**	**15.05**	**1**		**1**

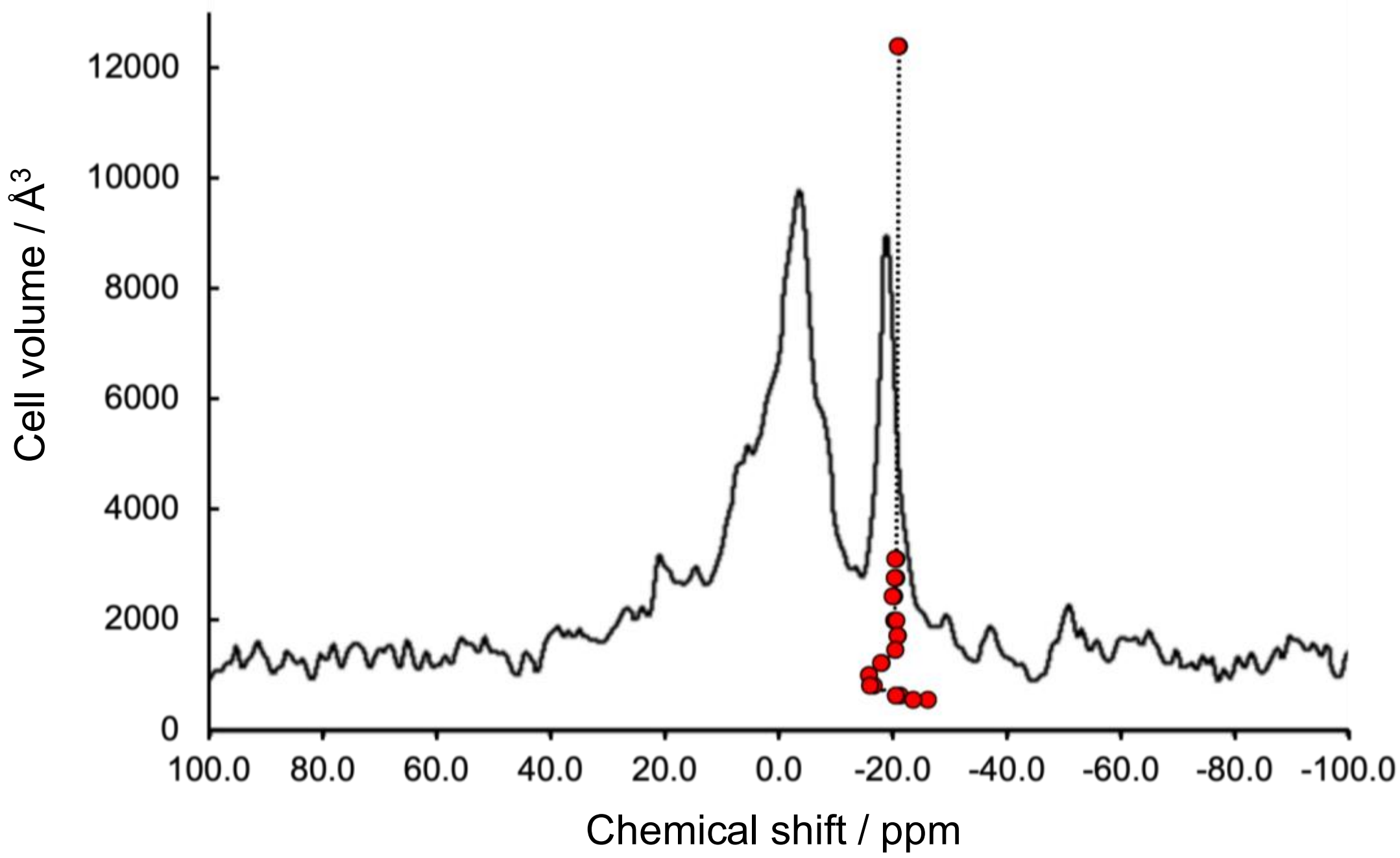

Figure SI.49: Experimental ^{29}Si NMR spectrum (black) compared to the calculated ^{29}Si NMR spectrum (CASTEP) for the two Si atoms in the model (red) as a function of cell volume for models BG-BQ and an additional high-volume model. Calculated NMR referenced to the calculated value of a TMS molecule.

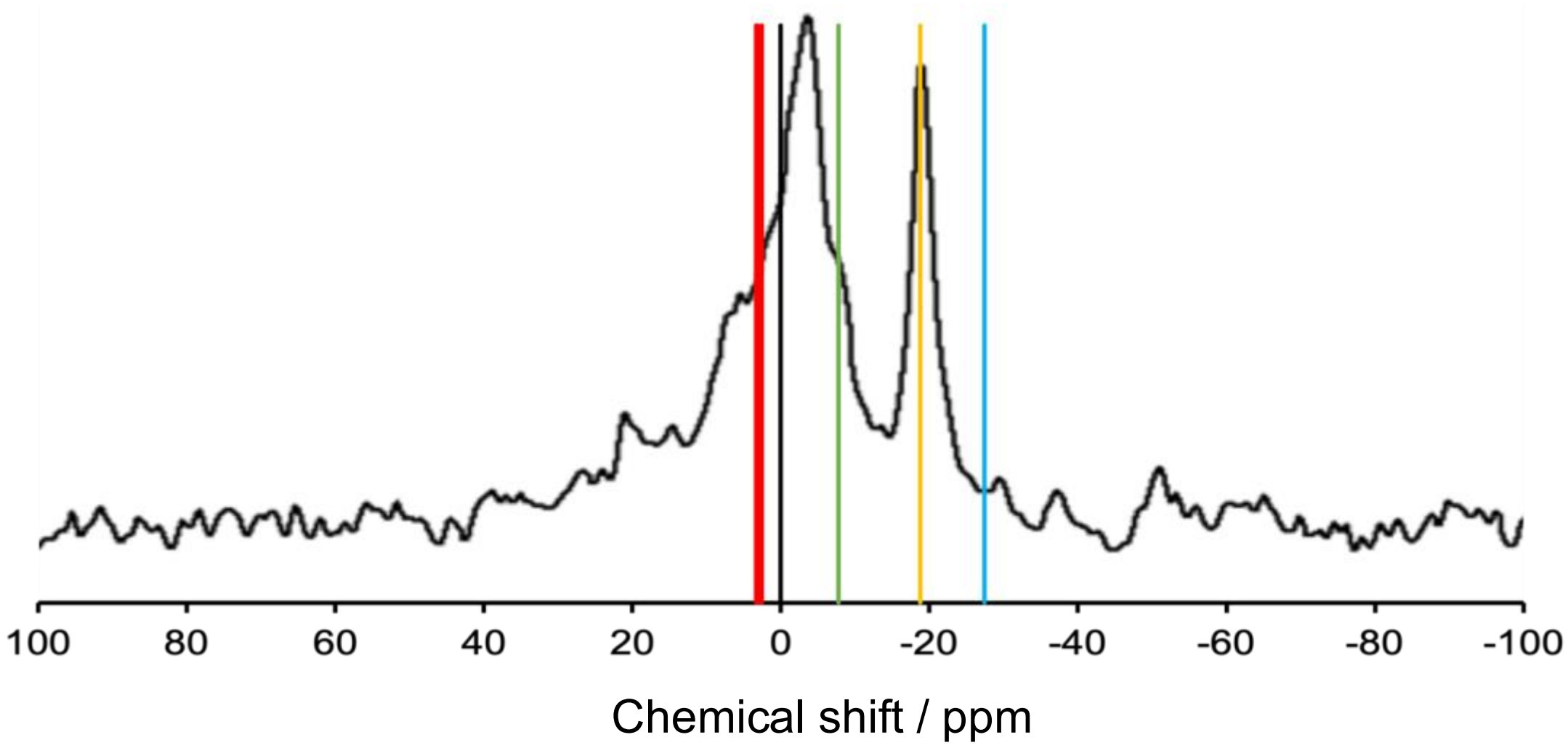

Figure SI.50: Experimental ^{29}Si NMR spectrum (black) compared to the calculated ^{29}Si NMR spectrum (Gaussian) for models of systems containing Si atoms. Calculated NMR referenced to the calculated value of a TMS molecule. Red lines are a TMS molecule in close proximity to an OSPC fragment at distances between ~14 and 8 Å to the central OSPC node or Br terminated OSPC end group (they overlap). The black line is a TMS molecule. The green line is the Si atom of a TMS molecule in close proximity to a TMS terminated OSPC fragment. The yellow line is a TMS terminated OSPC fragment. The blue line is the Si in a TMS terminated OSPC fragment in close proximity to a TMS molecule.

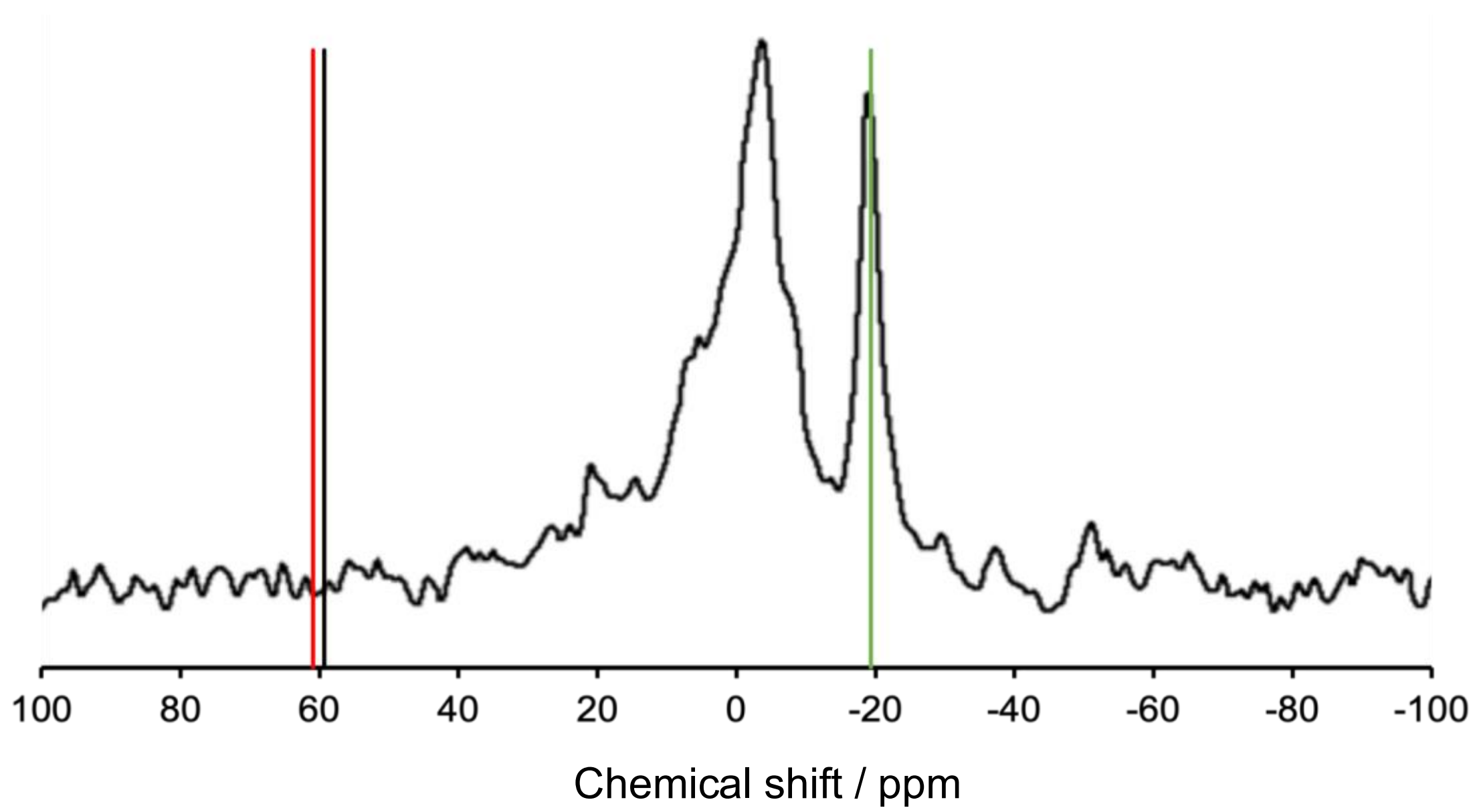

Figure SI.51: Experimental ^{29}Si NMR spectrum (black) compared to the calculated ^{29}Si NMR spectra (Gaussian) for models of systems containing Si atoms. Calculated NMR referenced to the calculated value of a TMS molecule. Red lines are $(Me)_2SiF_2$. The black line is a $(Me)_3SiF$ and $(Me)SiF_3$ (they overlap). The green line is the SiF_4.

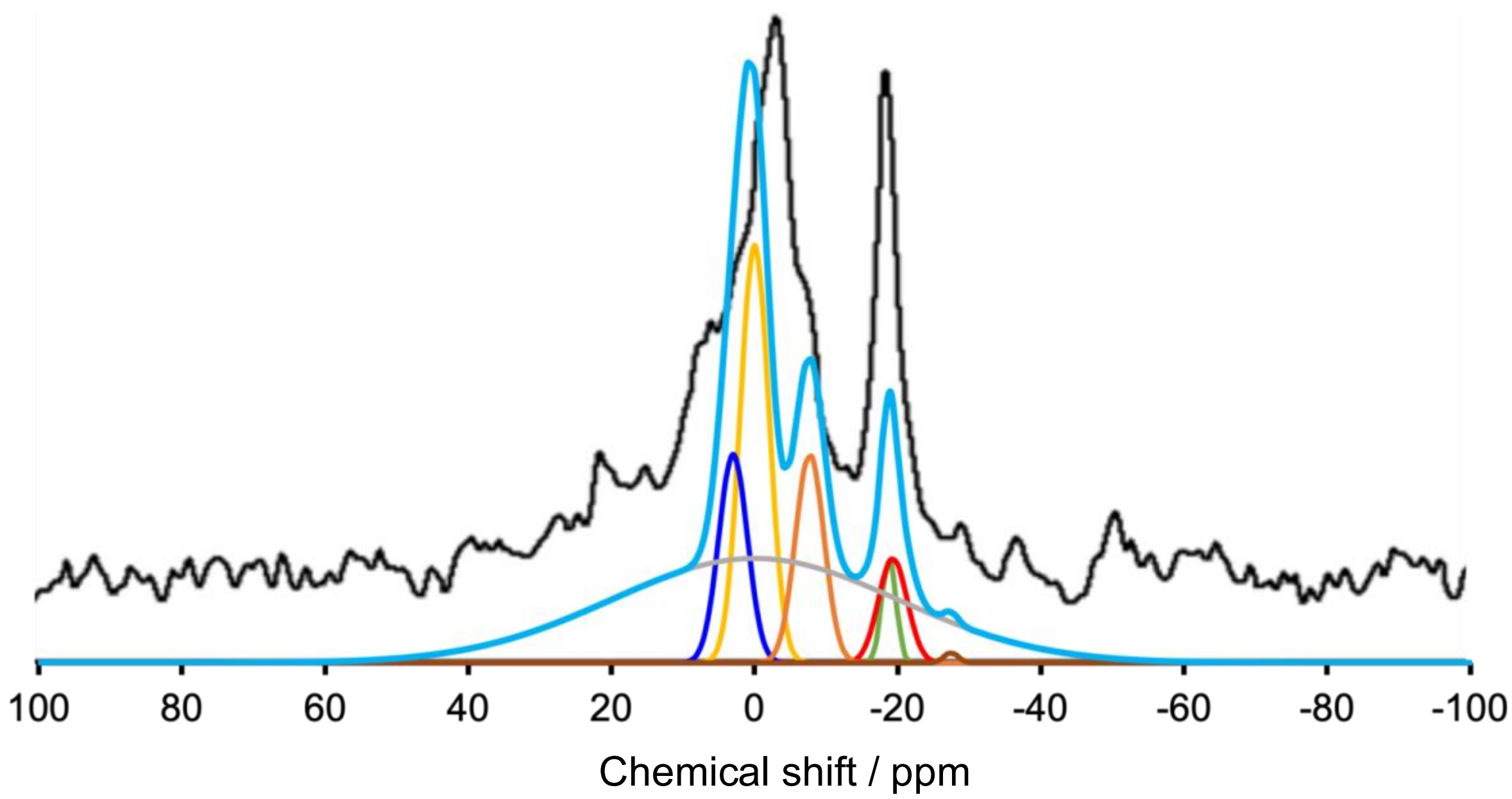

Figure SI.52: Experimental ^{29}Si NMR spectrum (black) compared to a modelled spectrum derived from the computationally determined ^{29}Si NMR peak positions shown in Figures SI.50 –SI.51 with a Gaussian function applied. The light blue line is the total modelled spectrum. The individual Gaussian function assignments are where green is attributed to a Si atom in a TMS end group, red is attributed to the Si atom of an adsorbed SiF_4, brown is attributed to the Si atom of a TMS end group interacting with molecular TMS, orange is attributed to the Si atom of a molecular TMS interacting with a TMS end group, yellow is attributed to the Si atom of molecular TMS, dark blue is attributed to molecular TMS interacting with OSPC-1 nodal centres, grey is attributed to molecular TMS interacting with OSPC-1 in a broad range of polymeric sites. Full assignments shown below.

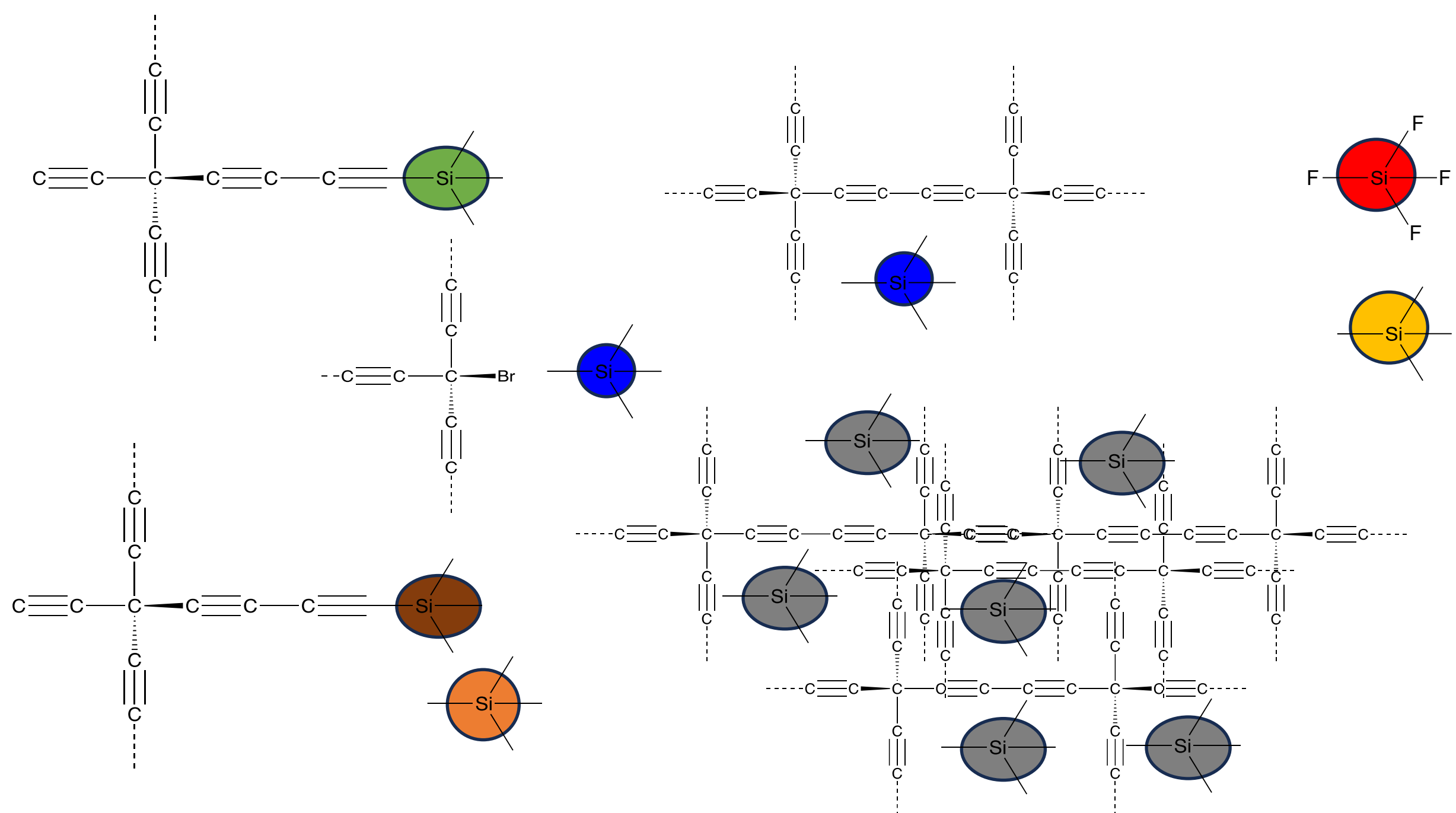

Section 12. Pore size distribution and TEM

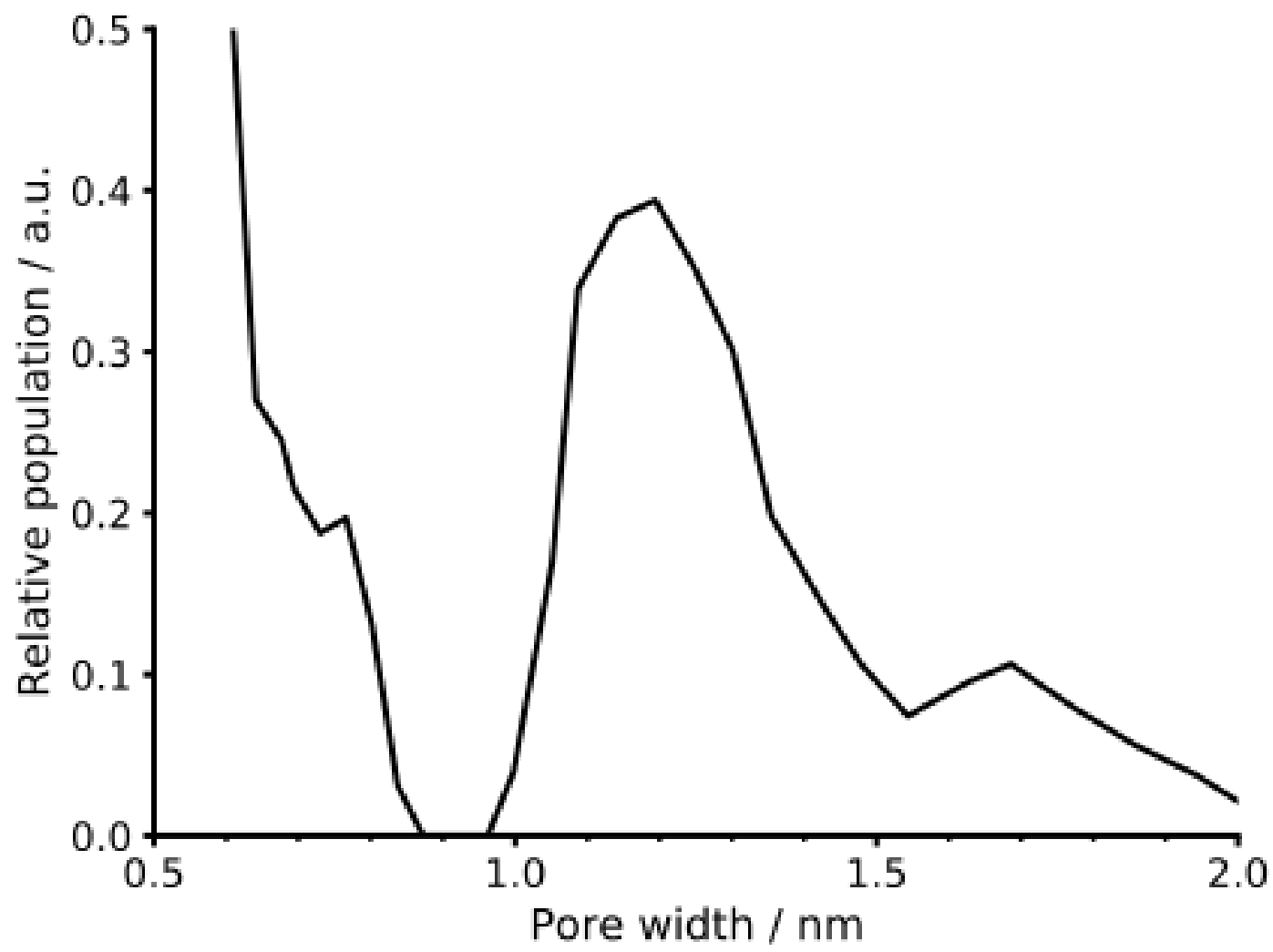

Figure SI.53: PSD of OSPC-1b.[2]

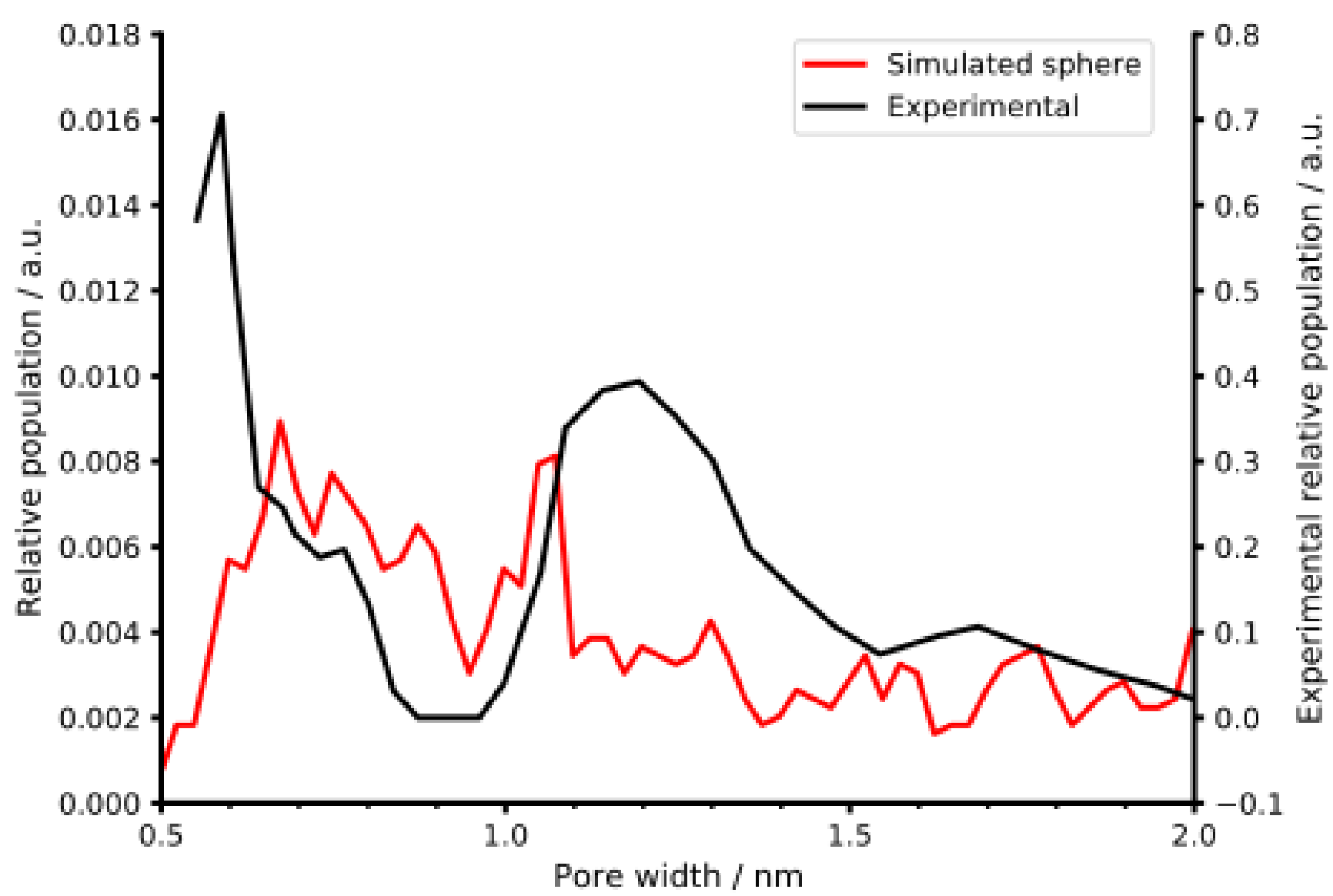

Figure SI.54: PSD of the spherical model. Key: experimental PSD – black,[2] PSD of the simulated sphere (calculated using Poreblazer 4.0[24]) – red.

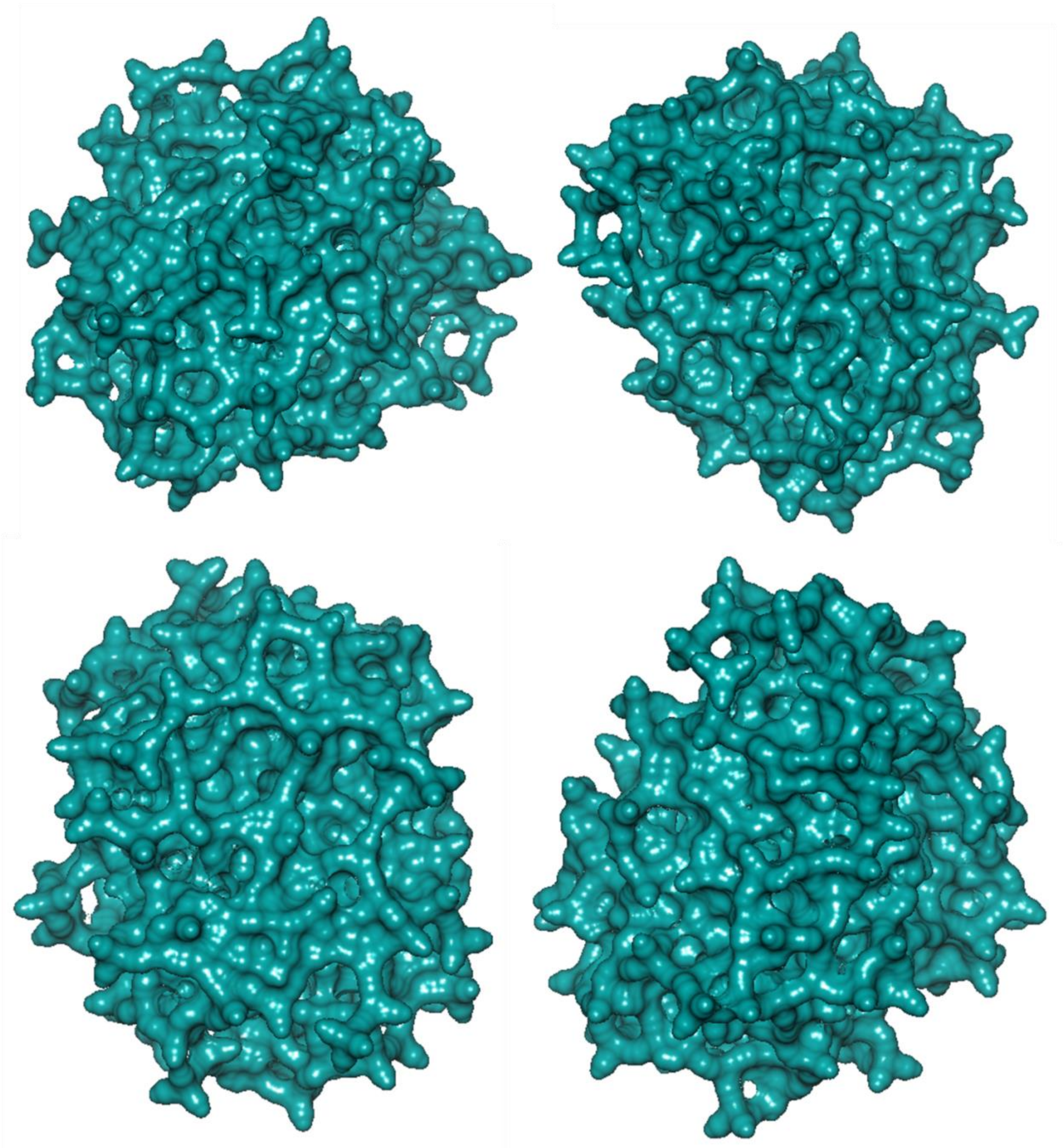

Figure SI.55: OSPC-1 sphere showing a van der Waals surface.

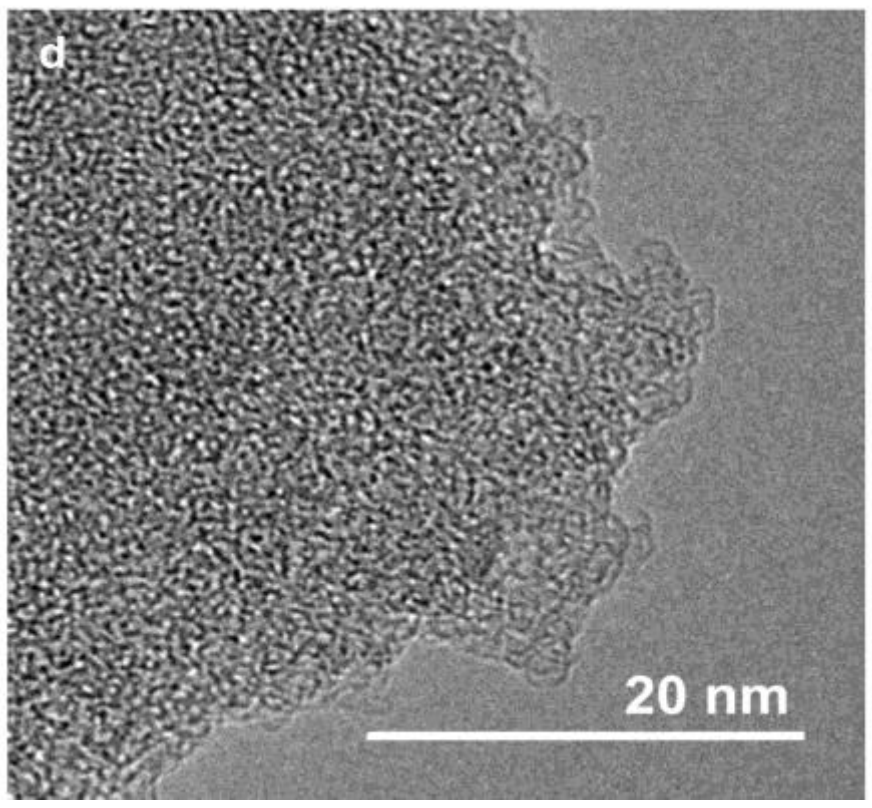

(a)

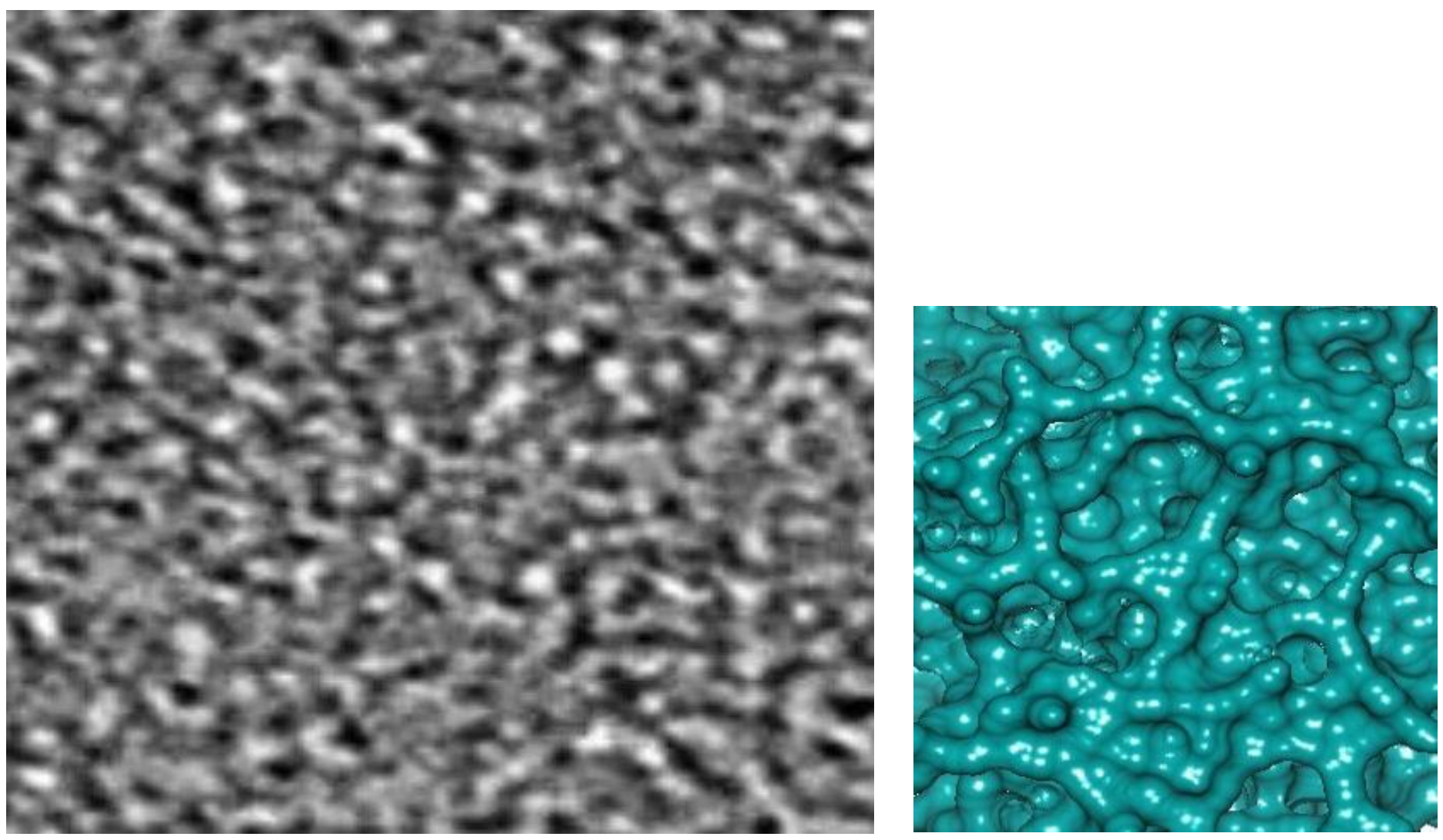

(b)

Figure SI.56: (a) High resolution TEM of OSPC-1a[1]. **(b)** A zoomed in section (left) in comparison to a similarly scaled section of the spherical model of OSPC-1 (right).

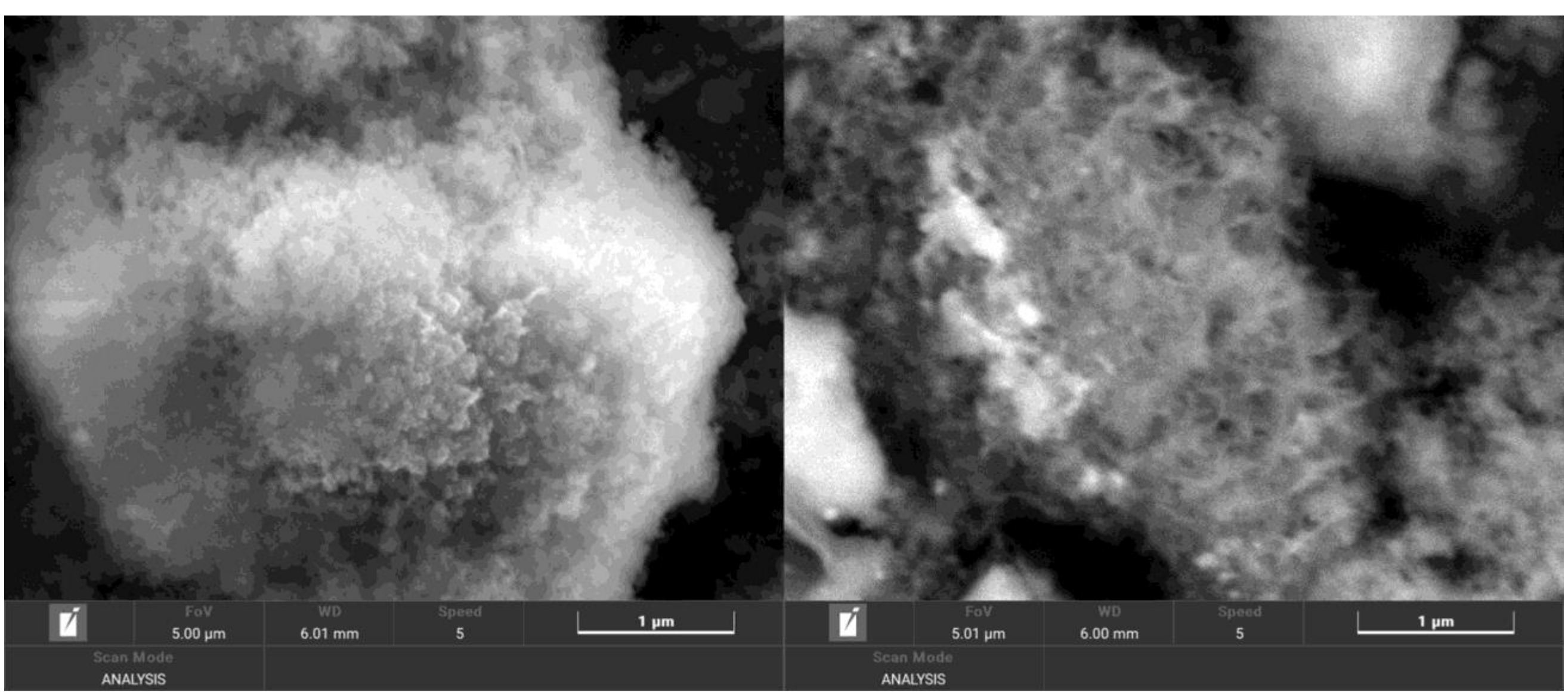

Figure SI.57: High magnification images of OSPC-1b shell regions, showing porosity.

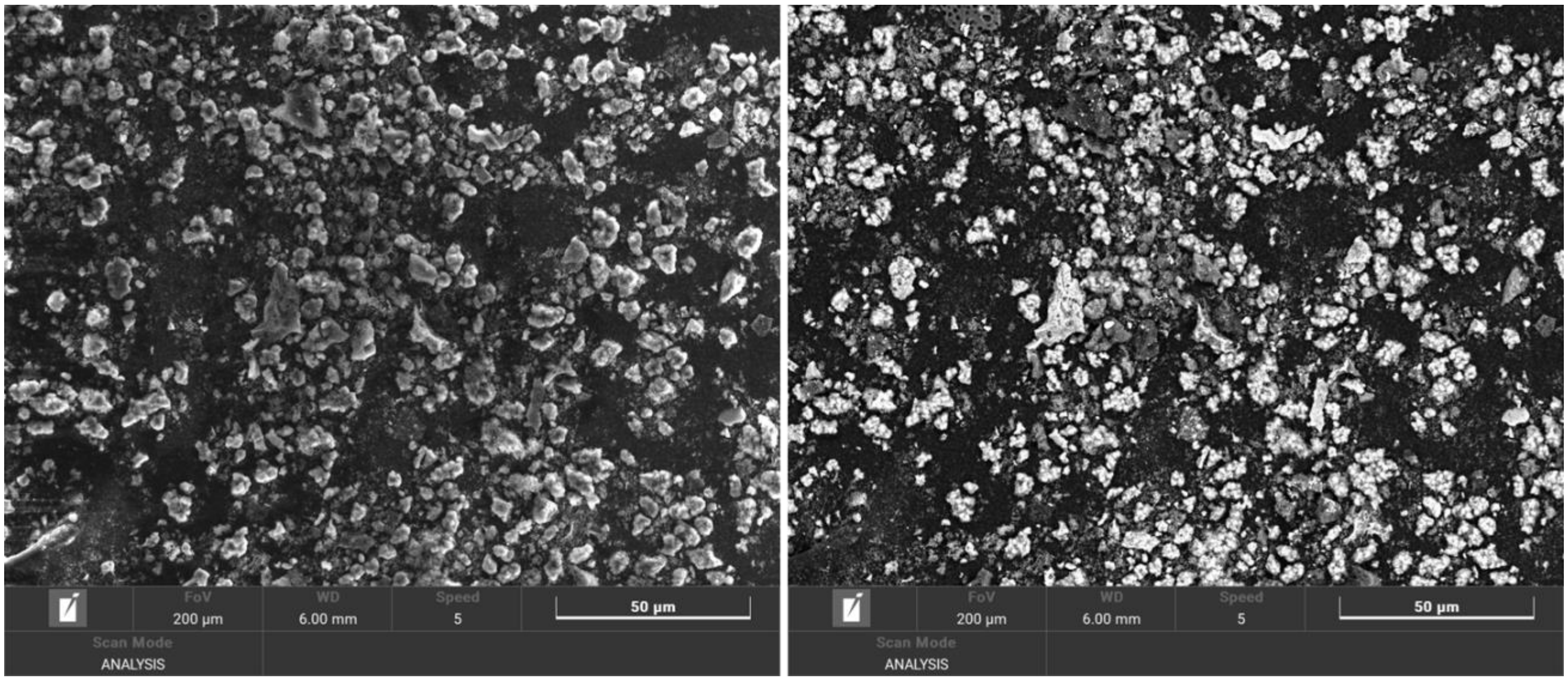

Figure SI.58: Lower magnification image (left: without backscatter, right: with) showing presence of shell regions across all particles.

Surface Area approximation

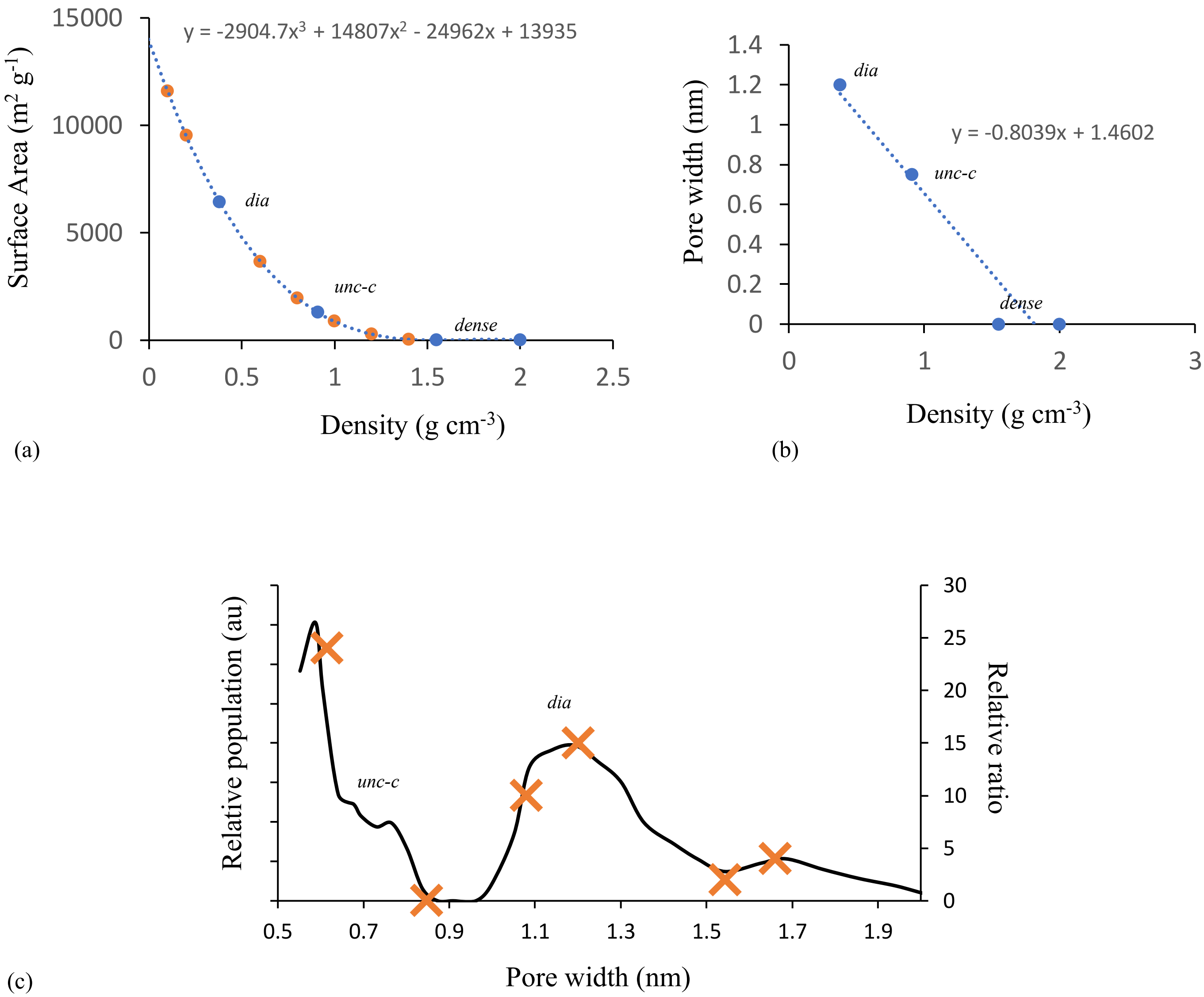

Figure SI.59: (a) Plot of surface area versus density. The *dia*, *unc-c*, and dense model densities and modelled surface area were used to generate the trend line and the associated equation from which the density and associated surface areas were calculated shown in orange. (b) Plot of pore width versus density. The *dia*, *unc-c*, and dense model densities and modelled pore widths were used to generate the trend line and the associated equation from which the pore widths for the associated densities were calculated. (c) Plot of pore width versus relative population (black line – experimental pore size distribution) and the relative ratio for each pore width used to determine the surface area (orange). The pore widths of *dia* and *unc-c* models are labelled.

Protocol:

1. Density to surface area relationship was determined using plot SI.59(a)
2. Density to Pore width relationship was determined using plot SI.59(b)
3. Density used to determine the surface area contribution for each associated % volume.

4. The ratio of densities relative to each other used for the microporous region is determined from the ratios fitted to the pore widths shown in plot SI.59(c).
5. The relative ratios of microporous region are kept the same as the 'fuzzy' phase but the total fuzzy to dense phase ratio is differed to match the modelled fuzzy to dense ratios.

Note, that this protocol assumes that the surface area in the micropore region arises solely from the OSPC structure. In practice, pore volume can arise from a range of physical sources including inter-particulate voids which typically produce apparentin pore widths in the 1.5-2 nm region. This approach therefore provides only a rough estimate, suitable as a sanity check, and the resulting surface areas should not be over interpreted.

70:30 dense to fuzzy ratio:

	volume ratio	% volume	pore width (nm)	density (g/cm3)	mass (g) if have 1 cm3	Surface Area. (m2 g-1)	surface area contribution (m2 g-1)
dense	70.04	70.04	0.21	1.55	1.09	0.00	0.00
fuzzy	29.96	0.00	0.15	1.40	0.00	39.42	0.00
		0.00	0.38	1.20	0.00	283.36	0.00
		13.07	0.61	1.00	0.13	875.30	114.42
		0.00	0.85	0.80	0.00	1954.67	0.00
		5.45	1.08	0.60	0.03	3660.90	119.64
		8.17	1.20	0.38	0.03	6428.00	199.14
		1.09	1.54	0.20	0.00	9511.64	20.72
		2.18	1.66	0.10	0.00	11583.97	25.24
		Total Surface area (m2 g-1)					479.16

45:55 dense to fuzzy ratio:

	volume ratio	% volume	pore width (nm)	density (g/cm3)	mass (g) if have 1 cm3	Surface Area. (m2 g-1)	surface area contribution
dense	45.00	45.00	0.21	1.55	0.70	0.00	0.00
fuzzy	55.00	0.00	0.15	1.40	0.00	39.42	0.00
		0.00	0.38	1.20	0.00	283.36	0.00
		24.00	0.61	1.00	0.24	875.30	210.07
		0.00	0.85	0.80	0.00	1954.67	0.00
		10.00	1.08	0.60	0.06	3660.90	219.65
		15.00	1.20	0.38	0.06	6428.00	365.63
		2.00	1.54	0.20	0.00	9511.64	38.05
		4.00	1.66	0.10	0.00	11583.97	46.34
		Total Surface area (m2 g-1)					879.74

90:10 dense to fuzzy ratio:

	volume ratio	% volume	pore width (nm)	density (g/cm3)	mass (g) if have 1 cm3	Surface Area. (m2 g-1)	surface area contribution
dense	90.00	90.00	0.21	1.55	1.40	0.00	0.00
fuzzy	10.00	0.00	0.15	1.40	0.00	39.42	0.00
		0.00	0.38	1.20	0.00	283.36	0.00
		4.36	0.61	1.00	0.04	875.30	38.19
		0.00	0.85	0.80	0.00	1954.67	0.00
		1.82	1.08	0.60	0.01	3660.90	39.94
		2.73	1.20	0.38	0.01	6428.00	66.48
		0.36	1.54	0.20	0.00	9511.64	6.92
		0.73	1.66	0.10	0.00	11583.97	8.42
				Total Surface area (m2 g-1)			159.95

References

(1) Zhao, Z.; Das, S.; Xing, G.; Fayon, P.; Heasman, P.; Jay, M.; Bailey, S.; Lambert, C.; Yamada, H.; Wakihara, T.; et al. A 3D Organically Synthesized Porous Carbon Material for Lithium-Ion Batteries. *Angew. Chem. - Int. Ed.* **2018**, *57* (37), 11952-11956. DOI: https://doi.org/10.1002/anie.201805924 (acccessed 2023/08/07).
(2) Rowling, A.; Doulcet, J.; Dawson, R.; Tapia-Ruiz, N.; Trewin, A. *Carbon, submitted*.
(3) Dong, C.; Chu, J.; Cao, L.; Cui, F.; Liang, S.; Zhang, X.; Tao, X.; Wang, H.-G.; Zhu, G. A Three-Dimensional Silicon-Diacetylene Porous Organic Radical Polymer. *CCS Chem.* **2022**, *5* (3), 607-615. DOI: 10.31635/ccschem.022.202202351 (acccessed 2023/11/27).
(4) Yang, Z.; Ren, X.; Song, Y.; Li, X.; Zhang, C.; Hu, X.; He, J.; Li, J.; Huang, C. Germanium-Carbdiyne: A 3D Well-Defined sp-Hybridized Carbon-Based Material with Superhigh Li Storage Property. *Energy Environ. Mater.* **2023**, *6* (1), e12269. DOI: https://doi.org/10.1002/eem2.12269 (acccessed 2023/12/01).
(5) Ren, S.; Dawson, R.; Laybourn, A.; Jiang, J.-X.; Khimyak, Y.; Adams, D. J.; Cooper, A. I. Functional conjugated microporous polymers: from 1,3,5-benzene to 1,3,5-triazine. *Polym. Chem.* **2012**, *3* (4), 928-934, 10.1039/C2PY00585A. DOI: 10.1039/C2PY00585A.
(6) Dubois, M.; Guérin, K.; Petit, E.; Batisse, N.; Hamwi, A.; Komatsu, N.; Giraudet, J.; Pirotte, P.; Masin, F. Solid-State NMR Study of Nanodiamonds Produced by the Detonation Technique. *J. Phys. Chem. C* **2009**, *113* (24), 10371-10378. DOI: 10.1021/jp901274f.
(7) He, H.; Riedl, T.; Lerf, A.; Klinowski, J. Solid-State NMR Studies of the Structure of Graphite Oxide. *J. Phys. Chem.* **1996**, *100* (51), 19954-19958. DOI: 10.1021/jp961563t.
(8) Panich, A. M. Solid state nuclear magnetic resonance studies of nanocarbons. *Diam. Relat. Mater.* **2007**, *16* (12), 2044-2049. DOI: https://doi.org/10.1016/j.diamond.2007.07.014.
(9) Brownbill, N. J.; Sprick, R. S.; Bonillo, B.; Pawsey, S.; Aussenac, F.; Fielding, A. J.; Cooper, A. I.; Blanc, F. Structural Elucidation of Amorphous Photocatalytic Polymers from Dynamic Nuclear Polarization Enhanced Solid State NMR. *Macromolecules* **2018**, *51* (8), 3088-3096. DOI: 10.1021/acs.macromol.7b02544.
(10) Jiang, J.-X.; Su, F.; Trewin, A.; Wood, C. D.; Niu, H.; Jones, J. T. A.; Khimyak, Y. Z.; Cooper, A. I. Synthetic Control of the Pore Dimension and Surface Area in Conjugated Microporous Polymer and Copolymer Networks. *J. Am. Chem. Soc.* **2008**, *130* (24), 7710-7720. DOI: 10.1021/ja8010176.
(11) Harris, K. J.; Bernard, G. M.; McDonald, C.; McDonald, R.; Ferguson, M. J.; Wasylishen, R. E. Acetylenic Carbon-13 Chemical Shift Tensors for Diphenylacetylene and (η2-Diphenylacetylene)Pt(PPh3)2: A Solid-State NMR and Theoretical Study. *Inorg. Chem.* **2006**, *45* (6), 2461-2473. DOI: 10.1021/ic051548f.
(12) Karakhanov, E.; Maximov, A.; Terenina, M.; Vinokurov, V.; Kulikov, L.; Makeeva, D.; Glotov, A. Selective hydrogenation of terminal alkynes over palladium nanoparticles within the pores of amino-modified porous aromatic frameworks. *Catal. Today* **2020**, *357*, 176-184. DOI: https://doi.org/10.1016/j.cattod.2019.05.028.

(13) Cataldo, F.; Capitani, D. Preparation and characterization of carbonaceous matter rich in diamond-like carbon and carbyne moieties. *Mater. Chem. Phys.* **1999**, *59* (3), 225-231. DOI: https://doi.org/10.1016/S0254-0584(99)00055-3.
(14) Fukui, K.; Sato, K.; Shiomi, D.; Takui, T.; Itoh, K.; Gotoh, K.; Kubo, T.; Yamamoto, K.; Nakasuji, K.; Naito, A. Electronic structure of a stable phenalenyl radical in crystalline state as studied by SQUID measurements, cw-ESR, and 13C CP/MAS NMR spectroscopy. *Synth. Met.* **1999**, *103* (1), 2257-2258. DOI: https://doi.org/10.1016/S0379-6779(98)00396-8.
(15) Cooper, T. M.; Krein, D. M.; Burke, A. R.; McLean, D. G.; Rogers, J. E.; Slagle, J. E.; Fleitz, P. A. Spectroscopic Characterization of a Series of Platinum Acetylide Complexes Having a Localized Triplet Exciton. *J. Phys. Chem. A* **2006**, *110* (13), 4369-4375. DOI: 10.1021/jp056663q.
(16) Breher, F.; Grunenberg, J.; Lawrence, S. C.; Mountford, P.; Rüegger, H. A Monomeric Organolithium Compound Containing a Free Pyramidal Carbanion in Solution and in the Solid State. *Angew. Chem. - Int. Ed.* **2004**, *43* (19), 2521-2524. DOI: https://doi.org/10.1002/anie.200353308 (acccessed 2023/11/27).
(17) Gu, S.; Wu, S.; Cao, L.; Li, M.; Qin, N.; Zhu, J.; Wang, Z.; Li, Y.; Li, Z.; Chen, J.; et al. Tunable Redox Chemistry and Stability of Radical Intermediates in 2D Covalent Organic Frameworks for High Performance Sodium Ion Batteries. *J. Am. Chem. Soc.* **2019**, *141* (24), 9623-9628. DOI: 10.1021/jacs.9b03467.
(18) Huang, T.-K.; Chi, Y.; Peng, S.-M.; Lee, G.-H.; Wang, S.-L.; Liao, F.-L. Synthesis and Characterization of a (Ketenyl)metal Cluster Complex, an Intermediate in the Oxidative Decarbonation of an Acetylide Ligand. *Organometallics* **1995**, *14* (5), 2164-2166. DOI: 10.1021/om00005a014.
(19) Chisholm, M. H.; Ho, D.; Huffman, J. C.; Marchant, N. S. Reactions between certain trialkoxytungsten alkylidyne complexes and carbon monoxide. Competitive carbon-carbon bond formation leading to alkyne or ketenyl complexes. *Organometallics* **1989**, *8* (7), 1626-1636. DOI: 10.1021/om00109a009.
(20) George, L.; Netsch, K.-P.; Penn, G.; Kollenz, G.; Wentrup, C. Oxoketene–oxoketene, imidoylketene–imidoylketene and oxoketenimine–imidoylketene rearrangements. 1,3-Shifts of phenyl groups. *Org. Biomol. Chem.* **2006**, *4* (3), 558-564, 10.1039/B515917B. DOI: 10.1039/B515917B.
(21) Anderson, J. A.; Lorenz, C. D.; Travesset, A. General purpose molecular dynamics simulations fully implemented on graphics processing units. *J. Comput. Phys.* **2008**, *227* (10), 5342-5359. DOI: https://doi.org/10.1016/j.jcp.2008.01.047.
(22) Glaser, J.; Nguyen, T. D.; Anderson, J. A.; Lui, P.; Spiga, F.; Millan, J. A.; Morse, D. C.; Glotzer, S. C. Strong scaling of general-purpose molecular dynamics simulations on GPUs. *Comput. Phys. Commun.* **2015**, *192*, 97-107. DOI: https://doi.org/10.1016/j.cpc.2015.02.028.
(23) Nguyen, T. D.; Phillips, C. L.; Anderson, J. A.; Glotzer, S. C. Rigid body constraints realized in massively-parallel molecular dynamics on graphics processing units. *Comput. Phys. Commun.* **2011**, *182* (11), 2307-2313. DOI: https://doi.org/10.1016/j.cpc.2011.06.005.
(24) Sarkisov, L.; Bueno-Perez, R.; Sutharson, M.; Fairen-Jimenez, D. Materials Informatics with PoreBlazer v4.0 and the CSD MOF Database. *Chem. Mater.* **2020**, *32* (23), 9849-9867. DOI: 10.1021/acs.chemmater.0c03575.
(25) Sarkisov, L.; Harrison, A. Computational structure characterisation tools in application to ordered and disordered porous materials. *Mol. Simul.* **2011**, *37* (15), 1248-1257. DOI: 10.1080/08927022.2011.592832.
(26) Fayon, P.; Thomas, J. M. H.; Trewin, A. Structure and Properties of a Nanoporous Supercapacitor. *J. Phys. Chem. C* **2016**, *120* (45), 25880-25891. DOI: 10.1021/acs.jpcc.6b08712.
(27) Thomas, J. M. H.; Mollart, C.; Turner, L.; Heasman, P.; Fayon, P.; Trewin, A. Artificial Synthesis of Conjugated Microporous Polymers via Sonogashira–Hagihara Coupling. *J. Phys. Chem. B* **2020**, *124* (33), 7318-7326. DOI: 10.1021/acs.jpcb.0c04850.
(28) Thomas, J. M. H.; Trewin, A. Amorphous PAF-1: Guiding the Rational Design of Ultraporous Materials. *J. Phys. Chem. C* **2014**, *118* (34), 19712-19722. DOI: 10.1021/jp502336a.
(29) Becke, A. D. Density-functional thermochemistry. III. The role of exact exchange. *J. Chem. Phys.* **1993**, *98* (7), 5648-5652. DOI: 10.1063/1.464913 (acccessed 9/19/2023).
(30) Frisch, M. J.; Trucks, G. W.; Schlegel, H. B.; Scuseria, G. E.; Robb, M. A.; Cheeseman, J. R.; Scalmani, G.; Barone, V.; Petersson, G. A.; Nakatsuji, H.; et al. *Gaussian 09*; Gaussian, Inc., 2009.
(31) Krishnan, R.; Binkley, J. S.; Seeger, R.; Pople, J. A. Self-consistent molecular orbital methods. XX. A basis set for correlated wave functions. *J. Chem. Phys.* **2008**, *72* (1), 650-654. DOI: 10.1063/1.438955 (acccessed 11/6/2023).
(32) Lee, C.; Yang, W.; Parr, R. G. Development of the Colle-Salvetti correlation-energy formula into a functional of the electron density. *Phys. Rev. B* **1988**, *37* (2), 785-789. DOI: 10.1103/PhysRevB.37.785.
(33) Stephens, P. J.; Devlin, F. J.; Chabalowski, C. F.; Frisch, M. J. Ab Initio Calculation of Vibrational Absorption and Circular Dichroism Spectra Using Density Functional Force Fields. *J. Phys. Chem.* **1994**, *98* (45), 11623-11627. DOI: 10.1021/j100096a001.
(34) Vosko, S. H.; Wilk, L.; Nusair, M. *Can. J. Phys.* **1980**, *58*, 1200-1211.
(35) Dunning, T. H., Jr.; Hay, P. J. In *Modern Theoretical Chemistry*, Schaefer III, H. F. Ed.; Vol. 3; 1977; pp 1-28.
(36) Hay, P. J.; Wadt, W. R. Ab initio effective core potentials for molecular calculations. Potentials for the transition metal atoms Sc to Hg. *J. Chem. Phys.* **1985**, *82*, 270-283. DOI: 10.1063/1.448799 (acccessed 11/27/2023).

(37) Hay, P. J.; Wadt, W. R. Ab initio effective core potentials for molecular calculations. Potentials for K to Au including the outermost core orbitals. *J. Chem. Phys.* **1985**, *82*, 299-310. DOI: 10.1063/1.448975 (acccessed 11/27/2023).
(38) Wadt, W. R.; Hay, P. J. Ab initio effective core potentials for molecular calculations. Potentials for main group elements Na to Bi. *J. Chem. Phys.* **1985**, *82*, 284-298. DOI: 10.1063/1.448800 (acccessed 11/27/2023).
(39) Cheeseman, J. R.; Trucks, G. W.; Keith, T. A.; Frisch, M. J. A comparison of models for calculating nuclear magnetic resonance shielding tensors. *J. Chem. Phys.* **1996**, *104*, 5497-5509. (acccessed 8/25/2023).
(40) Keith, T. A.; Bader, R. F. W. Calculation of magnetic response properties using atoms in molecules. *Chem. Phys. Lett.* **1992**, *194* (1), 1-8. DOI: https://doi.org/10.1016/0009-2614(92)85733-Q.
(41) Keith, T. A.; Bader, R. F. W. Calculation of magnetic response properties using a continuous set of gauge transformations. *Chem. Phys. Lett.* **1993**, *210* (1), 223-231. DOI: https://doi.org/10.1016/0009-2614(93)89127-4.
(42) Deng, W.; Cheeseman, J. R.; Frisch, M. J. Calculation of Nuclear Spin−Spin Coupling Constants of Molecules with First and Second Row Atoms in Study of Basis Set Dependence. *J. Chem. Theory Comput.* **2006**, *2* (4), 1028-1037. DOI: 10.1021/ct600110u.
(43) Clark, S. J.; Segall, M. D.; Pickard, C. J.; Hasnip, P. J.; Probert, M. I. J.; Refson, K.; Payne, M. C. First principles methods using CASTEP. *Z. für Kristallogr. - Cryst. Mater.* **2005**, *220* (5-6), 567-570. DOI: doi:10.1524/zkri.220.5.567.65075 (acccessed 2023-11-06).
(44) Perdew, J. P.; Burke, K.; Ernzerhof, M. Generalized Gradient Approximation Made Simple. *Phys. Rev. Lett.* **1996**, *77* (18), 3865-3868. DOI: 10.1103/PhysRevLett.77.3865.
(45) Francis, G. P.; Payne, M. C. Finite basis set corrections to total energy pseudopotential calculations. *J. Phys.: Condens. Matter* **1990**, *2* (19), 4395-4404. DOI: 10.1088/0953-8984/2/19/007.
(46) Yates, J. R.; Pickard, C. J.; Mauri, F. Calculation of NMR chemical shifts for extended systems using ultrasoft pseudopotentials. *Phys. Rev. B* **2007**, *76* (2), 024401. DOI: 10.1103/PhysRevB.76.024401.
(47) Bonhomme, C.; Gervais, C.; Babonneau, F.; Coelho, C.; Pourpoint, F.; Azaïs, T.; Ashbrook, S. E.; Griffin, J. M.; Yates, J. R.; Mauri, F.; et al. First-Principles Calculation of NMR Parameters Using the Gauge Including Projector Augmented Wave Method: A Chemist's Point of View. *Chem. Rev.* **2012**, *112* (11), 5733-5779. DOI: 10.1021/cr300108a.
(48) Pickard, C. J.; Mauri, F. All-electron magnetic response with pseudopotentials: NMR chemical shifts. *Phys. Rev. B* **2001**, *63* (24), 245101. DOI: 10.1103/PhysRevB.63.245101.
(49) Monkhorst, H. J.; Pack, J. D. Special points for Brillouin-zone integrations. *Phys. Rev. B* **1976**, *13* (12), 5188-5192. DOI: 10.1103/PhysRevB.13.5188.
